# FBXW5 blunts RIGI/MDA5/IFN signaling to drive immune evasion and chemo-immune resistance in triple-negative breast cancer models

**DOI:** 10.1172/JCI201573

**Published:** 2026-08-25

**Authors:** Xin Li, Tong Chen, Wenjing Zhao, Jiaxing Li, Yifan Shang, Bing Chen, Lijuan Wang, Ning Zhang, Xiaoli Kong, Yiran Liang, Yaming Li, Chen Li, Dianwen Han, Xi Chen, Shan Jiang, Chao Yang, Dan Luo, Tingting Ma, Qifeng Yang

**Affiliations:** 1 Department of Breast Surgery, General Surgery, and; 2 Biological Resource Center, Qilu Hospital of Shandong University, Jinan, Shandong, China.; 3 Research Institute of Breast Cancer, Shandong University, Jinan, Shandong, China.

**Keywords:** Cell biology, Immunology, Oncology, Breast cancer

## Abstract

Triple-negative breast cancer (TNBC), characterized by aggressive behavior and poor prognosis, presents a formidable clinical challenge. Despite guideline endorsement of chemoimmunotherapy as a standard treatment in TNBC, durable responses remain rare, largely due to an immunologically “cold” tumor microenvironment (TME). Through integrated analysis, we identified the F-box protein FBXW5 as a tumor-intrinsic immunosuppressive regulator, whose expression is elevated in immunologically “cold” TNBC and correlates with dismal patient survival. Genetic knockdown of murine *Fbxw5* suppressed tumor growth, reinvigorated CD8^+^ T cell–mediated antitumor immunity, and sensitized TNBC tumors to both single-agent and combined chemoimmunotherapy in preclinical models. Mechanistically, FBXW5 acts within the SKP1/CUL1/F-box protein E3 ligase complex to bind RIGI and MDA5, promoting their K27-linked polyubiquitination and subsequent SQSTM1-mediated autophagic degradation. This process blunts cytosolic RNA sensing and type I interferon signaling, thereby limiting CD8^+^ T cell infiltration and activation. Our findings establish FBXW5 as a master regulator of the “cold” TME, presenting a potential predictive biomarker and actionable therapeutic target for enhancing chemoimmunotherapy in TNBC.

## Abstract 2

FBXW5 helps TNBC tumors evade immunity by destroying RIG-I and MDA5. Targeting FBXW5 overcomes resistance to chemoimmunotherapy.

## Graphical abstract

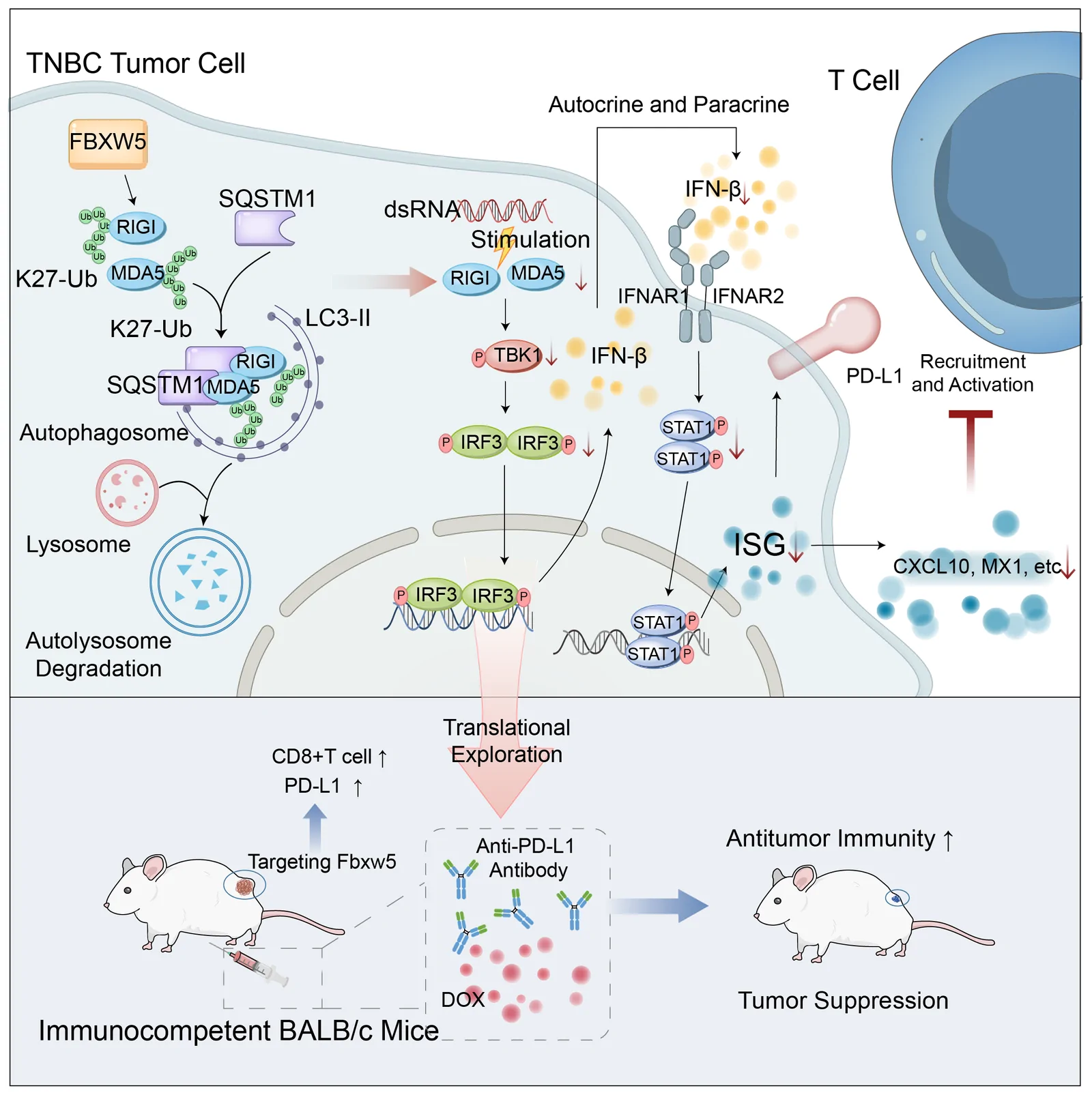

## Introduction

Triple-negative breast cancer (TNBC) is a particularly aggressive subset of breast cancer with a high recurrence rate and poor prognosis, constituting approximately 15%–20% of all breast cancer cases (1). Due to the absence of actionable therapeutic targets, conventional chemotherapy has long been the cornerstone of treatment, yet intrinsic and acquired resistance frequently limits its efficacy (1, 2), necessitating the development of novel therapeutic paradigms. Recently, immune checkpoint blockade (ICB) therapy represented by anti–programmed cell death protein 1/programmed cell death ligand 1 (anti–PD1/PD-L1) has remarkably revolutionized the treatment of certain cancers, such as lung cancer and melanoma (3). Nevertheless, ICB monotherapy has yielded only modest response rates in patients with TNBC, ranging 5%–23% (4–6). Preclinical evidence suggests certain chemotherapeutic drugs (e.g., anthracyclines and taxanes) can act as immunomodulators by inducing immunogenic cell death and priming antitumor immunity, thereby providing a rationale for chemotherapy-ICB combinations (chemoimmunotherapy) (7). Moreover, clinical trials including IMpassion130 (anti–PD-L1/chemotherapy) (8) and KEYNOTE-355 (anti–PD-1/chemotherapy) (9) have established clinically meaningful survival improvements in PD-L1–positive advanced TNBC patients. Chemoimmunotherapy has been formally incorporated into the National Comprehensive Cancer Network guidelines as standard frontline treatment for PD-L1–positive TNBC. However, the phase III IMpassion131 trial yielded discordant results, showing no statistically significant progression-free survival benefit with the addition of atezolizumab (anti–PD-L1) to paclitaxel compared to paclitaxel alone in a similar patient population (10). These contrasting clinical outcomes underscore the critical need to decipher the underlying biological mechanisms driving heterogeneity in treatment response. Elucidating the determinants of sensitivity versus resistance is essential for refining patient stratification, optimizing therapeutic strategies for this recalcitrant disease.

Clinical evidence has established features of the tumor microenvironment (TME) as validated biomarkers predictive of response to chemoimmunotherapy (8, 9, 11). Tumors are broadly categorized as immunologically “cold” or “hot” based on the density and spatial distribution nature of immune cell infiltration (12). Immunologically “hot” tumors are typically characterized by abundant infiltration of T lymphocytes, particularly cytotoxic CD8^+^ T cells, and exhibit favorable responses to the chemoimmunotherapy, while “cold” tumors are marked by sparse CD8^+^ T cell presence and inherent therapeutic resistance (13, 14). Additionally, higher PD-L1 expression typically correlates with enhanced clinical responses to ICB, though its predictive utility remains contingent upon the baseline TME landscape (9, 15). Reflecting this heterogeneity, TNBC molecular subtyping by Bareche et al. revealed distinct immune profiles: the immunomodulatory (IM) subtype exhibits a hot TME enriched with T cell infiltration, whereas the basal-like (BL) subtype presents as an immunologically cold or quiescent phenotype (16–18). Crucially, emerging research has highlighted that these TME architectures are largely orchestrated by tumor-intrinsic factors. Dysregulated signaling pathways and molecular aberrations within tumor cells can modulate key immunologic processes, such as antigen presentation, immune checkpoint expression, and chemokine secretion, thereby shaping an immunosuppressive milieu conducive to immune evasion (19). Consequently, identifying the principal tumor-intrinsic modulators of TME heterogeneity is imperative for reprogramming the cold tumors into the hot state and overcoming chemoimmunotherapy resistance in TNBC.

E3 ubiquitin ligases are pivotal regulators in the remodeling of the TME (20). Among these, the SKP1-CUL1-F-box (SCF) complex, a canonical member of the really interesting new gene (RING) domain E3 ligase family, is indispensable for maintaining cellular proteostasis. This complex comprises 4 core components: a variable F-box protein that recognizes specific substrates, RING box 1 (RBX1) to recruit the E2 ubiquitin-conjugating enzyme, cullin 1 (CUL1) as the structural scaffold, and S-phase kinase associated protein 1 (SKP1) as the adaptor (21). Notably, the F-box proteins typically recognize their substrates in a phosphorylation-dependent manner, ensuring the precise degradation of key regulatory proteins (22). Within this family, the FBXW subfamily, characterized by WD40 repeat domains, has been implicated in modulating antitumor immunity across various cancers. For instance, tumor-intrinsic FBXW7 enhances T cell–mediated tumor clearance by governing the degradation of PD-1 protein in melanoma (23). However, whether FBXW5, another prominent FBXW family member, contributes to the immunological landscape and tumor progression in TNBC remains unexplored. Here, we identify tumor-intrinsic FBXW5 as a key driver of immunologically cold TME and a predictor of poor prognosis in TNBC. We found targeting FBXW5 substantially inhibits TNBC progression and sensitizes tumors to chemotherapy, ICB, or combined chemoimmunotherapy. Mechanistically, we reveal that FBXW5 orchestrates an immunosuppressive milieu by dampening type I IFN signaling mediated by retinoic acid–inducible gene I (RIGI) and melanoma differentiation–associated protein 5 (MDA5) in a phosphorylation-dependent manner. Although activating tumor-intrinsic type I IFN signaling holds immense potential for reinvigorating antitumor immunity, the regulatory mechanisms governing this pathway in TNBC remain poorly understood. Our findings directly address this knowledge gap by uncovering FBXW5 as a central repressor of cytosolic RNA sensing. Ultimately, our study not only provides important insights into the regulatory mechanisms controlling type I IFN pathway activation in TNBC, but also reveals targeting FBXW5 as a therapeutically actionable strategy to overcome chemoimmunotherapy resistance.

## Results

### Tumor-intrinsic FBXW5 is associated with immunologically cold TME and poor prognosis in TNBC.

To systematically identify tumor-intrinsic determinants of the TME in TNBC, we performed an integrated transcriptomic analysis (Figure 1A). We first stratified human TNBC samples from a scRNA-seq dataset (NCBI GEO GSE176078) into immunologically cold and hot subgroups based on CD8^+^ T cell infiltration levels (24). Differential gene expression analysis in the epithelial compartment between 2 groups was conducted using the *FindMarkers* package in R software, identifying 2,931 genes substantially upregulated in epithelial cells of the cold TME (Figure 1, B and C).

To validate these findings in a larger clinical cohort, we applied the BayesPrism deconvolution method to reconstruct tumor epithelial specific gene expression profiles and infer cellular composition from TCGA bulk RNA-seq data, using the scRNA-seq dataset as a reference (Figure 1A). After filtering out uninformative transcripts (e.g., ribosomal and mitochondrial genes; Supplemental Figure 1A; supplemental material available online with this article; https://doi.org/10.1172/JCI201573DS1), the resulting epithelial specific expression matrix showed concordance between bulk and single-cell data (Supplemental Figure 1B). Based on BayesPrism-inferred T cell proportions, we classified 21 TNBC tumors from TCGA as immunologically hot and 86 as cold. Differential expression analysis using *edgeR* identified 1,307 immunologically relevant genes upregulated in epithelial cells of cold tumors (Figure 1C). A crossplatform intersection of the candidates derived from both scRNA-seq and TCGA cohorts yielded 292 overlapping genes consistently associated with an immunologically cold TME.

Using the *survival* and *survminer* R packages, we performed survival analysis on the 292 candidates within TCGA TNBC subcohort (Figure 1A), identifying 32 genes significantly correlated with unfavorable clinical outcomes (Figure 1D). To objectively pinpoint the most biologically relevant and therapeutically actionable oncogenic driver, we implemented a multifaceted scoring system drawing on the systematic target prioritization approach described by Behan et al. (25). Our scoring system encompassed 4 parameters: druggability, prior immunological characterization, publication density, and reported protumorigenic evidence. By aggregating these scores to reflect both translational potential and biological relevance, FBXW5 emerged as the highest priority target, achieving the top overall score among the 32 prognostic candidates (Supplemental Table 1).

Although FBXW5 has previously been implicated as an oncogene in various contexts (26), its potential role in orchestrating the cancer immune landscape remains largely uncharacterized. Our scRNA-seq analysis demonstrated that both the transcript levels of human *FBXW5* and the proportion of FBXW5-positive epithelial cells were elevated in tumors with low T cell infiltration compared with those with high T cell infiltration (Figure 1, E and F). These results were subsequently validated in TNBC samples from the Qilu Hospital cohort using immunohistochemical (IHC) analysis. To ensure the technical rigor of the IHC assay, we first established the specificity of the FBXW5 antibody using FFPE cell blocks derived from TNBC cell lines with manipulated FBXW5 expression. As shown in Supplemental Figure 1C, the IHC signal intensity precisely mirrored the modulation of FBXW5 protein levels, confirming the reliability of our staining protocol. In line with our bioinformatic predictions, IHC analysis of the Qilu cohort demonstrated that FBXW5-low tumors manifested higher CD8^+^ T cell infiltration (Figure 1G and Supplemental Figure 1, D and E). Furthermore, elevated epithelial FBXW5 expression predicted poor prognosis in the TNBC subset from both TCGA and Qilu cohorts (Figure 1, H and I). Extended analysis via the Breast Cancer Integrative Platform (http://www.omicsnet.org/bcancer/database) confirmed that *FBXW5* was upregulated in TNBC compared with non-TNBC (Supplemental Figure 1F). Notably, further subtyping analysis revealed that *FBXW5* expression was consistently higher across the BL, mesenchymal-like (MSL), IM, and luminal androgen receptor (LAR) subtypes (27) than in non-TNBC while exhibiting lower expression in the M subtype (Supplemental Figure 1G). Furthermore, *FBXW5* expression positively associated with advanced clinical stage and pathological grade (Supplemental Figure 1H). Taken together, these findings identify FBXW5 as a clinically relevant biomarker that closely correlates with tumor progression and immune exclusion in TNBC, prompting further investigation into its functional contributions.

### FBXW5 suppresses CD8^+^ T cell–mediated antitumor immunity and promotes TNBC progression.

To delineate the functional role of FBXW5 in TNBC, we first established stable murine 4T1 cell lines with murine *Fbxw5* overexpression or knockdown, with their efficiencies validated by RT-qPCR and Western blotting (Supplemental Figure 2, A and B). The sequences of all nucleic acids used in this study are provided in Supplemental Table 2. We then employed mass cytometry by time of flight (CyTOF) to dissect the immunological landscape of *Fbxw5*-knockdown and control 4T1 tumors harvested from immunocompetent BALB/c mice (Figure 2A). A panel of 32 metal-conjugated antibodies (Supplemental Table 3) was used to evaluate the alterations of major immune cell populations. We identified 35 distinct clusters, which were further categorized into 17 major immune cell subpopulations based on lineage marker expression profiles (Figure 2B and Supplemental Figure 2C). In comparison with the control group, murine *Fbxw5*-knockdown tumors exhibited a significant enrichment of effector CD8^+^ T cells and conventional CD4^+^ T cells (Figure 2C), whereas other immune populations, including regulatory T cells, remained unaffected (Supplemental Figure 2, D–F).

To determine whether these T cell alterations are functionally essential for FBXW5-mediated modulation of TNBC progression, we evaluated tumor growth across different models. While *Fbxw5* manipulation had no impact on cancer cell proliferation in vitro (Supplemental Figure 3, A–C) or on tumor growth in T cell–deficient BALB/c nude mice, *Fbxw5* overexpression markedly accelerated tumor progression in immunocompetent hosts (Figure 2, D–F, and Supplemental Figure 3, D and E). Collectively, these findings demonstrate that FBXW5 promotes TNBC progression primarily by orchestrating T cell–mediated immune evasion rather than directly driving intrinsic cell proliferation.

Given that cytotoxic CD8^+^ T cells are the principal effectors of antitumor immunity and their intratumoral density is a critical determinant of clinical response (28–30), we focused our subsequent functional and mechanistic studies on this specific lineage. In agreement with the CyTOF data, flow cytometry and immunofluorescence (IF) analyses showed that murine *Fbxw5* knockdown substantially enhanced CD8^+^ T cell infiltration into tumors, while its overexpression exerted the opposite effect (Figure 2, G and H, and Supplemental Figure 4, A and B). We further examined the impact of tumor FBXW5 on CD8^+^ T cell functions in vitro. Conditioned medium from *FBXW5*-overexpressing MDA-MB-468 cells significantly impaired human CD8^+^ T cell migration (Figure 2I). Additionally, in coculture systems, *FBXW5*-overexpressing TNBC cells diminished the cytolytic potency of CD8^+^ T cells, evidenced by reduced production of key effector molecules, including IFN-γ, TNF-α, and granzyme B (GZMB), and a concomitant reduction in tumor cell apoptosis (Figure 2, J–L).

To assess the chemotactic regulation underlying effector T cell recruitment, we quantified CXCL9 and CXCL10 levels, pivotal chemokines driving T cell trafficking (31–33), in conditioned medium. ELISA results revealed that *FBXW5* overexpression significantly downregulated the secretion of both chemokines (Supplemental Figure 4C), indicating that FBXW5-mediated remodeling of the chemokine landscape hinders T cell migration. Taken together, the above findings demonstrate that FBXW5 facilitates immune evasion and TNBC progression predominantly by impairing the recruitment and effector functions of CD8^+^ T cells.

### FBXW5 blunts cytosolic dsRNA-induced type I IFN signaling.

To elucidate the molecular mechanisms underlying the oncogenic roles of FBXW5 in TNBC, we performed high-throughput RNA-seq analysis using human (MDA-MB-468, *FBXW5*-overexpressing) and murine (4T1, *Fbxw5*-overexpressing) TNBC cell lines, with the corresponding empty vector transfection serving as a control (Supplemental Figure 4D). Gene Ontology (GO) enrichment analysis revealed that *FBXW5* overexpression was closely associated with suppressed type I IFN signaling and impaired T cell activation (Figure 2M and Supplemental Figure 4E). These results suggest that FBXW5 antagonizes the type I IFN axis, which serves as a master regulator of T cell recruitment and the development of an inflamed TME. This signaling cascade is typically initiated by pattern recognition receptors, such as RIGI, MDA5, or STING, leading to interferon regulatory factor 3/7–mediated (IRF3/7-mediated) production of type I IFN and the subsequent induction of ISGs, including the pivotal T cell–attracting chemokines CXCL9, CXCL10, and CXCL11 (34).

To determine whether our findings apply broadly across diverse TNBC subtypes, we evaluated endogenous FBXW5 protein expression levels in a panel of TNBC cell lines representing various molecular subtypes (27). We observed that FBXW5 was more abundant in the LAR subtype cell line, MDA-MB-453, compared with other subtypes (Supplemental Figure 4F). Consequently, MDA-MB-453 was selected as a loss-of-function model for human *FBXW5* knockdown, while MDA-MB-468 (BL subtype) and BT549 (MSL subtype) cell lines, which exhibited lower endogenous expression, were employed for gain-of-function experiments. Consistent with the RNA-seq data, RT-qPCR assays in these models demonstrated an inverse correlation between *FBXW5* expression and ISG transcript levels (Supplemental Figure 4, G and H). Additionally, overexpression of *FBXW5* led to substantially reduced phosphorylation of TBK1, IRF3, and STAT1 (key signaling nodes in type I IFN pathway), without altering their baseline protein expression. Conversely, *FBXW5* knockdown profoundly augmented the phosphorylation of these effectors (Supplemental Figure 5A). Moreover, FBXW5 inhibited type I IFN signaling in a dose-dependent manner (Supplemental Figure 4I), underscoring its pivotal role in attenuating this pathway.

To assess the functional contribution of type I IFN signaling in FBXW5-driven tumor progression, we treated tumor-bearing immunocompetent BALB/c mice with an interferon-α/β receptor 1–blocking (IFNAR1-blocking) monoclonal antibody (anti-IFNAR1) or a control IgG. IFNAR1 blockade markedly accelerated tumor growth and reversed the tumor-suppressive effect of *Fbxw5* knockdown, without influencing mouse body weight (Figure 2N and Supplemental Figure 5B). Critically, anti-IFNAR1 treatment neutralized the enhancement of CD8^+^ T cell infiltration and activation triggered by *Fbxw5* knockdown (Figure 2O and Supplemental Figure 5, C and D). Collectively, the above results underscore an indispensable role for intact IFN signaling in mediating FBXW5-dependent tumor progression.

In light of the central role of nucleic acid sensing in activating type I IFN and antitumor immunity, we systematically interrogated how FBXW5 influences specific nucleic acid–sensing cascades. We employed 3 in vitro models: (a) intracellular poly(I:C) transfection to recapitulate cytosolic dsRNA sensing, where high–molecular weight (HMW) poly(I:C) is preferentially recognized by MDA5 and low–molecular weight (LMW) poly(I:C) by RIGI (35); (b) extracellular LMW poly(I:C) treatment to simulate TLR3-mediated endosomal dsRNA recognition; and (c) intracellular HT-DNA transfection to model cytosolic DNA sensing (36). Notably, FBXW5 selectively attenuated intracellular HMW or LMW poly(I:C)-induced expression of *IFNB1*, *CXCL10*, *ISG15*, *IFI44*, and *MX1* mRNAs while exhibiting a negligible impact on responses stimulated by extracellular dsRNA or cytosolic DNA (Supplemental Figure 5, E and F, and Supplemental Figure 6, A–C). Conversely, human or murine *FBXW5* knockdown markedly sensitized TNBC cells to cytosolic dsRNA, as manifested by the potentiated induction of ISGs and augmented downstream signaling (Supplemental Figure 6, D and E). Western blotting and ELISA analyses further confirmed FBXW5-mediated suppression of intracellular HMW or LMW poly(I:C)-induced type I IFN signaling pathway (Figure 3A and Supplemental Figure 5, G–K). Together, these data identify FBXW5 as a selective negative regulator of the cytosolic RNA-sensing pathway in TNBC.

### FBXW5 modulates type I IFN signaling via physical interaction with RIGI and MDA5.

The aberrant accumulation of cytosolic dsRNA activates both cytoplasmic RIGI-like receptors (RLRs; predominantly RIGI and MDA5) and endosomal TLR3 (36, 37). To pinpoint the specific sensors through which FBXW5 exerts its inhibitory effect, we initially performed single-gene knockdown experiments. Interestingly, individual depletion of *RIGI*, *MDA5*, or *TLR3* failed to rescue FBXW5-mediated suppression of HMW/LMW poly(I:C)-induced *CXCL10* expression (Figure 3B and Supplemental Figure 7, B and C) or TBK1/IRF3/STAT1 phosphorylation (Figure 3C and Supplemental Figure 7A). However, concurrent silencing of *RIGI* and *MDA5* completely abrogated FBXW5’s inhibitory capacity (Figure 3, B and C, and Supplemental Figure 7, A–C). These findings establish that FBXW5 requires the cooperative involvement of both RIGI and MDA5 to suppress type I IFN signaling, while excluding TLR3 as a key mediator.

To further delineate the precise regulatory node of FBXW5 within the RIGI/MDA5/type I IFN signaling axis, we co-overexpressed *FBXW5* with key mediators across various signaling cascades in HEK293T cells, followed by dual luciferase reporter assays. Notably, FBXW5 specifically blunted *IFNB1* promoter activation, elicited by constitutively active RIGI-N218 and MDA5-N315 truncations, while exhibiting no inhibitory effect on downstream effectors, including MAVS, TBK1, IKBKE, IRF3-5D, or STAT1 (Figure 3D). This mechanistic specificity was further corroborated by co-immunoprecipitation (co-IP) assays revealing obvious binding of FBXW5 to RIGI and MDA5 but not to downstream signaling components (Supplemental Figure 7D). These complementary approaches converge to indicate that FBXW5 exerts its immunoregulatory function by targeting the upstream sensors RIGI and MDA5, rather than intervening at later stages of the type I IFN signaling cascade. Consistent with their functional interplay, IF microscopy‌ and proximity ligation assays (PLAs) revealed that FBXW5 colocalized with both RIGI and MDA5 in the cytosolic compartment (Figure 3E and Supplemental Figure 7E). To further investigate the interactions between FBXW5 and RIGI/MDA5, ‌we performed mass spectrometry (MS)‌ and confirmed the presence of RIGI and MDA5 in the immunoprecipitate enriched by FBXW5-specific antibodies (Figure 3F). This result was corroborated through reciprocal endogenous and exogenous reciprocal co-IP assays‌, which confirmed robust physical interactions between FBXW5 and both RLR sensors (Supplemental Figure 7F). Besides, the interactions were markedly potentiated following intracellular HMW or LMW poly(I:C) stimulation (Figure 3G and Supplemental Figure 7G).

Then, truncations of FBXW5 were constructed to map its functional interaction domains (Figure 3H). Co-IP assays demonstrated that the amino acid 133–466 region of FBXW5 mediated its binding to both RIGI and MDA5 (Figure 3I). Crucially, deletion of *FBXW5*’s 133–466 region (Δ133–466) completely abolished FBXW5’s ability to suppress HMW/LMW poly(I:C)-induced type I IFN signaling (Figure 3J and Supplemental Figure 7H). Structurally, RIGI and MDA5 share a conserved structural architecture: 2 caspase activation and recruitment domains (CARDs) at the N-terminus essential for initiating downstream signaling cascades, a central DExD/H-box RNA helicase domain that enables ATP-dependent RNA unwinding, and a C-terminal regulatory domain involved in ligand recognition and autoinhibition (38). Intriguingly, FBXW5 selectively targeted the N-terminus containing CARDs of RIGI (N218) while engaging the MDA5 protein across all structural domains (Figure 3, K–M). These above domain-specific engagement patterns were further supported by structural predictions using AlphaFold3 modeling (Figure 3N). Together, these findings suggest that FBXW5 acts as a direct molecular brake on the type I IFN signaling pathway via direct physical interactions with RIGI and MDA5.

### FBXW5 orchestrates the autophagic degradation of RIGI and MDA5.

Given the physical interaction between FBXW5 and RIGI/MDA5, we next assessed whether the FBXW5 governs the protein abundance and metabolic stability of RIGI and MDA5 in TNBC. We found FBXW5 decreased RIGI and MDA5 proteins levels in a dose-dependent manner (Figure 4A). Cycloheximide (CHX) treatment demonstrated that *FBXW5* knockdown substantially stabilized RIGI and MDA5 proteins (Supplemental Figure 8, A and B), whereas *FBXW5* overexpression dramatically accelerated their turnover (Figure 4, B and C, and Supplemental Figure 8C), establishing FBXW5 as a critical posttranslational regulator of RIGI/MDA5 stability. Notably, *FBXW5* overexpression also led to a decline in mRNA levels of *RIGI* and *MDA5*, an effect that was abrogated by knockdown of downstream core components, such as *TBK1*, *IRF3*, and *STAT1* (Supplemental Figure 8, D and F). The suppressive effect of FBXW5 on *CXCL10* expression was similarly rescued by *TBK1*, *IRF3*, or *STAT1* silencing (Supplemental Figure 8F). However, FBXW5-induced reduction of RIGI/MDA5 protein abundance persisted even under *TBK1*/*IRF3*/*STAT1*–knockdown conditions (Supplemental Figure 8E). Since RIGI and MDA5 also belonged to ISGs subject to transcriptional amplification (39), these findings suggest that FBXW5 directly destabilizes RIGI/MDA5 proteins, whereas its regulatory effect on their transcript levels occurs indirectly through a positive feedback loop downstream of type I IFN signaling.

Intracellular protein degradation primarily occurs through the ubiquitin-proteasome system and the autophagy-lysosomal pathway (40). To delineate the specific route underlying the FBXW5-mediated degradation of RIGI and MDA5, we employed specific pharmacological inhibitors targeting these distinct pathways. Intriguingly, lysosomal inhibitors (chloroquine, leupeptin, bafilomycin A1) and the autophagy-specific blocker 3-methyladenine (3-MA) rescued FBXW5-induced RIGI/MDA5 degradation, whereas the proteasome inhibitor MG132 exerted no discernible effect (Figure 4D and Supplemental Figure 8, G and H). Knockdown of human autophagy-related gene 5 (*ATG5*), a core component of the autophagic machinery, also abolished FBXW5-mediated degradation (Figure 4E). Conversely, autophagy inducers (EBSS, rapamycin) synergized with *FBXW5* overexpression markedly enhanced RLR turnover in TNBC cells (Figure 4, F and G), collectively identifying autophagy as the requisite pathway for FBXW5-mediated RLR destabilization. Given the pivotal role of LC3B in autophagosome formation and the recruitment of cargo receptors linked to ubiquitinated substrates (41), we investigated whether FBXW5 facilitated the molecular bridging between LC3B and RIGI/MDA5. Co-IP experiments in HEK293T cells revealed FBXW5 substantially enhanced LC3B binding to both RIGI and MDA5 (Figure 4H). Importantly, IF and PLAs demonstrated that *FBXW5* overexpression and EBSS treatment synergistically promoted the cytosolic colocalization of LC3B with RIGI/MDA5 (Figure 4I and Supplemental Figure 8I), establishing a functional link between FBXW5 and autophagy. In summary, our data reveal that FBXW5 selectively promotes RIGI and MDA5 degradation through an autophagy-lysosomal pathway.

### FBXW5 targets RIGI and MDA5 for K27-linked ubiquitination.

Ubiquitination serves as a critical posttranslational modification that directs the selective autophagic clearance of cellular substrates. We observed that ubiquitin conjugation to both RLRs was dramatically enhanced by *FBXW5* overexpression, whereas deletion of the RLR-binding domain (residues 133–466 in FBXW5) completely abolished this enhancement (Figure 5A), establishing that a direct interaction between FBXW5 and RLRs is functionally required. Besides, FBXW5 specifically promoted K27-linked ubiquitination of RLRs, with no effect on other linkage types (K6, K11, K29, K33, K48, K63) (Figure 5, B and C). To rigorously validate this linkage specificity, we employed a panel of linkage-restricted ubiquitin mutants (lysine-to-arginine; K-to-R). Consistent with our initial findings, FBXW5-mediated ubiquitination of RIGI and MDA5 was abolished only by the K27R mutant, remaining intact with others (K6R/K11R/K29R/K33R/K48R/K63R) (Figure 5, D and E). Together, these results provide compelling evidence that FBXW5 specifically enhances K27-linked ubiquitination of RIGI and MDA5.

Domain-mapping experiments next established that FBXW5-driven ubiquitination of RIGI and MDA5 required their N-terminal CARD-containing regions (Supplemental Figure 9A). To precisely identify FBXW5-dependent ubiquitination sites on RLRs, we generated a series of RLR mutants with K-to-R substitutions at specific lysines within their N-termini that were previously reported as ubiquitination sites (Figure 5F) (41–43). Our screening identified Lys193 (K193) of RIGI and Lys235 (K235) of MDA5 as the requisite residues, as their individual mutations completely disrupted FBXW5-mediated ubiquitination (Figure 5, G and H). Importantly, the RIGI K193R and MDA5 K235R mutants exhibited remarkable resistance to FBXW5-mediated downregulation, implicating these specific lysine residues as critical determinants for ubiquitination-dependent degradation enhanced by FBXW5 (Supplemental Figure 9B).

As a canonical F-box protein, FBXW5 serves as the substrate recognition subunit of the SCF E3 ubiquitin ligase complex while CUL1 and RBX1 orchestrate ubiquitin transfer via E2 enzyme recruitment and SKP1 acts as the adaptor bridging with the F-box domain of FBXW5 (Figure 5I) (21). To establish whether SCF complex integrity is essential for FBXW5 function, we first performed targeted knockdown of core SCF components (CUL1, RBX1, or SKP1) using siRNAs. Depletion of these core subunits led to the stabilization of RIGI and MDA5 proteins and restored downstream type I IFN signaling (Figure 5J and Supplemental Figure 9, C–F). To further validate that FBXW5 acts through the SCF ubiquitin ligase complex, we engineered FBXW5 deletion mutants lacking the F-box domain (FBXW5-ΔF) or C-terminal region (FBXW5-ΔC, as a control). Consistently, deletion of the F-box domain (but not ΔC) completely abolished FBXW5’s capacity to regulate RIGI and MDA5 and their downstream phosphorylated signaling effectors (Supplemental Figure 9G), as well as their polyubiquitination process (Figure 5, K and L).

Typically, F-box proteins recognize substrates in a phosphorylation-dependent manner, ensuring the stringent regulation of SCF complex-mediated ubiquitination (22). To determine whether FBXW5-RLR interaction also adheres to this paradigm, we first employed staurosporine, a broad-spectrum kinase inhibitor, to induce global dephosphorylation in MDA-MB-468 cells. Strikingly, dephosphorylation mediated by staurosporine markedly disrupted the physical association between FBXW5 and RLRs, while attenuating FBXW5-mediated signaling modulation and RLR ubiquitination (Supplemental Figure 10, A–C). To pinpoint the specific phosphorylation sites requisite for FBXW5-mediated regulation, we generated mutants within the N-terminal CARDs of RIGI and MDA5 to mimic either constitutive phosphorylation or dephosphorylation at previously reported residues (44, 45). We identified Ser8 of RIGI and Ser88 of MDA5 as critical residues: phospho-dead mutations (S8A, S88A) abrogated FBXW5-mediated ubiquitination, whereas phospho-mimetic mutations (S8E, S88E) enhanced it (Figure 5, M and N, and Supplemental Figure 10D). Importantly, dephosphorylation-mimetic RLR mutants were largely refractory to FBXW5-mediated downregulation, whereas those phospho-mimetic mutants were more susceptible to FBXW5-induced degradation, an effect further exacerbated by EBSS treatment (Supplemental Figure 10, E and F). Taken together, these findings demonstrated that FBXW5 selectively promotes K27-linked ubiquitination of RIGI/MDA5 in a phosphorylation-dependent manner, requiring the functional integrity of the SCF complex.

### SQSTM1 acts as the cargo receptor recognizing FBXW5-ubiquitinated RLRs for autophagy.

Autophagic degradation is a highly selective process, contingent upon the recognition of ubiquitinated substrates’ recognition by cargo receptors (41). To identify the specific cargo receptor mediating FBXW5-dependent degradation of RIGI and MDA5, we examined the interactions between RIGI/MDA5 and various autophagy receptors — including sequestosome 1 (SQSTM1; also known as p62), NDP52, OPTN, TOLLIP, NBR1, and BNIP. Only SQSTM1 exhibited robust binding to both RIGI and MDA5 (Figure 6A). Subsequent experiments revealed that FBXW5 also physically associates with SQSTM1 (Figure 6B), an interaction further corroborated by MS analysis (Supplemental Figure 10H). Notably, *FBXW5* overexpression markedly enhanced the SQSTM1/RIGI/MDA5 interaction under basal conditions, with further amplification observed following poly(I:C) or EBSS treatment (Figure 6, C and D, and Supplemental Figure 10G). These results suggest that FBXW5 serves as a scaffold protein bridging RLRs to SQSTM1, facilitating their autophagic clearance. To formally validate the requirement for SQSTM1, specific siRNAs targeting *SQSTM1* were designed. Importantly, *SQSTM1* silencing completely abolished EBSS-induced autophagic degradation of RIGI and MDA5 (Figure 6E). The shortened half-life and enhanced autophagic degradation of RLR proteins driven by FBXW5 were also abolished upon *SQSTM1* silencing (Figure 6, F and G).

Then we interrogated the functional architecture of SQSTM1 responsible for its interaction with RLRs. The SQSTM1 protein contains 3 functionally critical domains: a C-terminal ubiquitin-associated (UBA) domain for recognizing ubiquitinated substrates, an LC3-interacting region (LIR) for autophagosomal membrane anchoring, and an N-terminal PB1 domain enabling self-oligomerization. Based on co-IP assays with engineered *SQSTM1* truncation mutants (Figure 6H), we found that deletion of the UBA, LIR, or PB1 domain each partially impaired the interaction of SQSTM1 with RLRs, with deletion of the UBA domain exhibiting the most pronounced effect (Figure 6I). Furthermore, reexpression of full-length *SQSTM1* restored FBXW5-driven RLR downregulation in *SQSTM1*-knockdown cells, but a mutant lacking the UBA domain (SQSTM1-ΔUBA) failed to restore this effect (Figure 6J). These results demonstrate the UBA domain of SQSTM1 is particularly essential for FBXW5-induced autophagic degradation of RLR proteins. Collectively, our findings establish SQSTM1 as the autophagy receptor mediating FBXW5-directed degradation of RIGI and MDA5.

### FBXW5 inhibits chemotherapy efficacy by blunting type I IFN signaling.

Accumulating evidence demonstrates that chemotherapeutic agents, such as doxorubicin (DOX), can activate type I IFN signaling via inducing the release of damage-associated molecular patterns (46). We found FBXW5 substantially attenuated DOX-induced type I IFN signaling (Figure 7A and Supplemental Figure 11A). Simultaneous knockdown of *RIGI* and *MDA5* significantly weakened the DOX-induced activation of type I IFN pathway and eliminated FBXW5’s regulatory effects (Figure 7, B and C). Similarly, pharmacological blockade of IFNAR1 with anifrolumab not only attenuated DOX-induced ISG expression and STAT1 phosphorylation but also completely abolished FBXW5’s regulatory effects (Figure 7, D and E, and Supplemental Figure 11B). These findings confirm that FBXW5 dampens the immunomodulatory potential of chemotherapy by inhibiting RIGI/MDA5-dependent type I IFN signaling.

Consistent with established associations between RLR deficiency and chemoresistance in TNBC (47, 48), we observed that *FBXW5* overexpression significantly increased the half-maximal inhibitory concentration (IC_50_) of DOX and conferred protection against DOX-induced cell apoptosis in TNBC (Supplemental Figure 11, C and D). This protective effect was abolished by either dual *RIGI/MDA5* knockdown or anifrolumab treatment (Supplemental Figure 11D and Figure 7F). Ectopic expression of *RIGI* or *MDA5* partially reversed FBXW5-induced chemoresistance in TNBC cells (Supplemental Figure 11E). Given drug efflux mediated by ATP binding cassette subfamily B member 1 (ABCB1; also known as P-GP) drives DOX resistance, we investigated whether FBXW5 influences this pathway. Western blot analysis revealed that FBXW5 manipulation did not alter P-gp expression in either MDA-MB-468 or MDA-MB-453 cells (Supplemental Figure 11F). Furthermore, flow cytometry revealed *FBXW5* overexpression led to no marked changes in intracellular DOX accumulation or efflux (Supplemental Figure 11, G and H). These results dispel the possibility that FBXW5 influences drug efflux capacity, thereby underscoring that the suppression of RLR-mediated type I IFN signaling acts as the key mediator for FBXW5-induced chemoresistance.

To validate these findings in vivo, immunocompetent BALB/c mice or immunodeficient nude mice bearing subcutaneous 4T1 TNBC tumors (genetically engineered for stable murine *Fbxw5* knockdown or overexpression) were randomized to receive intraperitoneal injections of PBS or DOX (Figure 7G). Corroborating our in vitro observations, *Fbxw5* knockdown significantly sensitized TNBC tumors to DOX treatment in both mouse models, despite having a negligible impact on baseline tumor growth under vehicle conditions (Figure 7H and Supplemental Figure 11, I–K). By contrast, *Fbxw5* overexpression conferred marked chemoresistance, as evidenced by accelerated tumor progression and significantly larger final tumor burdens following DOX treatment compared with the respective control groups (Figure 7I and Supplemental Figure 11L). No adverse effects on body weight were observed upon murine *Fbxw5* overexpression or knockdown (Supplemental Figure 11M).

Further dissection of the TME in BALB/c mice revealed that DOX treatment typically elicits a robust influx and functional activation of CD8^+^ cytotoxic T cells. However, this chemotherapy-induced immunological invigoration was substantially blunted by murine *Fbxw5* overexpression (Figure 7J). Conversely, murine *Fbxw5*-knockdown tumors exhibited a remodeled, immunologically hot TME characterized by amplified CD8^+^ T cell recruitment and superior effector potency (Figure 7K). Collectively, these findings demonstrate that FBXW5 orchestrates the chemotherapy response through a bimodal RLR-dependent mechanism: (a) conferring tumor cell–intrinsic apoptosis resistance and (b) antagonizing the immunostimulatory effect of chemotherapy by extinguishing cytotoxic T cell–mediated antitumor immunity within the TME.

### FBXW5 knockdown improves the efficacy of ICB and chemoimmunotherapy.

Recent studies have demonstrated that chemoimmunotherapy offers clinical benefit in patients with TNBC, particularly in those exhibiting elevated CD274 (encoding PD-L1) expression in tumor cells or immune cells (49, 50). Notably, as a member of ISGs, PD-L1 expression can be driven by chemotherapy-induced type I IFN signaling (51). Therefore, we hypothesized that FBXW5-mediated modulation of type I IFN pathway might consequently govern PD-L1 expression in TNBC. RT-qPCR demonstrated murine *Fbxw5* overexpression suppressed poly(I:C)-induced *Cd274* mRNA expression, while knockdown enhanced its induction (Supplemental Figure 11N). Flow cytometry of human TNBC cells validated this regulation at the protein level (Supplemental Figure 11O). Critically, *FBXW5* overexpression attenuated DOX-induced elevation of membrane-bound PD-L1 (Figure 8A). In vivo studies further confirmed that murine *Fbxw5* knockdown potentiated DOX-induced cell surface PD-L1 elevation, whereas murine *Fbxw5* overexpression effectively counteracted this effect (Figure 8, B and C).

These findings demonstrate that *Fbxw5* knockdown not only remodels the tumor immune microenvironment toward a T cell–inflamed phenotype in TNBC but also markedly upregulates intratumoral PD-L1 expression. This dual effect simultaneously addresses 2 major limitations of ICB therapy: the paucity of tumor-specific antigens and insufficient immune cell infiltration (12, 29, 52, 53). To assess the translational potential, we evaluated whether murine *Fbxw5* knockdown enhanced ICB or chemoimmunotherapy responsiveness. While anti–PD-L1 monotherapy showed negligible antitumor activity compared with isotype control IgG2b, *Fbxw5* knockdown or DOX treatment markedly potentiated anti–PD-L1 therapeutic efficacy in 4T1 tumors. Notably, *Fbxw5* knockdown synergized with chemoimmunotherapy achieved superior tumor growth inhibition and substantially reduced terminal tumor burden (Figure 8, D–F, and Supplemental Figure 11P). Flow cytometry analysis revealed that *Fbxw5* knockdown augmented DOX-induced PD-L1 surface expression (Figure 8G) and amplified intratumoral CD8^+^ T cell infiltration and activation (Figure 8H). Collectively, these findings demonstrate that FBXW5 inhibition synergizes with ICB or chemoimmunotherapy to evoke powerful immunogenic activation for preventing tumor progression, offering a promising strategy to overcome immunotherapy resistance in TNBC.

## Discussion

Although ICB has revolutionized cancer treatment by reinvigorating antitumor CD8^+^ T cells, primary resistance persists in most patients with TNBC (54). Chemoimmunotherapy has been deployed to potentiate ICB efficacy, especially in PD-L1–high cohorts, but its clinical responses remain curtailed by immunologically cold microenvironments with poor T cell infiltration (55). Deciphering drivers that underpin this refractory state is critical for therapeutic innovation. Here, we identify the E3 ubiquitin ligase FBXW5 as a key molecular determinant of cold TME and immune evasion in TNBC. Mechanistically, tumor-intrinsic FBXW5 targets the RNA sensors RIGI and MDA5 for lysosomal degradation, thereby precipitating the collapse of the type I IFN signaling axis and subsequent immune evasion. Targeting FBXW5 potently activates CD8^+^ T cell–mediated immunity, retards tumor progression, and sensitizes TNBC to PD-L1 blockade and chemoimmunotherapy. Clinically, elevated levels of tumor-intrinsic FBXW5 in patients with TNBC patients correlates with diminished intratumoral T cell infiltration and dismal prognosis. These findings reveal FBXW5 as a potential molecular biomarker and a therapeutic target to reverse ICB and chemoimmunotherapy resistance in TNBC.

Based on the distribution and abundance of cytotoxic immune cells within the TME, tumors can be classified into 3 dominant immunophenotypes: immune-inflamed, immune-excluded, and immune-desert (19, 56, 57). Inflamed tumors exhibit 3 hallmark immunogenic features: robust infiltration of T cells, especially activated CD8^+^ T cells, upregulated PD-L1 expression on tumor and immune cells, and elevated tumor mutational burden, collectively indicative of an immunologically active microenvironment conducive to ICB response (58). Conversely, immune-excluded or immune-desert phenotypes exhibit minimal immune engagement and poor responses to ICB (58, 59). As a highly heterogenous disease, TNBC comprises different molecular subtypes with distinct TME landscapes. According to the refined molecular subtyping system established by Bareche et al., the IM subtype displays robust antitumor immune response signatures with fully inflamed T cell distribution. By contrast, the BL tumor typically manifests as an immunologically cold state with metabolic aberrations and dismal clinical outcomes (16–18). To identify tumor-intrinsic regulators correlated to both TME and clinical prognosis in TNBC, we leveraged the scRNA-seq analysis and BayesPrism algorithm to reconstruct epithelial cell–specific expression profiles as previously described (60, 61). BayesPrism is an advanced deconvolution method to simulate cell type–specific gene expression profiles and cellular composition from bulk RNA-seq, with superior performance compared with existing methods in benchmark studies (60, 62). Through this integrated transcriptomic analysis, DEGs in tumor epithelial cells were identified between the immunologically hot and cold TNBC tumors. Our screening pinpointed that the E3 ligase FBXW5 is markedly overexpressed in immunologically cold TNBC tumors, and its tumor-intrinsic upregulation predicted unfavorable prognosis, suggesting a potential role in TME reprogramming and immune evasion.

As a core component of the SCF (SKP1/CUL1/F-box) E3 ligase complex, FBXW5 governs protein homeostasis and signal transduction by mediating substrate ubiquitination (21). Although the biological scope of FBXW5 remains underexplored, emerging evidence has characterized it as a cell-autonomous driver of progression across various malignancies. For instance, FBXW5 promotes non–small cell lung cancer progression by targeting the RhoGAP protein DLC1 for degradation (26) and facilitates hepatocellular carcinoma progression via AQP3 regulation (63). Similarly, its orchestration of LATS1 ubiquitination and degradation has been shown to accelerate gastric cancer development (64). However, our functional assays — including soft agar, cell counting kit-8, and MTT studies — did not detect a significant impact of FBXW5 on TNBC cell proliferation or anchorage-independent growth. Our study demonstrates that FBXW5 drives TNBC progression primarily by restraining CD8^+^ T cell recruitment and activation. Mechanistically, FBXW5 orchestrates the polyubiquitination and autophagic degradation of the cytosolic RNA sensors RIGI and MDA5, thereby blunting type I IFN signaling and the subsequent production of T cell–attracting chemokines. These observations highlight a distinctive functional landscape for FBXW5 that appears to be specific to the TNBC. This divergence may be orchestrated by tissue-specific factors, such as differential substrate accessibility, the relative abundance of competing cofactors and scaffold proteins, or posttranslational modifications that shift FBXW5’s substrate preference. This functional rewiring is consistent with established precedents in E3 ubiquitin ligase biology, such as speckle-type POZ protein and STIP1 homology and U-box containing protein 1, which exert divergent tumor-suppressive or oncogenic effects across diverse malignancies through context-dependent substrate targeting (65–67). Furthermore, the inherent immunogenicity of TNBC might render the suppression of RLR-mediated innate immunity a more critical driver of tumor progression than the modulation of cell-intrinsic growth pathways. While the precise molecular mechanisms underlying this specificity remain to be fully elucidated, our study provides evidence supporting the adaptable nature of E3 ubiquitin ligases in response to distinct TMEs. Collectively, our findings elucidated potentially previously unrecognized regulatory functions of FBXW5 in antitumor immunity, providing compelling evidence that targeting FBXW5 can reshape immune surveillance and reinvigorate the TME.

The tumor cell–intrinsic type I IFN pathway has emerged as a pivotal modulator of cancer progression and therapeutic efficacy (68, 69). This signaling axis exerts a dual influence on the TME: it facilitates the recruitment and expansion of effector T cells by orchestrating the production of chemokines, such as CXCL10, while paradoxically upregulating PD-L1 expression as part of an ISG signature (51). This induction of PD-L1, though immunosuppressive, creates a “primed” state that is highly susceptible to ICB (36, 70). However, in many refractory tumors, the innate pathways required to initiate this priming remain silenced, limiting the efficacy of chemoimmunotherapy. Chemotherapeutic agents such as doxorubicin are well known to break this silence by triggering a “viral mimicry” response (71). This process involves the accumulation of endogenous dsRNA, such as transcripts from endogenous retroelements or mtRNA, which serve as ligands for the cytosolic sensors RIGI and MDA5, thereby triggering the type I IFN axis (72). We identify FBXW5 as a critical “molecular brake” that terminates this response by targeting these sensors for autophagic-lysosomal degradation, effectively preventing the transition to an immunologically hot TME during treatment. Consequently, *Fbxw5* knockdown not only revitalizes CD8^+^ T cell infiltration but also heightens PD-L1 expression, thereby sensitizing tumors to PD-L1 blockade. Furthermore, RLR-induced IFN signaling directly enhances the cytotoxic effects of chemotherapy by lowering the threshold for tumor cell apoptosis (48). Collectively, our data demonstrate that targeting FBXW5 represents a multipronged therapeutic strategy to synergize with chemoimmunotherapy by (a) fostering a CD8^+^ T cell–inflamed microenvironment, (b) potentiating chemotherapy-induced cytotoxic cell death, and (c) optimizing ICB responsiveness through the adaptive upregulation of PD-L1.

The potency of the aforementioned therapeutic strategy depends on FBXW5-mediated precise regulation of innate immune sensing. Nucleic acid sensors, categorized into DNA and RNA sensors, are central to initiating type I IFN–mediated innate immunity (73, 74). Although TLRs primarily monitor the extracellular and endosomal compartments for foreign RNA, RLRs — including RIGI, MDA5, and LGP2 — serve as the predominant intracellular sentinels for cytosolic dsRNA (74). Despite their pivotal roles in immune surveillance, the posttranslational mechanisms governing RLR stability within the immunosuppressive TME have remained largely elusive. In this study, we identify FBXW5 as a selective negative regulator of RLR-mediated cytosolic RNA sensing in TNBC. Our data demonstrate that FBXW5 specifically dictates the stability of RIGI and MDA5 without affecting TLR- or DNA-sensing pathways. Mechanistic studies further demonstrate that FBXW5 directly binds to the CARDs of RIGI and MDA5, facilitating K27-linked polyubiquitination at K193 (RIGI) and K235 (MDA5) to promote their degradation through the autophagic-lysosomal pathway. As a conserved quality control process, selective autophagy employs specialized receptors to recognize ubiquitinated substrates, facilitating their sequestration into LC3-containing autophagosomes for lysosomal degradation (41). In our study, we verified SQSTM1 serves as the autophagy receptor for FBXW5-mediated RIGI/MDA5 degradation, and the UBA domain of SQSTM1 is indispensable for this process. Collectively, our findings uncover key mechanisms of how selective autophagy intersects with type I IFN signaling to orchestrate immunoregulatory networks.

In summary, our study suggests that FBXW5 serves as a tumor-intrinsic negative regulator of RIGI/MDA5-dependent type I IFN signaling, driving immune evasion and progression in TNBC (Figure 8I). Targeting FBXW5 may represent a strategy to reprogram cold TNBC into hot tumors, potentiate response to ICB, and enhance combinatorial chemoimmunotherapy. In addition, FBXW5 expression may serve as an emerging biomarker for immunotherapy responsiveness.

## Methods

### Sex as a biological variable.

Our study exclusively examined female mice because the disease modeled is only relevant in females.

### Animal experiments.

Female immunocompetent BALB/c and immunodeficient mice (5-week-old, specific pathogen–free grade) were obtained from Vital River Laboratory Animal Technology. Animals were inoculated subcutaneously with 5 × 10^5^ 4T1 cells stably expressing *Fbxw5*-overexpression/knockdown plasmids or control vectors. Mice were randomly divided into different groups to receive indicated treatments. For in vivo IFNAR1 blockade study, tumor-bearing mice were injected intraperitoneally (IP) with anti-IFNAR1 IgG1 antibodies (10 mg/kg, Selleck, catalog A2121, RRID: AB_2927720) or rat IgG1 control antibodies (10 mg/kg, Selleck, catalog A2119, RRID: AB_3697767) starting on inoculation day, with subsequent administration 3 times per week. For DOX treatment, mice were treated with DOX (4 mg/kg, MedChemExpress) via IP injection twice a week. For combination treatment, mice were administered DOX or PBS on days 5, 8, 11, and 14 and anti–PD-L1 antibodies (5 mg/kg, RRID: AB_10949073, Bio X Cell) or rat IgG2b isotype control (5 mg/kg, RRID: AB_2687733, Bio X Cell) via IP injection on days 6, 9, 12, and 15. Tumor sizes were measured every 3 days starting on day 6 or day 7. The tumor volumes were calculated using the formula (length × width^2^)/2. Following treatment completion, all mice were humanely euthanized, and tumors were excised for downstream analyses.

### Patients and clinical samples.

Primary TNBC tissues were obtained from patients who underwent surgical resection at Qilu Hospital, Shandong University. None of the patients had received neoadjuvant chemotherapy, radiotherapy, or other systemic therapies before surgery. Exclusion criteria comprised male sex, distant metastatic disease, or concurrent malignancies. All collected tissue specimens were independently confirmed by 3 experienced pathologists. Overall survival was defined as the time interval from the date of surgery to the date of death or the last follow-up.

### Statistics.

Statistical analyses were performed using GraphPad Prism 10 (RRID:SCR_002798). Data normality was assessed via the Shapiro-Wilk test. Comparisons between 2 groups were performed using a 2-tailed Welch’s *t* test or the nonparametric Mann-Whitney *U* test, depending on the normality of the data distribution. For multiple-group comparisons, 1-way or 2-way ANOVA followed by Šídák’s post hoc test, or the Kruskal-Wallis test followed by the 2-stage linear step-up procedure of Benjamini, Krieger, and Yekutieli for FDR control, was applied accordingly. Survival curves were generated using the Kaplan-Meier method and compared via the log-rank test. Patients were stratified into high- and low-expression groups based on the median expression level of *FBXW5*. Quantitative data are presented as the mean ± SD (≥3 independent experiments), with *P* < 0.05 defined as statistically significant.

Detailed methods for bioinformatics analyses and in vitro experiments are described in the Supplemental Methods.

### Study approval.

All procedures performed in studies involving human participants were approved by the Ethics Committee on Scientific Research of Qilu Hospital, Shandong University (Approval No. KYLL-2016-255), and written informed consent was obtained from all participants prior to tissue collection. All in vivo animal studies received approval from the Animal Care and Use Committee of Qilu Hospital, Shandong University (Approval No. DWLL-2024-094), and were conducted according to the institutional guidelines for animal welfare.

### Data availability.

The RNA-seq data generated in this study have been deposited in the GEO database under the accession number GSE326436. The MS proteomics data have been deposited to the ProteomeXchange Consortium via the iProX partner repository with the dataset identifier PXD077632. Public expression profile data analyzed in this study were obtained from GEO (GSE176078) and TCGA databases. Other relevant data are provided in the article, the supplement, or the Supporting Data Values file. Additional information supporting this study are available from the corresponding author upon reasonable request.

## Author contributions

QY, TM, and TC conceived and coordinated the study. XL and TC designed and conducted the majority of experiments, performed data analysis, and visualized the data. WZ, JL, YS, CY, Y Li, CL, XC, XK, and NZ helped in molecular and cellular biology experiments. JL, YS, DH, and SJ assisted with animal studies. Clinical sample collection and data acquisition were performed by WZ, BC, LW, DL, and Y Liang. The manuscript was drafted by TC and XL, with critical review and approval by all authors. The order of the co–first authors was determined by mutual agreement among all authors, recognizing the complementary and essential nature of their respective contributions to the conceptualization and execution of the study.

## Conflict of interest

The authors have declared that no conflict of interest exists.

## Funding support

- National Natural Science Foundation of China (No. 82373005, No. 82573211) to QY.
- Special Foundation for Taishan Scholars (No. tstp20250509) to QY.
- National Natural Science Foundation of China (No. 82303814) to TC.
- Shandong Provincial Natural Science Foundation, China (No. ZR2023QH307) to TC.
- Shandong Provincial Natural Science Foundation, China (No. ZR2025MS1262) to XK.
- National Natural Science Foundation of China (No. 82373267) to NZ.

## Supplementary Material

Supplemental data

Unedited blot and gel images

Supporting data values

## Figures and Tables

**Figure 1:**
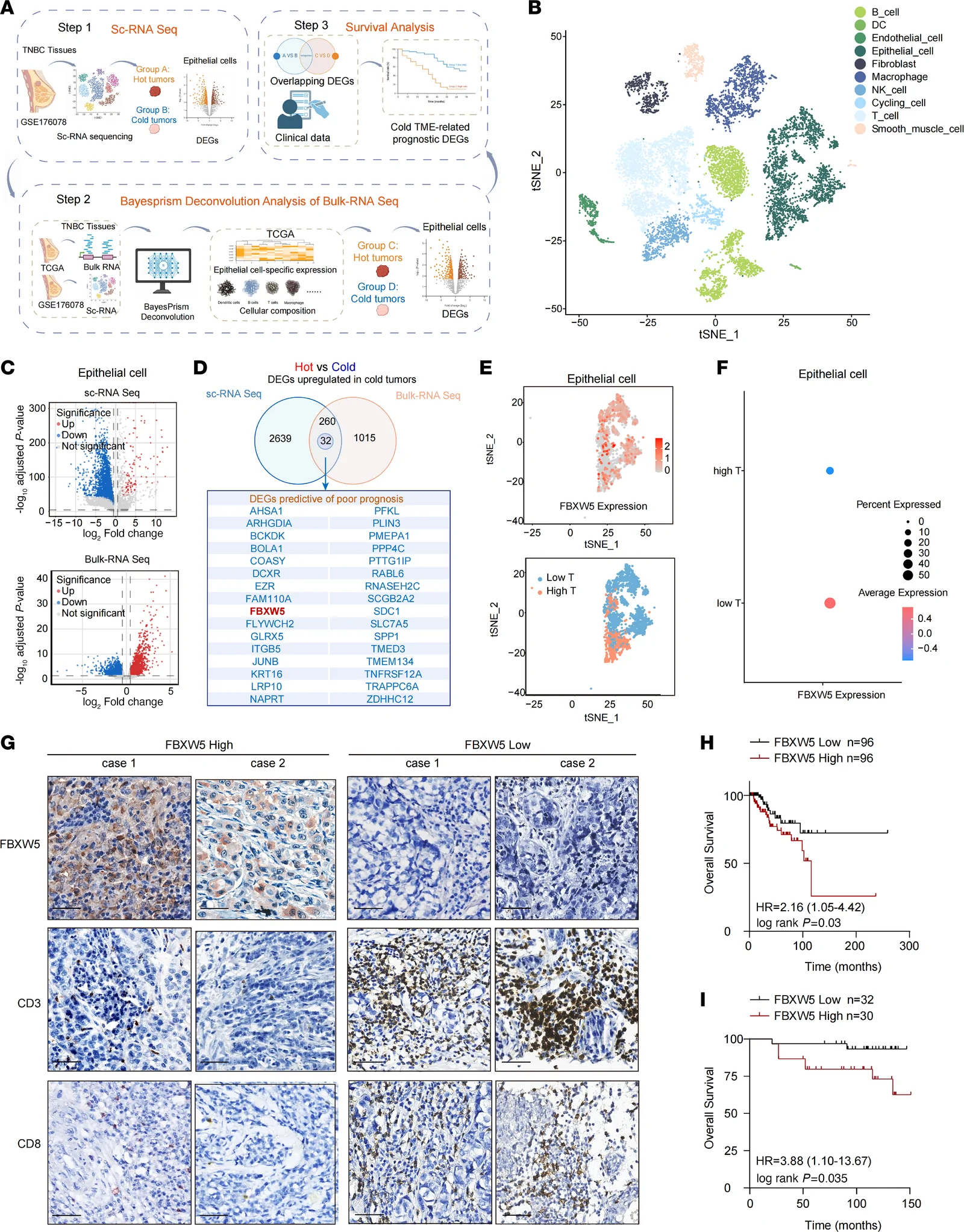
FBXW5 is associated with immunologically cold TME and unfavorable prognosis in TNBC. (**A**) Schematic workflow for identifying cold TME-related genes linked to poor prognosis through integrated analysis of single-cell RNA-seq (scRNA-seq) and bulk RNA-seq data. Some graphical elements were generated using BioRender.com under a publication license. DEGs, differentially expressed genes. (**B**) t-Distributed stochastic neighbor embedding (t-SNE) projection of single-cell transcriptomes, colored and annotated by major cell types. (**C**) Volcano plot displaying tumor-intrinsic DEGs between immunologically hot and cold TNBC samples. (**D**) Venn diagram illustrating the overlap of DEGs derived from scRNA-seq, bulk RNA-seq, and survival analyses, identifying 32 candidate genes at the intersection. (**E** and **F**) Expression of FBXW5 in epithelial cells from immunologically hot versus cold tumors (GSE176078 dataset), visualized by t-SNE projection (**E**) and dot plot (**F**). (**G**) Representative images of IHC staining for FBXW5 and T cell markers (CD3 and CD8) from 3 independent experiments. Scale bars = 50 μm. (**H** and **I**) Kaplan-Meier survival curves comparing outcomes between TNBC patients with epithelial FBXW5-high and FBXW5-low tumors in The Cancer Genome Atlas (TCGA) cohort (**H**) and the Qilu cohort (**I**). *P* values determined by log-rank test (**H** and **I**).

**Figure 2:**
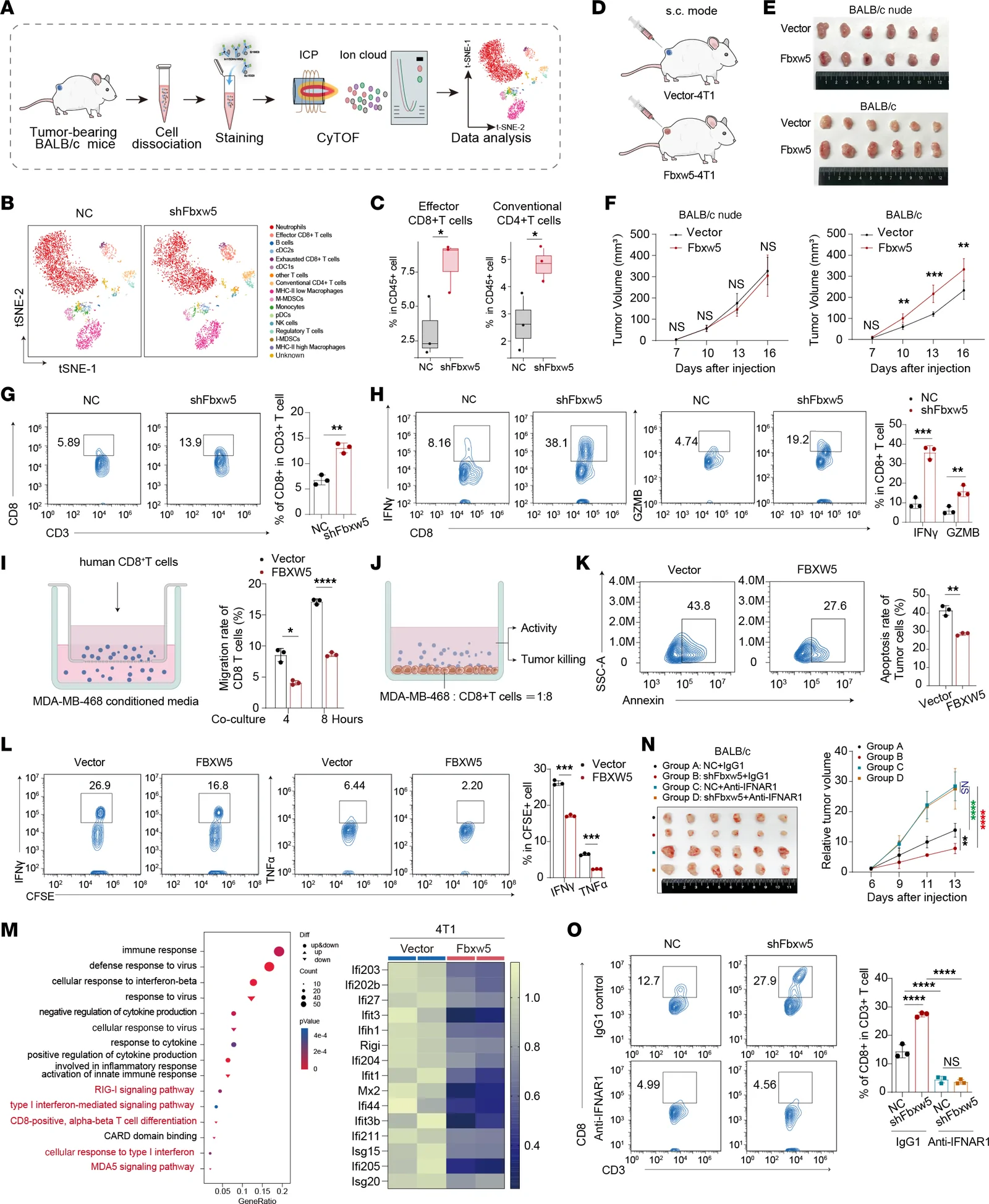
FBXW5 suppresses CD8^+^ T cell infiltration and activation via inhibition of type I IFN signaling in TNBC. (**A**–**C**) CyTOF profiling of control (NC) and murine *Fbxw5*-knockdown tumors: schematic workflow (**A**), t-SNE projections identifying 17 major immune clusters (**B**), and frequency quantification of cell populations (**C**). sh, shRNA. (**D** and **E**) Subcutaneous 4T1 tumor growth in immunocompetent BALB/c versus T cell–deficient nude mice, including schematics (**D**), representative images (**E**), and growth curves (**F**). (**G** and **H**) Flow cytometric quantification of tumor-infiltrating CD8^+^ T cells (**G**) and effector subsets (IFN-γ^+^ or GZMB^+^) in BALB/c mice (**H**). (**I**) Schematic of human CD8^+^ T cells treated with MDA-MB-468–conditioned medium and corresponding migration rates. (**J**–**L**) Schematic of human CD8^+^ T cell and MDA-MB-468 cell direct coculture model (**J**), flow cytometric analysis of apoptosis levels of tumor cells (**K**), and IFN-γ and TNF-α in CFSE-labeled CD8^+^ T cells (**L**) after 48 hours’ coculture. (**M**) GO enrichment of DEGs and heatmap depicting downregulated type I IFN signaling genes (ISGs) in *Fbxw5*-overexpressing versus control 4T1 cells. (**N** and **O**) In vivo rescue assays in BALB/c mice: tumor images and growth curves (**N**), with flow cytometric quantification of total CD8^+^ T cells (**O**). Statistical significance: unpaired 2-tailed Welch’s *t* test (**C** and **G**–**L**); 2-way ANOVA with Šídák’s post hoc test (**F**, **N**, and **O**). Group comparisons: black (**A** vs. **B**), green (**A** vs. **C**), red (**B** vs. **D**) in **N**. *, *P* < 0.05; **, *P* < 0.01; ***, *P* < 0.001; ****, *P* < 0.0001.

**Figure 3:**
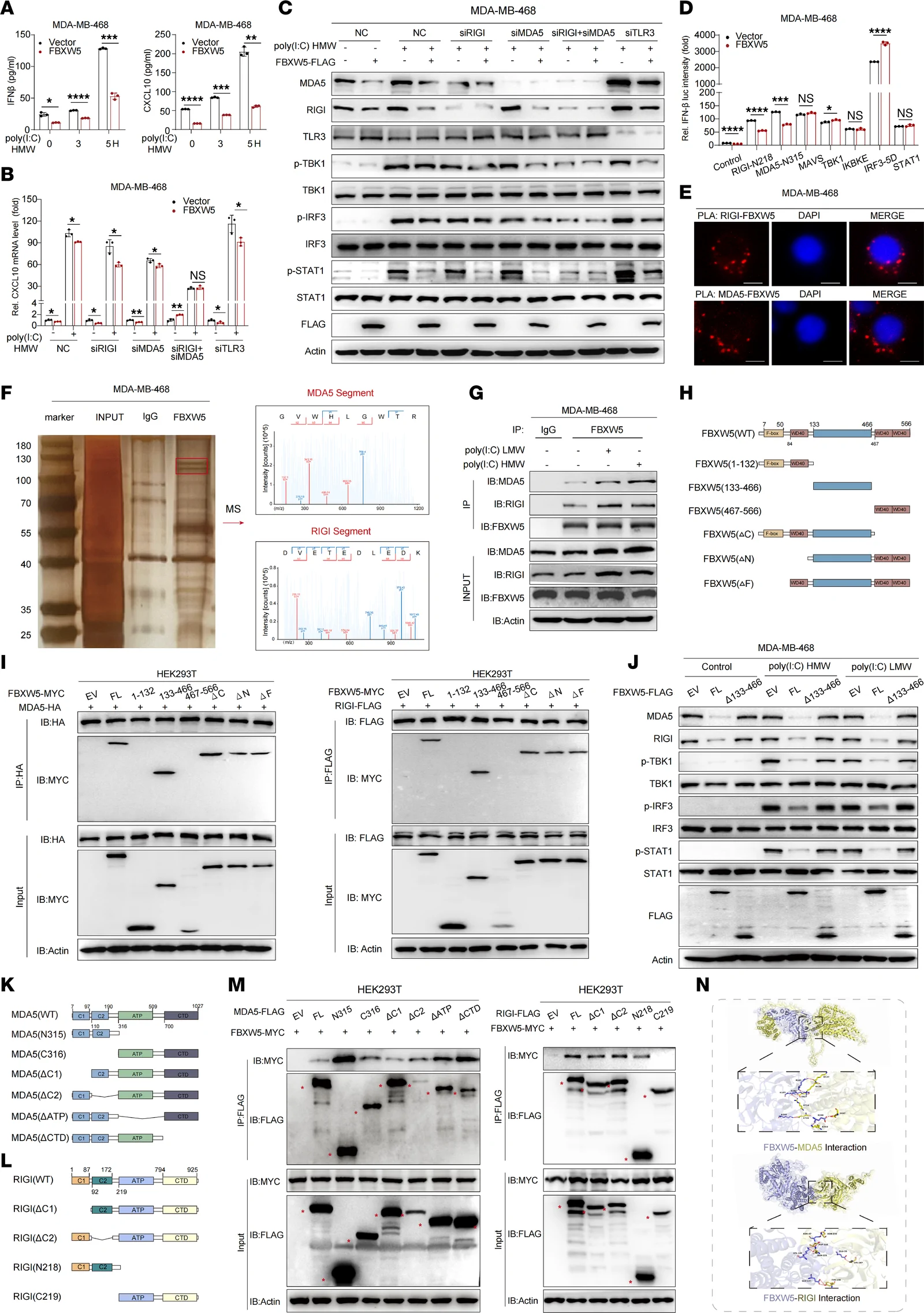
FBXW5 modulates type I IFN signaling via physical interaction with RIGI and MDA5. (**A**) ELISA of IFN-β and CXCL10 in supernatants from MDA-MB-468 cells treated with HMW poly(I:C) (3 μg/mL). (**B**) RT-qPCR of *CXCL10* mRNA in MDA-MB-468 cells with HMW poly(I:C) treatment. si, siRNA. (**C**) Western blot analysis of indicated protein expression upon indicated treatment. TBK1, tank binding kinase 1. (**D**) Luciferase assays of IFNB1 promoter activity in HEK293T cells cotransfected with *FBXW5* and the indicated signaling components. (**E**) PLAs visualizing the spatial proximity of FBXW5 with MDA5 or RIGI in the cytoplasm. The images are representative of 3 independent experiments (scale bars: 10 μm). (**F**) Silver staining and mass spectrometry of FBXW5-specific immunoprecipitates identifying RIGI/MDA5 as interacting partners. (**G**) Co-IP analysis of FBXW5/RIGI/MDA5 interaction in TNBC cells transfected with poly(I:C) HMW (3 μg/mL) or poly(I:C) LMW (10 μg/mL). (**H**) Schematic representation of truncated constructs of FBXW5. ΔC, ΔF, and ΔN refer to the C-terminal, F-box, and N-terminal deletion constructs of FBXW5, respectively. (**I**) Interaction of RIGI/MDA5 with FBXW5 truncations analyzed by co-IP. (**J**) Immunoblotting analysis of indicated protein levels in MDA-MB-468 cells expressing full-length FBXW5 or Δ133-466 mutant upon indicated treatment. (**K** and **L**) Schematic representation of truncated constructs of MDA5 and RIGI. (**M**) Interaction of FBXW5 with RIGI/MDA5 truncations analyzed by co-IP. (**N**) AlphaFold3 modeling of FBXW5/RIGI/MDA5 interactions. Red asterisks denote specific protein bands to distinguish them from nonspecific signals. Statistical significance was determined by unpaired 2-tailed Welch’s *t* test. *, *P* < 0.05; **, *P* < 0.01; ***, *P* < 0.001; ****, *P* < 0.0001.

**Figure 4:**
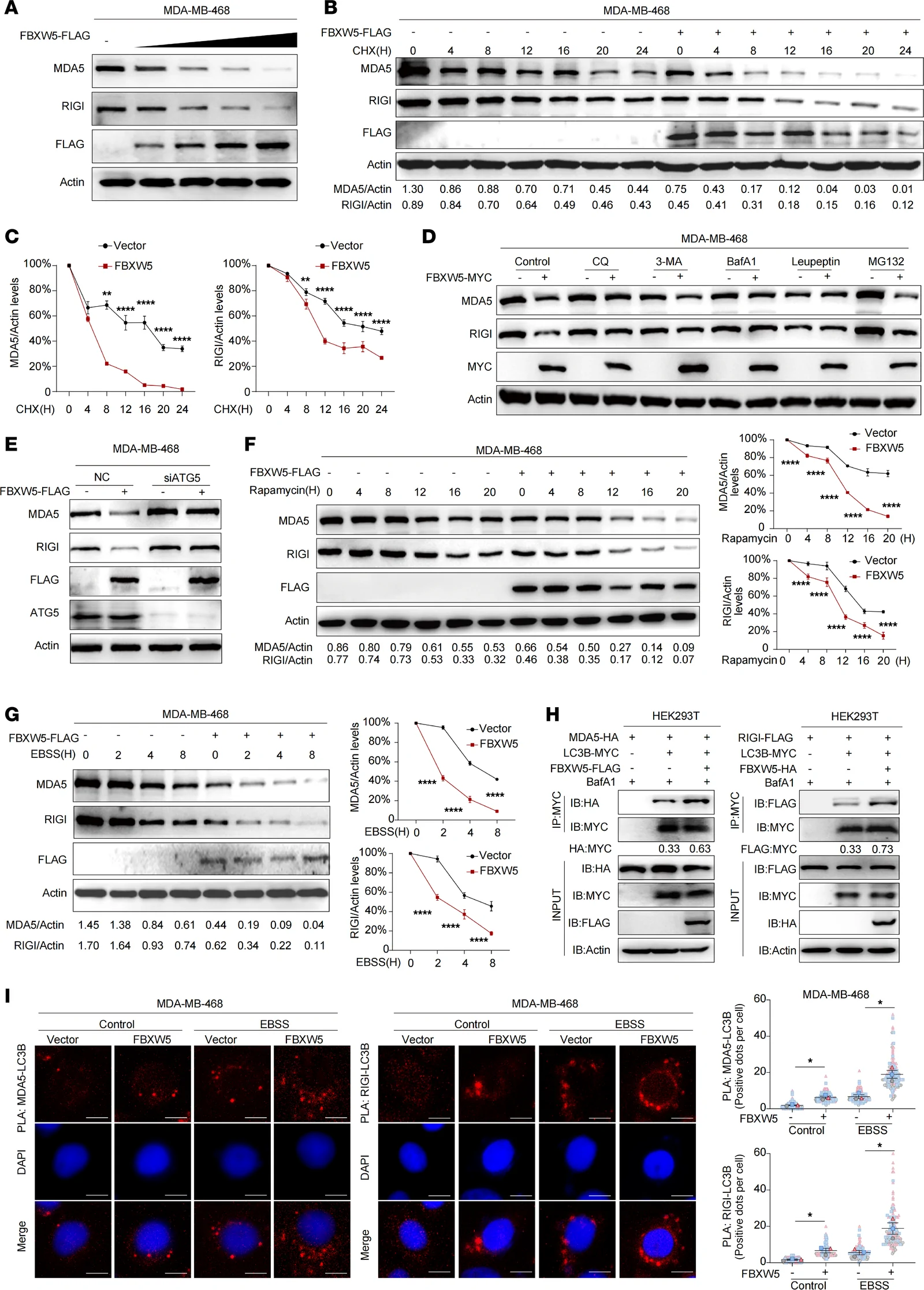
FBXW5 orchestrates autophagic degradation of RIGI/MDA5 proteins. (**A**) Western blot analysis showing RIGI/MDA5 suppression by FBXW5. (**B** and **C**) RIGI/MDA5 turnover in MDA-MB-468 cells treated with cycloheximide (CHX, 50 μg/mL). Quantification normalized to Actin (ImageJ, NIH). (**D**) Immunoblot of RIGI and MDA5 in cells treated with autophagy inhibitors: chloroquine (CQ; 100 μM), 3-MA (5 mM), bafilomycin A1 (BafA1, 100 nM), leupeptin (50 μM), or proteasome inhibitor MG132 (10 μM) for 8 hours. (**E**) Immunoblot of RIGI/MDA5 in cells transfected with control or *ATG5* siRNAs + empty vector (EV) or *FBXW5*-FLAG overexpression plasmids. (**F** and **G**) Western blot in MDA-MB-468 cells transfected with EV/*FBXW5*-FLAG, followed by treatment with rapamycin (200 nM) (**F**) or Earle’s balanced salt solution (EBSS) (**G**) for the indicated time points. Quantification normalized to Actin (ImageJ). (**H**) Co-IP analysis of interactions between *LC3B*-MYC with *MDA5*-HA or *RIGI*-FLAG in HEK293T cells. (**I**) PLAs visualizing the spatial colocalization of LC3B with MDA5 or RIGI in TNBC cells subjected to the indicated treatments. Images are representative of 3 independent experiments. Scale bars: 10 μm. Statistical significance was determined by paired (for PLA) 2-tailed Welch’s *t* tests, or 2-way ANOVA with Šídák’s multiple-comparison test (for Western blot analysis). *, *P* < 0.05; **, *P* < 0.01; ****, *P* < 0.0001.

**Figure 5:**
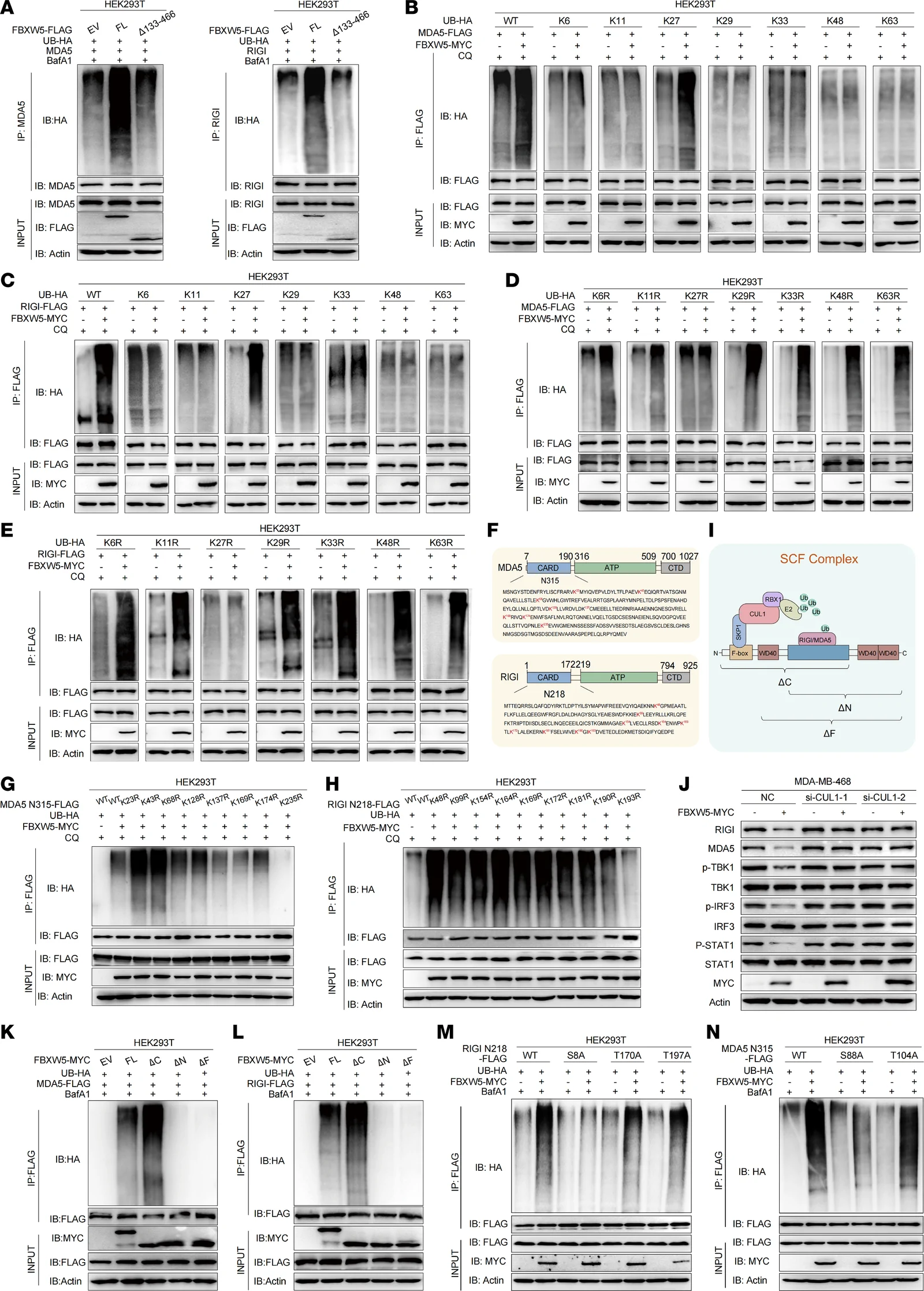
FBXW5 promotes K27-linked ubiquitination of RLRs. (**A**) Polyubiquitination levels of MDA5/RIGI in HEK293T cells expressing *FBXW5*-full length (FL) or Δ133-466 mutant, with bafilomycin A1 (BafA1) (600 nM, 8 hours) treatment. (**B** and **C**) Analysis of the ubiquitin linkage types on MDA5 (**B**) and RIGI (**C**) in HEK293T cells expressing HA-tagged WT ubiquitin or lysine-only mutants (K6, K11, K27, K29, K33, K48, or K63) after CQ treatment (100 μM, 8 hours). (**D** and **E**) Identification of the specific linkage required for FBXW5-mediated ubiquitination using a panel of linkage-restricted ubiquitin mutants (single Lys-to-Arg substitutions) under CQ treatment in MDA5 (**D**) and RIGI (**E**). (**F**) Schematic representation of key ubiquitination sites in MDA5 or RIGI N-terminal CARD-containing domains. (**G** and **H**) Ubiquitination assays in HEK293T cells expressing WT or mutated N-terminal CARD-containing truncations of MDA5 (**G**) or RIGI (**H**), with CQ treatment. (**I**) Schematic representation of the structure of SCF complex. (**J**) Western blot analysis of indicated protein levels in MDA-MB-468 cells cotransfected with NC or *CUL1*-targeting siRNAs and EV or FBXW5-MYC. (**K** and **L**) Polyubiquitination levels of MDA5/RIGI in HEK293T cells expressing FBXW5-FL or indicated mutant, with BafA1 treatment. (**M** and **N**) Ubiquitination assays in HEK293T cells expressing WT or phospho-dead (S-to-A) mutants of the N-terminal CARD-containing truncations of RIGI (**M**) or MDA5 (**N**) after BafA1 treatment.

**Figure 6:**
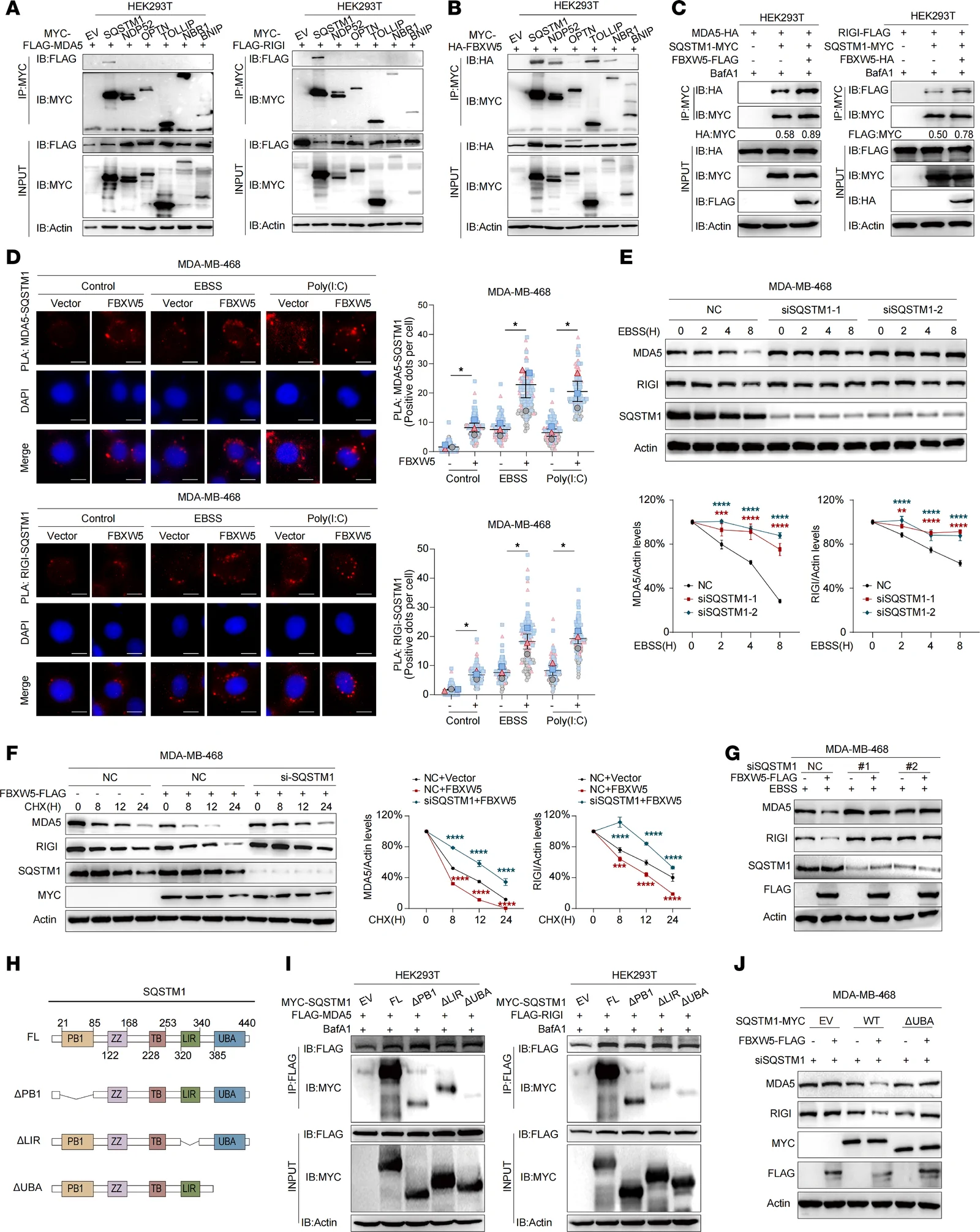
FBXW5 bridges RLRs to SQSTM1 for autophagic degradation. (**A** and **B**) Co-IP analysis of MYC-tagged cargo receptors and MDA5- or RIGI-FLAG (**A**) or FBXW5-HA (**B**) in HEK293T cells. (**C**) Co-IP analysis of interactions between SQSTM1-MYC and MDA5-FLAG/RIGI-FLAG in HEK293T cells upon indicated treatment. (**D**) PLAs visualizing the spatial colocalization of SQSTM1 with MDA5 or RIGI in TNBC cells subjected to PBS, EBSS, or poly(I:C) treatments. Images are representative of 3 independent experiments. Scale bars: 10 μm. (**E**) Western blot analysis of indicated protein levels in MDA-MB-468 cells transfected with NC/*SQSTM1*-targeting siRNAs (si*SQSTM1*). Quantification normalized to Actin (ImageJ). (**F** and **G**) Western blot analysis of indicated protein levels in MDA-MB-468 cells cotransfected with NC/si*SQSTM1* and EV/*FBXW5*-FLAG plasmids, with CHX (50 μg/mL) (**F**) or EBSS (**G**) treatment. Quantification normalized to Actin (ImageJ). (**H**) Schematic representation of truncated constructs of SQSTM1 proteins. (**I**) Co-IP analysis of interactions between MDA5/RIGI and SQSTM1 truncations. (**J**) Western blot analysis of indicated protein levels in MDA-MB-468 cells transfected with NC/si*SQSTM1*, followed by cotransfection with *FBXW5*-FLAG and full-length *SQSTM1* (WT) or UBA domain–deleted *SQSTM1* (ΔUBA). Statistical significance: paired 2-tailed Welch’s *t* test (PLA); 2-way ANOVA with Šídák’s multiple-comparison test (Western blot). *, *P* < 0.05; ***, *P* < 0.001; ****, *P* < 0.0001.

**Figure 7:**
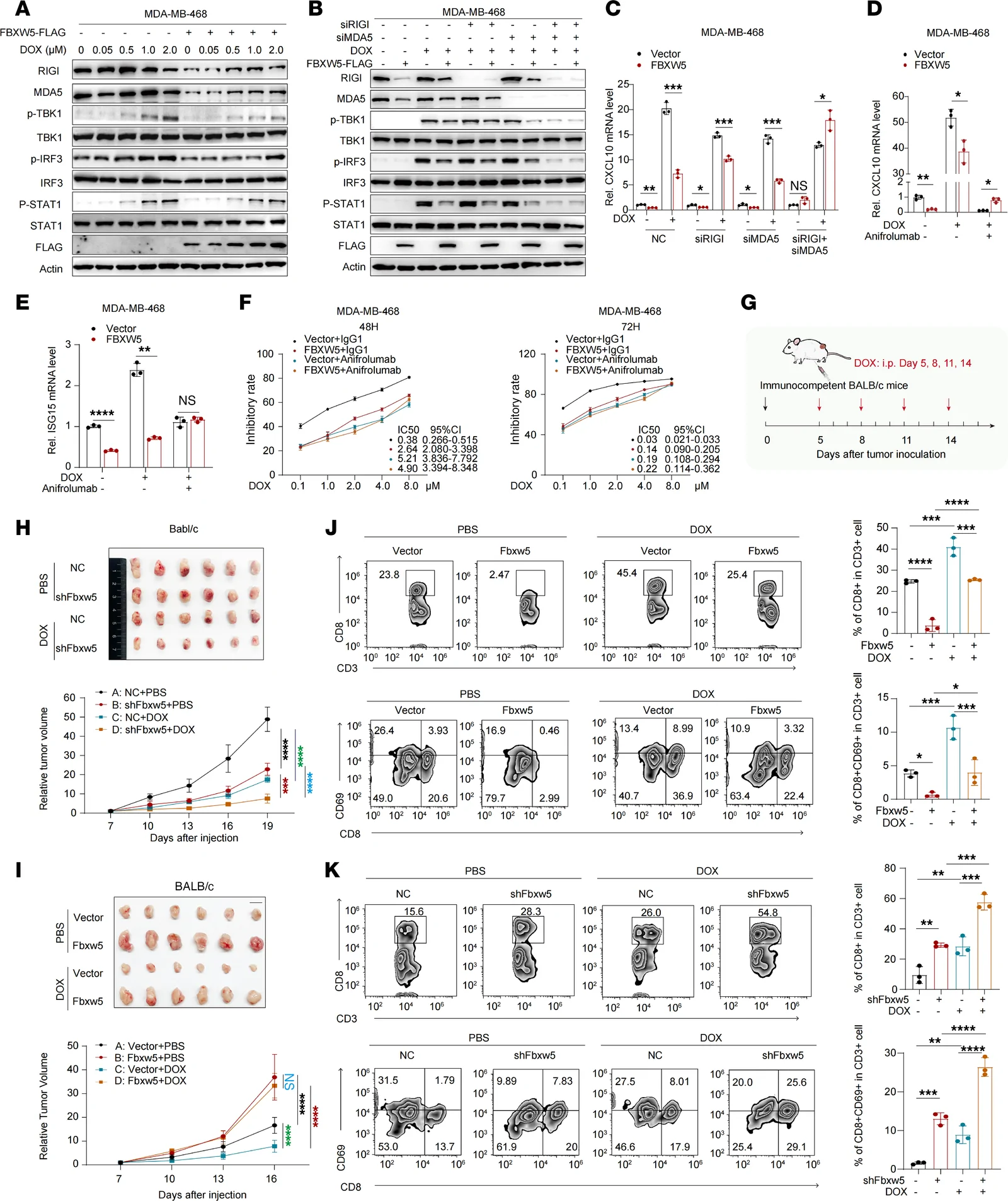
FBXW5 inhibits chemotherapy efficacy by blunting type I IFN signaling. (**A**) Immunoblot analysis of proteins in EV/*FBXW5*-overexpressing MDA-MB-468 cells treated with DOX for 24 hours. (**B** and **C**) Western blot (**B**) and RT-qPCR (**C**) analysis of indicated gene levels in MDA-MB-468 cells cotransfected with *RIGI*/*MDA5*-targeting siRNAs and EV/*FBXW5*-overexpressing plasmids, followed by DOX treatment (2 μM, 24 hours). (**D** and **E**) RT-qPCR of *CXCL10* (**D**) and *ISG15* (**E**) levels in *FBXW5*-overexpressing cells treated with anifrolumab and DOX. (**F**) Dose-response curves showing reduced chemosensitivity (increased IC_50_) in *FBXW5*-overexpressing TNBC cells. IC_50_ values with 95% confidence intervals are indicated. (**G**) Schematic of animal experiments. (**H** and **I**) Tumor growth curves and photographs of subcutaneous tumors across treatment groups (group A: as control). Scale bars = 10 mm. (**J** and **K**) Flow cytometry analysis of tumor-infiltrating CD8^+^ T cell percentages. Statistical significance: unpaired Welch’s *t* test for RT-qPCR; 2-way ANOVA with Šídák’s post hoc test for data in **H**–**K**; asterisks denote comparisons, and black (**A** vs. **B**), green (**A** vs. **C**), red (**C** vs. **D**), blue (**B** and **D**) in **H** and **I**. *, *P* < 0.05; **, *P* < 0.01; ***, *P* < 0.001; ****, *P* < 0.0001.

**Figure 8:**
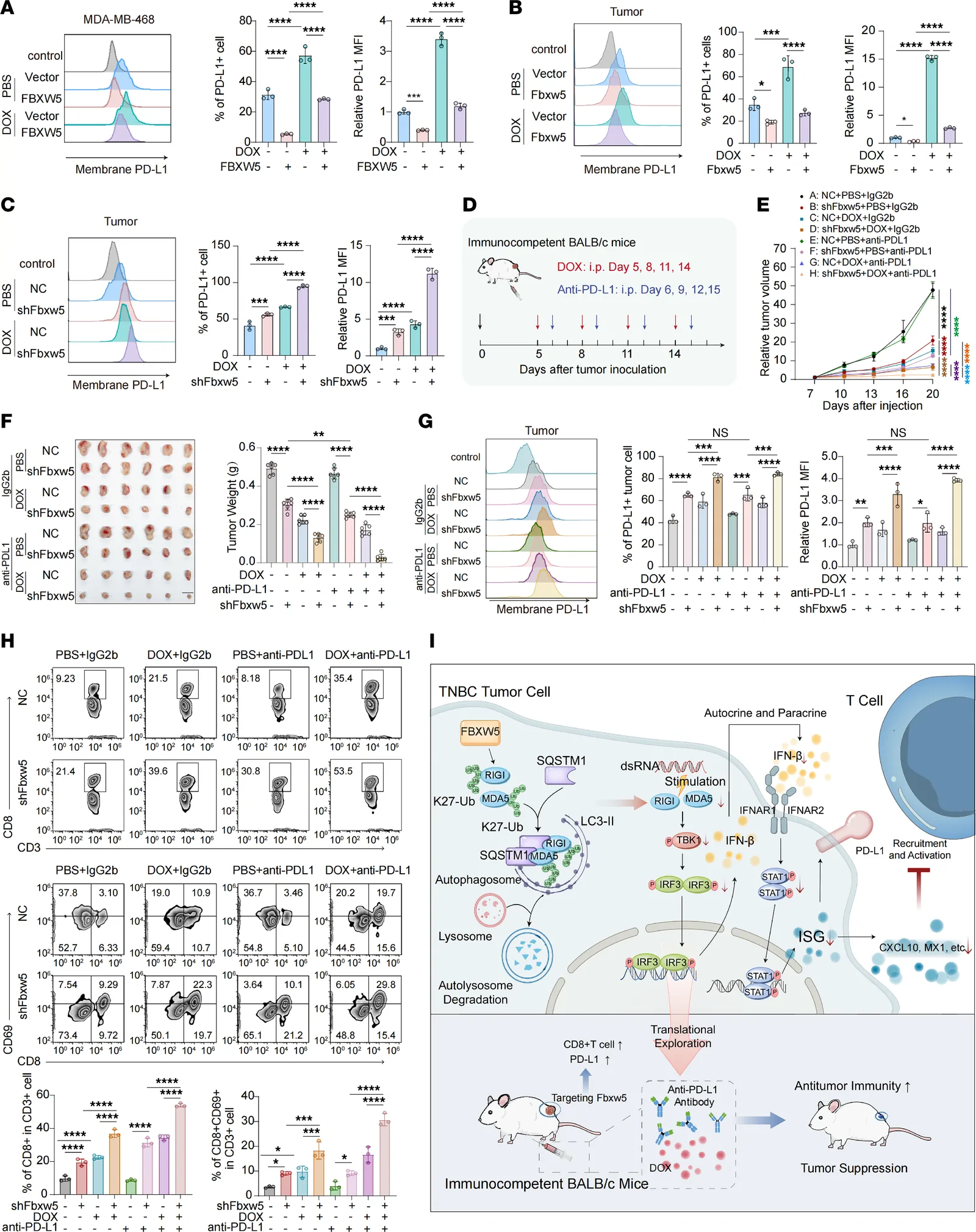
*FBXW5* knockdown improves the efficacy of ICB and chemoimmunotherapy. (**A**–**C**) Flow cytometry analysis of PD-L1 surface expression in MDA-MB-468 cells (**A**) or mouse subcutaneous tumors (**B** and **C**) upon indicated treatment. (**D**) The schematic diagram showing the process of animal experiments. (**E**) Growth curves of relative volumes of subcutaneous tumors in different groups with group A as control. (**F**) Photographs and weight of subcutaneous tumors in different groups. (**G**) Flow cytometry analysis of PD-L1 surface expression in mouse subcutaneous tumors from different groups. (**H**) Flow cytometric analysis of percentages of tumor-infiltrating CD8^+^ T cells from subcutaneous tumors. (**I**) Mechanistic diagram illustrating FBXW5-mediated regulation of RLRs’ autophagic degradation and subsequent type I IFN signaling attenuation, resulting in an immune-evasive tumor microenvironment and ICB resistance in TNBC. Statistical significance: 2-way ANOVA with Šídák’s post hoc test; asterisks denote comparisons, and black (**A** vs. **B**), green (**E** vs. **F**), purple (**G** vs. **H**), red (**B** vs. **F**), yellow (**F** vs. **H**), blue (**D** vs. **H**), and orange (**B** vs. **D**) in **E**. *, *P* < 0.05; **, *P* < 0.01; ***, *P* < 0.001; ****, *P* < 0.0001.

## Version 1 08/25/2026

In-Press Preview

## Version 2 10/15/2026

Electronic publication
