# Supplementary material for: FBXW5 blunts RIGI/MDA5/IFN signaling to drive immune evasion and chemo-immune resistance in triple-negative breast cancer models
Source: J Clin Invest. 2026 Aug 25;136(20):e201573. doi: 10.1172/JCI201573 (PMC13668642; doi:10.1172/JCI201573)
Supplement: Supplementary file 1 [file jci-136-201573-s087.pdf]

SUPPLEMENTARY METHODS

Cell Culture and Transfection

Human TNBC cell lines [HS578T (RRID: CVCL_0332), MDA-MB-468 (RRID: CVCL_0419), BT549 (RRID: CVCL_1092), MDA-MB-453 (RRID: CVCL_ 0418), MDA-MB-231 (RRID: CVCL_0062), MDA-MB-436 (RRID: CVCL_0623), HCC1937 (RRID: CVCL_0290)], murine TNBC cell line [4T1 (RRID: CVCL_0125)], and HEK-293T (RRID: CVCL_0063) cells were purchased from American Type Culture Collection (ATCC, VA, USA) and cultured in High-glucose DMEM (Macgene, Beijing, China) supplemented with 10% fetal bovine serum (Gemini, CA, USA), 100 U/ml penicillin (Macgene, Beijing, China) and 100 μg/ml streptomycin (Macgene, Beijing, China). All cell cultures were maintained at 37°C under humidified conditions containing 5% CO_2.

Cells were transfected with indicated overexpression plasmids, short-hairpin RNA (shRNA) plasmids or siRNAs using Lipofectamine 2000 (Invitrogen, CA, USA) according to the instructions of manufacturers. The target sequences of shRNAs and siRNAs designed for this study are detailed in **Supplemental Table 2**. Truncation variants of FBXW5, RIGI, MDA5, and SQSTM1, as well as lysine-substitution mutants within the CARD domains of RIGI and MDA5, were generated using the KOD-Plus Mutagenesis Kit (Toyobo, Japan). The primers used for mutagenesis are listed in **Supplemental Table 2**.

Cytometry by Time-of-Flight (CyTOF) Analysis

Single-cell suspensions were prepared from murine TNBC tumors via enzymatic dissociation. Cell viability was confirmed to be > 90% by trypan blue exclusion, and the resulting suspensions were filtered through a 40 μm cell strainer to eliminate aggregates. For each sample, $3\times10^{\wedge 6}$ cells were incubated with 5 μM Cisplatin-195Pt (Standard BioTools, CA, USA) for 5 minutes at room temperature to label non-viable cells, followed by quenching and washing with FACS buffer (PBS containing 0.5% BSA). After blocking Fc receptors with anti-CD16/32 (BioLegend, CA, USA) for 15 minutes at room temperature, cells were stained with metal-conjugated surface antibody cocktail (**Supplemental Table 3**) at 4 ℃ for 30 minutes. Following fixation, samples were incubated with DNA intercalator overnight at 4 ℃. For intracellular marker detection, samples were processed with eBioscience™ Intracellular Fixation & Permeabilization Buffer (Invitrogen, CA, USA), followed by further incubation with a metal-labeled monoclonal antibody cocktail targeting intracellular proteins. Data were acquired on a CyTOF system (Standard BioTools, CA, USA) and normalized using 10% EQ Four Element Calibration Beads (Standard BioTools, CA, USA). FCS files were pre-processed in FlowJo (v10.8.1) to isolate live, single, CD45+ immune cells populations. Downstream analysis was performed using the *cytofWorkflow* R package.

CD8+ T cell isolation and in vitro assay

Human peripheral blood mononuclear cells (PBMCs) were isolated using Human Lymphocyte Separation Medium (Dakewe Biotech Co., Ltd., Shenzhen, China). Subsequently, CD8+ T cells were purified from PBMCs using Human CD8+ T cell Isolation Kit (Miltenyi Biotec, BG, Germany), labeling with CFSE (MedChemExpress,

NJ, USA) followed by culture for 2–3 days in RPMI 1640 medium supplemented with 10 ng/mL IL-2 (PeproTech, NJ, USA) and 25 μL/mL ImmunoCult™ Human CD3/CD28 T Cell Activator (Stemcell Technologies, Vancouver, Canada).

For the CD8+ T cell migration assay, activated CD8+ T cells adjusted to a density of 5 × 10^6 cells/mL. A 100 μL aliquot of the CD8+ T cell suspension was then placed into the upper chamber of Transwell insert with a 5.0 μm pore size. Cells were allowed to migrate into the lower chamber, which contained supernatant culture medium from MDA-MB-468 cells under different treatment conditions. Following a migration period of 4 or 8 hours, media from the lower chamber were collected, and centrifuged at 500g for 5 minutes. Cells were suspended and counted using a cell counting analyzer (Ruiyu Biotech Co., Ltd, Shanghai, China). The chemotactic rate was calculated as the percentage of migrated cells in the lower chamber relative to the total cells seeded.

For the T-cell mediated tumor killing assay, CFSE-labeled CD8+ T cells were co-cultured with MDA-MB-468 cells at an 8:1 ratio in RPMI 1640 medium. After 24 hours, apoptosis of tumor cells was assessed using the PE Annexin V Apoptosis Detection Kit (BD Pharmingen, NJ, USA). Briefly, both suspended and adherent cells were collected, washed twice with cold PBS, and resuspended in 100 μL of 1× binding buffer. Cells were then stained with PE-conjugated Annexin V and incubated for 15 minutes at room temperature in the dark. Samples were analyzed on a DxFLEX flow cytometer (Beckman Coulter, CA, USA). Apoptotic tumor cells were defined as Annexin V^+/$CFSE^-$ populations.

To assess CD8+ T cell activity, supernatant cells were harvested from cocultures of MDA-MB-468 cells with CFSE-labeled CD8+ T cells, washed twice with cold PBS, and subjected to sequential staining. Surface staining was conducted initially using a PC5.5-conjugated anti-human CD8 antibody (BD Pharmingen, NJ, USA) for 30 min at 4°C in the dark. After washing with PBS, cells were fixed and permeabilized with the eBioscience™ Intracellular Fixation & Permeabilization Buffer (Invitrogen, CA, USA), followed by intracellular staining with fluorochrome-conjugated anti-IFN-γ and anti-TNF-α antibodies for 30 min at 25°C protected from light. Following final washes, samples were analyzed on a DxFLEX flow cytometer (Beckman Coulter, CA, USA).

Cell Viability and Chemosensitivity Assays

Cell viability and proliferation were assessed using the 3-(4,5-dimethyl-2-thiazolyl)-2,5–diphenyl-2H-tetrazolium bromide (MTT) assay, Cell Counting Kit-8 (CCK8), and soft Agar Colony Formation Assay.

For MTT assay, MDA-MB-468 and 4T1 cells were seeded in 96-well plates at 2,000 and 100 cells/well, respectively. For chemosensitivity evaluations, cells were plated at 4,000 cells/well and treated with varying concentrations of doxorubicin (DOX) or DMSO as a vehicle control. Following the indicated treatments, 20 μL MTT solution (5 mg/mL) was added to each well. After 4-hour incubation at 37°C, supernatants were carefully removed, and intracellular formazan crystals were solubilized with 100 μL DMSO. Absorbance at 490 nm was measured using a Spark multimode microplate reader (TECAN, Männedorf, Switzerland). The growth curves and half-maximal

inhibitory concentrations (IC50) were calculated using GraphPad Prism v10 (GraphPad Software).

For CCK8 assay, MDA-MB-468, MDA-MB-231 and 4T1 cells were seeded in 96-well plates (2,000, 800 and 50 cells/well, respectively) and cultured for the indicated time points. At each interval, 10 μL of CCK-8 reagent was added to each well containing 100 μL of culture medium, followed by incubation at 37°C for 4 hours. Absorbance at 450 nm was recorded. Growth curves were generated by normalizing absorbance values to the initial cell density at Day 0.

Anchorage-independent growth was assessed using a two-layer agarose system. The base layer consisted of 3% low-melting-point agarose mixed with 2× complete medium. MDA-MB-231 and 4T1 cells were dissociated into a single-cell suspension and resuspended in a 0.3% top agarose layer, then were seeded in 6 cm plates at densities of 200 and 400 cells/well, respectively. To ensure cell viability, the agarose temperature was strictly maintained between 37°C and 40°C during the plating process. After 2–3 weeks of incubation, colonies were imaged using a Nexcope microscope (Ningbo, Zhejiang, China) and colonies exceeding 50 μM in diameter were quantified.

Immunohistochemistry

Immunohistochemical staining was performed on tumor tissue specimens using a Universal SP Kit (ZSGB-BIO, Beijing, China). Briefly, Formalin-fixed, paraffin-embedded (FFPE) tumor specimens were sectioned at 4 μm thickness. After deparaffinization in xylene and rehydration through graded ethanol, antigen retrieval

was performed in citrate buffer (pH 6.0) or EDTA buffer (pH 8.0). Endogenous peroxidase activity was quenched with 3% hydrogen peroxide (10 min, RT), followed by protein blocking with 5% BSA (30 min, RT). Sections were incubated with primary antibodies (**Supplementary Table 3**) overnight at 4°C in a humid chamber. The next day, sections were incubated with HRP-conjugated secondary antibodies (30 min, RT), followed by DAB chromogen (ZSGB-BIO, Beijing, China) treatment, and counterstained with Mayer's hematoxylin. After dehydration and mounting, whole-slide scanning was performed using a digital slide scanner (Winmedic Tech Co., Jinan, China). Staining intensity was semi-quantitatively scored by two blinded pathologists using a validated 0–3 scale (negative to strong) as previously reported.

RNA Isolation and Quantitative Real-Time PCR (qPCR) Analysis

Total RNA was extracted from cells using TRIzol™ reagent (Invitrogen, CA, USA). Complementary DNA (cDNA) was synthesized from total RNA using the Evo M-MLV Reverse Transcription Kit (Accurate Biology, Hunan, China). qPCR was performed using SYBR® Green Premix Pro Taq HS qPCR Kit (Accurate Biology, Hunan, China) on a LightCycler® 480 II Real-Time PCR System (Roche, Switzerland) to determine indicated RNA levels. Beta-actin served as an endogenous control. Primer sequences used in our study are provided in **Supplementary Table 2**.

Western Blot and Immunoprecipitation

For protein extraction, cells were lysed in Cell lysis buffer for Western and IP (Beyotime Biotechnology, Shanghai, China) supplemented with protease/phosphatase inhibitors.

Protein concentrations were determined by BCA assay prior to separation by SDS-PAGE gel electrophoresis. For Western Blot, proteins were transferred to PVDF membranes (Millipore) and blocked with 5% non-fat milk. Membranes were probed with primary antibodies (4°C overnight) and HRP-conjugated secondary antibodies (room temperature, 2 h), followed by signal detection with the ECL detection Reagent (Millipore, MA, USA). For silver staining, gels were fixed and stained using the Fast Silver Stain Kit (Beyotime Biotechnology, Shanghai, China) according to the manufacturer's protocol. For immunoprecipitation studies, equal amounts of protein lysates were incubated with target-specific antibodies overnight at 4°C. Immune complexes were captured using Protein A/G Magnetic Beads (MedChemExpress, NJ, USA) at 4°C. Then beads were washed for five times with washing buffer and eluted for downstream analysis.

Reagents and Antibodies

Reagents used in this study were as follows: poly(I:C) HMW, poly(I:C) LMW were purchased from Invivogen (CA, USA); HtDNA was purchased from Sigma-Aldrich (MO, USA); Chloroquine, MG132, Leupeptin HemisuLfate, Cycloheximide (CHX) were obtained from Selleck (TX, USA); Bafilomycin A1, 3-Methyladenine (3-MA), Rapamycin, DOX were obtained from MedChemExpress (NJ, USA); EBSS was obtained from Solarbio (Beijing, China).

Antibodies used in this study were as follows: anti-HA, anti-p-TBK1, anti-p-IRF3, anti-STAT1 were obtained from Abcam (Cambridge, UK); anti-MDA5, anti-TBK1, anti-

IRF3 were from Abclonal (Wuhan, China); anti-MYC were from Bethyl Laboratories (TX, USA); anti-mouse IgG, anti-rabbit IgG, anti-RIGI, anti-p-STAT1 were from Cell Signaling Technology (MA, USA); anti-TLR3, anti-SQSTM1, anti-LC3B, anti-FBXW5, anti-Actin were from Proteintech (CHI, USA); anti-Flag were from Invitrogen (CA, USA).

Detailed information of reagents and antibodies (e.g., catalog numbers, RRIDs) are provided in **Supplementary Table 3**.

Flow Cytometry

DOX-induced apoptosis in TNBC cells was detected using the FITC Annexin V Apoptosis Detection Kit (BD Pharmingen, NJ, USA) according to the manufacturer's protocol. Briefly, cells were harvested upon indicated treatment, washed in cold PBS, and stained with FITC-Annexin V for 15 min at room temperature (RT) in the dark. For cell surface CD274 (PD-L1) detection, cells were collected after indicated treatment, and stained with APC-conjugated anti-human CD274 antibody or isotype control for 30 min at 4°C. For DOX accumulation and efflux assays, MDA-MB-468 cells were seeded in 12-well plates (3×10^5 cells/well). In the accumulation assay, cells were incubated with 5 μg/ml DOX for 6 hours. In the efflux assay, cells were pre-loaded with 5 μg/ml DOX for 2 hours, washed with 37°C PBS, and then incubated in DOX-free medium—with or without reversal agents—for indicated durations (0–3 hours). Intracellular DOX levels were quantified by measuring mean fluorescence intensity (MFI). For analysis of tumor-infiltrating CD8+ T cells, subcutaneous tumors were

minced on ice and digested in RPMI 1640 containing 2 mg/mL collagenase IV (Solarbio, Beijing, China) and 0.2 mg/mL DNase I (Solarbio, Beijing, China) at 37°C for 45 min. Single-cell suspensions were obtained through passing 70 µm strainers, then lysed for RBC removal. After washing, cells were resuspended in PBS and stained for surface markers with antibodies against mouse CD45, CD3, CD8, and CD69 for 40 minutes at 4°C in the dark. For detection of intracellular cytokines (IFN-γ, Granzyme B), cells were incubated in culture medium containing Leukocyte Activation Cocktail (BD Pharmingen, NJ, USA) for 4 hours. Following surface staining, cells were washed, fixed, and permeabilized with the eBioscience™ Intracellular Fixation & Permeabilization Buffer (Invitrogen, CA, USA) according to manufacturer's instructions. Intracellular staining was performed with anti-mouse IFN-γ and GZMB antibodies for 40 min at 4 °C. Above samples were then washed with cold PBS and were performed using on the DxFLEX Flow Cytometer (BeckmanCoulter, CA, USA).

Proximity Ligation Assay (PLA)

PLA was performed using the NaveniFlex Cell MR kit (Navinci, Sweden, Uppsala). Cells were fixed, permeabilized, and incubated with Block solution for 60 minutes at 37°C. Samples were then incubated with primary antibodies, followed by Navenibody M1 and R2 (1:40 dilution) for 60 minutes at 37°C. After washing with pre-warmed 1× TBS-T, Reaction 1 (Buffer 1 and Enzyme 1) was performed for 30 minutes, followed by Reaction 2 (Buffer 2 and Enzyme 2) for 90 minutes at 37°C. Nuclei were counterstained with DAPI before the slides were mounted with an antifade medium. Proximity signals were visualized via confocal microscopy using appropriate filter sets

(Cy3.5 or Cy5). Fluorescence microscopy (ZEISS, Oberkochen, Germany) was employed for image acquisition.

Immunofluorescence

For immunofluorescence staining of tumor cells, MDA-MB-468 were plated on glass slides in 24-well plates and transfected with the indicated plasmids for 24 hours. Following treatments, cells were washed three times with PBS, fixed with 4% paraformaldehyde (15 min, RT), permeabilized with 0.3% Triton X-100 (25 min, RT), and blocked with 10% goat serum (1 h, RT). The slides were incubated with indicated primary antibodies overnight at 4°C, followed by incubation with secondary antibodies (1 h, RT) and DAPI staining (5 min, RT). Following three washing steps, DAPI staining was applied to visualize nuclei. Fluorescence microscopy (ZEISS, Oberkochen, Germany) was employed for image acquisition.

For immunofluorescence staining of formalin-fixed paraffin-embedded (FFPE) mouse tumor tissues, sections were deparaffinized in xylene and rehydrated through a graded ethanol series. Antigen retrieval was subsequently performed using citrate buffer (pH 6.0) or EDTA buffer (pH 8.0) under high-temperature conditions (95°C, 20 min). Endogenous peroxidase activity was blocked with 3% hydrogen peroxide (10 min, RT), followed by incubation with 10% goat serum in PBS (1 h, RT) to block nonspecific binding. Sections were then incubated with primary antibodies (1:100 dilution) at 4°C overnight. After thorough washing with PBS (three times, 5 min), samples were incubated with fluorophore-conjugated secondary antibodies (1:100 dilution, 1 h, RT)

and counterstained with DAPI (10 min, RT) for nuclear visualization. Following washing with PBS (three times, 5 min), fluorescence images were acquired using a Fluorescence microscopy (ZEISS, Oberkochen, Germany).

Enzyme-Linked Immunosorbent Assay (ELISA) Analysis

Quantification of CXCL10 and IFNB in TNBC conditioned media was performed using validated commercial ELISA systems. Briefly, CXCL10 concentrations were determined with the Human IP10 ELISA Kit (DaKeWei Biotech, Shenzhen, China) and IFNB levels were assessed using the ELISA MAX™ Deluxe Human IFN-beta Kit (BioLegend, CA, USA). All assays were conducted following standardized protocols provided by the manufacturers, with optical density measurements obtained using a microplate reader (TECAN, Männedorf, Switzerland) at recommended wavelengths.

Dual-Luciferase Reporter Assay

Luciferase activity was determined with the Dual-Luciferase Reporter Assay System (Promega, WI, USA) following the manufacturer's protocol. To account for transfection variability, firefly luciferase activity values were normalized to Renilla luciferase activity. Briefly, HEK-293T cells were co-transfected with IFNB1 firefly luciferase reporter plasmids, pRL-TK-Renilla-luciferase plasmid, and indicated overexpression plasmids. Luciferase activity was measured 24-36h post-transfection on a microplate reader (TECAN, Männedorf, Switzerland).

scRNA-seq Analysis and BayesPrism Deconvolution Analysis

ScRNA-seq data from three treatment-naïve TNBC samples (GSE176078: CID4465,

CID4495, CID4515) were retrieved from the GEO repository and analyzed using the Seurat package in R. Following quality control to remove low-quality cells, data were log-normalized and 2,000 highly variable genes were selected using the variance stabilizing transformation (vst) method. Principal component analysis was performed for dimensionality reduction, followed by *Harmony* integration to correct for batch effects across samples. Cell clusters were identified using the *FindNeighbors* and *FindClusters* functions and visualized via t-distributed stochastic neighbor embedding (t-SNE). Cluster annotation was performed based on established marker genes after differential expression analysis by *FindAllMarkers* function. Then samples were classified into "Hot tumors" (CID4495) and "Cold tumors" (CID4465, CID4515) groups according to T cell infiltration. Differential expression analysis between these groups within epithelial cells was conducted using the Wilcoxon rank sum test with the following criteria: absolute average $\log_2$ |fold change| > 0.5, adjusted p-value < 0.0001, and absolute difference in expression proportions between groups > 0.2.

BayesPrism deconvolution algorithm was utilized to construct epithelial cell gene expression matrix and infer cell type proportions from bulk RNA-seq data of the TCGA cohort (portal.gdc.cancer.gov/) as previously reported (1, 2). Briefly, prior knowledge integration was achieved using the above annotated scRNA-seq data. Protein-coding genes from bulk RNA-seq were deconvoluted using the *BayesPrism* R package after excluding uninformative genes. Cell type proportions and tumor epithelial cell-specific gene expression profiles were derived from the deconvolution results. Quality assessment included visualization of cell type relationships through pairwise

correlation matrices generated by the *plot.cor.phi* function, and examination of outlier gene distributions in both scRNA-seq and bulk RNA-seq data using the *plot.scRNA.outlier* and *plot.bulk.outlier* functions, respectively.

RNA High-Throughput Sequencing

Human (MDA-MB-468) or murine (4T1) TNBC cells were transfected with FBXW5-overexpression plasmids or control empty vectors for 48 hours. TRIzol™ reagent was used to extract the total RNAs from above cells. The concentration and purity of RNAs were examined by a NanoDrop 2000 (Thermo Fisher Scientific, Waltham, USA) and the integrity was evaluated using Agilent2100 (RRID:SCR_019715, Agilent, CA, USA). The cDNA libraries were constructed from high-quality RNA samples on the Illumina sequencing platform by Beijing Tsingke Biotech Co., Ltd. (Beijing, China). R package *DEseq2* (RRID:SCR_015687) was utilized to obtain differentially expressed genes with the criteria of fold change > 2 (4T1 cells) or 1.5 (MDA-MB-468 cells) and p value < 0.05. Significantly dysregulated genes were functionally annotated using Gene Ontology (GO) and Kyoto Encyclopedia of Genes and Genomes (KEGG, RRID:SCR_012773) pathway analyses.

SUPPLEMENTARY FIGURES

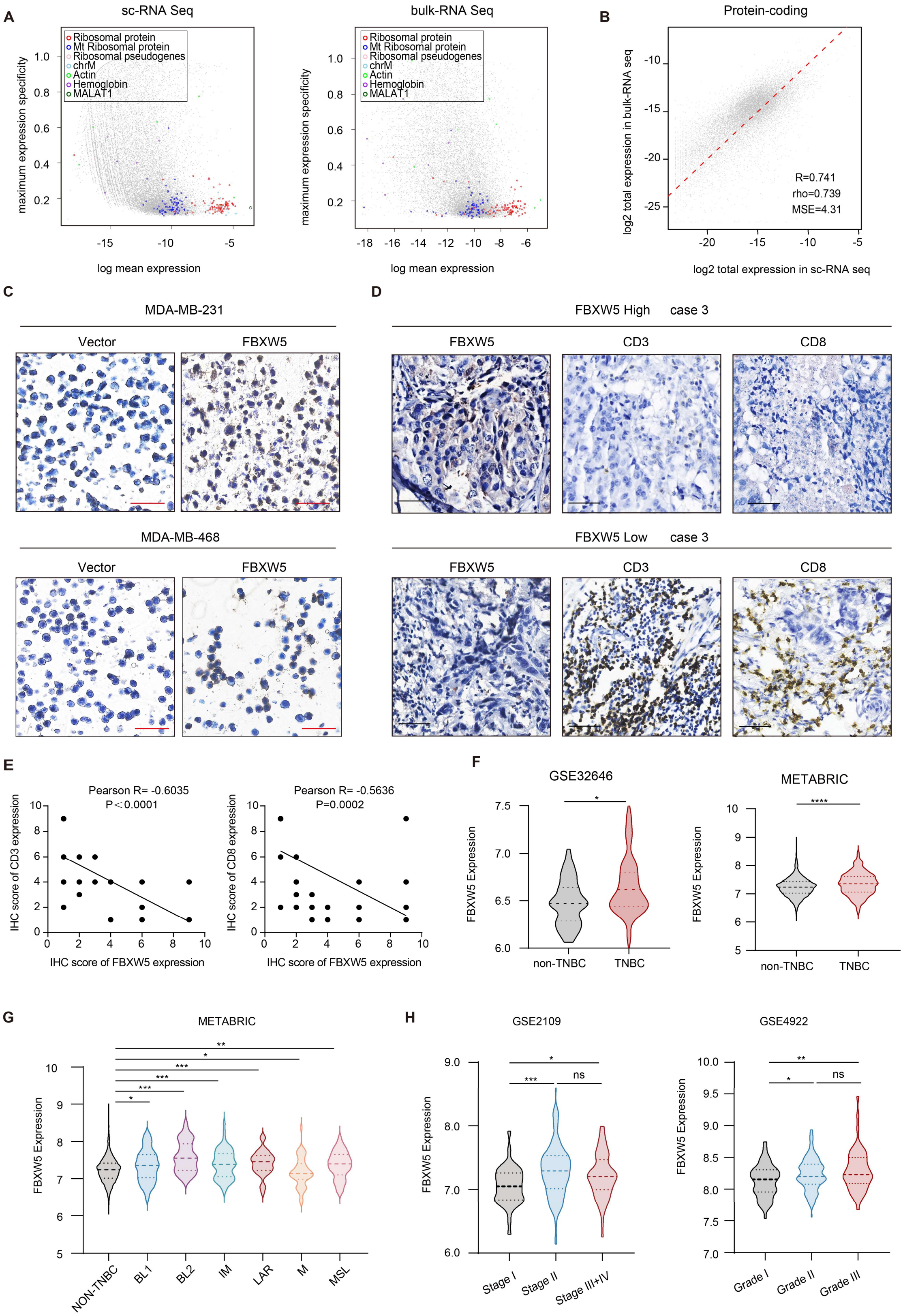

Supplementary Figure 1. Integration and expression analysis of FBXW5 in breast

cancer. (A and B) Visualization of the uninformative genes from the scRNA-seq data **(A)** and bulk RNA-seq data **(B)**. **(C)** IHC validation of FBXW5 antibody in control and *FBXW5*-overexpressing TNBC cells. The images are representative of 3 independent experiments (scale bars = 50 μm). **(D)** Representative images of IHC staining for FBXW5 and T-cell markers (CD3 and CD8) from 3 independent experiments. Scale bars = 50 μm. **(E)** Correlation analysis between FBXW5 and T-cell marker IHC scores. **(F)** Violin plot showing the expression level of FBXW5 in TNBC and non-TNBC samples from GEO database and METABRIC database. **(G and H)** *FBXW5* mRNA levels stratified by breast cancer molecular subtypes **(G)**, and by clinical stages or histological grades **(H)**. Statistical significance: Pearson correlation analysis (panel E); Mann-Whitney test (panels F); Kruskal-Wallis test followed by the two-stage linear step-up procedure of Benjamini, Krieger, and Yekutieli (panels G and H). *, $P < 0.05$; **, $P < 0.01$; ***, $P < 0.001$; ns, not significant.

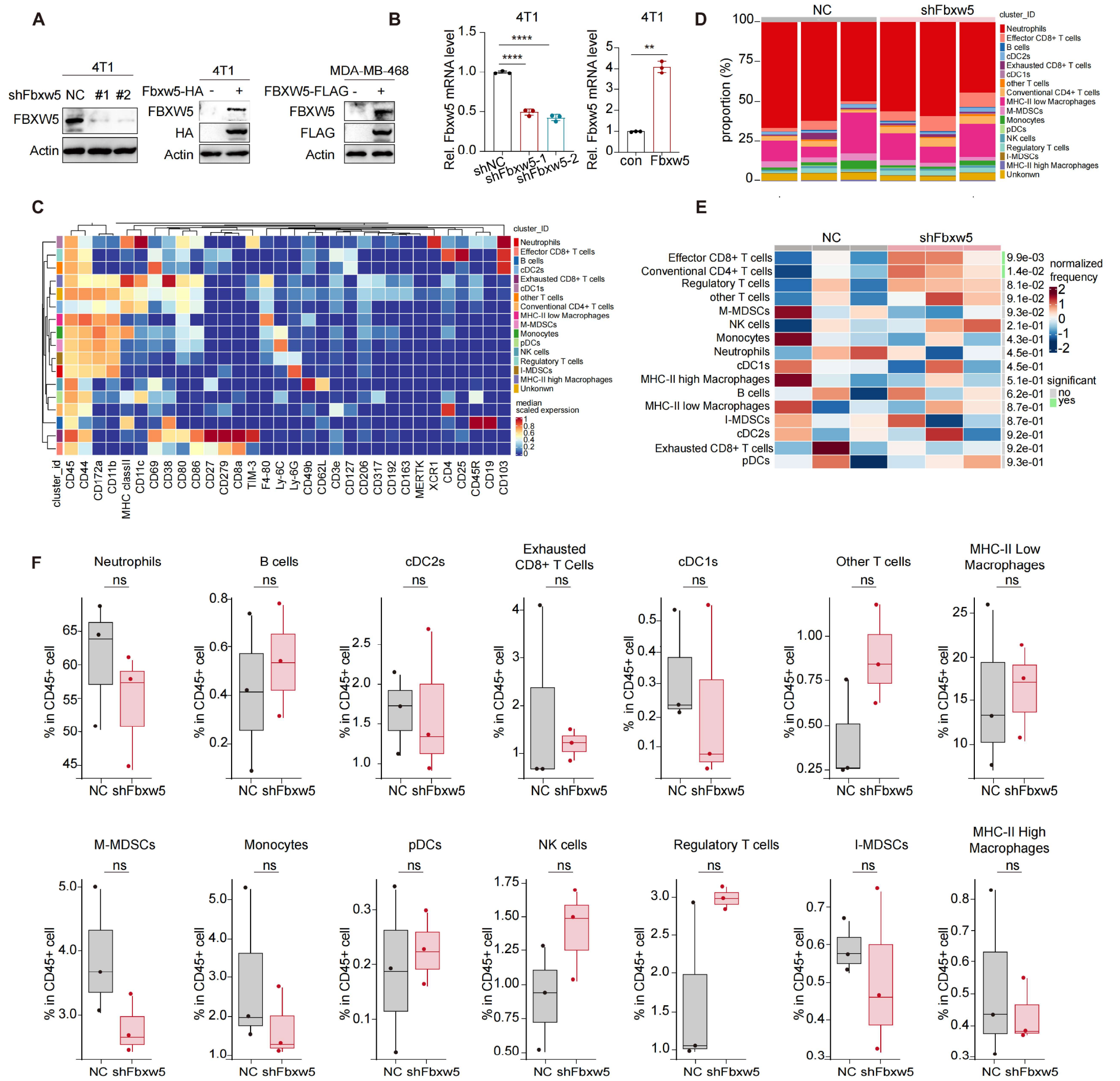

Supplementary Figure 2. FBXW5 correlates with an immunologically cold TME in TNBC. (A and B) RT-qPCR and Western blot analysis of *FBXW5* overexpression or knockdown efficiency in TNBC cells. **(C)** Heatmap displaying the median scaled expression of lineage-defining markers across 17 major clusters. **(D)** Bar graph illustrating the relative proportions of major immune cell clusters in control and murine *Fbxw5*-konckdown tumors from immunocompetent BALB/c mice. **(E)** Heatmap representing the normalized frequency and the statistical significance of specific immune cell clusters between two groups. **(F)** Box plots showing the percentage of indicated immune cell populations within the total CD45+ leukocyte population.

Statistical significance: unpaired two-tailed Welch's t-test (panels B, *Fbxw5* overexpression efficiency, and F); one-way ANOVA (panel B, *Fbxw5* knockdown efficiency) with Šidák’s multiple-comparison test. *, $P < 0.05$; **, $P < 0.01$; ns, not significant.

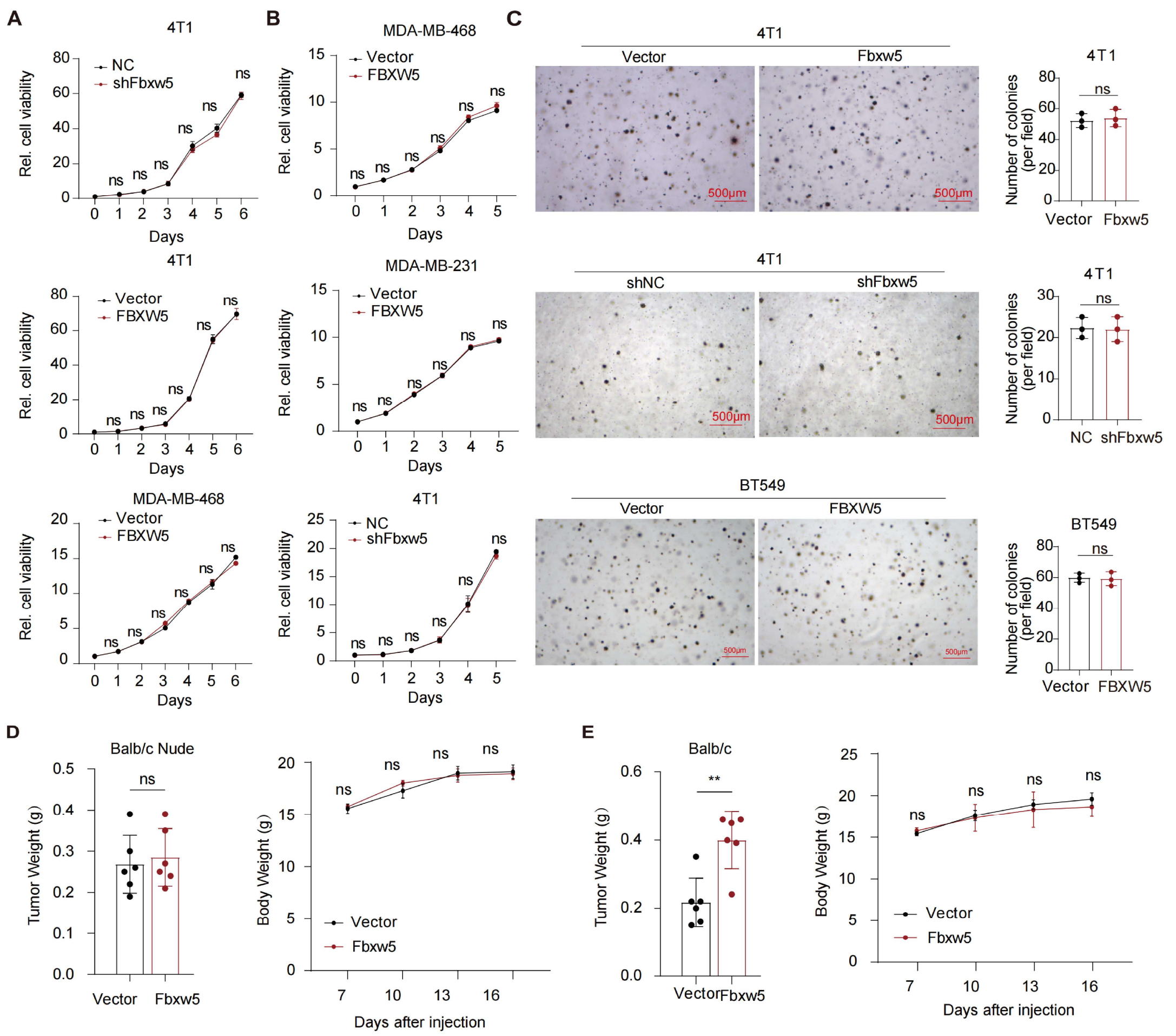

Supplementary Figure 3. FBXW5 does not alter TNBC cell proliferation. (A and B) Cell proliferation curves of indicated TNBC lines measured by MTT **(A)** and CCK-8 **(B)** following FBXW5 modulation. **(C)** Soft agar colony formation in 4T1 and BT549 cells upon FBXW5 modulation (murine or human, respectively). Representative images and quantification of colonies >50 μm are shown (n=3 independent experiments). **(D and E)** Final weights of subcutaneous 4T1 tumors and host body weights of Balb/c nude (T-cell deficient) **(D)** and Balb/c Immunocompetent mice **(E)**. Statistical significance was determined by unpaired two-tailed Welch's t-test. *$P < 0.05$, **$P < 0.01$, ***$P < 0.001$; ns, not significant.

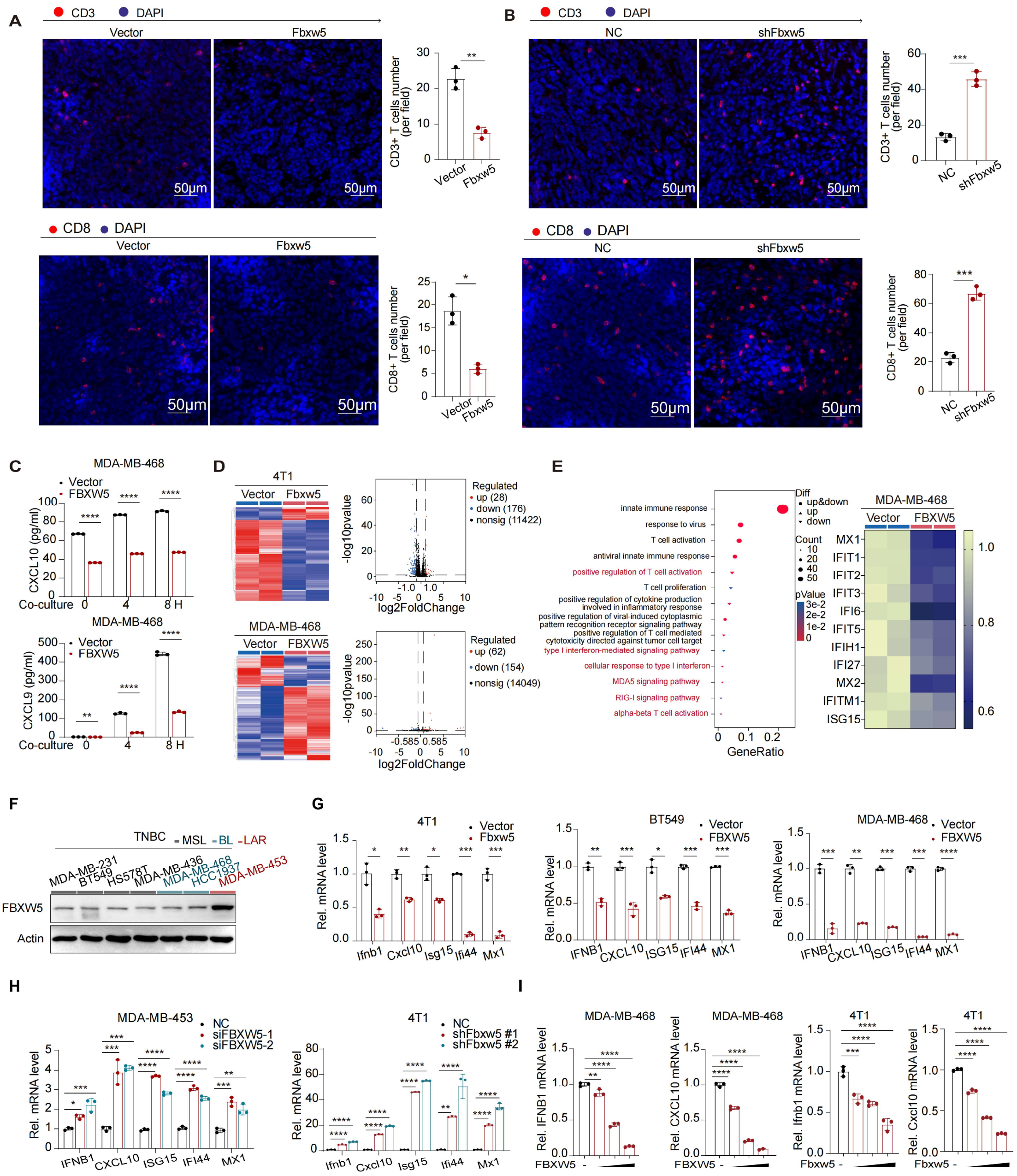

Supplementary Figure 4. FBXW5 inhibits T cell infiltration and negatively regulates type I IFN signaling pathway. (A and B) Representative images from 3 independent experiments and quantification of immunofluorescence staining for CD3+ and CD8+ T cells in tumor tissues from BALB/c immunocompetent mice. **(C)** ELISA of CXCL9 and CXCL10 levels in TNBC/T-cell culture medium. **(D and E)** Transcriptomic analysis of *FBXW5*-overexpressing TNBC cells: heatmap and volcano

plot of DEGs **(D)**, GO enrichment, and heatmap of downregulated IFN1 signaling genes **(E)**. **(F)** Western blot analysis of FBXW5 expression levels across a panel of TNBC cell line subtypes. **(G and H)** RT-qPCR of indicated genes across multiple TNBC lines following *FBXW5* overexpressing **(G)** or knockdown **(H)**. **(I)** RT-qPCR analysis demonstrating dose-dependent suppression of and *IFNB1* and *CXCL10* mRNA levels by FBXW5. Statistical significance: unpaired two-tailed Welch's t-test (panels A-C, G); one-way ANOVA (Panel H and I) with Šidák's multiple-comparison test. *, $P < 0.05$; **, $P < 0.01$; ***, $P < 0.001$; ****, $P < 0.0001$.

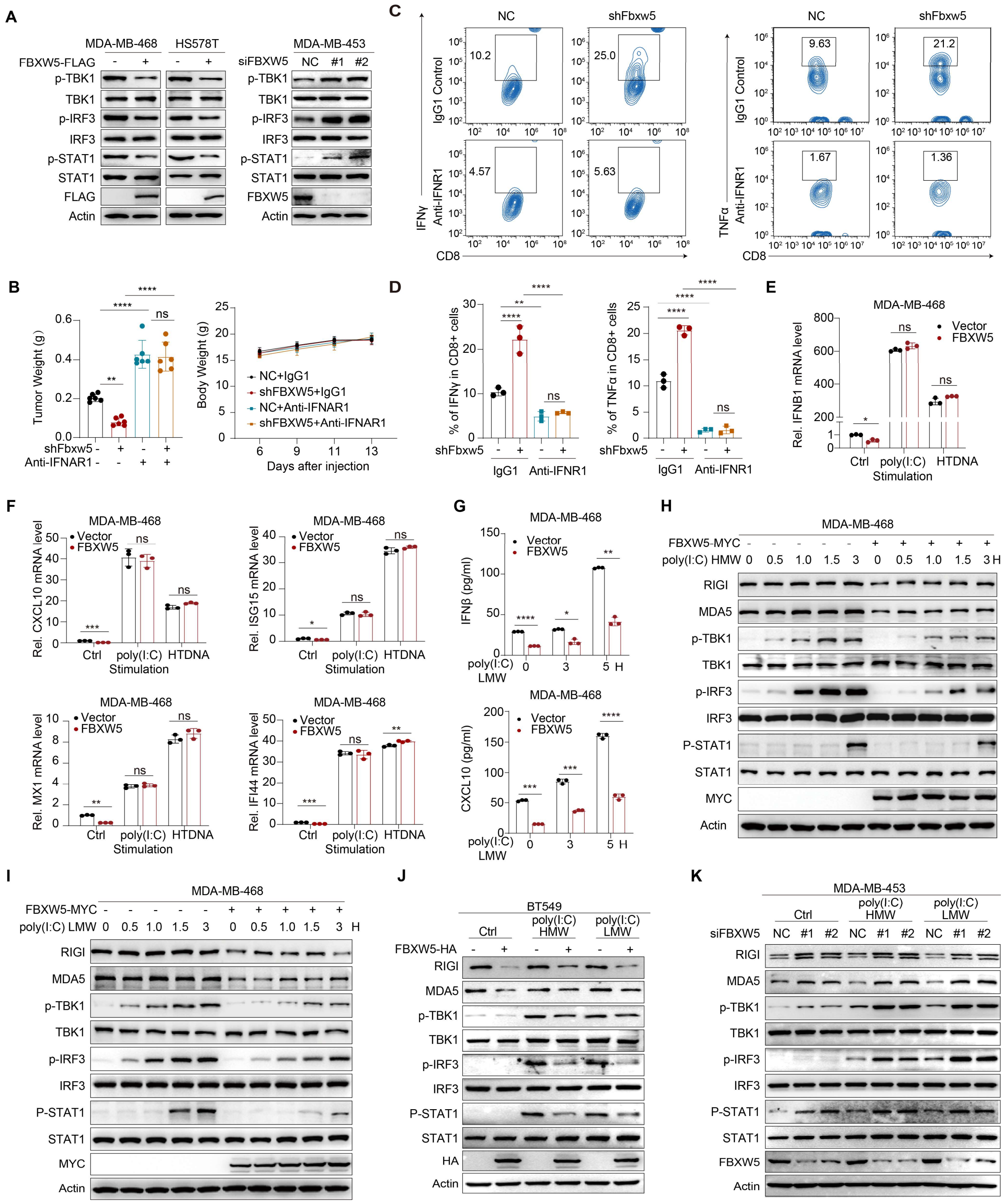

Supplementary Figure 5. FBXW5 promotes TNBC progression through negatively regulating type I IFN signaling pathway. (A) Western blot analysis assessing key type I IFN pathway components in *FBXW5*-modulated TNBC cells. **(B)** Final tumor and host body weights from immunocompetent BALB/c mice across experimental groups. **(C and D)** Flow cytometric quantification of effector subsets (IFN-γ+ or TNFα+) of tumor-infiltrating CD8+ T cells in BALB/c mice. **(E and F)** RT-qPCR

analysis of *IFNB1* and ISG mRNA levels in *FBXW5*-overexpressing MDA-MB-468 cells treated with extracellular LMW poly(I:C) incubation and HT-DNA transfection. **(G)** ELISA analysis of IFN-β and CXCL10 in TNBC cell supernatants. **(H-K)** Western blot analysis assessing key type I IFN pathway components across multiple TNBC lines in response to LMW or HMW poly(I:C). Statistical significance: unpaired two-tailed Welch's t-test (panels E-G); two-way ANOVA (Panel B and D) with Šidák's multiple-comparison test. *, $P < 0.05$; **, $P < 0.01$; ***, $P < 0.001$; ****, $P < 0.0001$; ns, not significant.

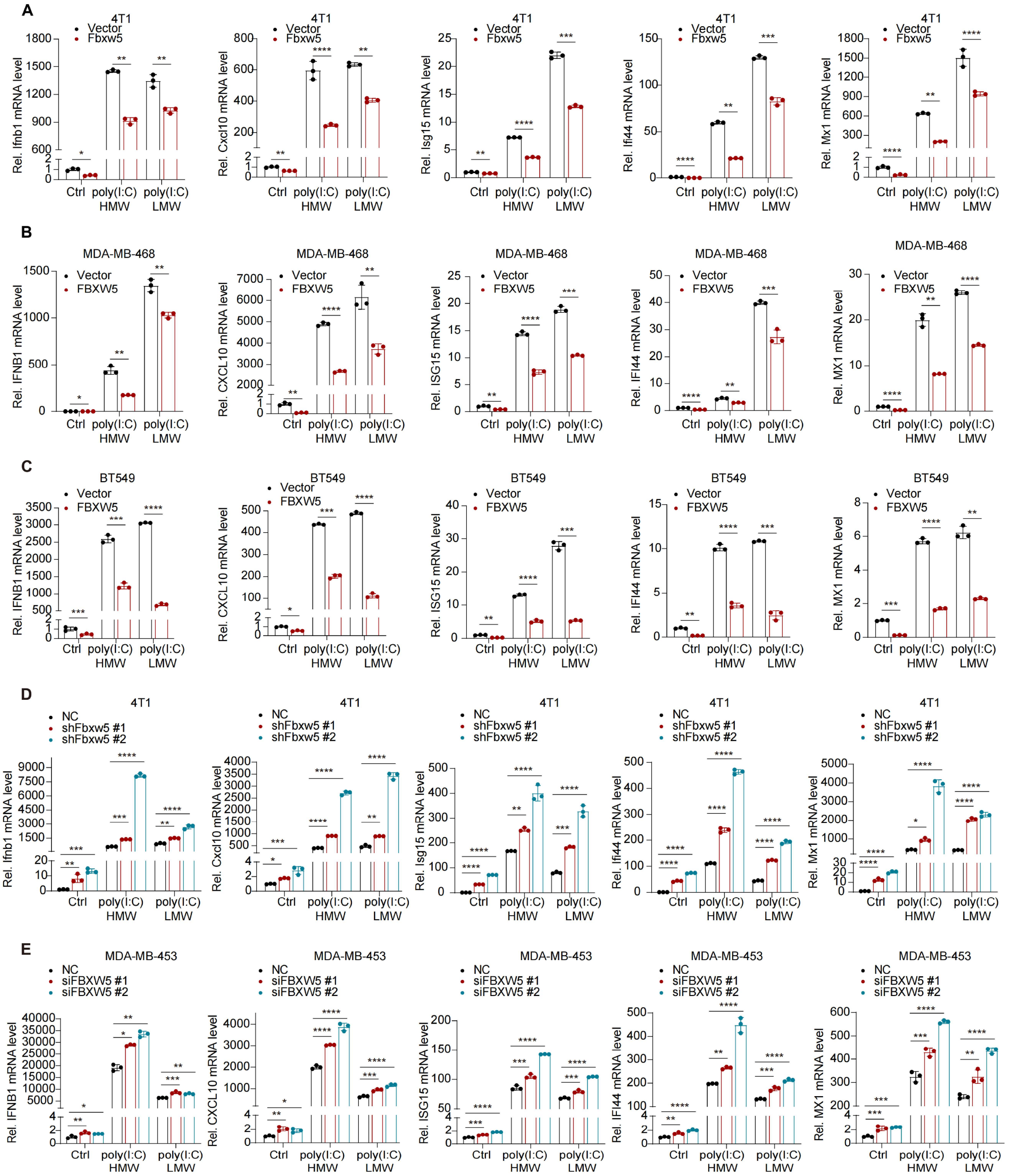

Supplementary Figure 6. FBXW5 negatively regulates dsRNA-dependent type I IFN signaling pathway. (A-C) RT-qPCR analysis of ISG mRNA levels in murine *Fbxw5*-overexpressing 4T1 **(A)**, human *FBXW5*-overexpressing MDA-MB-468 **(B)** and BT549 **(C)** cells treated with LMW poly(I:C) or HMW poly(I:C) transfection. **(D and E)** RT-qPCR analysis of ISGs mRNA levels in murine or human *FBXW5*-knockdown 4T1 **(D)** and MDA-MB-453 cells **(E)**. Statistical significance: unpaired

two-tailed Welch's t-test (panels A-C); one-way ANOVA (Panels D and E) with Šidák’s multiple-comparison test. *, $P < 0.05$; **, $P < 0.01$; ***, $P < 0.001$; ****, $P < 0.0001$.

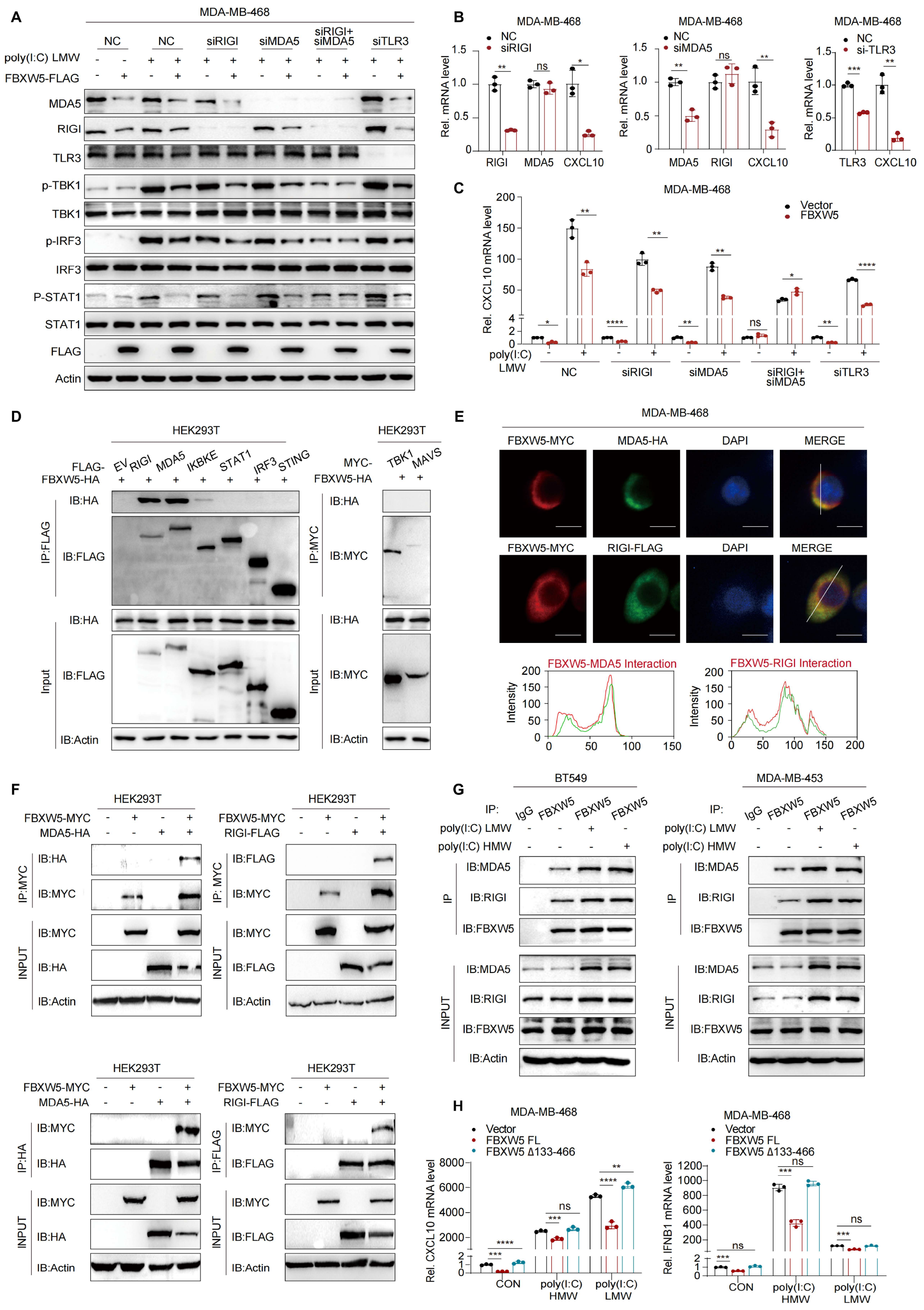

Supplementary Figure 7. FBXW5 physically interacts with MDA5 and RIGI. (A) Western blot analysis of indicated protein expression upon specified treatment. **(B)** RT-qPCR analysis of the indicated mRNA levels in MDA-MB-468 cells transfected with siRNAs targeting *RIGI*, *MDA5*, or *TLR3*. **(C)** RT-qPCR of *CXCL10* mRNA in MDA-

MB-468 cells with LMW Poly(I:C). **(D)** Co-IP assays assessing physical interactions with FBXW5 and key type I IFN pathway components. **(E)** Immunofluorescence of FBXW5-MYC (red) co-localizing with MDA5-HA/RIGI-FLAG (green). The images are representative of 3 independent experiments. Scale bars: 10 μm. **(F and G)** Exogenous **(F)** and Endogenous **(G)** co-IP analyses assessing interactions with FBXW5 and MDA5 and RIGI. **(H)** RT-qPCR of *CXCL10* and *IFNB1* mRNA levels in MDA-MB-468 cells expressing full-length FBXW5 or Δ133-466 mutant upon indicated treatment. Statistical significance: unpaired two-tailed Welch's t-test (panels B and C); one-way ANOVA (Panels H) with Šidák's multiple-comparison test. **, $P < 0.01$; ***, $P < 0.001$; ****, $P < 0.0001$; ns, not significant.

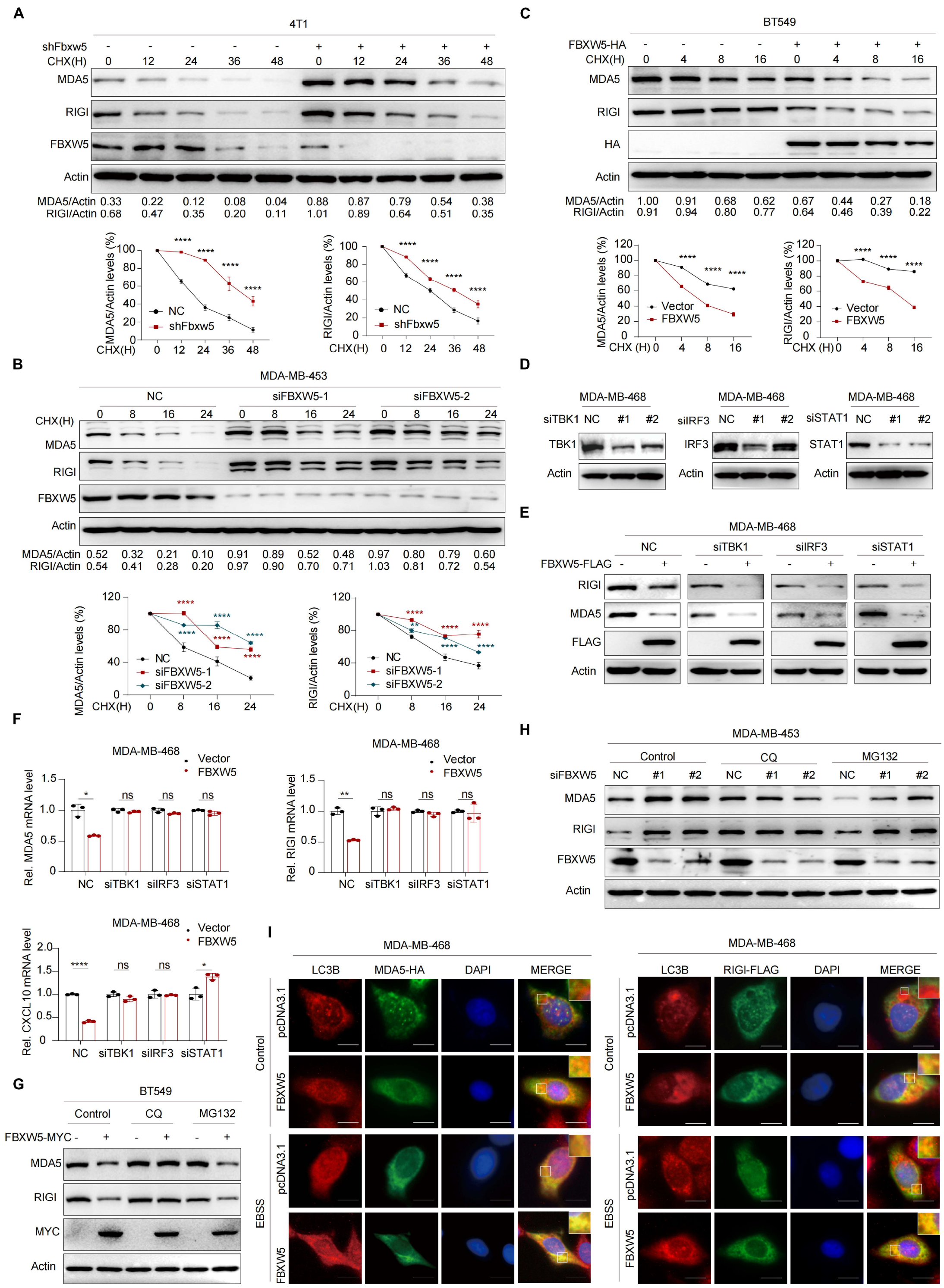

Supplementary Figure 8. FBXW5 orchestrates autophagic degradation of RIGI/MDA5 proteins. (A-C) Western blot analysis of indicated protein levels in *Fbxw5*-deficient 4T1 **(A)**, *FBXW5*-deficient MDA-MB-453 **(B)** and *FBXW5*-overexpressing BT549 **(C)** cells treated with cycloheximide (CHX, 50 μg/mL) for the

indicated durations. Relative protein levels were quantified using ImageJ and normalized to Actin (ImageJ). **(D)** Western blot analysis assessing the efficiencies of *TBK1*, *IRF3* or *STAT1* silencing. **(E and F)** Western blot **(E)** and RT-qPCR analysis **(F)** of indicated gene expression in MDA-MB-468 cells co-transfected with either empty vector (EV) or *FBXW5*-overexpressing plasmids, alongside siRNAs targeting *TBK1*, *IRF3*, *STAT1*, or a non-targeting control (NC). **(G and H)** Immunoblot of RIGI and MDA5 in cells treated for 8 h with autophagy inhibitors chloroquine (CQ, 100 μM) or proteasome inhibitor MG132 (10 μM). **(I)** Representative immunofluorescence images showing the colocalization of LC3B (red) co-localization with MDA5-HA (green) and RIGI-FLAG (green) in TNBC cells upon indicated treatments. The images are representative of 3 independent experiments. Scale bars: 10 μm. Statistical significance: unpaired two-tailed Welch's t-test (panel F); two-way ANOVA (Panels A-C) with Šidák's multiple-comparison test. *, $P < 0.05$; **, $P < 0.01$; ***, $P < 0.001$; ****, $P < 0.0001$; ns, not significant.

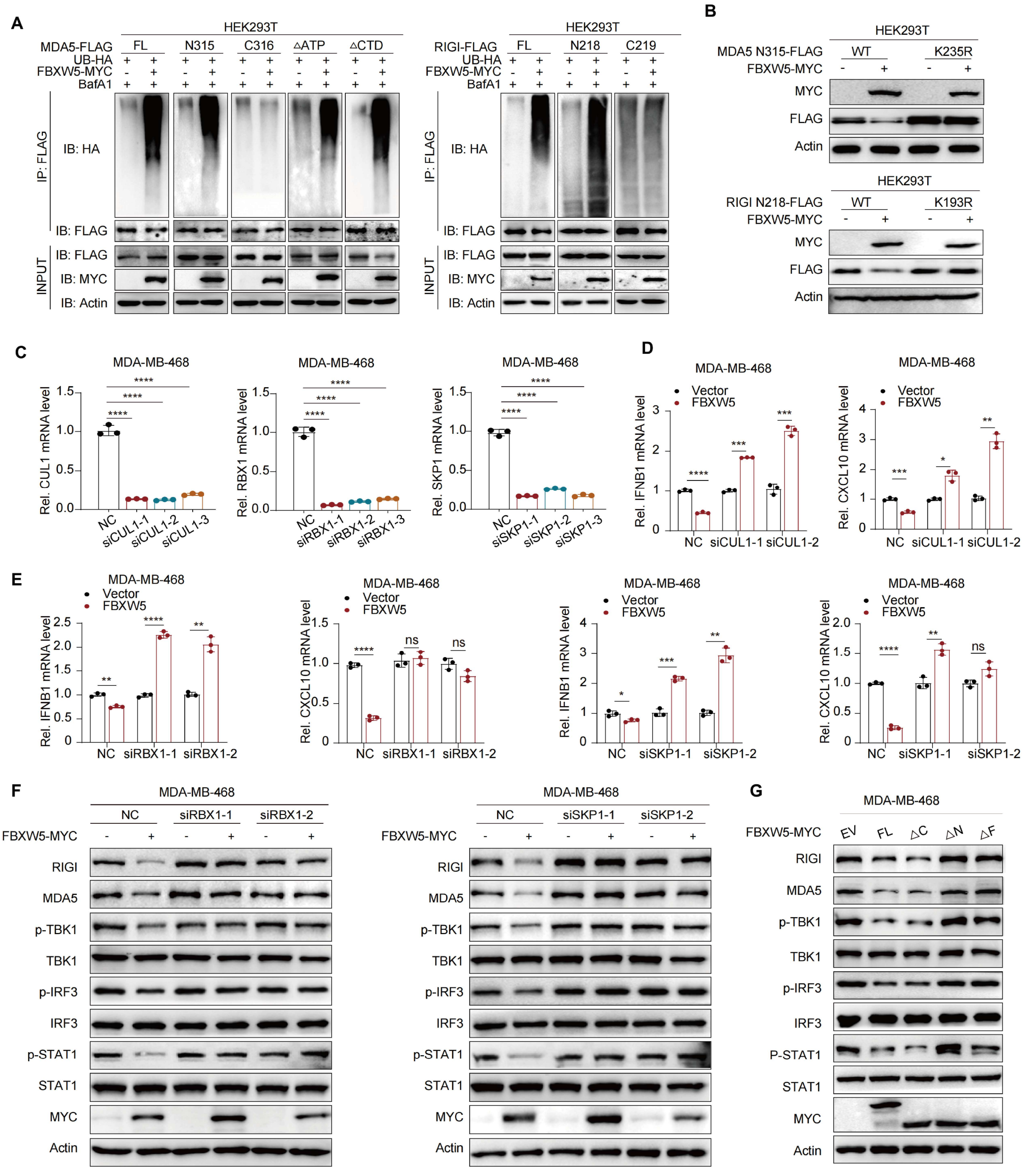

Supplementary Figure 9. FBXW5 downregulates RIGI and MDA5 protein dependent on SCF complex integrity. (A) Ubiquitination assays in HEK293T cells expressing full-length (FL) *MDA5*/*RIGI* or indicated truncations following BafA1 treatment. **(B)** Western blot analysis of wild-type (WT) and lysine-mutant (K to R) RLR N-terminal CARD-containing domains co-expressed with *FBXW5* in HEK293T cells. **(C)** RT-qPCR analysis of silencing efficiencies of *CUL1*, *RBX1* and *SKP1*. **(D-F)** RT-qPCR **(D and E)** and Western blot **(F)** analyses in MDA-MB-468 cells co-transfected

with EV or *FBXW5*-overexpressing plasmids alongside siRNAs targeting core SCF components, or negative control siRNAs. **(G)** Western blot analysis of indicated protein levels in MDA-MB-468 cells expressing FL or truncated FBXW5.Statistical significance: unpaired two-tailed Welch's t-test (panel D, E); one-way ANOVA (Panel C) with Šidák's multiple-comparison test. **, $P < 0.01$; ***, $P < 0.001$; ****, $P < 0.0001$; ns, not significant.

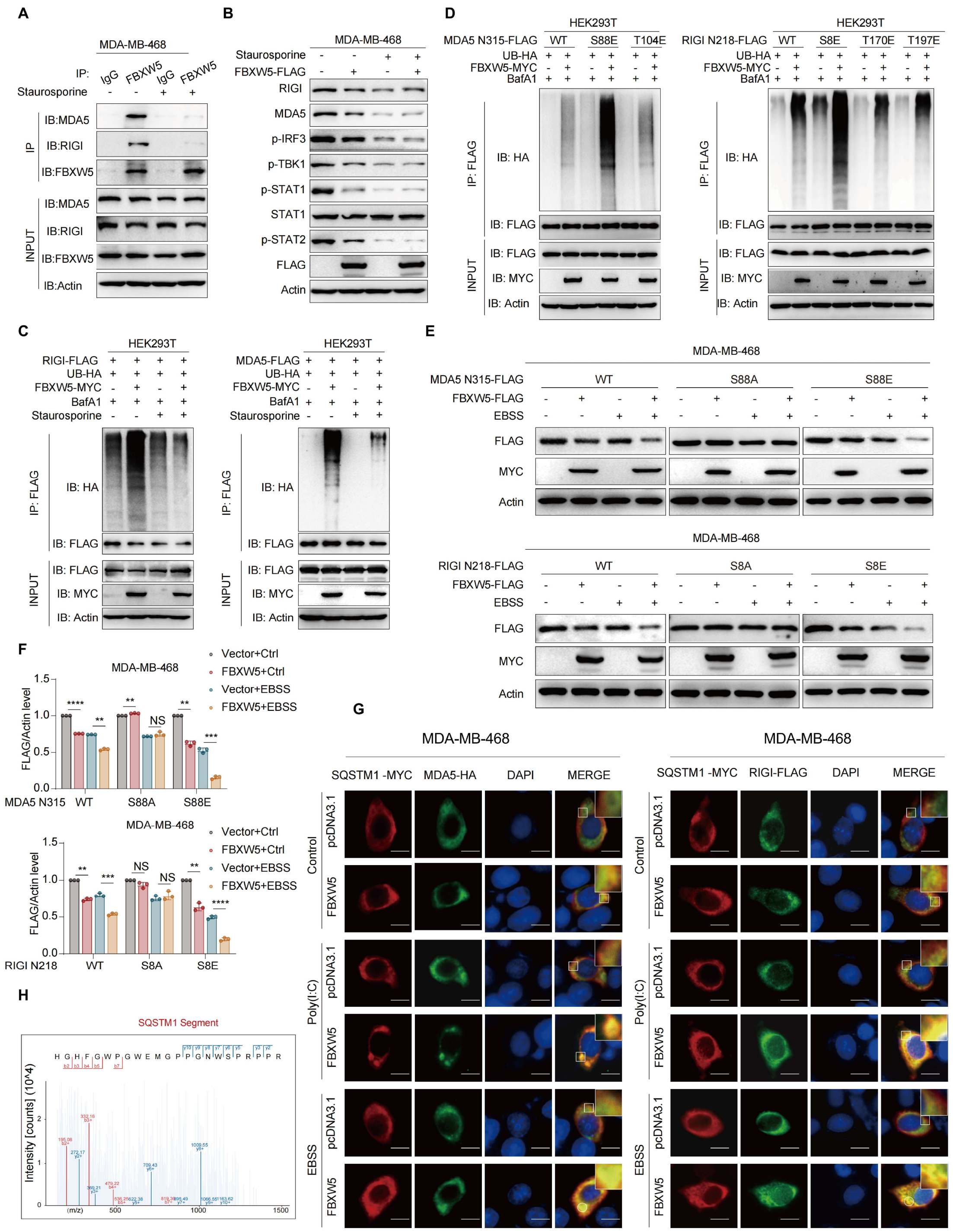

Supplementary Figure 10. FBXW5 regulates RLRs in a phosphorylation dependent-manner. (A) Co-IP analysis of FBXW5-RIGI/MDA5 interaction in TNBC cells following staurosporine treatment. **(B)** Western blot analysis of key RLR-sensing pathway components in MDA-MB-468 cells treated with staurosporine. **(C)** Ubiquitination assays in HEK-293T cells co-expressing FBXW5-MYC and

MDA5/RIGI-FLAG, with staurosporine and BafA1 treatment. **(D)** Ubiquitination assays in HEK293T cells expressing WT or phosphor-mimetic (S-to-E) mutants of the N-terminal CARD-containing truncations of *MDA5* or *RIGI* after BafA1 treatment. **(E and F)** Western blot analysis of wild-type (WT) and phospho-dead or phospho-mimetic mutations of RLR N-terminal CARD-containing domains co-expressed with FBXW5 in MDA-MB-468 cells. **(G)** Representative immunofluorescence of SQSTM1-MYC (red) co-localizing with MDA5-HA/RIGI-FLAG (green) in TNBC cells treated with PBS, EBSS or poly(I:C) from 3 independent experiments. **(H)** Mass spectrometry analysis of FBXW5-specific immunoprecipitates identifying SQSTM1 as interacting partners. Scale bars: 10 μm. Statistical significance was determined by unpaired two-tailed Welch's t-test. **, $P < 0.01$; ***, $P < 0.001$; ****, $P < 0.0001$; ns, not significant.

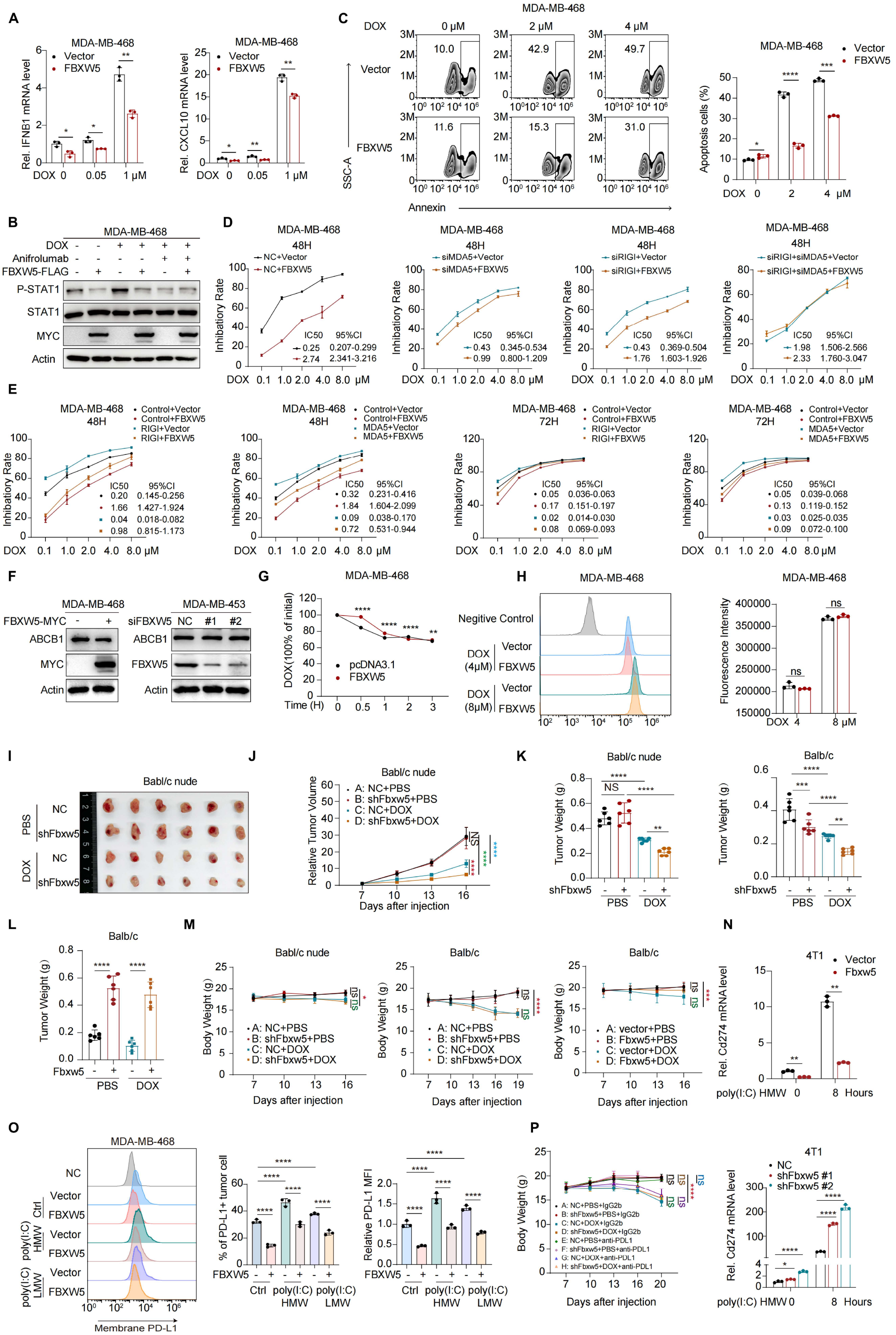

Supplementary Figure 11. FBXW5 enhances chemotherapy efficacy and regulates PD-L1 expression in TNBC. (A) RT-qPCR of *IFNB1* and *CXCL10* mRNA in MDA-

MB-468 cells transfected with EV or FBXW5-OE plasmids, followed by DOX treatment. **(B)** Western blot analysis of indicated proteins in transfected TNBC cells treated with DOX or the IFNAR1-blocking antibody anifrolumab. **(C)** Flow cytometry of apoptosis levels in TNBC cell followed by DOX treatment. **(D and E)** Concentration-inhibition rate curves of TNBC cells under indicated treatment followed by DOX treatment. IC50 with 95% confidence intervals (CI) indicated. **(F)** Western analysis of indicated protein in FBXW5-moducated TNBC cells. **(G and H)** DOX efflux **(G)** and accumulation **(H)** assays in MDA-MB-468 cells over the indicated time courses. **(I-L)** Subcutaneous tumor analysis in BALB/c nude or immunocompetent BALB/c mice: photographs **(I)**, growth curves **(J)**, and final tumor weights **(K and L)** across indicated groups. **(M)** Mice body weights for the indicated experimental groups. **(N and O)** PD-L1 expression determined by RT-qPCR **(N)** and flow cytometry **(O)** in TNBC cells under specified conditions. **(P)** Longitudinal monitoring of mouse body weights across treatment groups. Statistical significance: unpaired two-tailed Welch's t-test (panels A, C and H); two-way ANOVA (Panels G, and J-P) with Šidák's multiple-comparison test. Group comparisons: black (A vs. B), green (C vs. D), red (A vs. C), brown (E vs. F), purple (G vs. H), blue (A vs. E). *, $P < 0.05$; **, $P < 0.01$; ***, $P < 0.001$; ****, $P < 0.0001$; ns, not significant.

Supplementary Table 1: Multi-dimensional scoring of 32 prognostic genes

Gene symbol	Druggability[a]	Immunological Novelty[b]	Publication Density[c]	Pro-tumorigenic evidence[d]	Total Score
FLYWCH2	0	1	3	0	4
TMEM134	0	1	3	0	4
FAM110A	0	0	3	1	4
ZDHHC12	0	0	3	1	4
TRAPPC6A	1	1	3	0	5
BOLA1	1	1	3	0	5
RABL6	1	0	3	1	5
**FBXW5**	1	1	3	1	**6**
TMED3	1	0	3	1	5
PPP4C	1	0	3	1	5
RNASEH2C	1	0	3	1	5
DCXR	1	0	3	1	5
PTTG1IP	1	0	2	1	4
COASY	1	0	2	1	4
NAPRT	1	1	2	1	5
LRP10	1	0	2	1	4
AHSA1	1	0	2	1	4
BCKDK	1	0	2	1	4
GLRX5	1	0	2	1	4
PMEPA1	1	0	2	1	4
ARHGDIA	1	1	1	0	3
ITGB5	1	0	1	1	3
PFKL	1	0	1	1	3
KRT16	1	0	1	1	3
SCGB2A2	1	0	1	1	3
EZR	1	0	1	1	3
PLIN3	1	0	1	1	3
TNFRSF12A	1	0	1	1	3
SLC7A5	1	0	0	1	2
SDC1	1	0	0	1	2
JUNB	1	0	0	1	2
SPP1	1	0	0	1	2

Scoring criteria:

a. Druggability: Classified via the Pharos database (https://pharos.nih.gov); 1 for Tchem or Tbio (known ligands or biology) and 0 for Tdark (poorly characterized).

b. Prior Immunological Characterization: Assessed via PubMed database; 1 indicates no prior reports linking the gene to anti-tumor immunity or TME regulation, while 0 denotes established roles.

c. Publication Density: Quantified by the number of peer-reviewed articles retrieved from PubMed using the search term "[Gene Symbol] " at the time of scoring. 3 for <50, 2 for 50-100, 1 for 100-500, and 0 for >500.

d. Pro-tumorigenic evidence: 1 for genes with experimentally validated oncogenic functions in any malignancy; 0 for those with uncharacterized or purely observational roles.

Supplementary Table 2: Sequences of nucleic acids used in this study

**shRNA and siRNA sequences**		
Fbxw5	shRNA-1	GCGGCUCUUUAAGAUCCAGAAUUCAAGAG AUUCUGGAUCUUAAAGAGCCGC
	shRNA-2	GGGAUGAGUUCUUAUGGAAGGUUCAAGAG ACCUUCCAUAAGAACUCAUCCC
FBXW5	siRNA-1	AGCGCAUGCUAGAGACCAA
	siRNA-2	CAACAAGGACGACUCGCUA
RIGI	siRNA	GUAAACAACACCCGUACAATT
MDA5	siRNA	GGAGUUAUCGAACAUGAGATT
TLR3	siRNA	GCGCUUUAAUCCCUUUGAUTT
ATG5	siRNA	GCCAUCAAUCGGAAACUCATT
SQSTM1	siRNA-1	GCAUUGAAGUUGAUAUCGA
	siRNA-2	CCAGACUACGACUUGUGUA
TBK1	siRNA-1	GGCACAACAUUUCCCUAAATT
	siRNA-2	CGGCAGAGUUAGGUGAAAUTT
IRF3	siRNA-1	CUACGAGUUUGUGAACUCATT
	siRNA-2	CAGGAGGAUUUCGGAAUCUTT
STAT1	siRNA-1	GUAUAGAGCAUGAAAUCAA
	siRNA-2	CGAACAUGACCCUAUCACA
CUL1	siRNA-1	CAGCAUGAUCUCCAAGUUA
	siRNA-2	GGUUAUAUCAGUUGUCUAA
	siRNA-3	GGUGAAACCAUCAAUACAA
SKP1	siRNA-1	GAGAUUCGCAAGACCUUCA
	siRNA-2	CCGAACACCAUGCCUUCAA
	siRNA-3	GCGAACAGAUGAUAUCCCU
RBX1	siRNA-1	GGGAUAUUGUGGUUGAUAA
	siRNA-2	GCAUAGAAUGUCAAGCUAA
	siRNA-3	CUGCAUCUCUCGCUGGCUCAA

**Mutagenesis primers**		
FBXW5-MYC 1-132	F	GTCCGCGCTGTGCAGC
	R	GAGCAGAAACTCATCTCTGAAGAGG
FBXW5-MYC 133-466	F1	CATCCACCACACTGGACTAGTGG
	R1	ATGCGGCCCTACAACTGGA
	F2	GTAGGCGCGGTGCGCA
	R2	GAGCAGAAACTCATCTCTGAAGAGG
FBXW5-MYC 467-566	F	CATCCACCACACTGGACTAGTGG
	R	ACGCCCAACGACGAGTGC
FBXW5-MYC ΔC	F	GTAGGCGCGGTGCGCA
	R	GAGCAGAAACTCATCTCTGAAGAGG
FBXW5-MYC ΔN	F	CATCCACCACACTGGACTAGTGG
	R	ATGCGGCCCTACAACTGGA
FBXW5-MYC ΔF	F	CATCCACCACACTGGACTAGTGG
	R	GTGGAGGTGCAGACGCTGC
RIGI-FLAG ΔC1	F	CATGGTGGCGAATTCCGAGTTC
	R	GCCATTGAAAGTTGGGATTTCAAAA
RIGI-FLAG ΔC2	F	ACTTTCAATGGCTTCATAAAGTCCAGAA
	R	CTTGCTTTGGAGAAAGAAAGGAACA
RIGI-FLAG CARD	F	TTCTGGATCTTCTTGGTAGAAAATCTGTA
	R	GACTACAAAGACCATGACGGTGATTAT
RIGI-FLAG ΔCARD	F	GACTACAAAGACCATGACGGTGATTAT
	R	TGCCAGAATCTTAGTGAGAATTCATGT
MDA5-FLAG CARD	F	GACTACAAAGACCATGACGGTGATTATAAA
	R	AACTTCCATTTGGTAAGGCCTGAGC
MDA5-FLAG ΔCARD	F	GCCCAGCCAGCCTTGGAA
	R	CATGGTGGCGAATTCCACCA
MDA5-FLAG ΔC1	F	GAGCTCACGGACTTGCCCTCTC
	R	GGAATACCCATTCGACATGGTGG
MDA5-FLAG ΔC2	F	AATGAACTTGTCCAAGAGTTAACAGGCTC
	R	GTTCTCAAACGATGGAGAGGGCAAG
MDA5-FLAG ΔATP	F	TGTGCCAATCTTGATGCATTTACTATTAA
	R	AACTTCCATTTGGTAAGGCCTGAGC
MDA5-FLAG ΔCTD	F	GACTACAAAGACCATGACGGTGATTATAA
	R	TTCATTTTCATATTCTGGGTTTTCAGC
SQSTM1-MYC ΔPB1	F	CATGGTGGCGAATTCCACCA
	R	GCCATGTCCTACGTGAAGGATGA
SQSTM1-MYC ΔLIR	F	CTCGGACTCCAAGGCGATC
	R	CTGTCTTCAAAAGAAGTGGACCCG
SQSTM1-MYC ΔUBA	F	TGGGTACAAGGCAGCTTCCT
	R	CTCGAGGAACAAAAACTAATATCAGAGGAAG
RIGI CARD-K48R	F	GTTGTTTTTCTCAGCCTGAATATACTGC
	R	AGGGGCCCAATGGAGGCTG

RIGI CARD-K99R	F	TTCAATTTTTTTGAAATCCCAACTTTC
	R	AGGTTGGAGGAGTATAGATTACTTTTAAAACGTT
RIGI CARD-K154R	F	CTCTGCACCTGCCATCATCCCC
	R	AGGTTGGTGGAATGCCTTCTCAGAT
RIGI CARD-K164R	F	GTCTGATCTGAGAAGGCATTCCACCAA
	R	AGGGAAAACTGGCCCAAAACTTTGA
RIGI CARD-K169R	F	GGGCCAGTTTTCCTTGTCTGATCTGA
	R	AGGACTTTGAAACTTGCTTTGGAGAAAGAA
RIGI CARD-K172R	F	CAAAGTTTTGGGCCAGTTTTCCTTGT
	R	AGGCTTGCTTTGGAGAAAGAAAGGAAC
RIGI CARD-K181R	F	GTTCCTTTCTTTCTCCAAAGCAAGTT
	R	AGGTTCAGTGAACTGTGGATTGTAGAGAAAG
RIGI CARD-K190R	F	CTCTACAATCCACAGTTCACTGAACTTG
	R	AGGGGTATAAAAGATGTTGAAACAGAAGATCTTG
RIGI CARD-K193R	F	TATACCTTTCTCTACAATCCACAGTTCACT
	R	AGGGATGTTGAAACAGAAGATCTTGAGGATAA
MDA5 CARD-K23R	F	AGGATGTACATCCAGGTGGAGCCT
	R	CACCCTGGCCCTGAAGCAC
MDA5 CARD-K43R	F	AGGGAGCAGATTCAGAGGACAGTCG
	R	CACCTCTGCAGGCAGAAAGGTCA
MDA5 CARD-K68R	F	AGGGGAGTCTGGCACCTTGGTT
	R	CTCCAAGGTGCTCAGCAGCAGTT
MDA5 CARD-K128R	F	AGGCTTCTAGTTAGAGACGTCTTGGATAAG
	R	GTCCACCAGAGTGGGCTGAAGG
MDA5 CARD-K137R	F	AGGTGCATGGAGGAGGAACTGTTG
	R	ATCCAAGACGTCTCTAACTAGAAGCTTGTC
MDA5 CARD-K169R	F	AGGAGGATTGTGCAGAAAGAAAACTG
	R	TAGTAGCTCTCTTACACCTGATTCATTTCCA
MDA5 CARD-K174R	F	AGGGAAAACTGGTTCTCTGCATTTCT
	R	CTGCACAATCCTTTTTAGTAGCTCTCTTACA
MDA5 CARD-K193R	F	AGGATGTACATCCAGGTGGAGCCT
	R	CACCCTGGCCCTGAAGCAC
RIGI CARD-S8A	F	GAACTGCAAGCCTTCCAGGATTATATCCG
	R	GCGTCGCTGCTCGGTGGTC
RIGI CARD-S8E	F	GAACTGCAAGCCTTCCAGGATTATATCCG
	R	GCGTCGCTGCTCGGTGGTC
RIGI CARD-T170A	F	GCGTTGAAACTTGCTTTGGAGAAAGAAAGGAA
	R	TTTGGGCCAGTTTTCCTTGTCTGAT
RIGI CARD-T170E	F	GAATTGAAACTTGCTTTGGAGAAAGAAAGGAA
	R	TTTGGGCCAGTTTTCCTTGTCTGAT
RIGI CARD-T197A	F	GCGGAAGATCTTGAGGATAAGATGGAAACTTCTGAC
	R	TTCAACATCTTTTATACCTTTCTCTACAATCCA

RIGI CARD-T197E	F	GAAGAAGATCTTGAGGATAAGATGGAAACTTCTGAC
	R	TTCAACATCTTTTATACCTTTCTCTACAATCCA
MDA5 CARD-S88A	F	GCGCCTCTGGCCGCCCGCTACAT
	R	GCCGGTTCTCCGGAGGGC
MDA5 CARD-S88E	F	GAACCTCTGGCCGCCCGCTACAT
	R	GCCGGTTCTCCGGAGGGC
MDA5 CARD-S104A	F	GCGCCATCGTTTGAGAACGCTCATGATG
	R	GGGCAAGTCCGTGAGCTCAGGG
MDA5 CARD-S104E	F	GAACCATCGTTTGAGAACGCTCATGATG
	R	GGGCAAGTCCGTGAGCTCAGGG

qRT-PCR primers		
ACTB	F	CATGTACGTTGCTATCCAGGC
	R	CTCCTTAATGTCACGCACGAT
FBXW5	F	TCTGGAGCAACGACCTGAC
	R	GACAGCAATCTCGCCGGAT
IFNB1	F	GCTTGGATTCCTACAAAGAAGCA
	R	ATAGATGGTCAATGCGGCGTC
CXCL10	F	GTGGCATTCAAGGAGTACCTC
	R	TGATGGCCTTCGATTCTGGATT
ISG15	F	CGCAGATCACCCAGAAGATCG
	R	TTCGTCGCATTTGTCCACCA
IFI44	F	ATGGCAGTGACAACTCGTTTG
	R	TCCTGGTAACTCTCTTCTGCATA
MX1	F	GTTTCCGAAGTGGACATCGCA
	R	CTGCACAGGTTGTTCTCAGC
RIGI	F	TGTGCTCCTACAGGTTGTGGA
	R	CACTGGGATCTGATTCGCAAAA
MDA5	F	GCCCGCTACATGAACCCTG
	R	CAGCAATCCGGTTTCTGTCTT
TLR3	F	TCAACTCAGAAGATTACCAGCCG
	R	AGTTCAGTCAAATTCGTGCAGAA
CUL1	F	GGTTCGCCGTGAATGTGAC
	R	CCCCAATTCCACGTAAGACTGT
SKP1	F	GACCATGTTGGAAGATTTGGGA
	R	TGCACCACTGAATGACCTTTT
RBX1	F	TTGTGGTTGATAACTGTGCCAT
	R	GACGCCTGGTTAGCTTGACAT
Ifnb1	F	AGCTCCAAGAAAGGACGAACA
	R	GCCCTGTAGGTGAGGTTGAT
Cxcl10	F	ATCATCCCTGCGAGCCTATCCT
	R	GACCTTTTTTGGCTAAACGCTTTC
Isg15	F	GGTGTCCGTGACTAACTCCAT
	R	CTGTACCACTAGCATCACTGTG
Ifi44	F	CCTGGTTCAGCAAACACGAGT
	R	TGGCCTTGATGGAATATGTCCT
Mx1	F	TGGACATTGCTACCACAGAGGC
	R	TTGCCTTCAGCACCTCTGTCCA
Fbxw5	F	GAGTTCCGGCGGCTCTATG
	R	AGATGGTCAGGTCGTTGTTCC
ACTB	F	TGTTACCAACTGGGACGACA
	R	CTGGGTCATCTTTTCACGGT

Supplementary Table3: Detailed information of reagents and antibodies

**Reagents**		
Reagents	Vendor	Catalog #
poly(I:C) HMW	Invivogen	tlrl-pic
poly(I:C) LMW	Invivogen	tlrl-picw
HtDNA	Sigma	D6898
Chloroquine	Selleck	S6999
MG132	Selleck	S2619
Leupeptin HemisuLfate	Selleck	S7380
Cycloheximide	Selleck	S7418
Bafilomycin A1	MedChemExpress	HY-100558
3-Methyladenine	MedChemExpress	HY-19312
Rapamycin	MedChemExpress	HY-10219
Doxorubicin	MedChemExpress	HY-15142
EBSS	Solarbio	H2045
Leukocyte Activation Cocktail	BD Pharmingen	550583

Antibodies

Western blot and Immunoprecipitation

Antibody	Vendor	Catalog #	RRID
anti-HA	Abcam	ab182009	
anti-p-TBK1	Abcam	ab109272	AB_10862438
anti-p-IRF3	Abcam	ab76493	AB_1523836
anti-STAT1	Abcam	ab281999	AB_3697421
anti-MDA5	Abclonal	A2419	AB_3674625
anti-TBK1	Abclonal	A3458	AB_2863061
anti-IRF3	Abclonal	A19717	AB_2862750
anti-MYC	Bethyl Laboratories	A190-105A	AB_67390
anti-mouse IgG	Cell Signaling Technology	7076	AB_330924
anti-rabbit IgG	Cell Signaling Technology	7074	AB_2099233
anti-RIGI	Cell Signaling Technology	D14G6	AB_2269233
anti-p-STAT1	Cell Signaling Technology	7649	AB_10950970
anti-TLR3	Proteintech	83136-3-RR	AB_3670837
anti-SQSTM1	Proteintech	18420-1-AP	AB_10694431
anti-FBXW5	Proteintech	18321-1-AP	AB_2878530
anti-Actin	Proteintech	66009-I-Ig	AB_2883225
anti-Flag	invitrogen	PA1-984B	AB_347227

Flow cytometry

Antibody	Vendor	Catalog #	RRID
APC Anti-Mouse CD3 Antibody	elabscience	E-AB-F1013E	AB_3675272
PE Anti-Mouse CD8 Antibody	Biolegend	126608	AB_961298
FITC Anti-Mouse CD69 Antibody	Biolegend	104506	AB_313109
PE Anti-Mouse CD274 Antibody	Biolegend	124308	AB_2073556
APC Anti-Human CD274 Antibody	Biolegend	329708	AB_940360
FITC Rat Anti-Mouse CD45 Antibody	BD Pharmingen	553079	AB_394609
RB705 Rat Anti-Mouse TNF Antibody	BD Pharmingen	570733	AB_3686007

PE-Cy™7 Rat Anti-Mouse IFN-γ Antibody	BD Pharmingen	557649	AB_396766
PE/Cyanine7 anti-human/mouse Granzyme B Recombinant Antibody	Biolegend	372214	AB_2728381
RB705 Mouse Anti-Human CD8 Antibody	BD Pharmingen	757861	AB_3690017
APC Mouse Anti-Human TNF Antibody	BD Pharmingen	562084	AB_10893226
PE-Cy™7 Mouse Anti-Human IFNγ Antibody	BD Pharmingen	561036	AB_2033977

CyTOF

Metal categories	Antibody	Vendor	Catalog #	RRID
89Y	CD45	Biolegend	147702	AB_2563416
115In	CD3ε	Biolegend	100302	AB_312667
141Pr	Ly-6G	Biolegend	127602	AB_1089180
142Nd	CD11c	Biolegend	117302	AB_313771
143Nd	CD45R/B220	Biolegend	103202	AB_312987
144Nd	CD172a	Biolegend	144002	AB_11203711
145Nd	XCR1	Biolegend	148202	AB_2563841
146Nd	F4/80	Thermo	MA5-16630	AB_2538126
147Sm	CD103	Biolegend	121402	AB_535945
148Nd	CD80	Biolegend	104702	AB_313123
149Sm	CD19	Biolegend	152402	AB_2629714
150Sm	CD86	Biolegend	105002	AB_313145
151Eu	CD206	Biolegend	141702	AB_10900233
152Sm	CD25	Biolegend	101902	AB_312845
153Eu	CD49b	IDEAL	YD49b153	
154Sm	CD127	Biolegend	135002	AB_1937287
155Gd	MERTK	Biolegend	151502	AB_2566624
157Gd	CD279/PD-1	IDEAL	YD279157	
159Tb	CD317	IDEAL	YD317159	
162Dy	CD366/TIM-3	Biolegend	119702	AB_345376
163Dy	CD192	Biolegend	160102	AB_2876563
164Dy	CD163	Biolegend	155302	AB_2734239
165Ho	CD62L	Biolegend	104402	AB_313089
166Er	CD8a	Biolegend	100702	AB_312741
167Er	CD27	Biolegend	124202	AB_1236456
169Tm	CD69	Biolegend	104502	AB_313105
170Er	CD38	Biolegend	102702	AB_312923
171Yb	CD44	Biolegend	103002	AB_312953
173Yb	MHC class II	Biolegend	107602	AB_313317

174Yb	CD4	Biolegend	100506	AB_312709
175Lu	Ly-6C	Biolegend	128002	AB_1134214
176Yb	CD11b	Biolegend	101202	AB_312785

IF/IHC

Antibody	Vendor	Catalog #	RRID
anti-LC3B	Proteintech	14600	AB_2919097
Mouse anti-HA	Abclone	AE008	AB_2770404
Mouse anti-Flag	Beyotime	AF2852	AB_2943049
anti-FBXW5	Proteintech	18321-1-A	AB_2878530
Mouse anti-CD3	Abclone	A19017	AB_2862509
Rabbit anti-CD8α	Abcam	ab217344	AB_2890649
Mouse anti-CD8α	Cell Signaling Technology	70306T	AB_2799781

References

1. Tran KA, Addala V, Johnston RL, Lovell D, Bradley A, Koufariotis LT, et al. Performance of tumour microenvironment deconvolution methods in breast cancer using single-cell simulated bulk mixtures. Nat Commun. 2023;14(1):5758.
2. Zhang Y, Zhang C, He J, Lai G, Li W, Zeng H, et al. Comprehensive analysis of single cell and bulk RNA sequencing reveals the heterogeneity of melanoma tumor microenvironment and predicts the response of immunotherapy. *Inflamm Res.* 2024;73(8):1393-409.
