# Supplementary material for: FBXW5 blunts RIGI/MDA5/IFN signaling to drive immune evasion and chemo-immune resistance in triple-negative breast cancer models
Source: J Clin Invest. 2026 Aug 25;136(20):e201573. doi: 10.1172/JCI201573 (PMC13668642; doi:10.1172/JCI201573)

Figure 3C

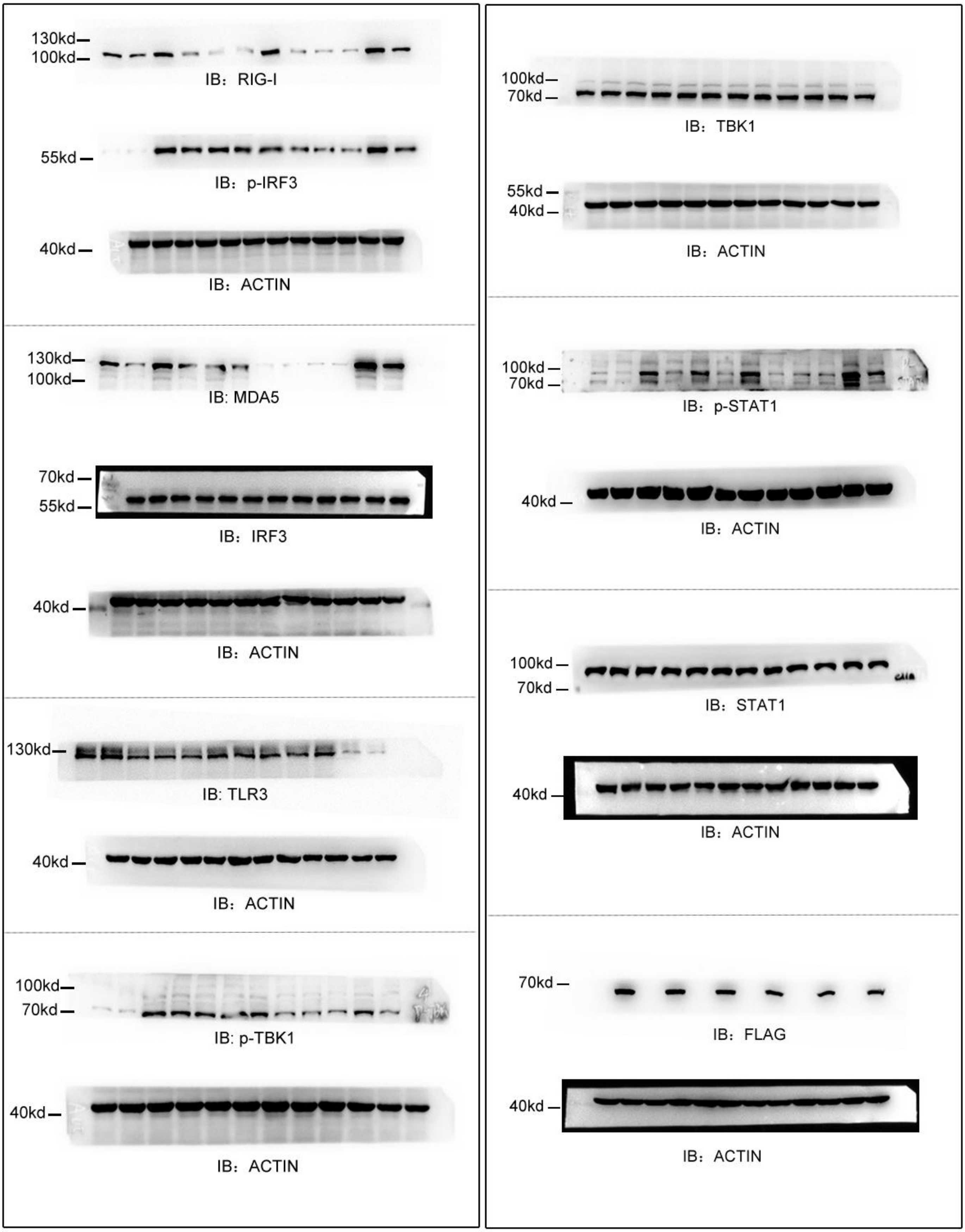

130kd
100kd
IB：RIG-I
55kd
IB：p-IRF3
40kd
IB：ACTIN
130kd
100kd
IB: MDA5
70kd
55kd
IB：IRF3
40kd
IB：ACTIN
130kd
IB: TLR3
40kd
IB：ACTIN
100kd
70kd
IB: p-TBK1
40kd
IB：ACTIN
100kd
70kd
IB：TBK1
55kd
40kd
IB：ACTIN
100kd
70kd
IB：p-STAT1
40kd
IB：ACTIN
100kd
70kd
IB：STAT1
40kd
IB：ACTIN
70kd
IB：FLAG
40kd
IB：ACTIN

Figure 3F

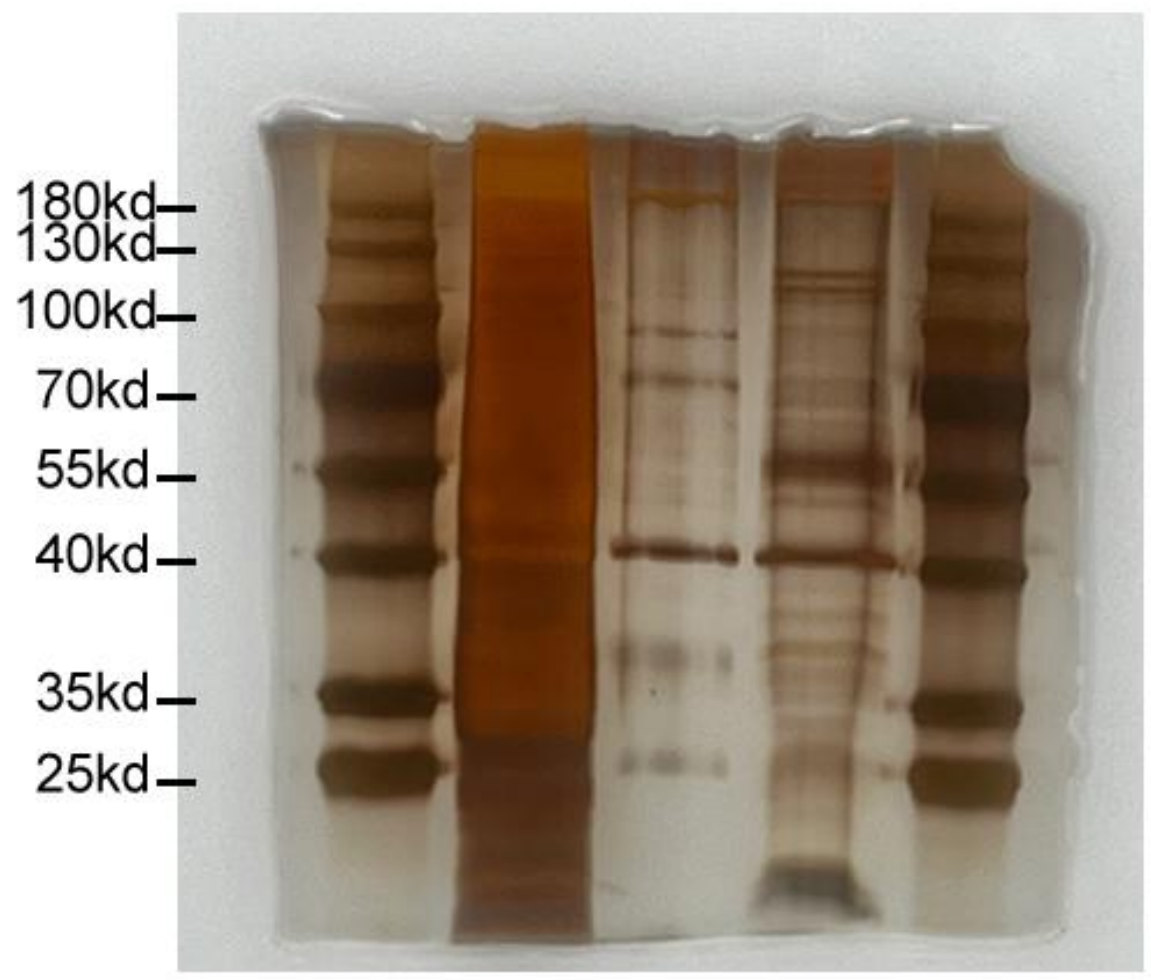

Figure 3G

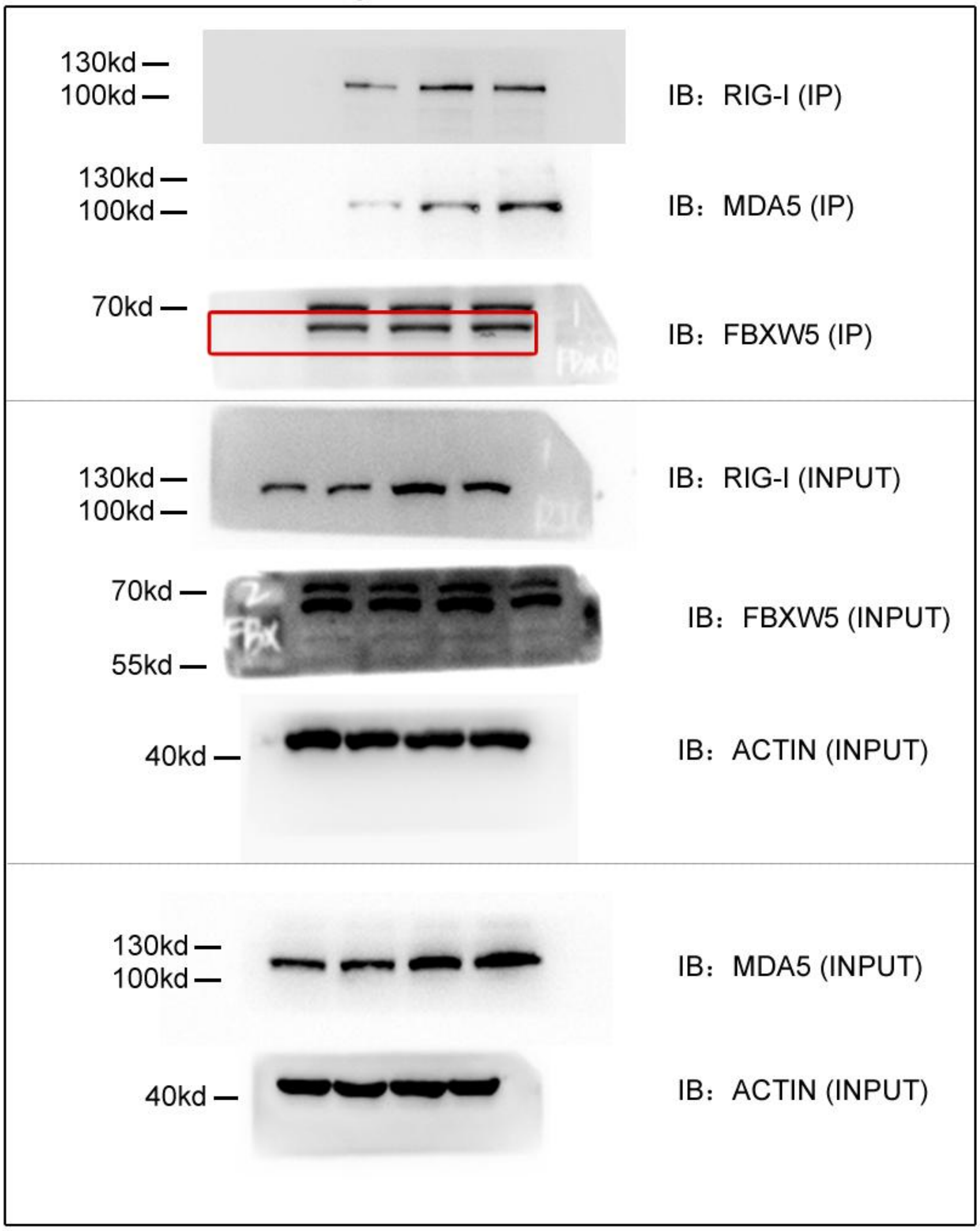

Figure 3I

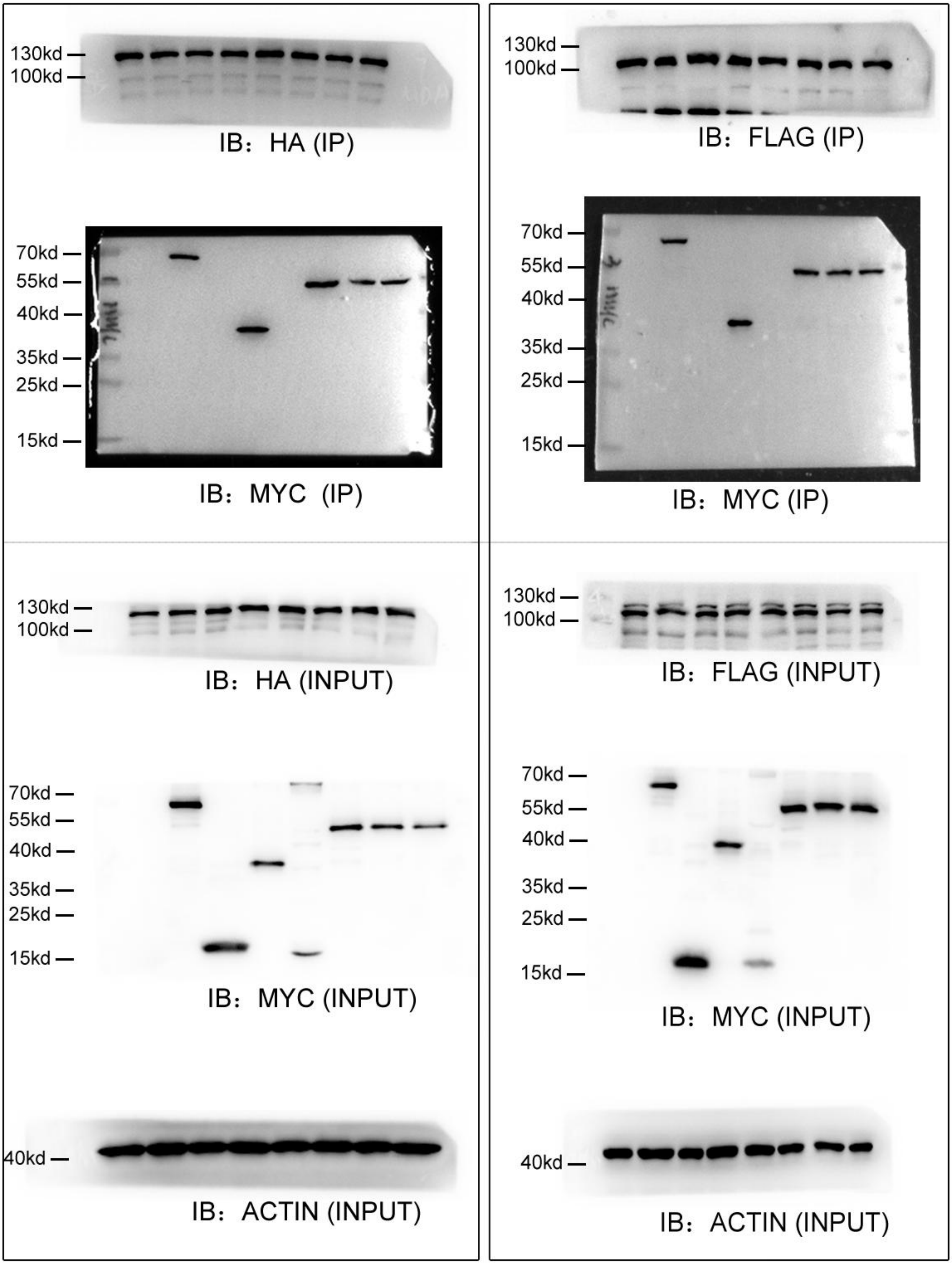
130kd
100kd
IB：HA (IP)
130kd
100kd
IB：FLAG (IP)
70kd
55kd
40kd
35kd
25kd
15kd
IB：MYC (IP)
70kd
55kd
40kd
35kd
25kd
15kd
IB：MYC (IP)
130kd
100kd
IB：HA (INPUT)
130kd
100kd
IB：FLAG (INPUT)
70kd
55kd
40kd
35kd
25kd
15kd
IB：MYC (INPUT)
70kd
55kd
40kd
35kd
25kd
15kd
IB：MYC (INPUT)
40kd
IB：ACTIN (INPUT)
40kd
IB：ACTIN (INPUT)

Figure 3J

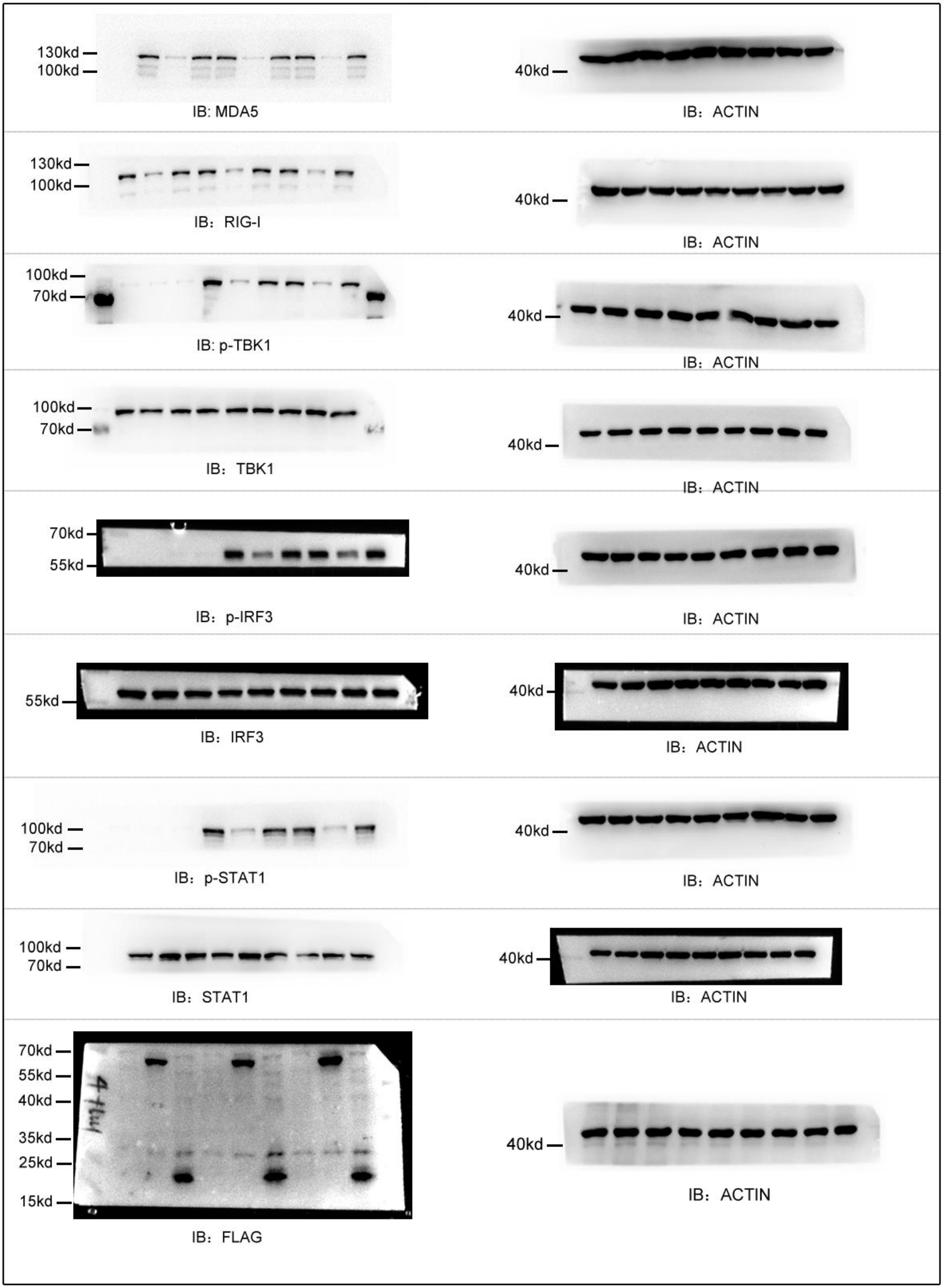
130kd
100kd
IB: MDA5
40kd
IB：ACTIN
130kd
100kd
IB：RIG-I
40kd
IB：ACTIN
100kd
70kd
IB: p-TBK1
40kd
IB：ACTIN
100kd
70kd
IB：TBK1
40kd
IB：ACTIN
70kd
55kd
IB：p-IRF3
40kd
IB：ACTIN
55kd
IB：IRF3
40kd
IB：ACTIN
100kd
70kd
IB：p-STAT1
40kd
IB：ACTIN
100kd
70kd
IB：STAT1
40kd
IB：ACTIN
70kd
55kd
40kd
35kd
25kd
15kd
IB：FLAG
40kd
IB：ACTIN

Figure 3M

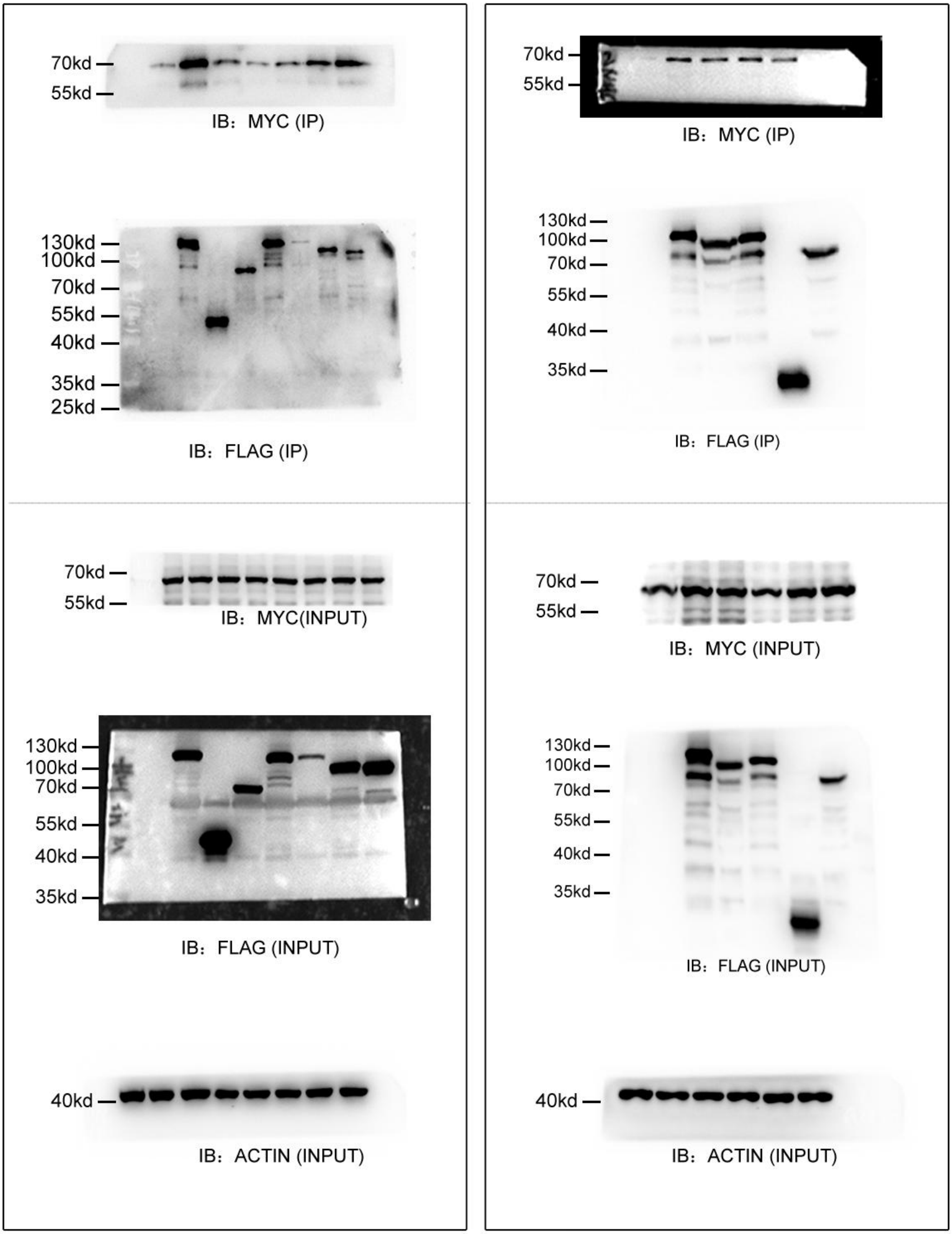
70kd
55kd
IB：MYC (IP)
130kd
100kd
70kd
55kd
40kd
35kd
25kd
IB：FLAG (IP)
70kd
55kd
IB：MYC(INPUT)
130kd
100kd
70kd
55kd
40kd
35kd
IB：FLAG (INPUT)
40kd
IB：ACTIN (INPUT)
70kd
55kd
IB：MYC (IP)
130kd
100kd
70kd
55kd
40kd
35kd
IB：FLAG (IP)
70kd
55kd
IB：MYC (INPUT)
130kd
100kd
70kd
55kd
40kd
35kd
IB：FLAG (INPUT)
40kd
IB：ACTIN (INPUT)

Figure 4A

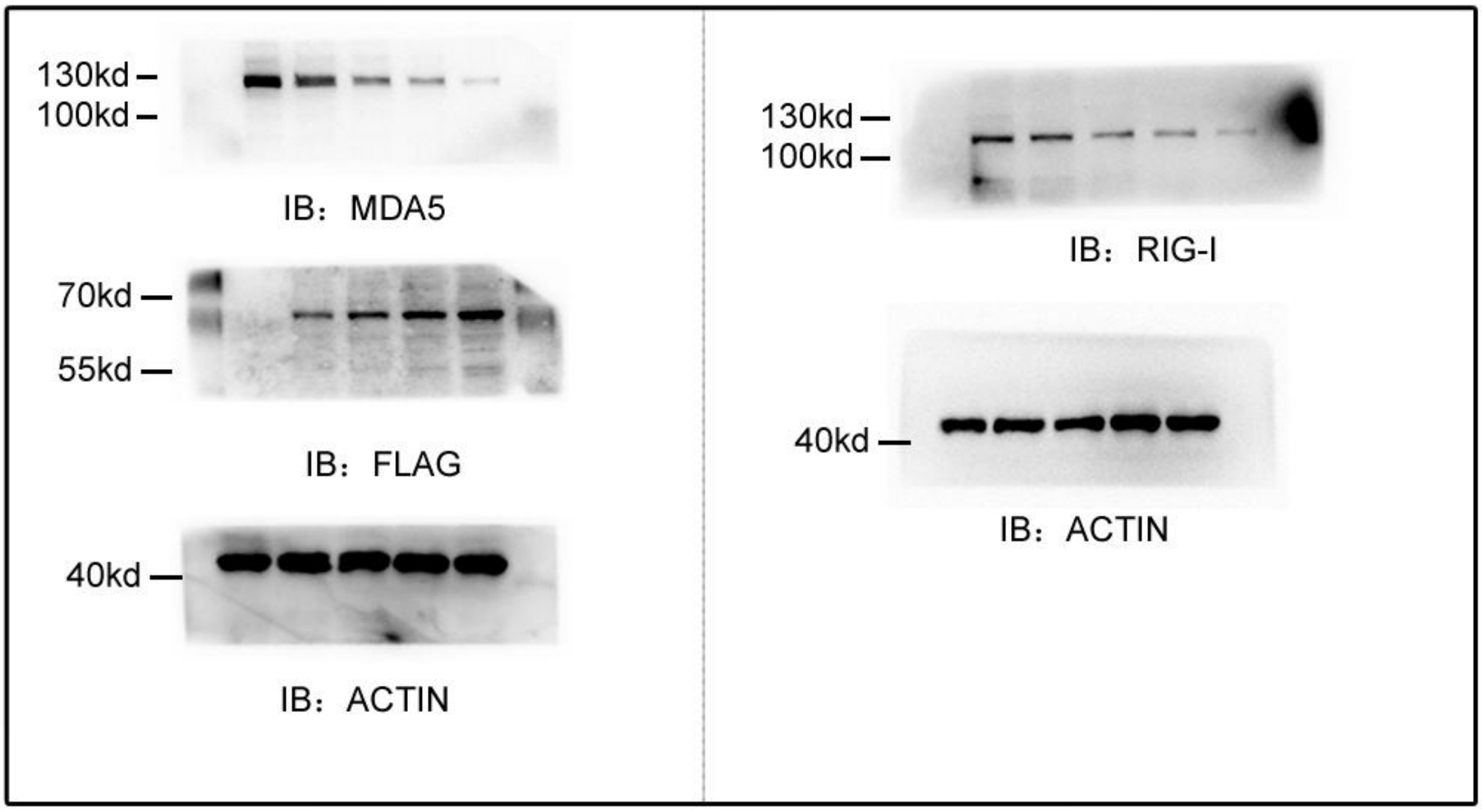

Figure 4B

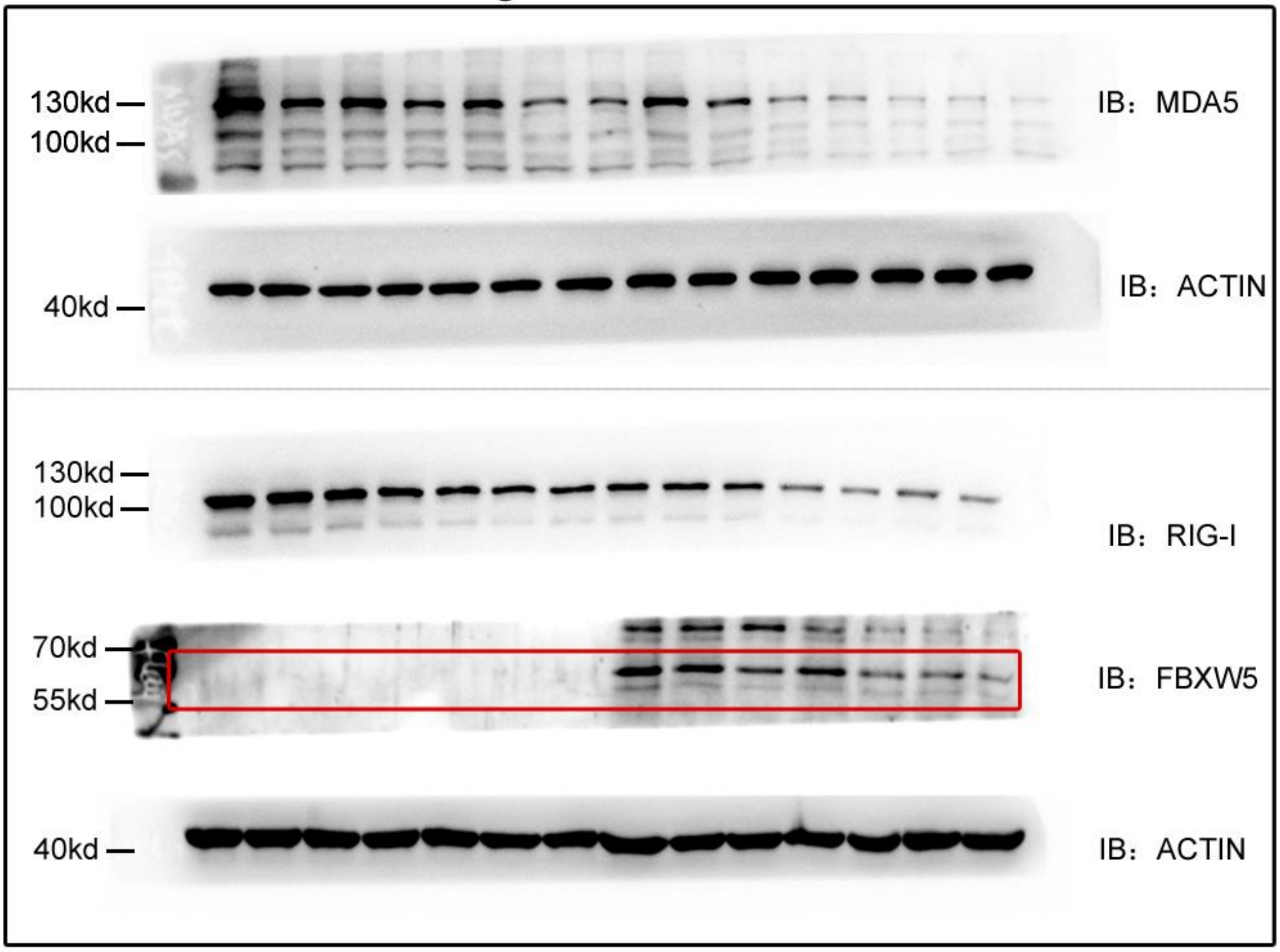

Figure 4D

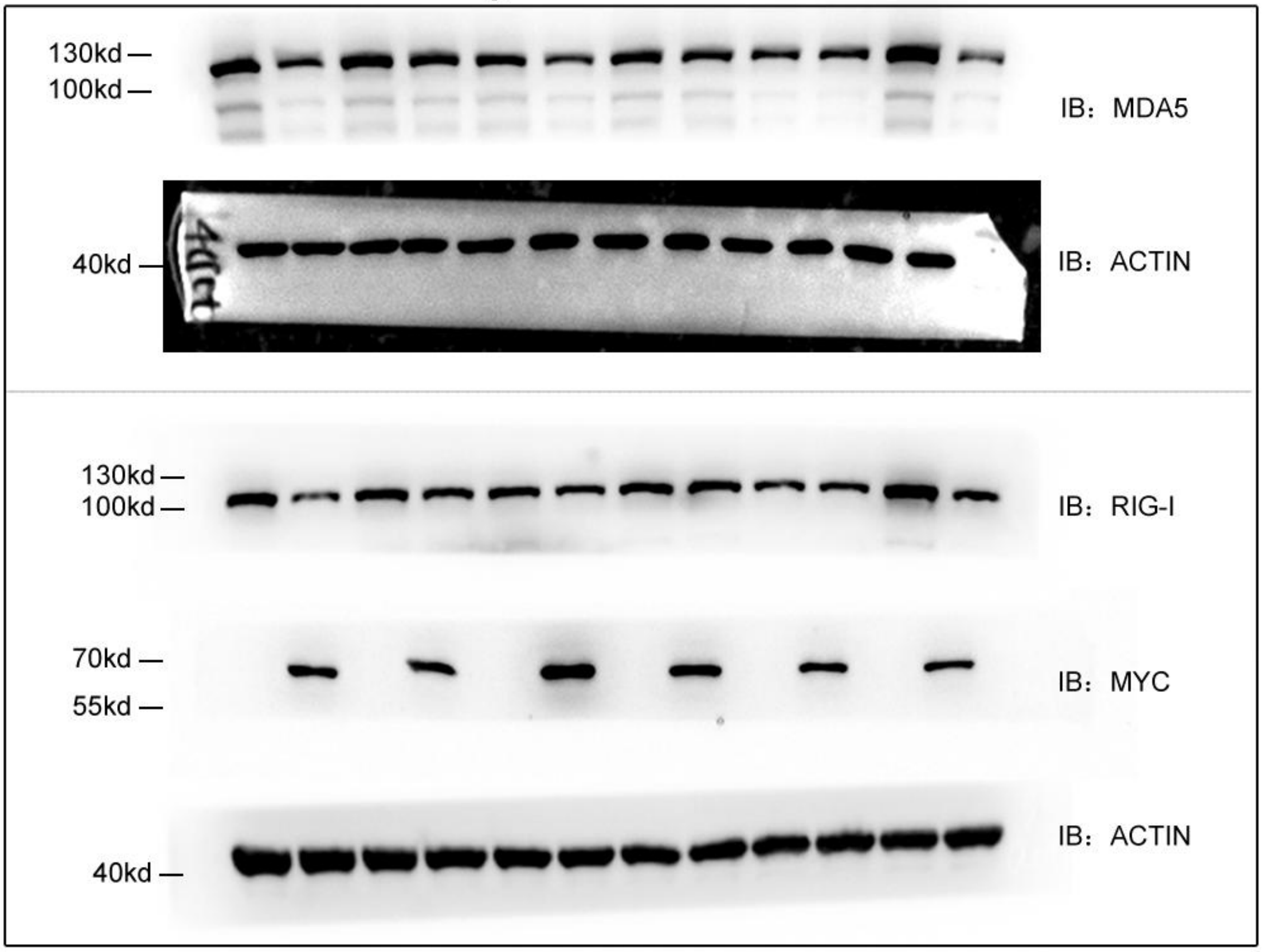

Figure 4E

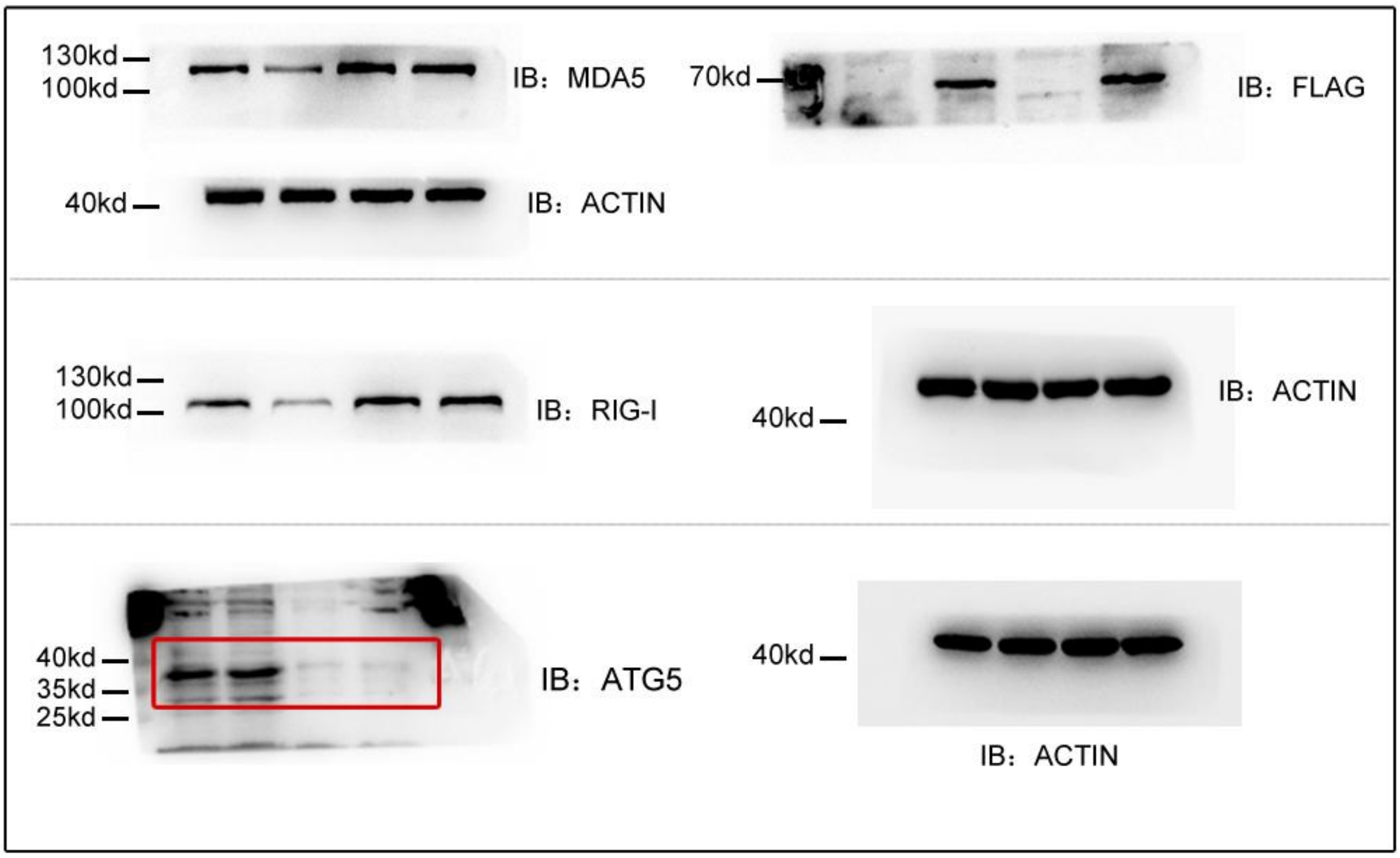

Figure 4F

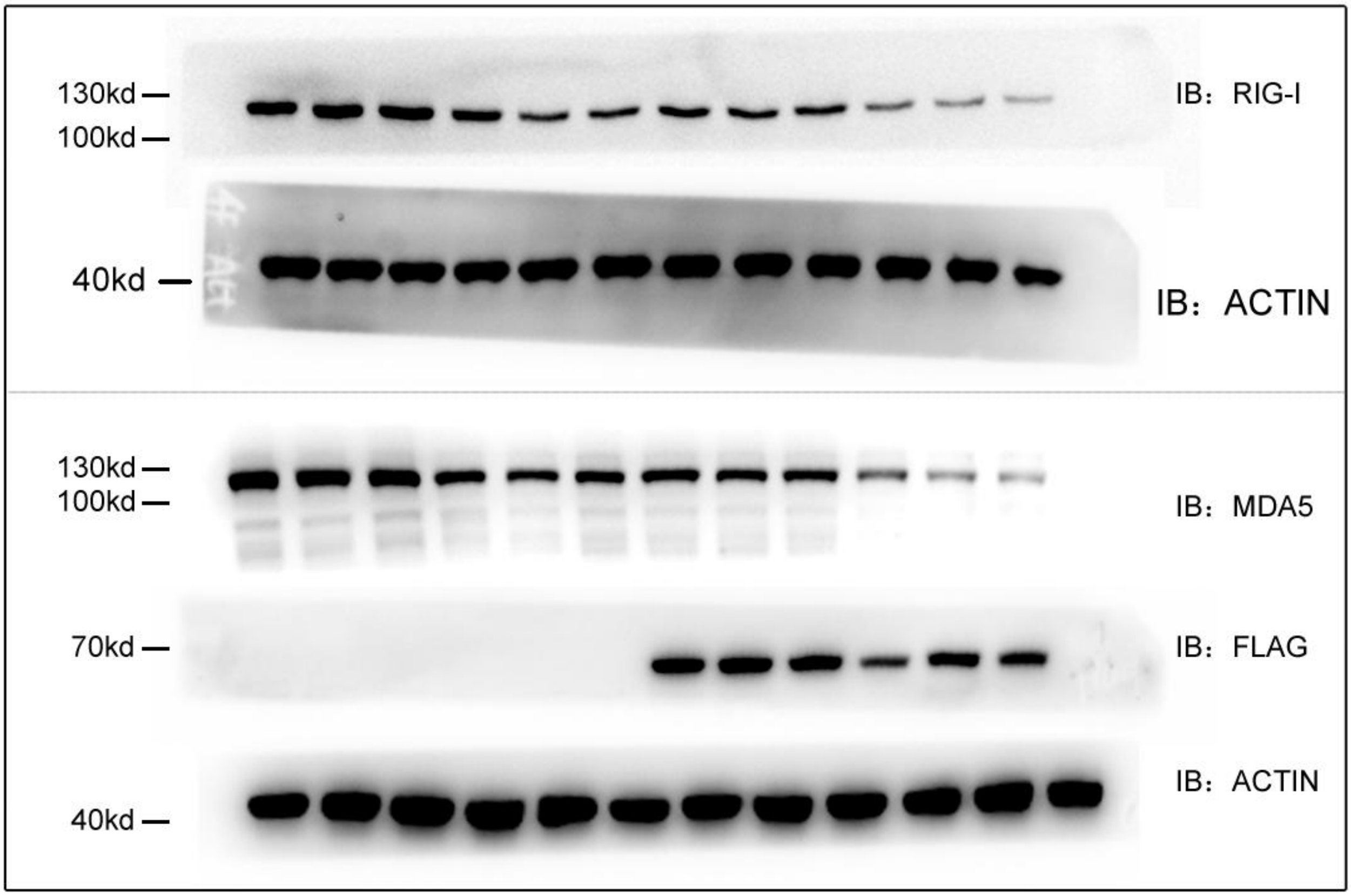

Figure 4G

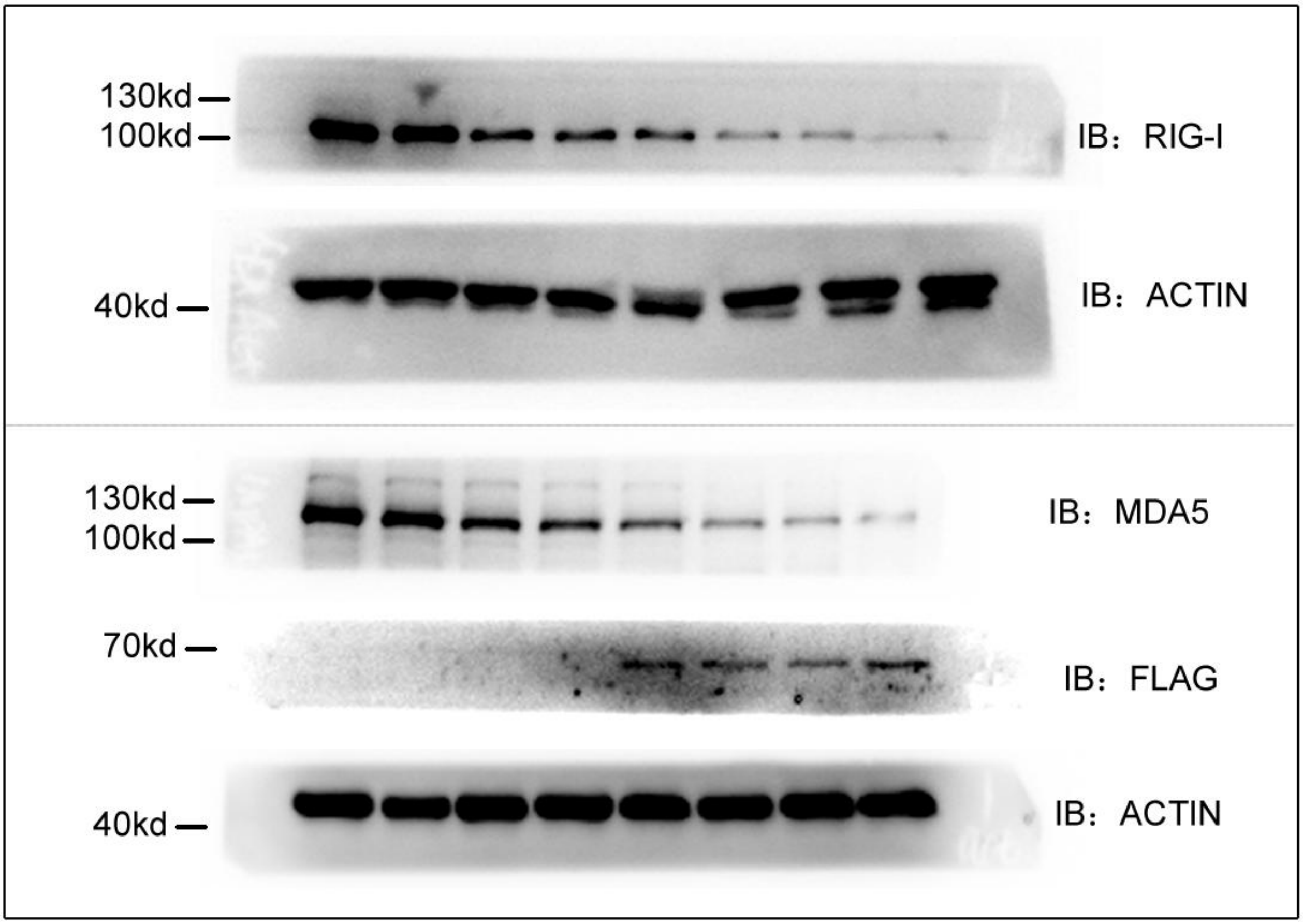

Figure 4H

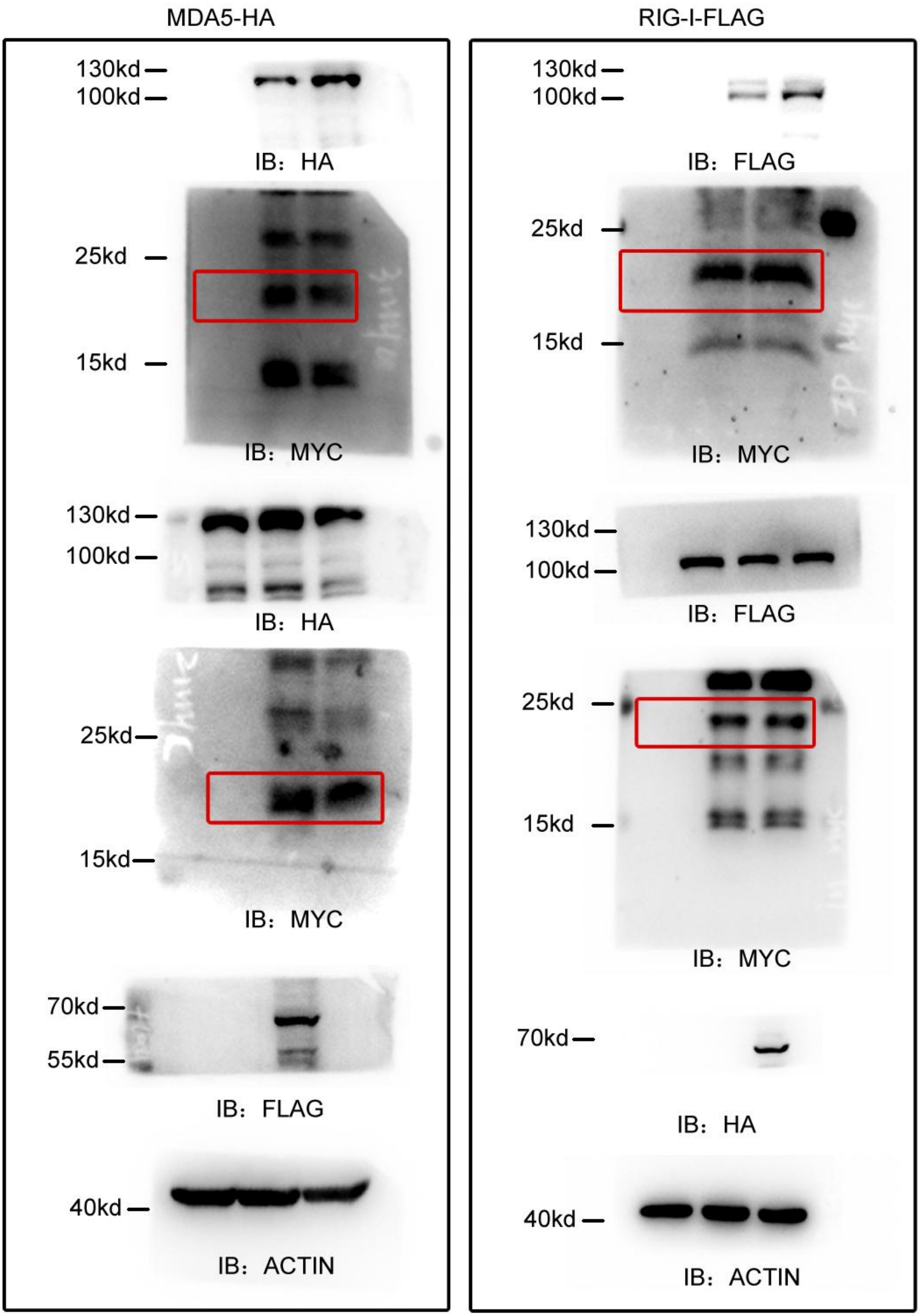
MDA5-HA
RIG-I-FLAG
130kd
100kd
IB：HA
25kd
15kd
IB：MYC
130kd
100kd
IB：HA
25kd
15kd
IB：MYC
70kd
55kd
IB：FLAG
40kd
IB：ACTIN
130kd
100kd
IB：FLAG
25kd
15kd
IB：MYC
130kd
100kd
IB：FLAG
25kd
15kd
IB：MYC
70kd
IB：HA
40kd
IB：ACTIN

Figure 5A

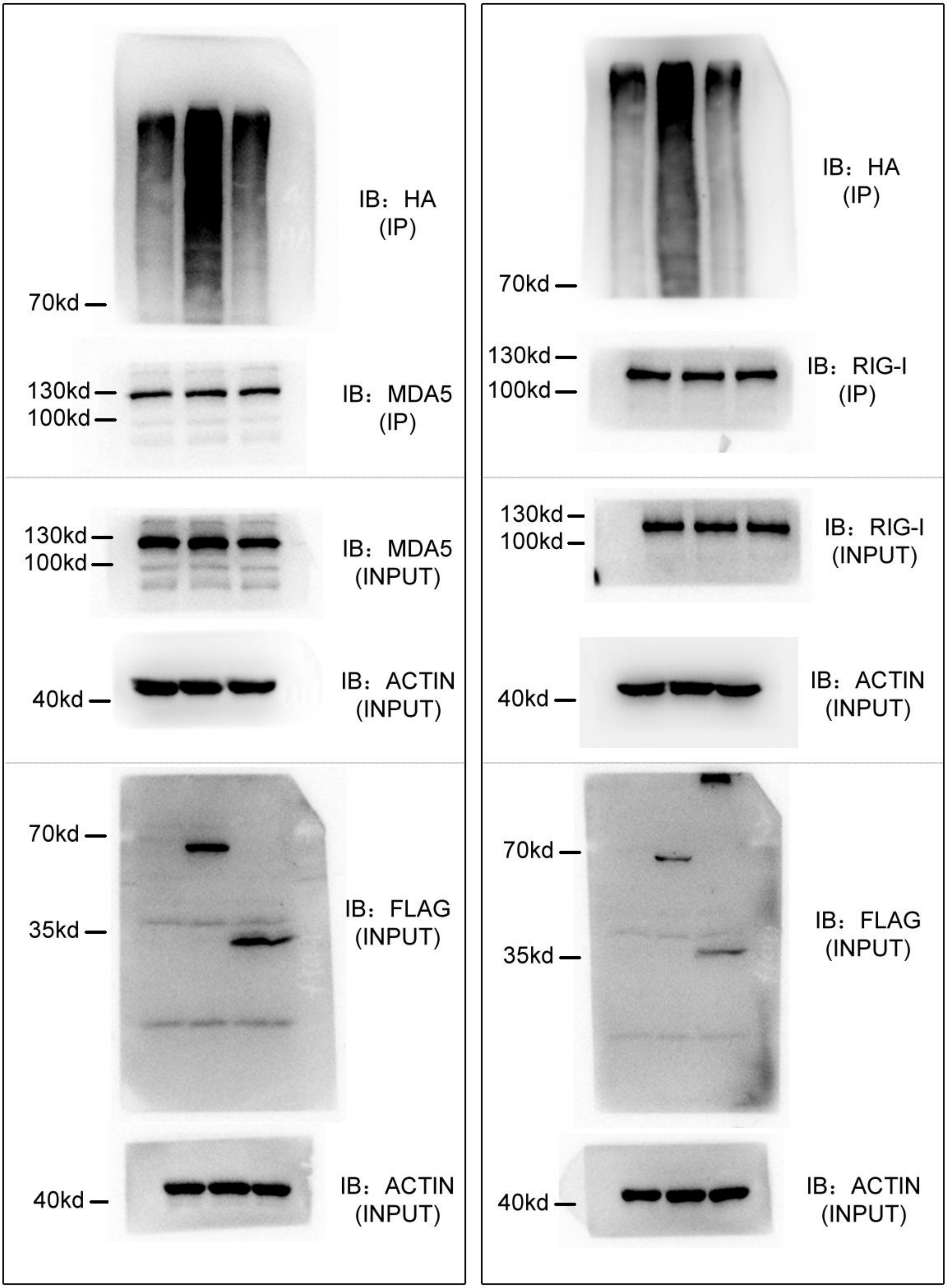
IB：HA
(IP)
70kd
IB：MDA5
(IP)
130kd
100kd
IB：MDA5
(INPUT)
130kd
100kd
IB：ACTIN
(INPUT)
40kd
IB：FLAG
(INPUT)
70kd
35kd
IB：ACTIN
(INPUT)
40kd
IB：HA
(IP)
70kd
IB：RIG-I
(IP)
130kd
100kd
IB：RIG-I
(INPUT)
130kd
100kd
IB：ACTIN
(INPUT)
40kd
IB：FLAG
(INPUT)
70kd
35kd
IB：ACTIN
(INPUT)
40kd

Figure 5B

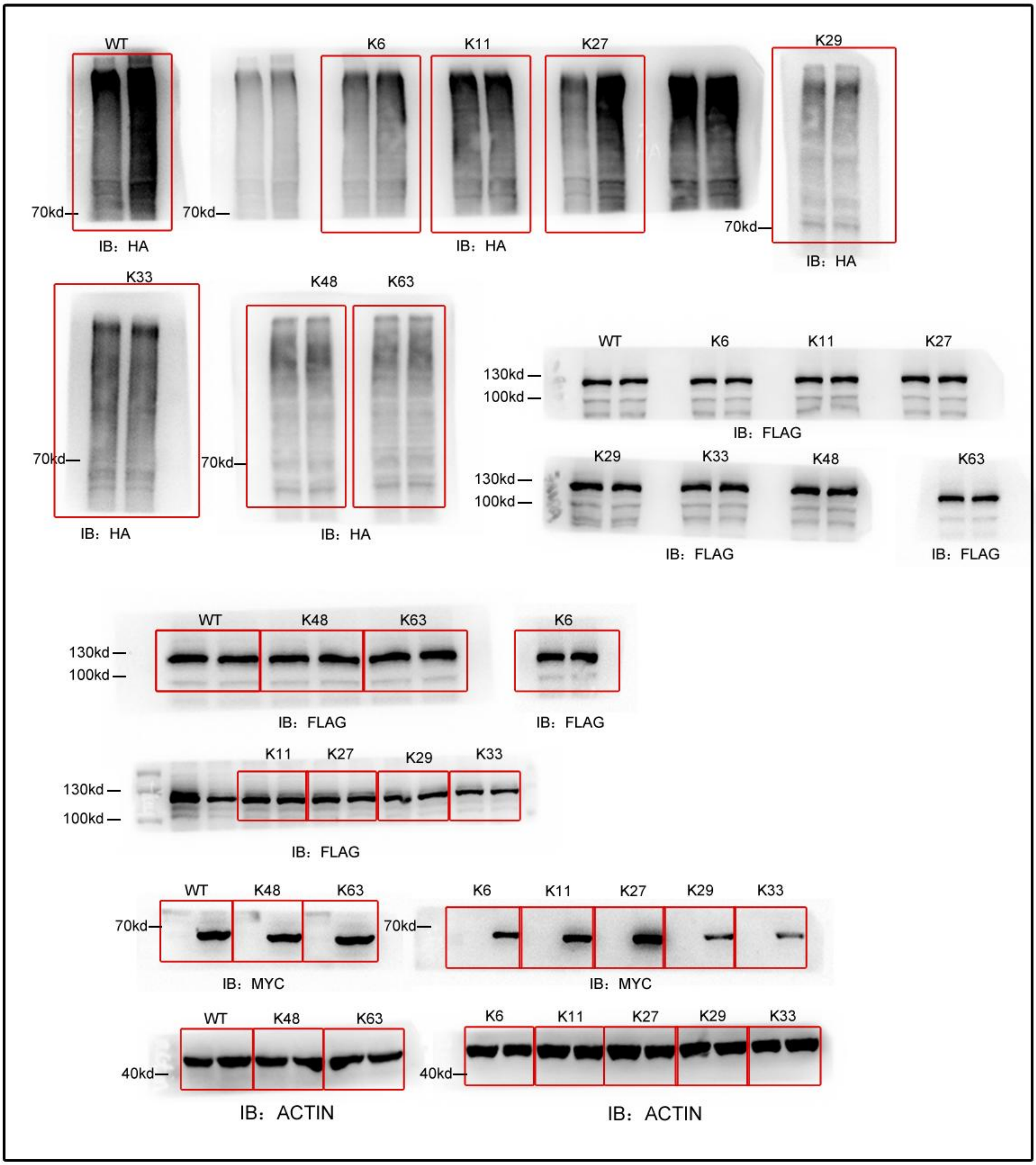

WT
K6
K11
K27
K29
70kd
IB：HA
K33
K48
K63
WT
K6
K11
K27
130kd
100kd
IB：FLAG
K29
K33
K48
K63
IB：FLAG
WT
K48
K63
K6
IB：FLAG
K11
K27
K29
K33
IB：FLAG
IB：MYC
40kd
IB：ACTIN

Figure 5C

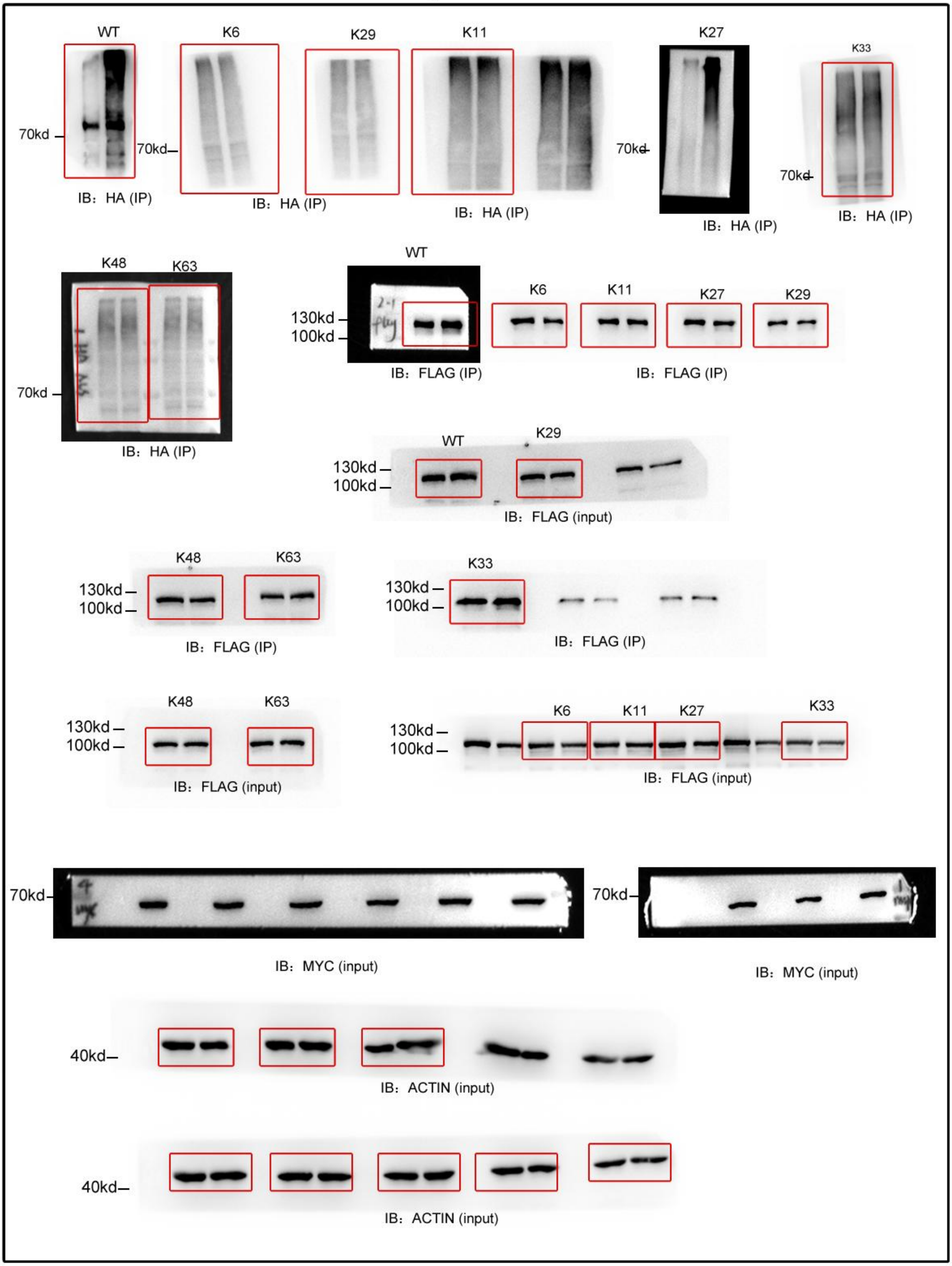

WT
K6
K29
K11
K27
K33
70kd
IB：HA (IP)
K48
K63
WT
K6
K11
K27
K29
130kd
100kd
IB：FLAG (IP)
WT
K29
IB：FLAG (input)
K48
K63
K33
IB：FLAG (IP)
K48
K63
K6
K11
K27
K33
IB：FLAG (input)
70kd
IB：MYC (input)
40kd
IB：ACTIN (input)

Figure 5D

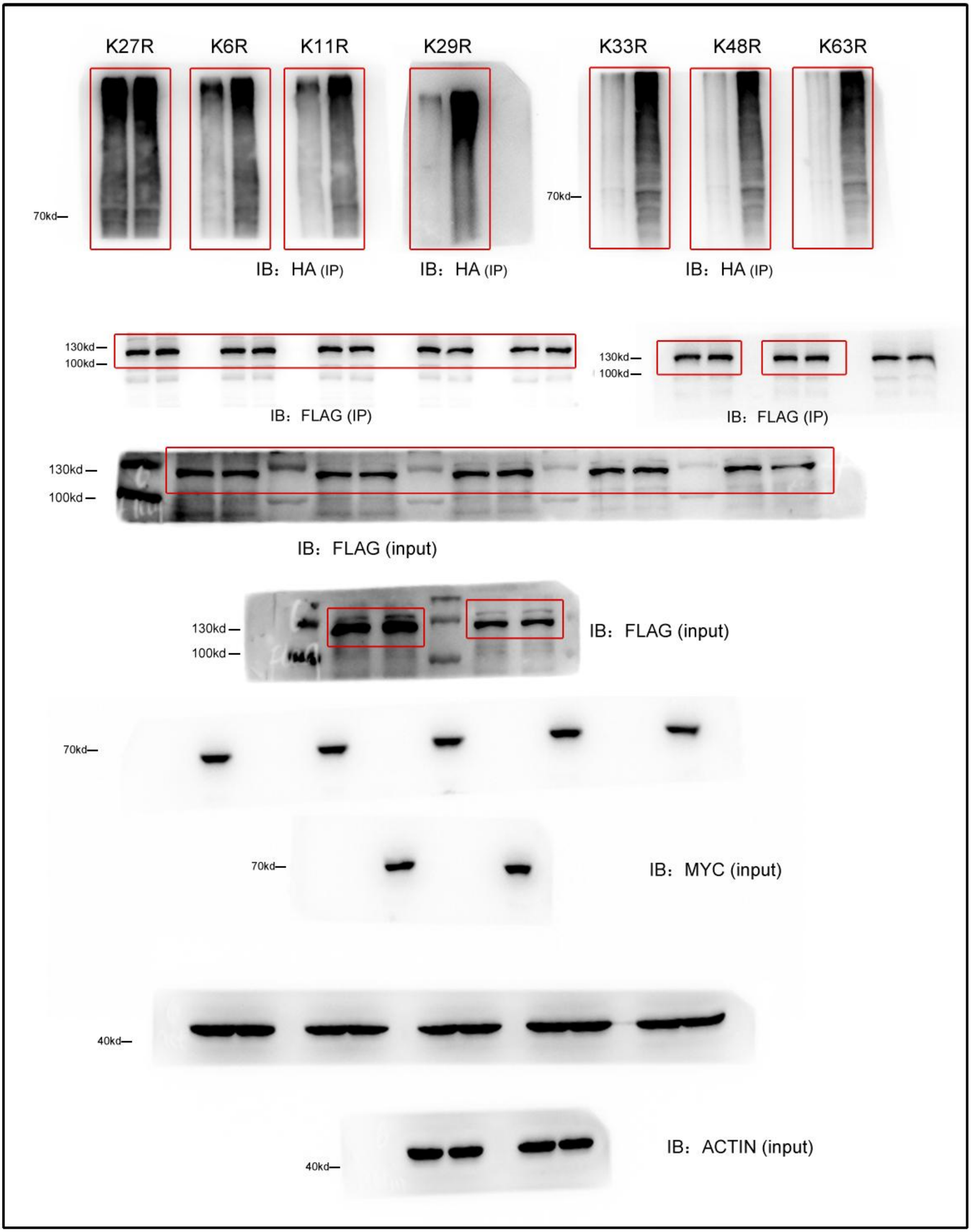
K27R
K6R
K11R
K29R
K33R
K48R
K63R
70kd
70kd
IB：HA (IP)
IB：HA (IP)
IB：HA (IP)
130kd
100kd
130kd
100kd
IB：FLAG (IP)
IB：FLAG (IP)
130kd
100kd
IB：FLAG (input)
130kd
100kd
IB：FLAG (input)
70kd
70kd
IB：MYC (input)
40kd
40kd
IB：ACTIN (input)

Figure 5E

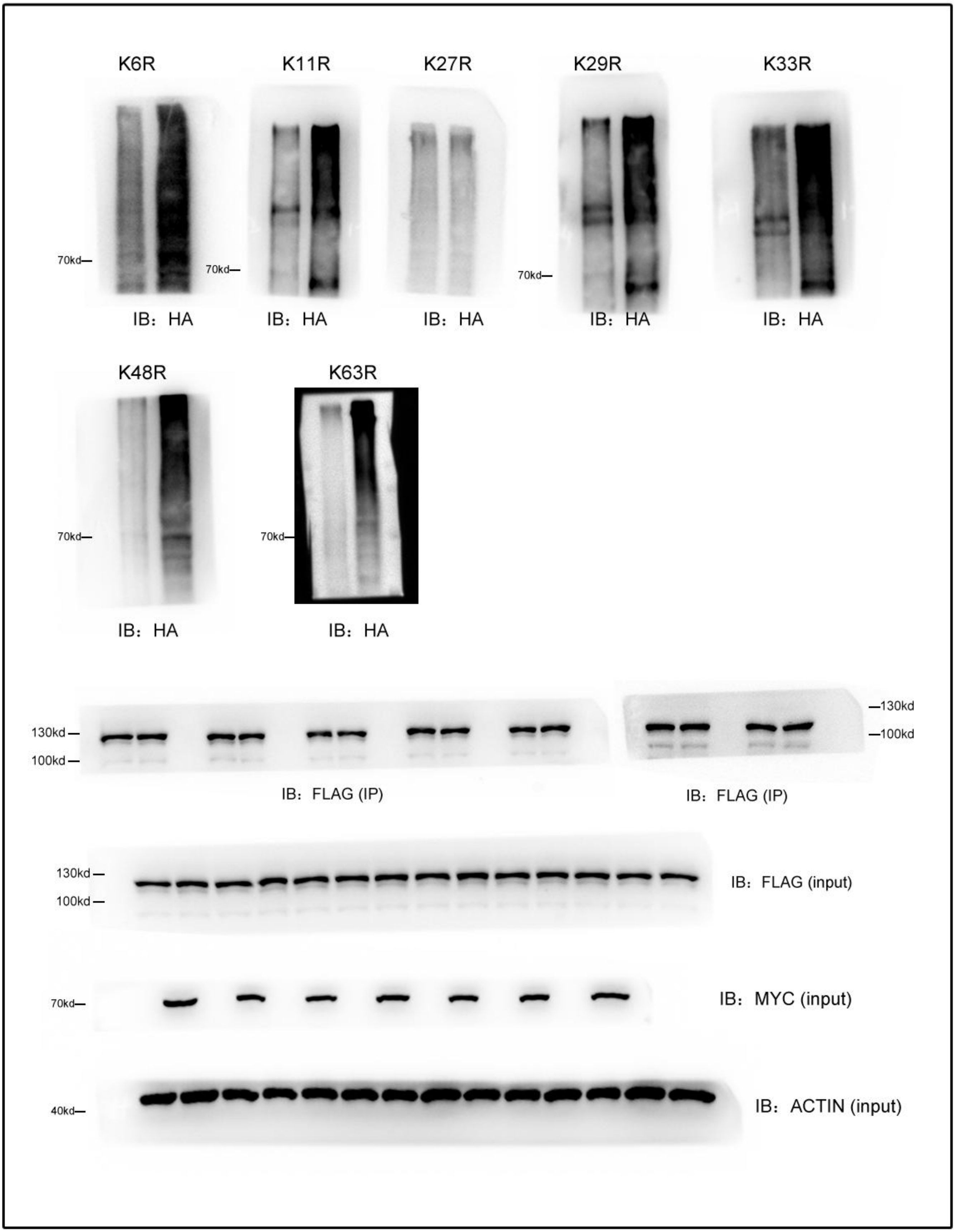
K6R
K11R
K27R
K29R
K33R
70kd
IB：HA
K48R
K63R
130kd
100kd
IB：FLAG (IP)
IB：FLAG (input)
IB：MYC (input)
IB：ACTIN (input)
40kd

Figure 5G

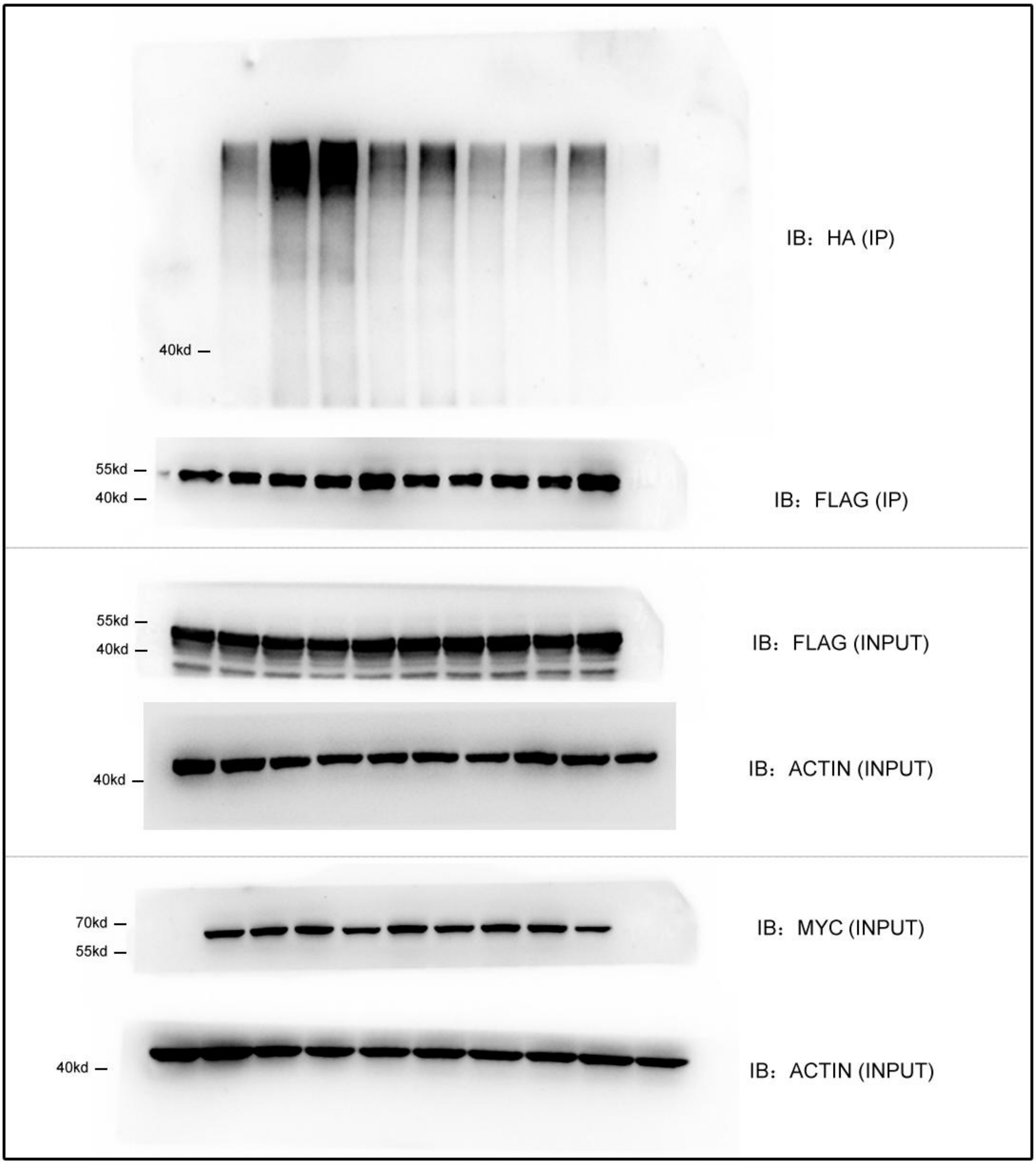
40kd
IB：HA (IP)
55kd
40kd
IB：FLAG (IP)
55kd
40kd
IB：FLAG (INPUT)
40kd
IB：ACTIN (INPUT)
70kd
55kd
IB：MYC (INPUT)
40kd
IB：ACTIN (INPUT)

Figure 5H

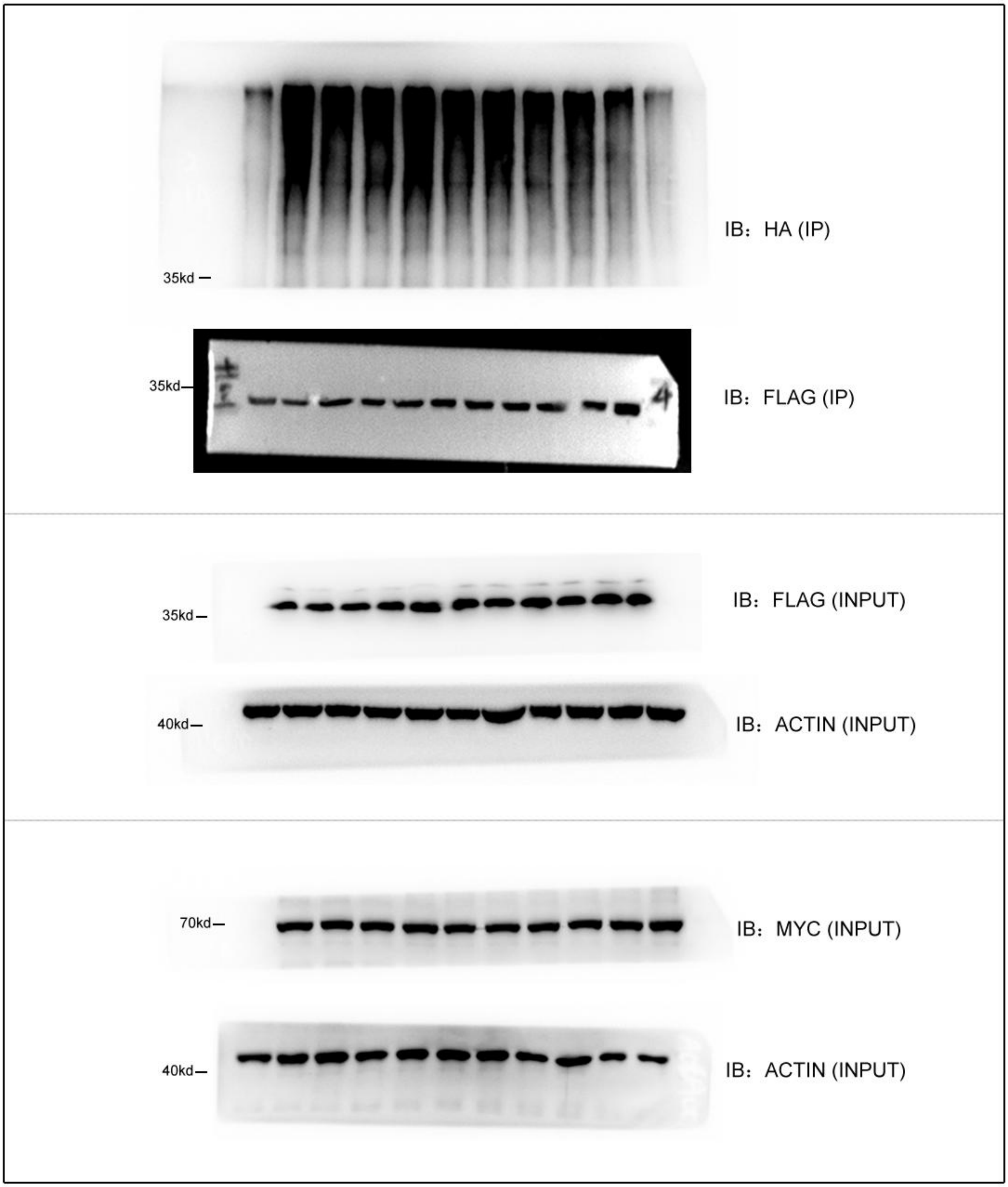
35kd
IB：HA (IP)
35kd
IB：FLAG (IP)
35kd
IB：FLAG (INPUT)
40kd
IB：ACTIN (INPUT)
70kd
IB：MYC (INPUT)
40kd
IB：ACTIN (INPUT)

Figure 5K

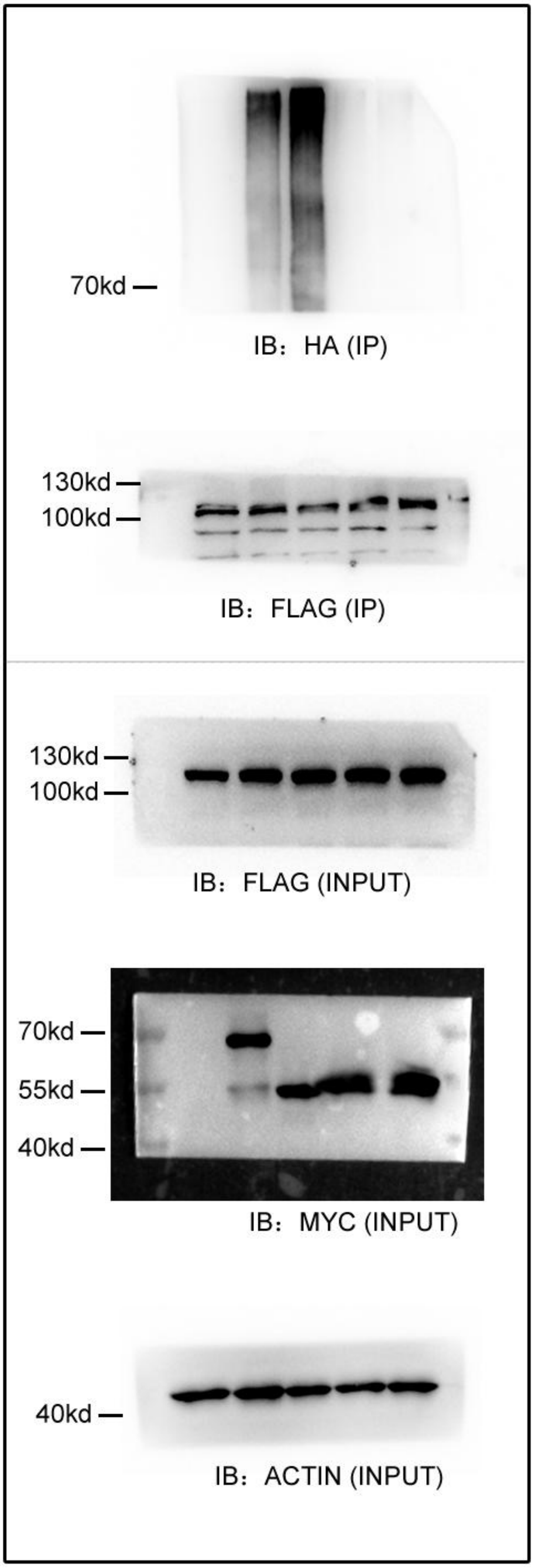

Figure 5L

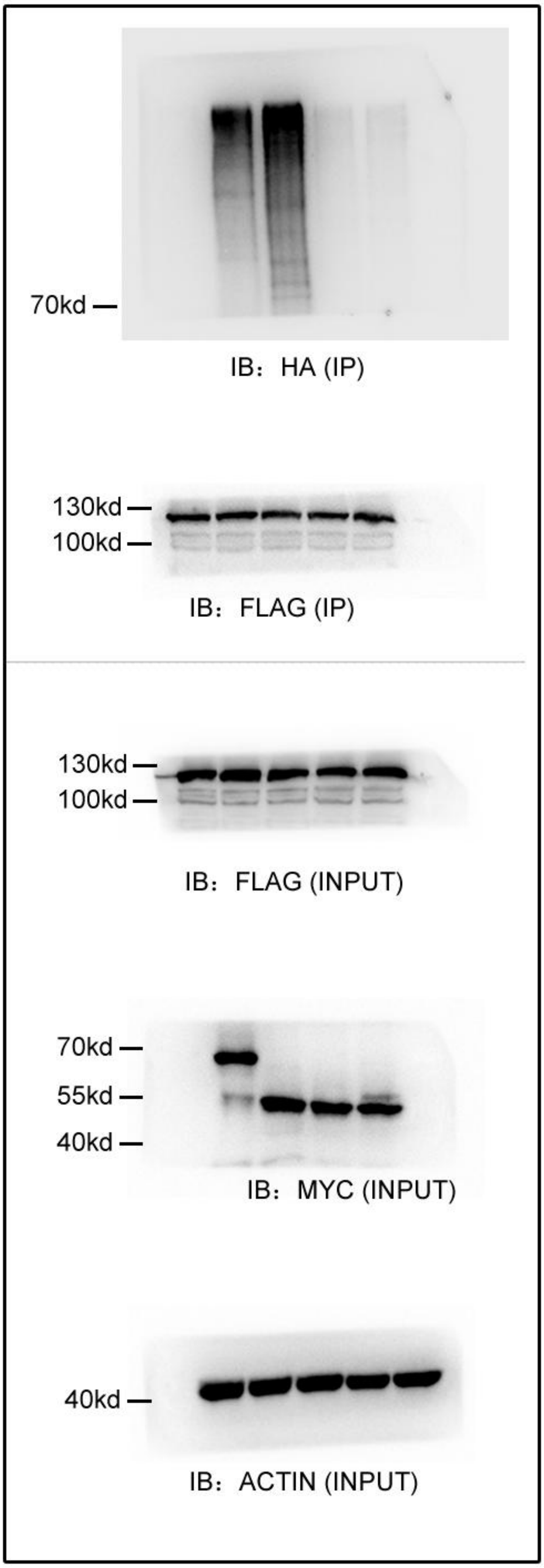

Figure 5J

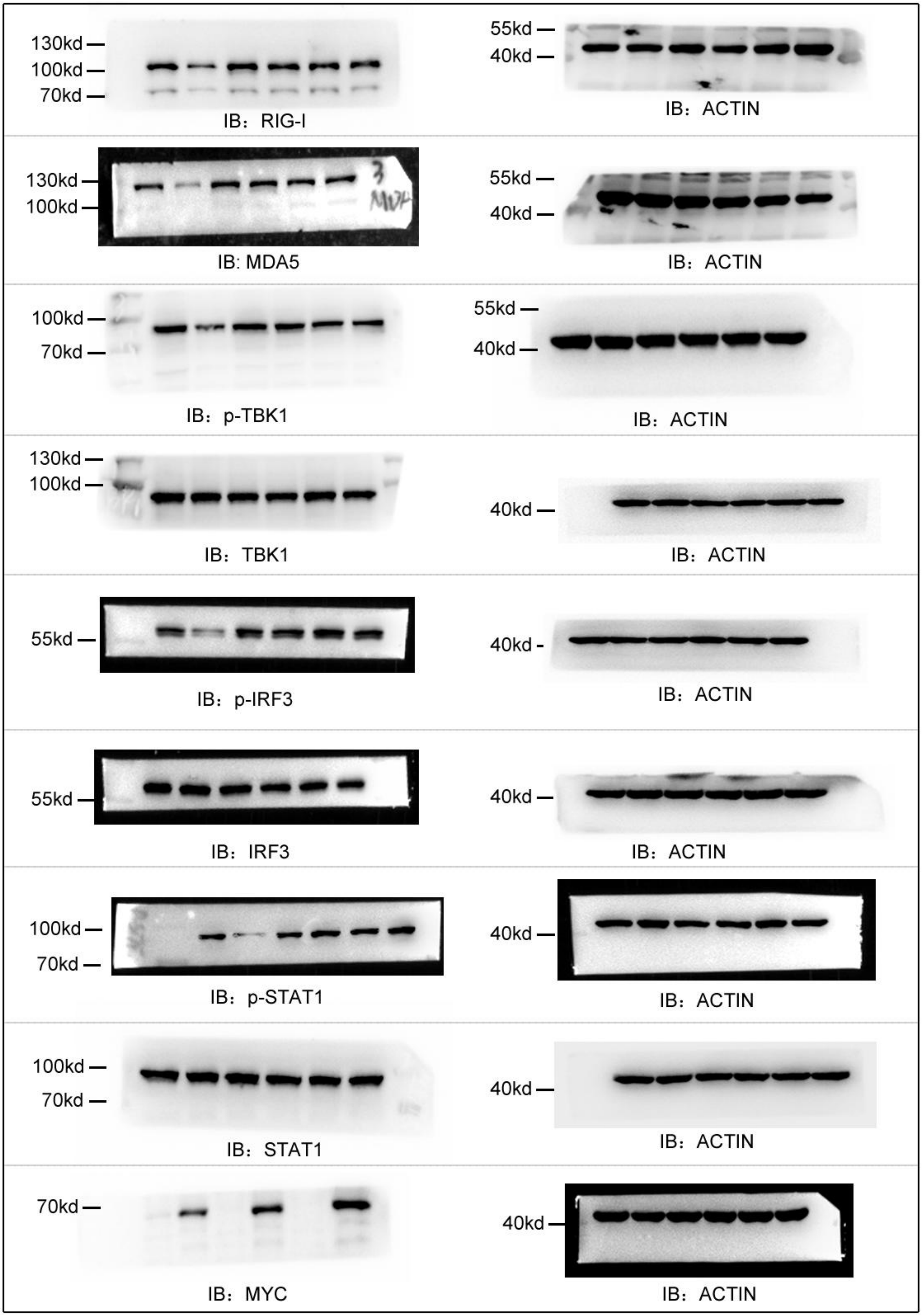

130kd
100kd
70kd
IB：RIG-I
55kd
40kd
IB：ACTIN
130kd
100kd
IB: MDA5
55kd
40kd
IB：ACTIN
100kd
70kd
IB：p-TBK1
55kd
40kd
IB：ACTIN
130kd
100kd
IB：TBK1
40kd
IB：ACTIN
55kd
IB：p-IRF3
40kd
IB：ACTIN
55kd
IB：IRF3
40kd
IB：ACTIN
100kd
70kd
IB：p-STAT1
40kd
IB：ACTIN
100kd
70kd
IB：STAT1
40kd
IB：ACTIN
70kd
IB：MYC
40kd
IB：ACTIN

Figure 5M

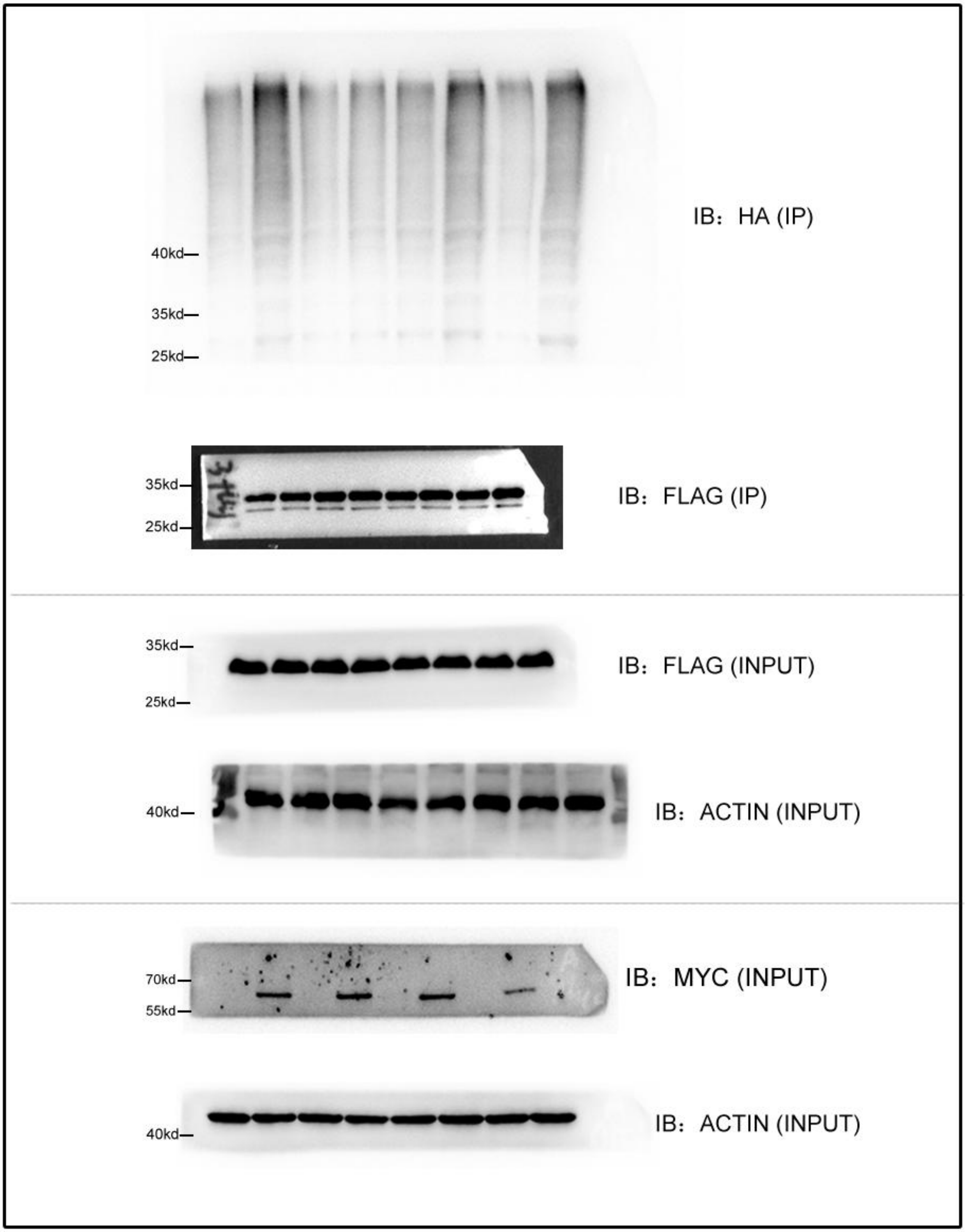
40kd
35kd
25kd
IB：HA (IP)
35kd
25kd
IB：FLAG (IP)
35kd
25kd
IB：FLAG (INPUT)
40kd
IB：ACTIN (INPUT)
70kd
55kd
IB：MYC (INPUT)
40kd
IB：ACTIN (INPUT)

Figure 5N

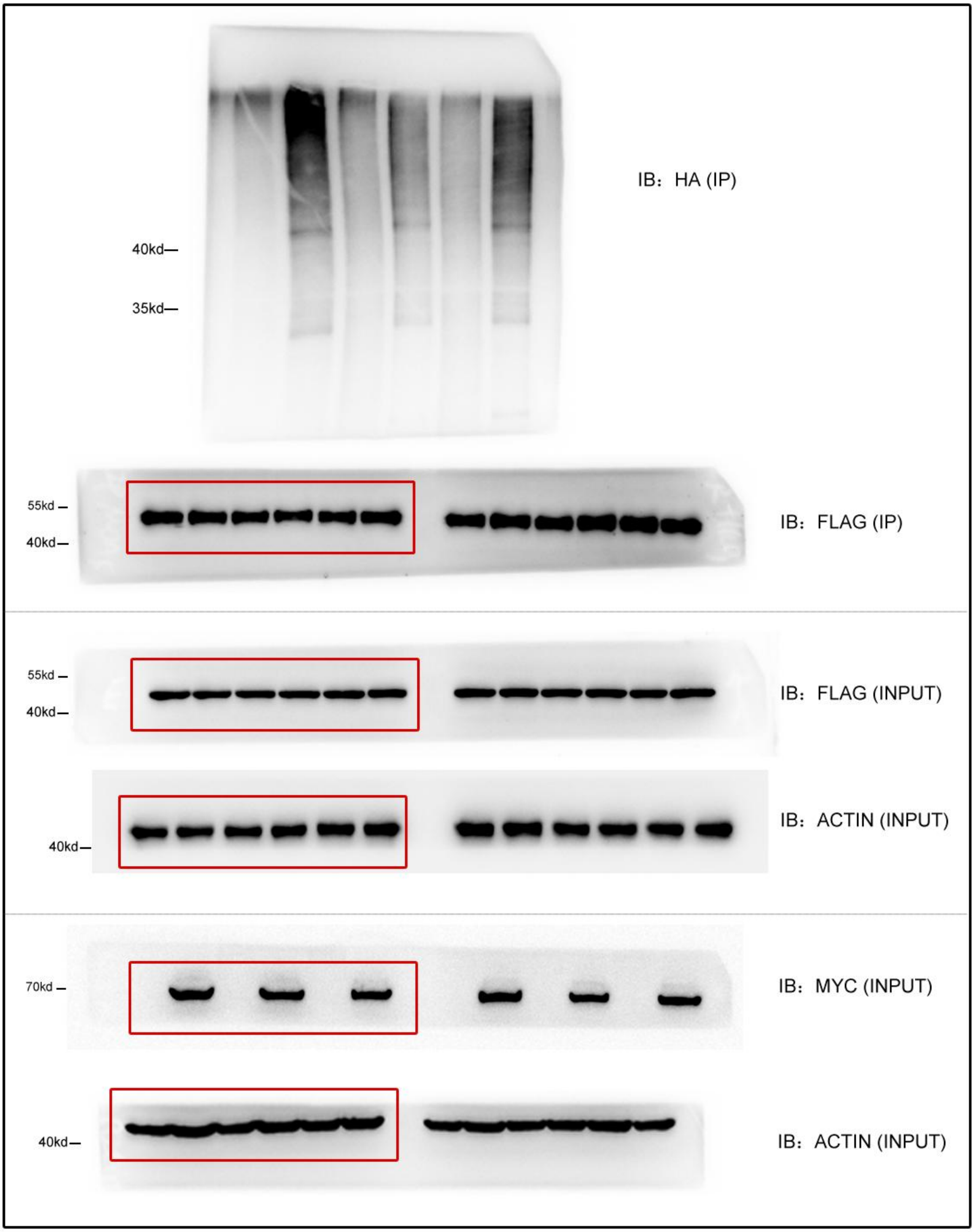

IB：HA (IP)
40kd
35kd
55kd
40kd
IB：FLAG (IP)
55kd
40kd
IB：FLAG (INPUT)
40kd
IB：ACTIN (INPUT)
70kd
IB：MYC (INPUT)
40kd
IB：ACTIN (INPUT)

Figure 6A

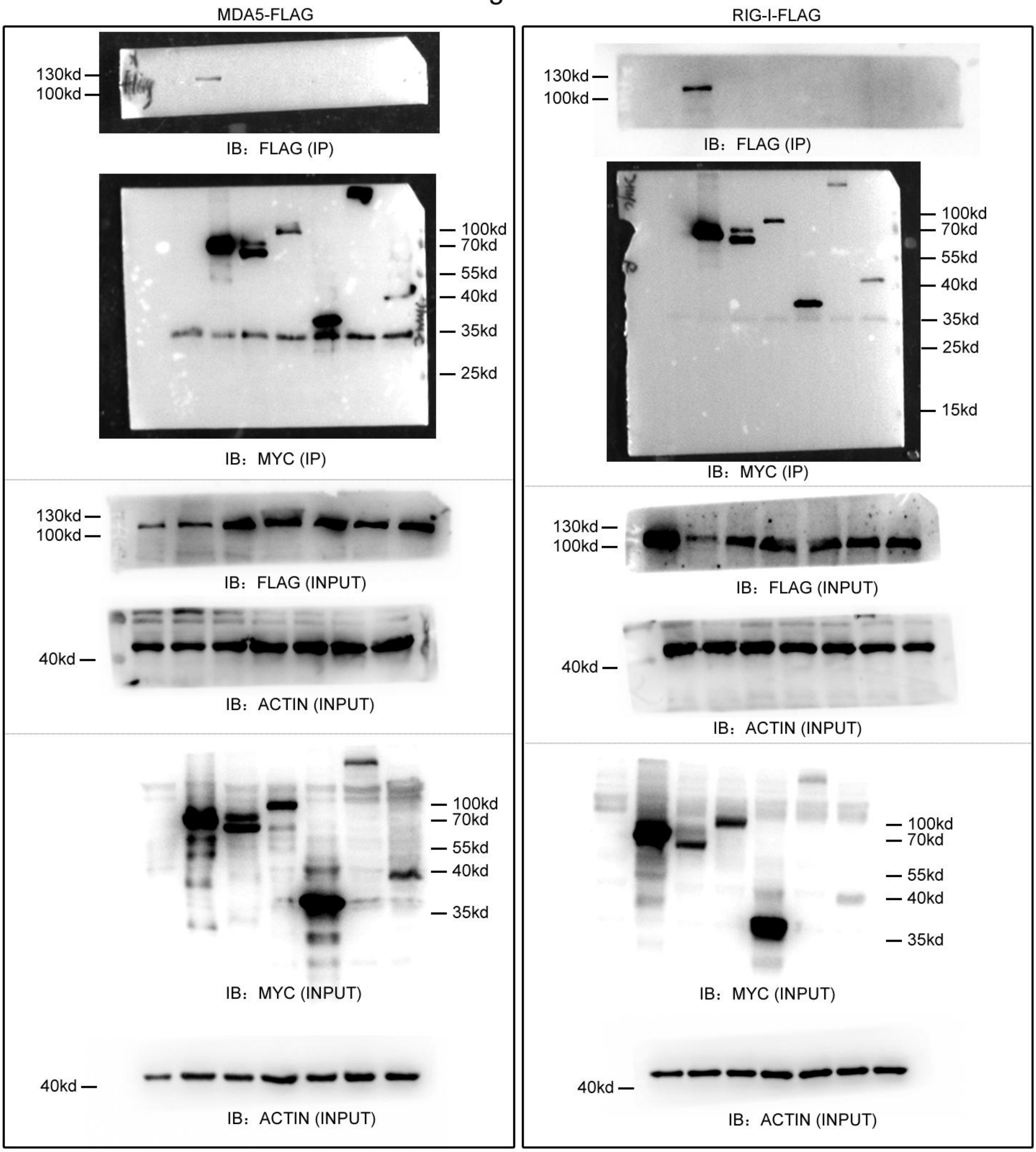

MDA5-FLAG
RIG-I-FLAG
130kd
100kd
IB：FLAG (IP)
100kd
70kd
55kd
40kd
35kd
25kd
15kd
IB：MYC (IP)
IB：FLAG (INPUT)
IB：ACTIN (INPUT)
IB：MYC (INPUT)
IB：ACTIN (INPUT)

Figure 6B

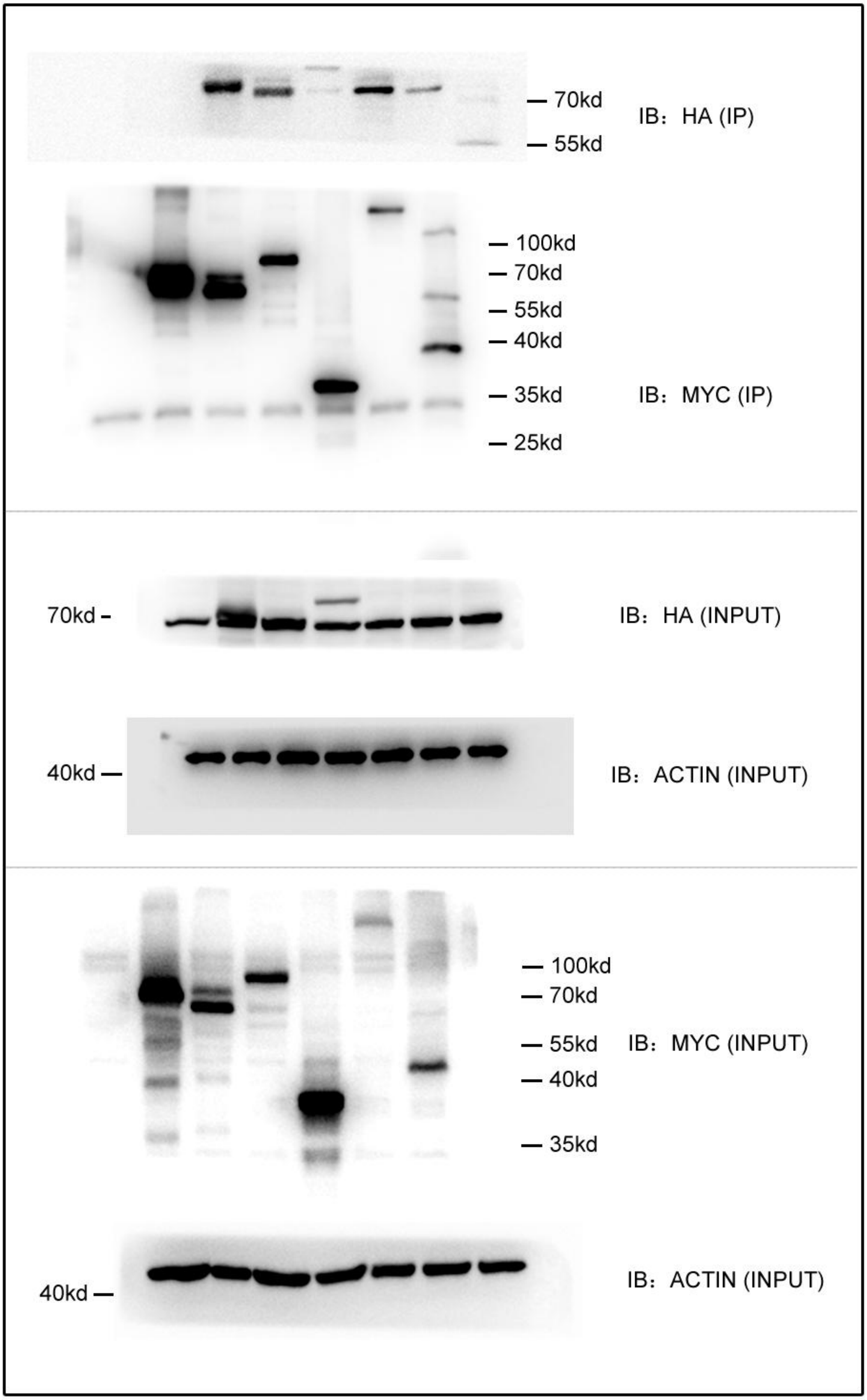
70kd
55kd
IB：HA (IP)
100kd
70kd
55kd
40kd
35kd
25kd
IB：MYC (IP)
70kd
IB：HA (INPUT)
40kd
IB：ACTIN (INPUT)
100kd
70kd
55kd
40kd
35kd
IB：MYC (INPUT)
40kd
IB：ACTIN (INPUT)

Figure 6C

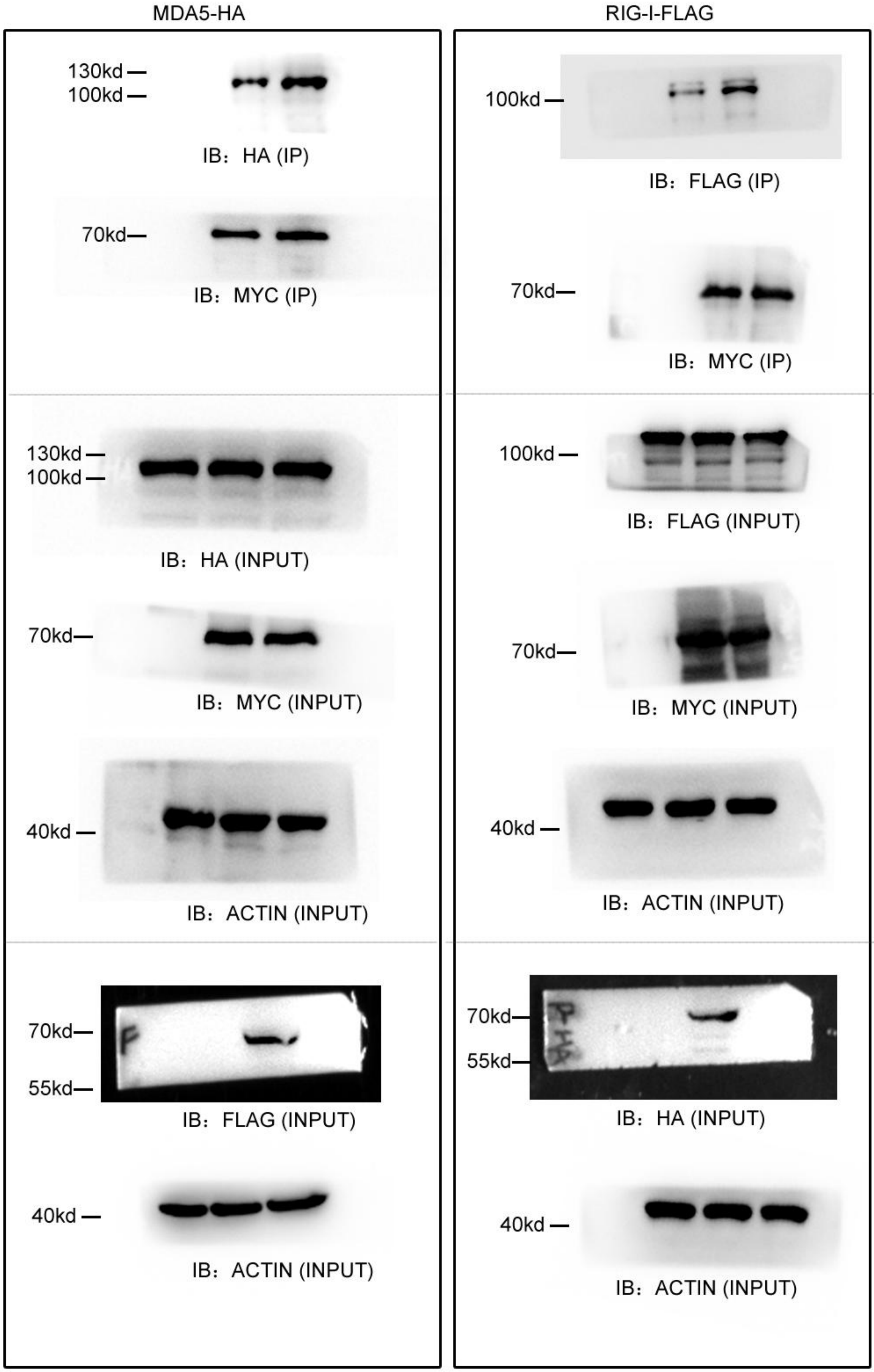
MDA5-HA
RIG-I-FLAG
130kd
100kd
IB：HA (IP)
70kd
IB：MYC (IP)
130kd
100kd
IB：HA (INPUT)
70kd
IB：MYC (INPUT)
40kd
IB：ACTIN (INPUT)
70kd
55kd
IB：FLAG (INPUT)
40kd
IB：ACTIN (INPUT)
100kd
IB：FLAG (IP)
70kd
IB：MYC (IP)
100kd
IB：FLAG (INPUT)
70kd
IB：MYC (INPUT)
40kd
IB：ACTIN (INPUT)
70kd
55kd
IB：HA (INPUT)
40kd
IB：ACTIN (INPUT)

Figure 6E

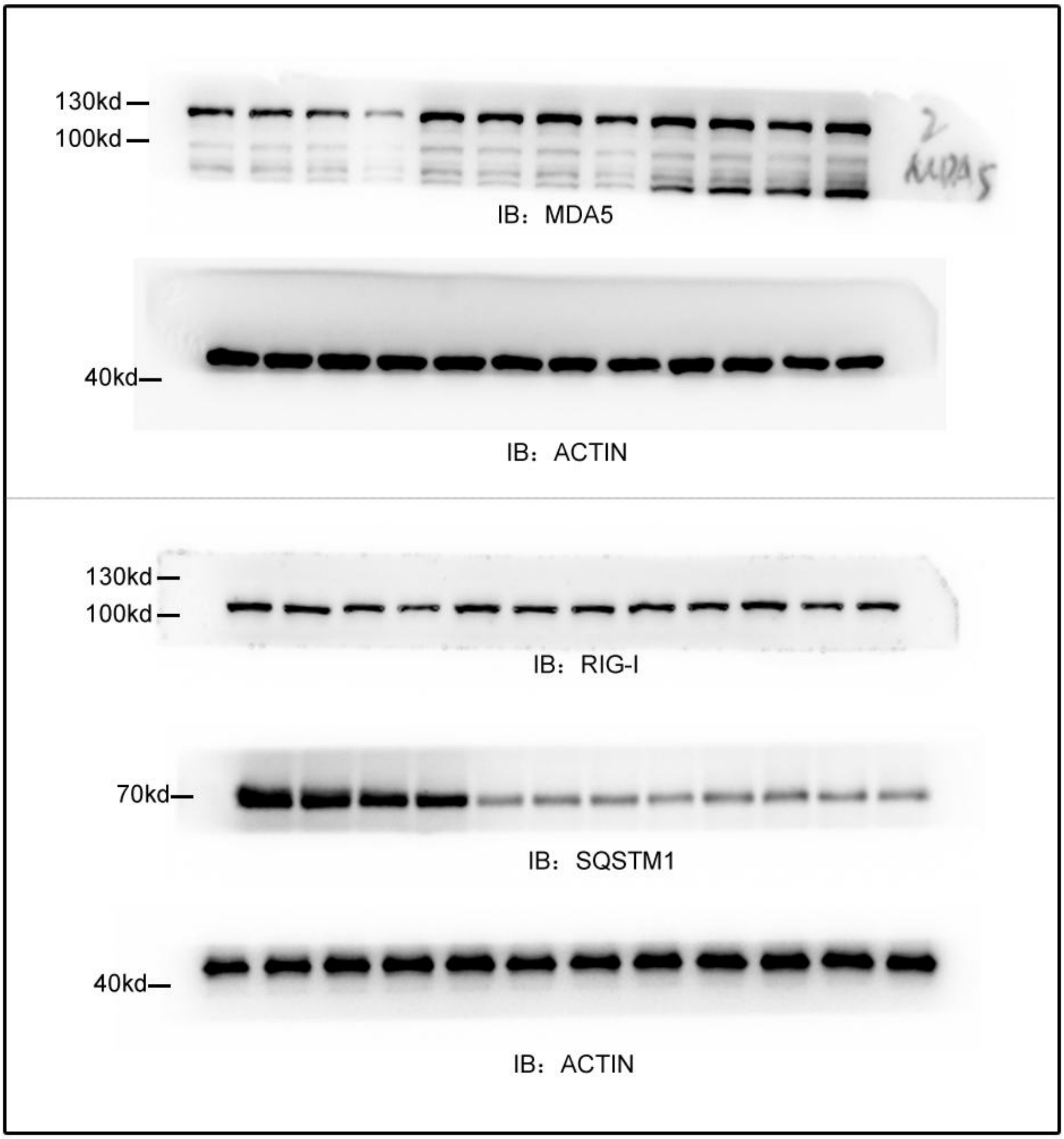

130kd
100kd
2
MDA5
IB：MDA5
40kd
IB：ACTIN
130kd
100kd
IB：RIG-I
70kd
IB：SQSTM1
40kd
IB：ACTIN

Figure 6F

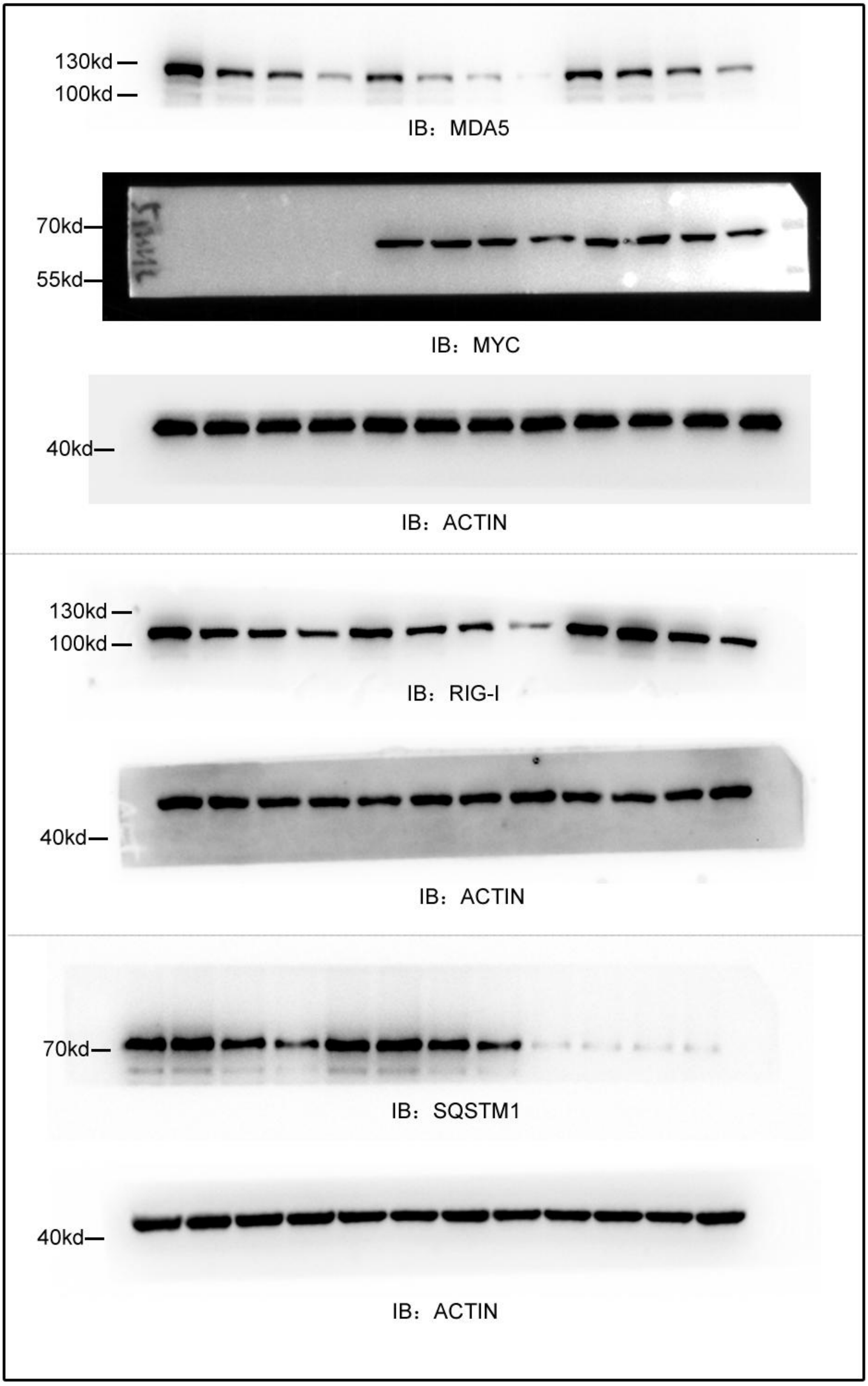
130kd
100kd
IB：MDA5
70kd
55kd
IB：MYC
40kd
IB：ACTIN
130kd
100kd
IB：RIG-I
40kd
IB：ACTIN
70kd
IB：SQSTM1
40kd
IB：ACTIN

Figure 6G

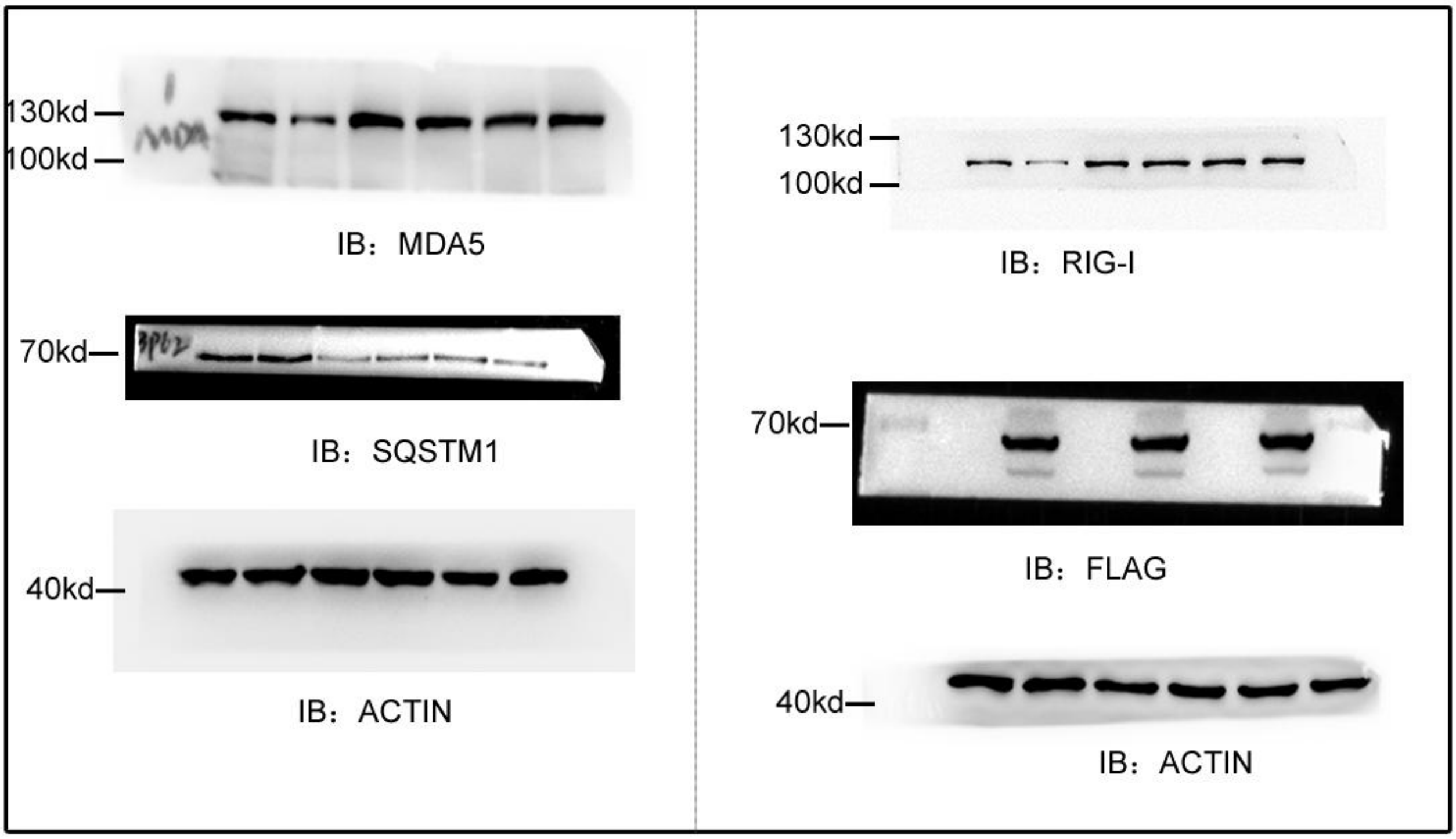

Figure 6J

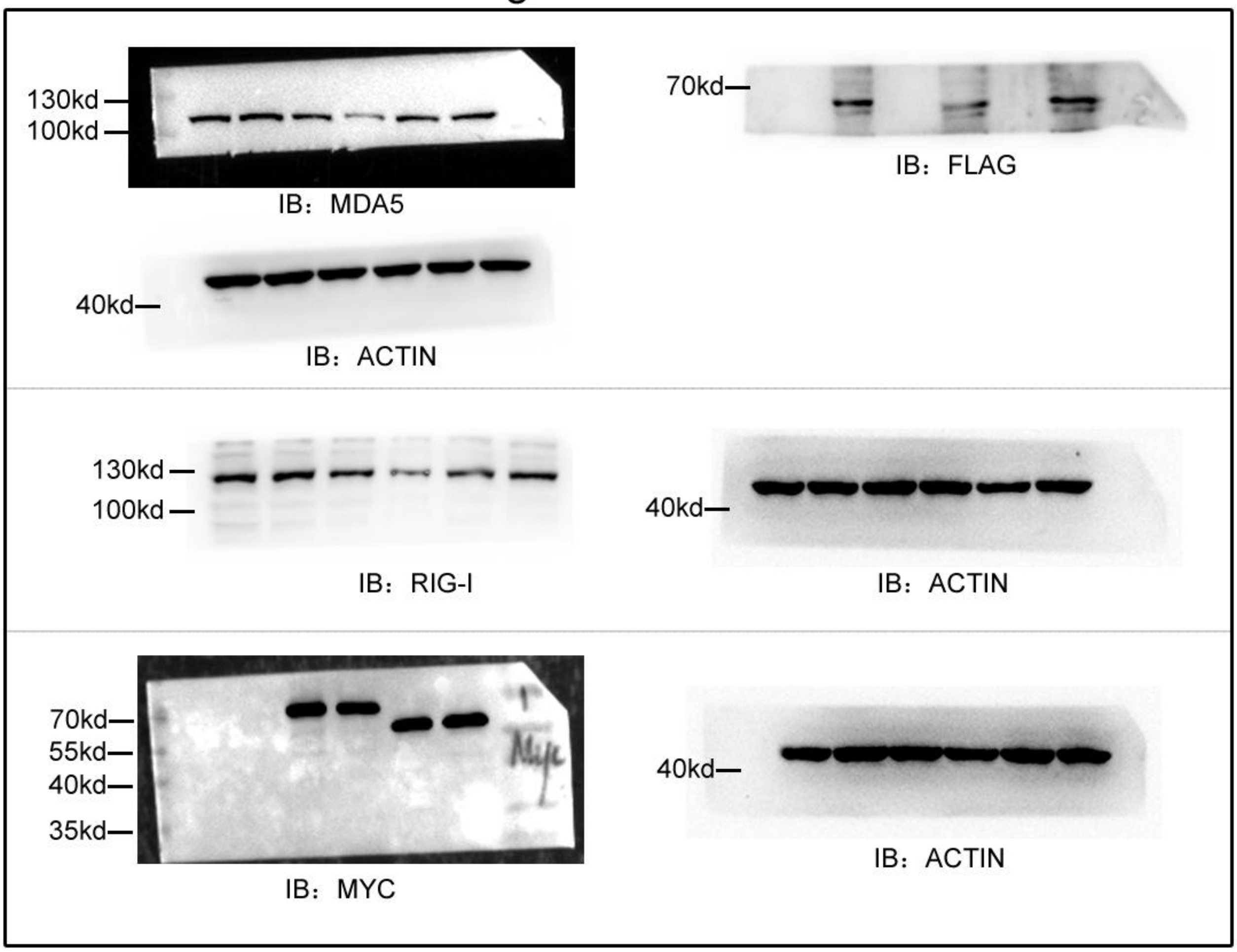

Figure 6I

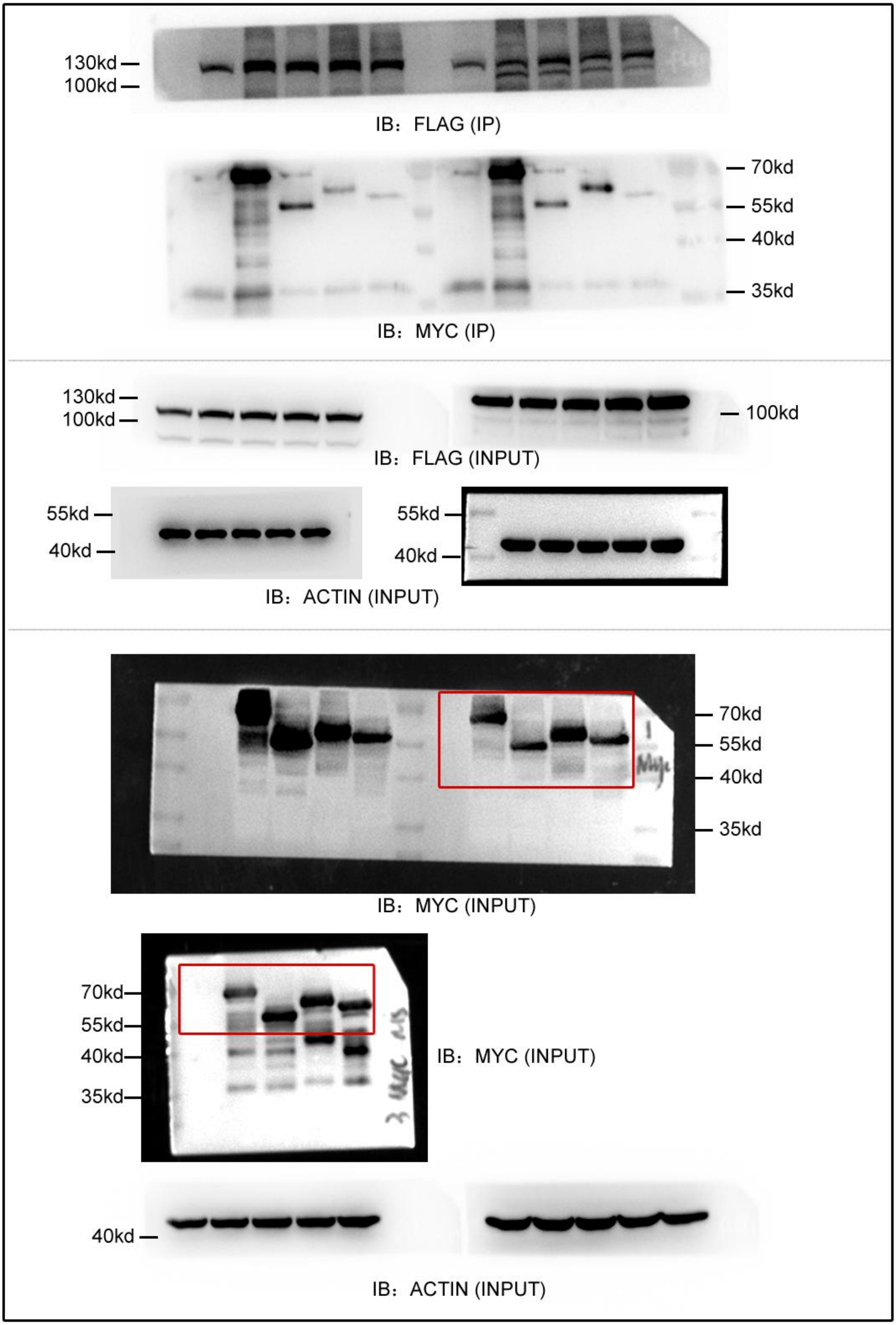
130kd
100kd
IB：FLAG (IP)
70kd
55kd
40kd
35kd
IB：MYC (IP)
130kd
100kd
100kd
IB：FLAG (INPUT)
55kd
40kd
55kd
40kd
IB：ACTIN (INPUT)
70kd
55kd
40kd
35kd
IB：MYC (INPUT)
70kd
55kd
40kd
35kd
IB：MYC (INPUT)
40kd
IB：ACTIN (INPUT)

Figure 7A

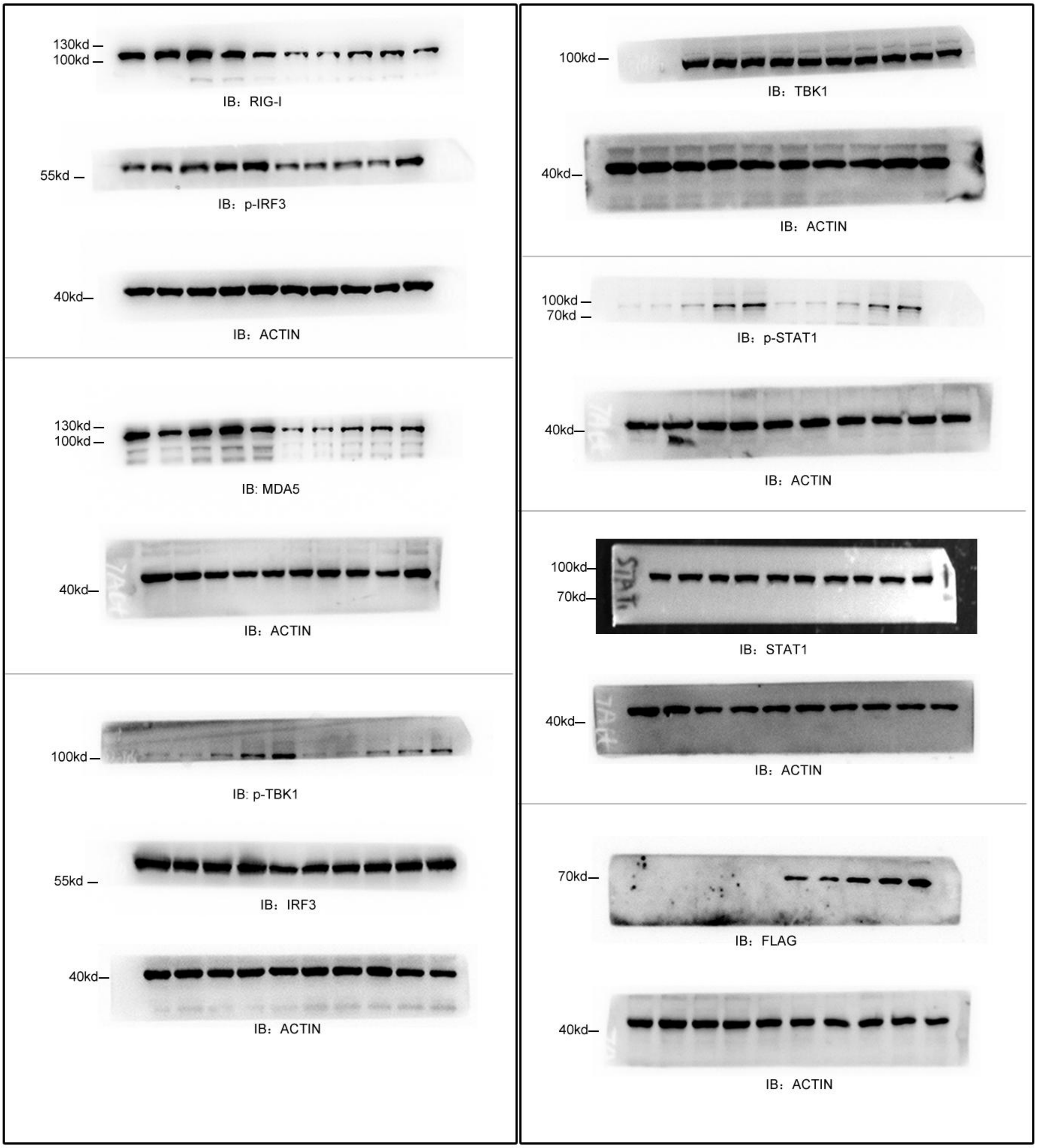

130kd
100kd
IB：RIG-I
55kd
IB：p-IRF3
40kd
IB：ACTIN
130kd
100kd
IB: MDA5
40kd
IB：ACTIN
100kd
IB: p-TBK1
55kd
IB：IRF3
40kd
IB：ACTIN
100kd
IB：TBK1
40kd
IB：ACTIN
100kd
70kd
IB：p-STAT1
40kd
IB：ACTIN
100kd
70kd
IB：STAT1
40kd
IB：ACTIN
70kd
IB：FLAG
40kd
IB：ACTIN

Figure 7B

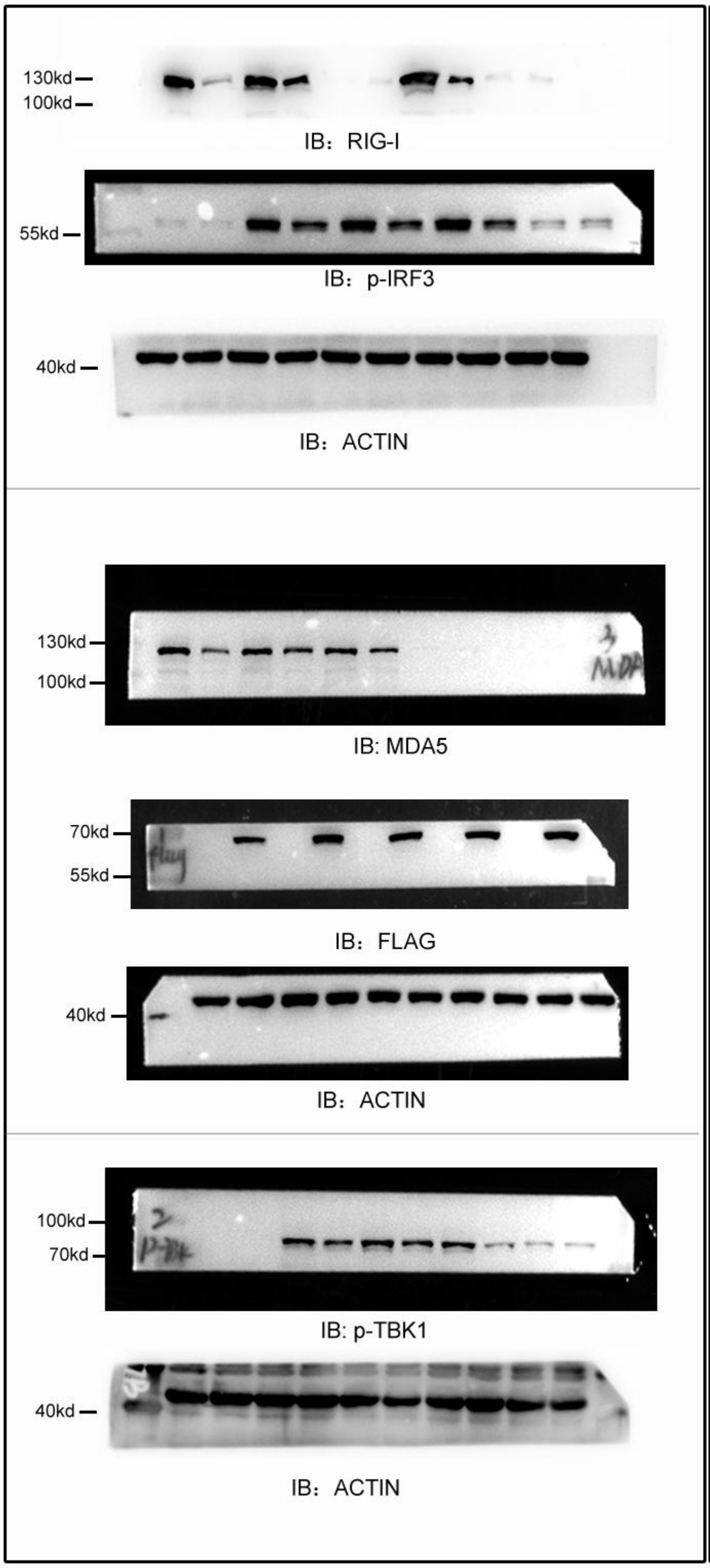

130kd
100kd
IB：RIG-I
55kd
IB：p-IRF3
40kd
IB：ACTIN
130kd
100kd
IB: MDA5
70kd
55kd
IB：FLAG
40kd
IB：ACTIN
100kd
70kd
IB: p-TBK1
40kd
IB：ACTIN

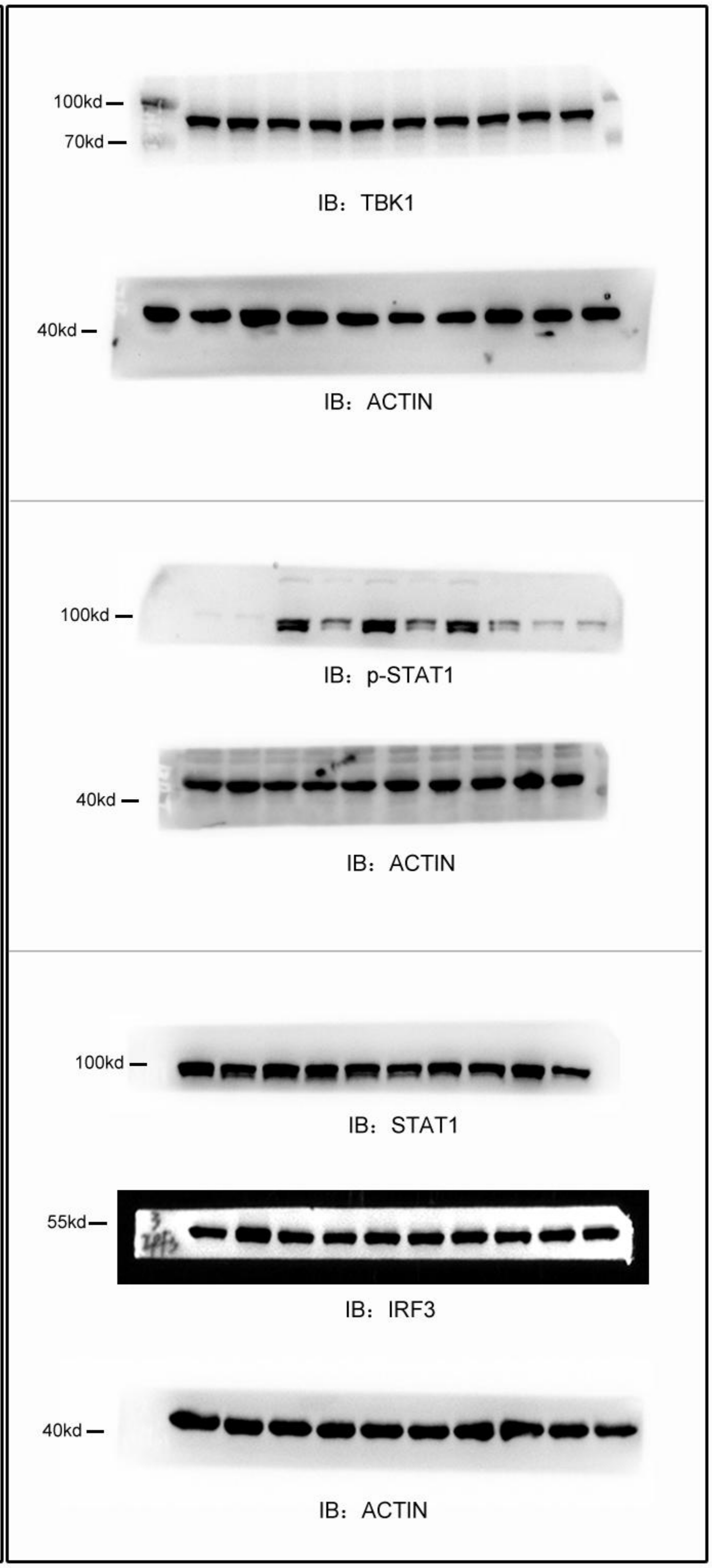

100kd
70kd
IB：TBK1
40kd
IB：ACTIN
100kd
IB：p-STAT1
40kd
IB：ACTIN
100kd
IB：STAT1
55kd
IB：IRF3
40kd
IB：ACTIN

Supplementary Figure 2A

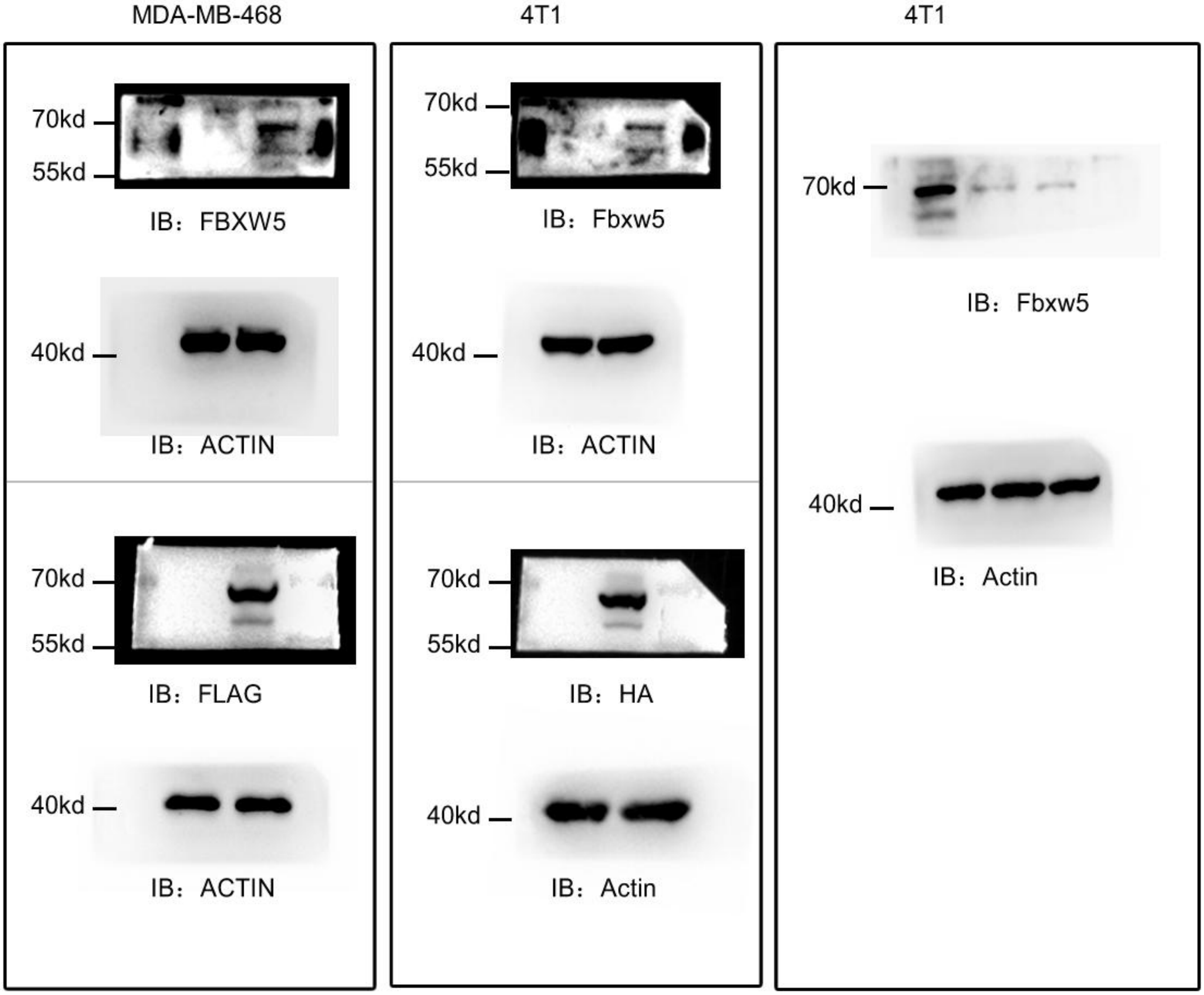

Supplementary Figure 4F

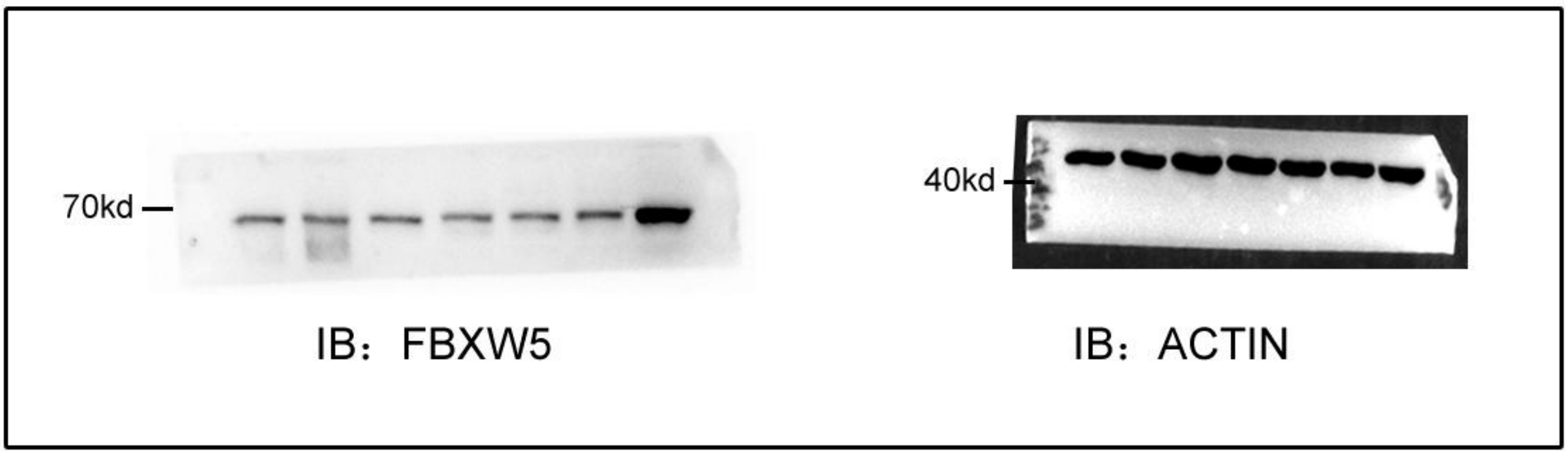

Supplementary Figure 5A

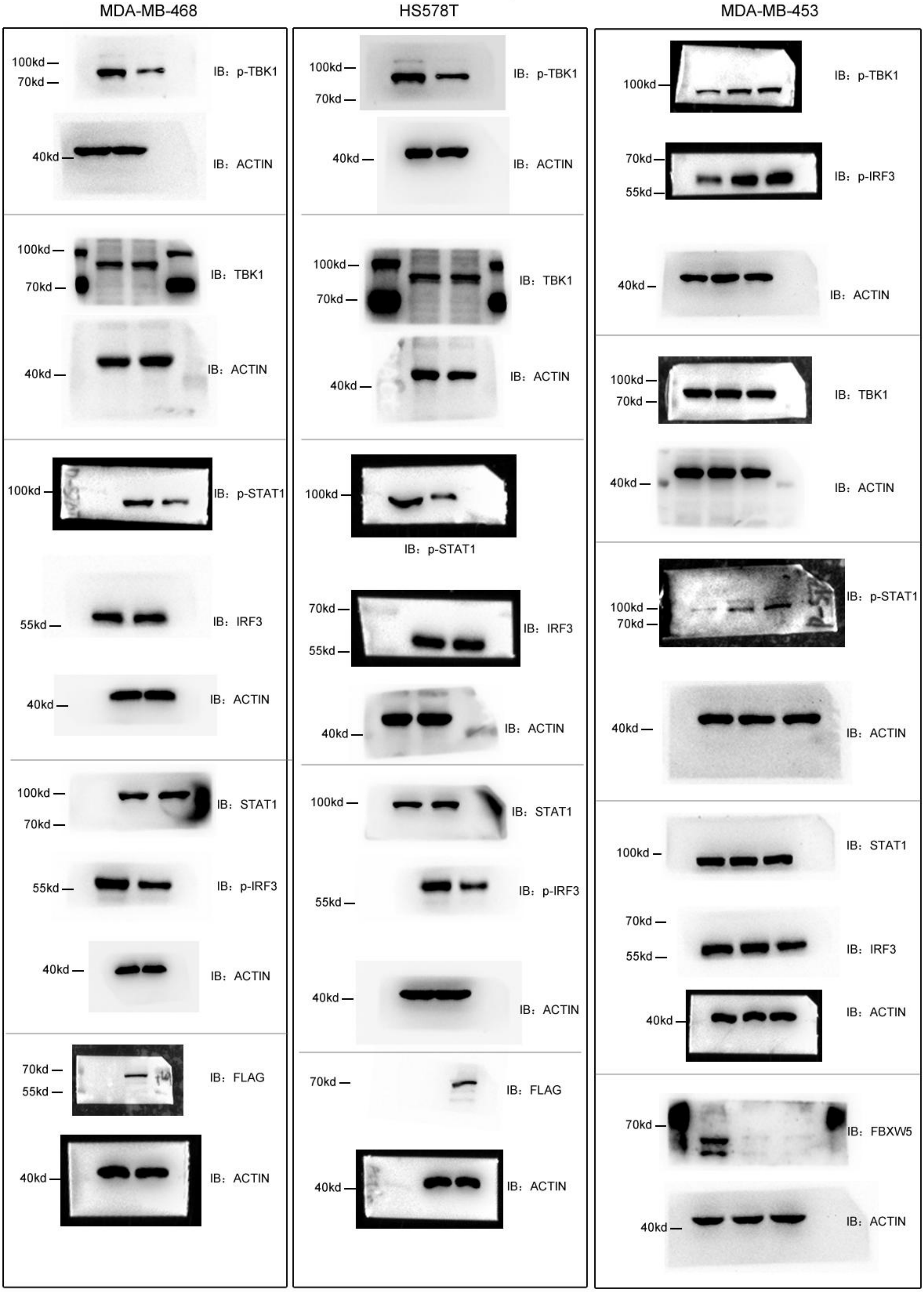

Supplementary Figure 5H

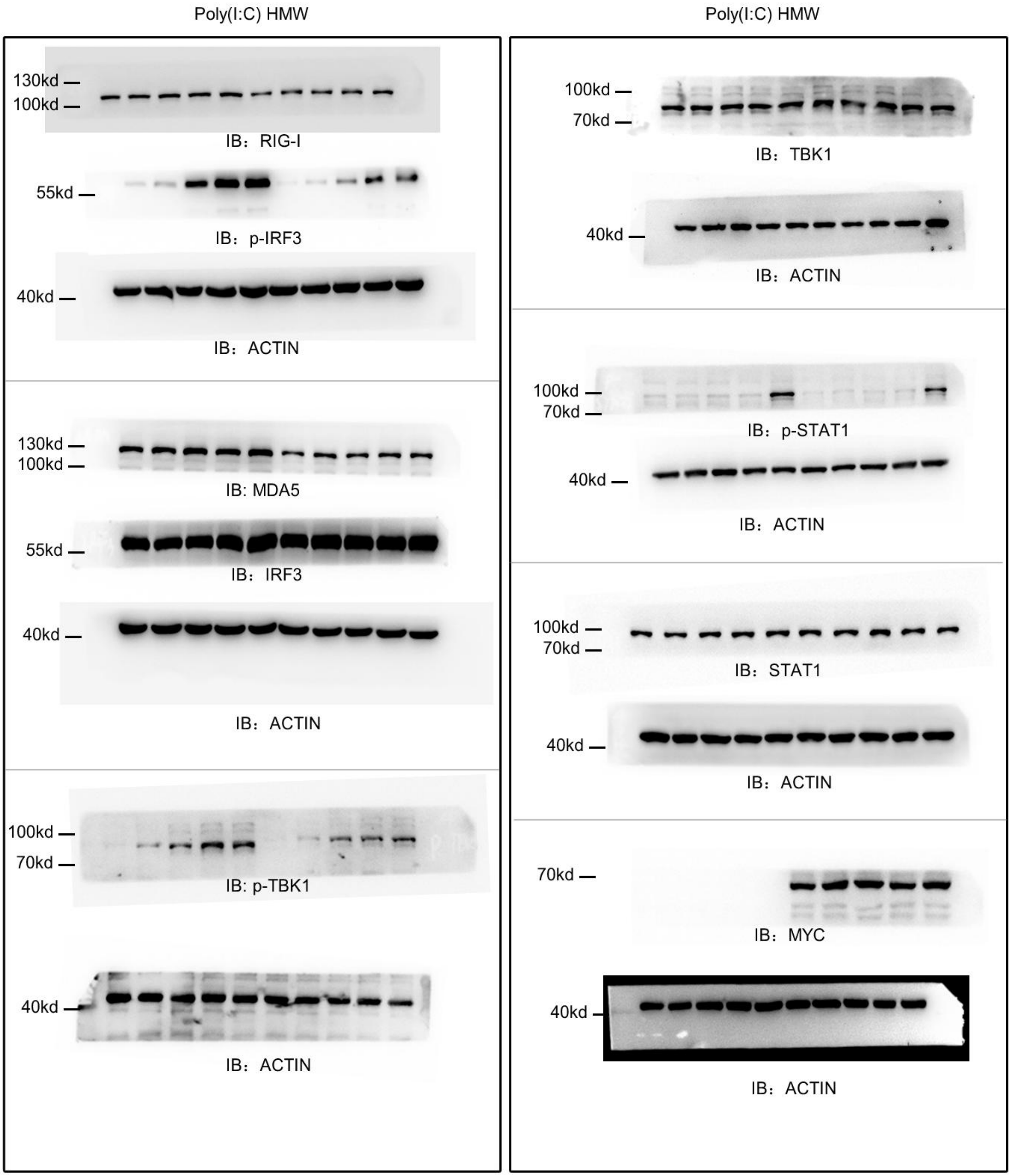

Supplementary Figure 5I

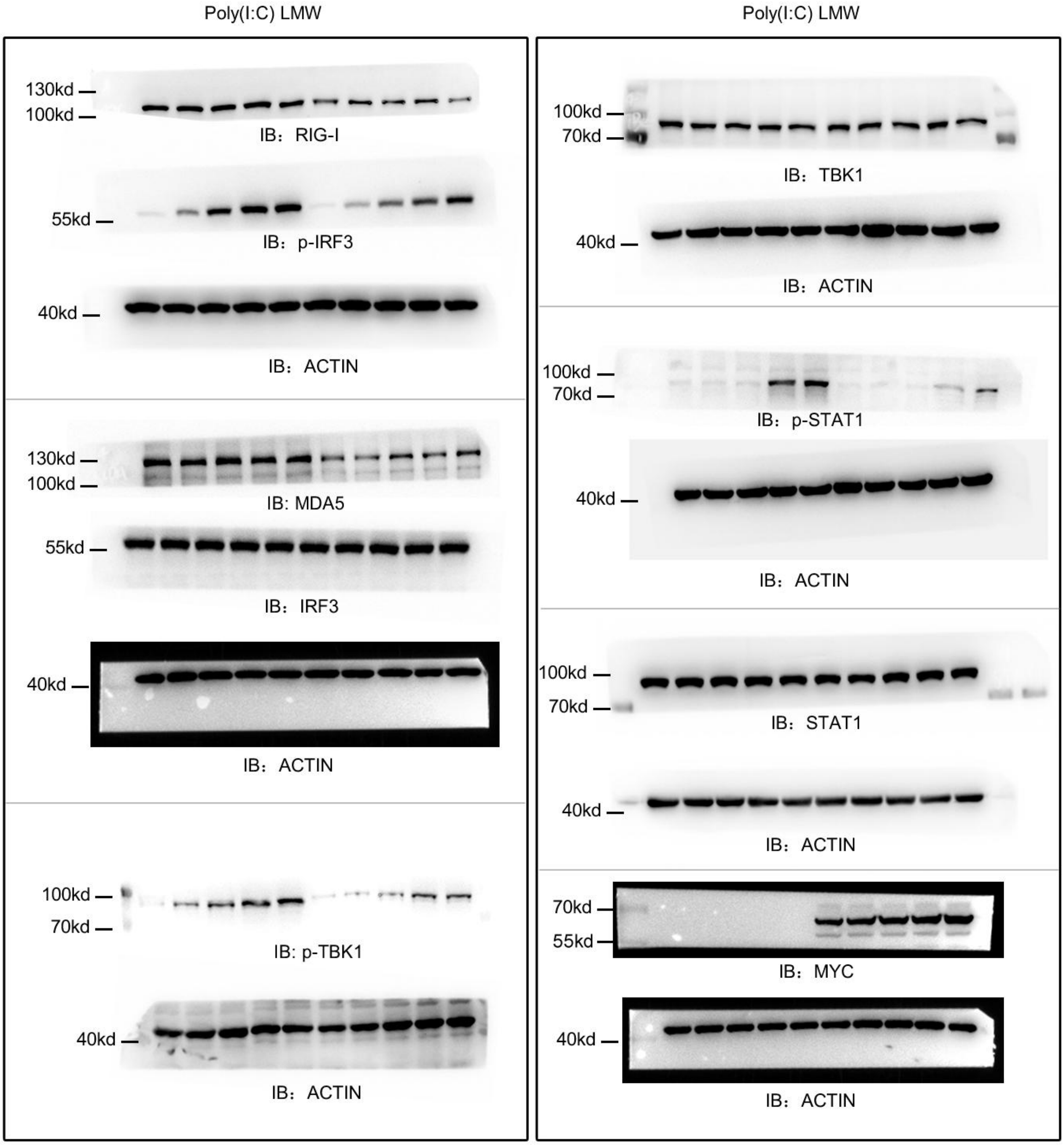

Supplementary Figure 5J

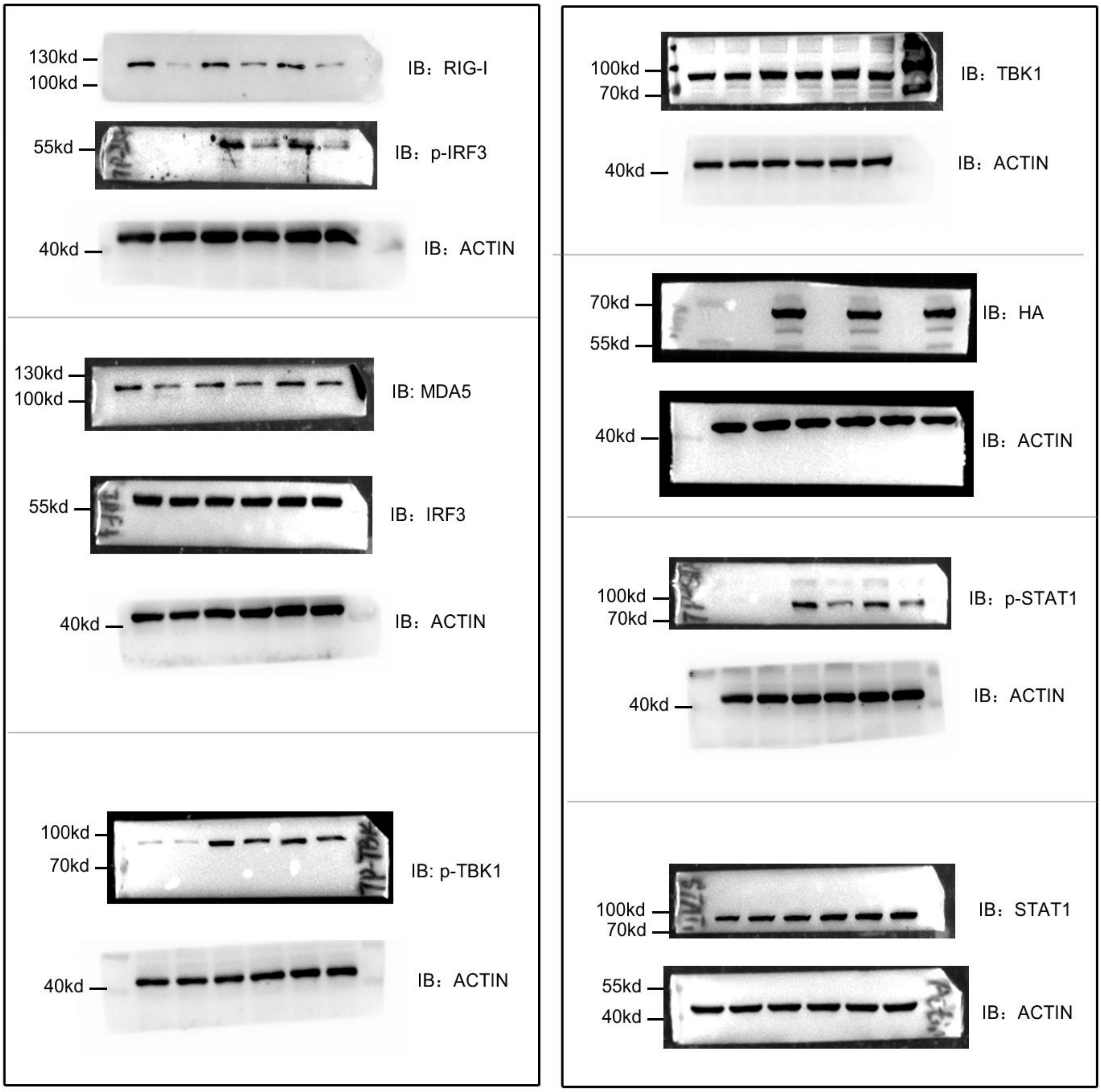

Supplementary Figure 5K

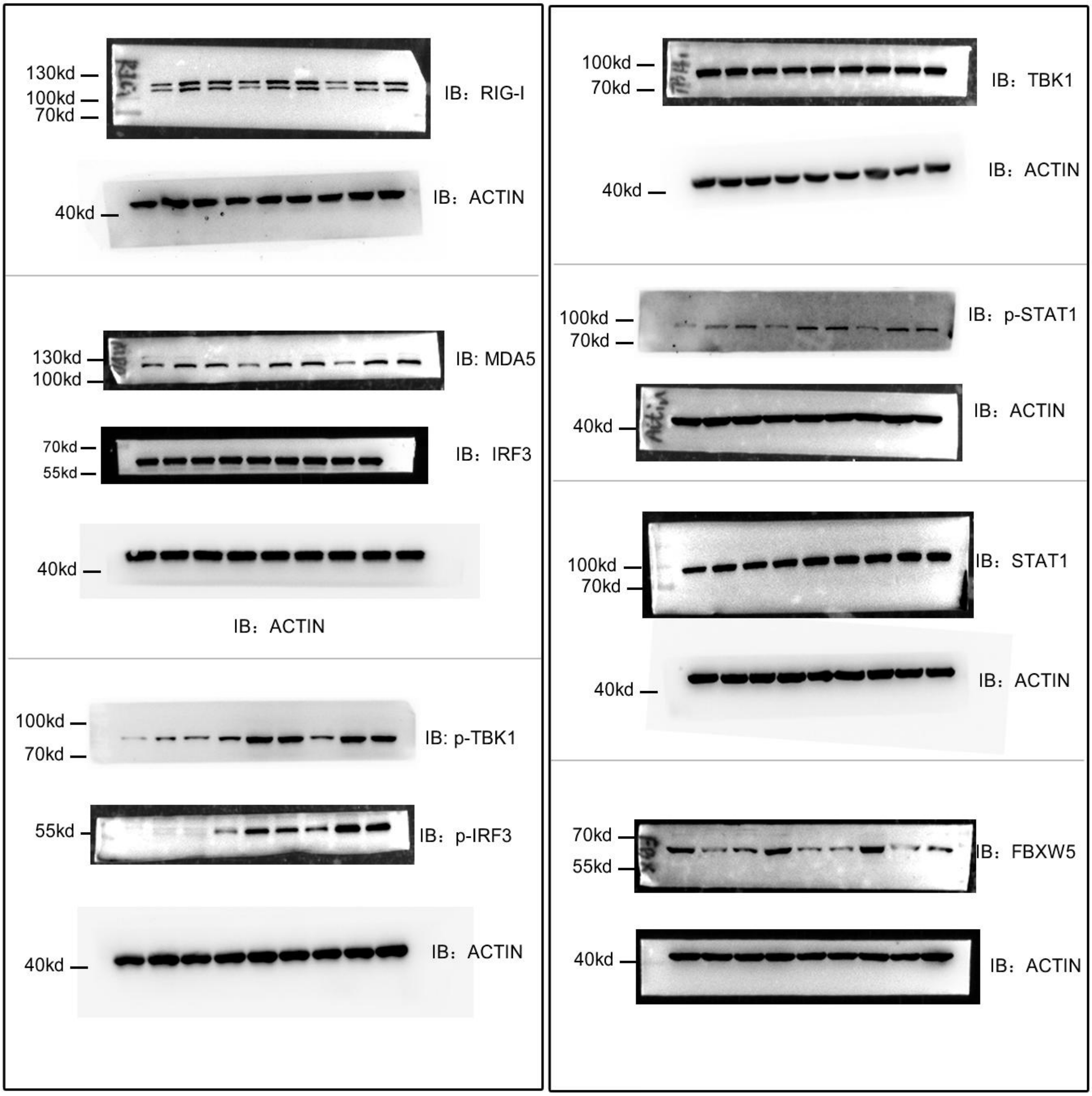

Supplementary Figure 7A

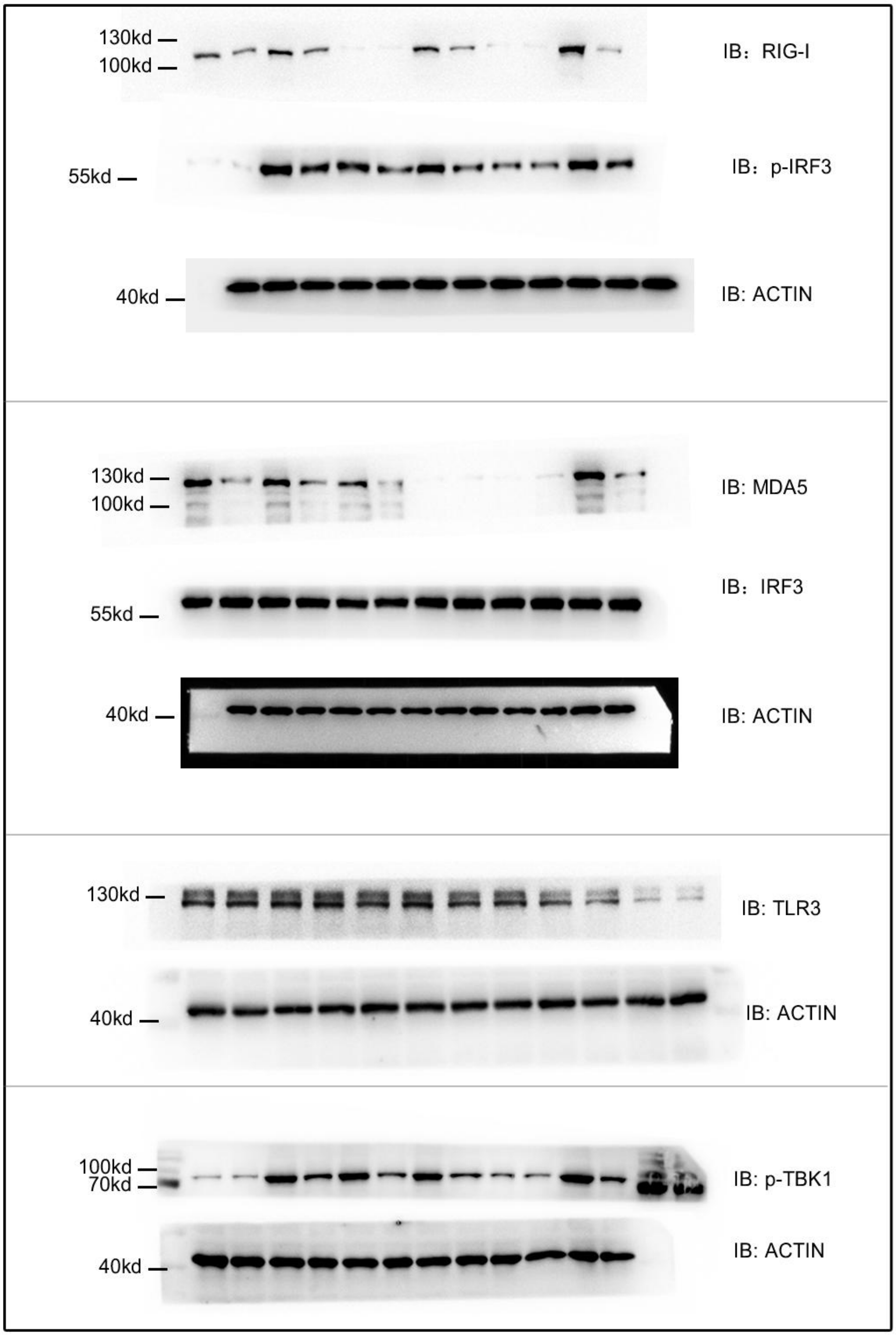

Supplementary Figure 7A

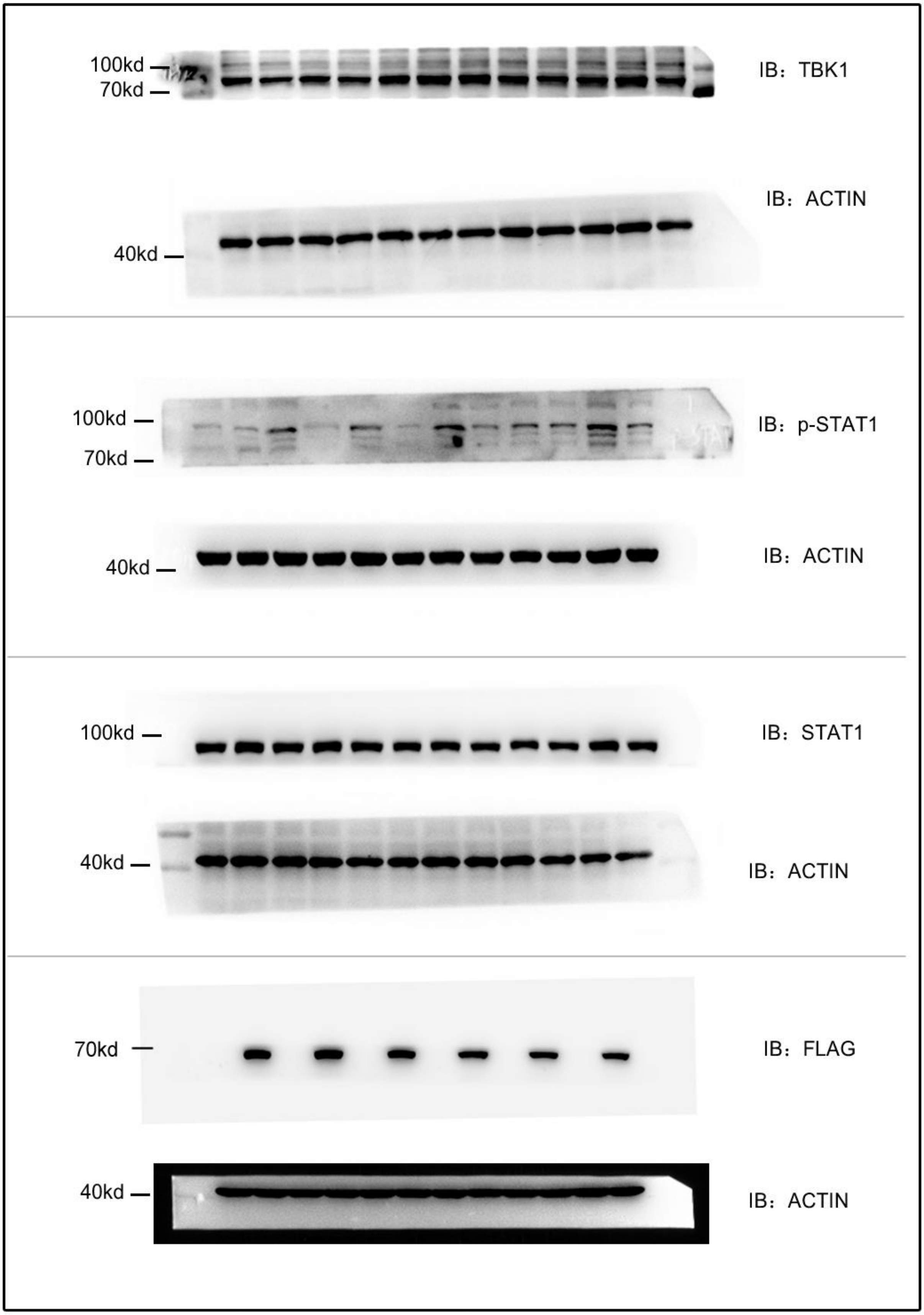

Supplementary Figure 7D

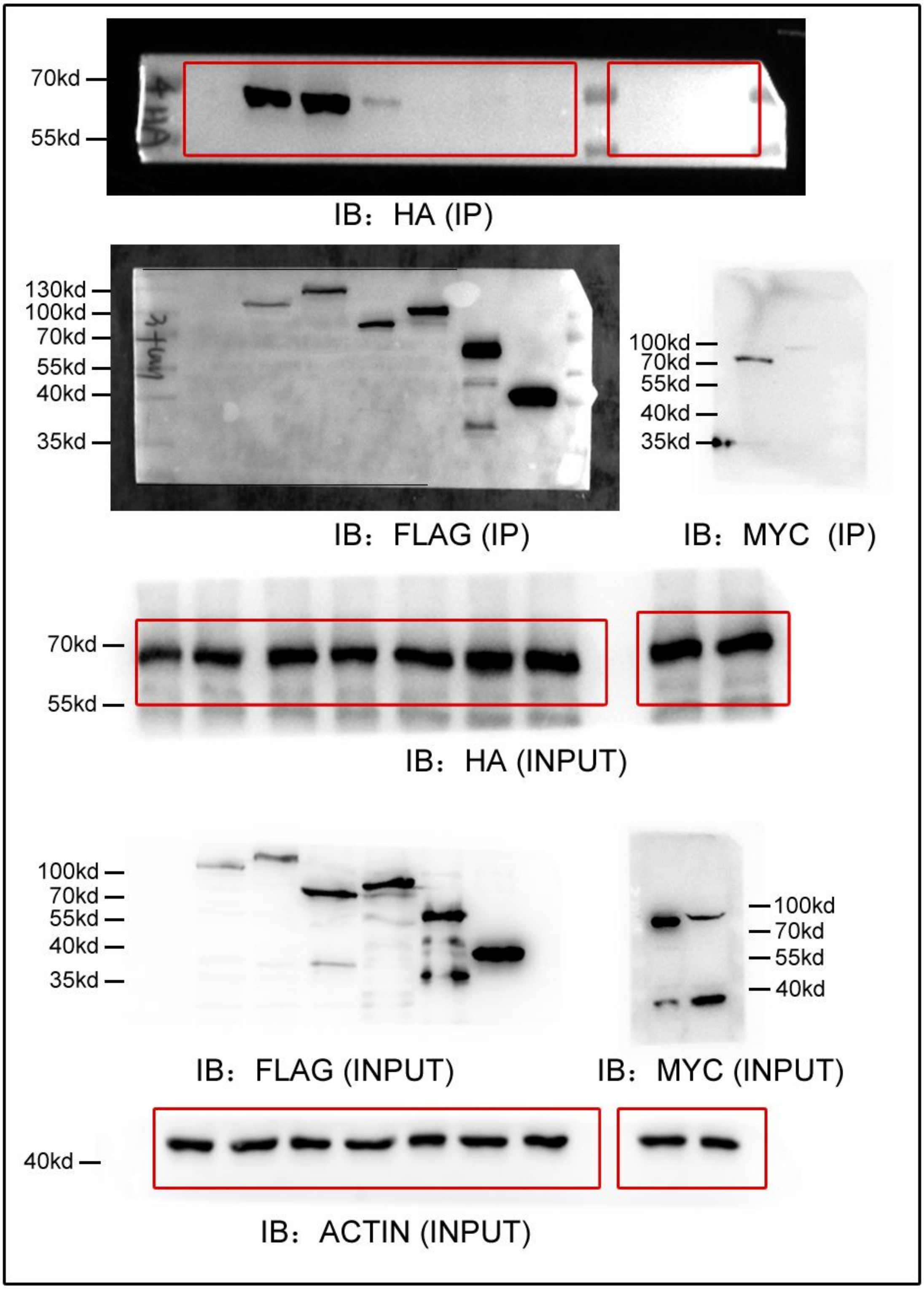

Supplementary Figure 7F

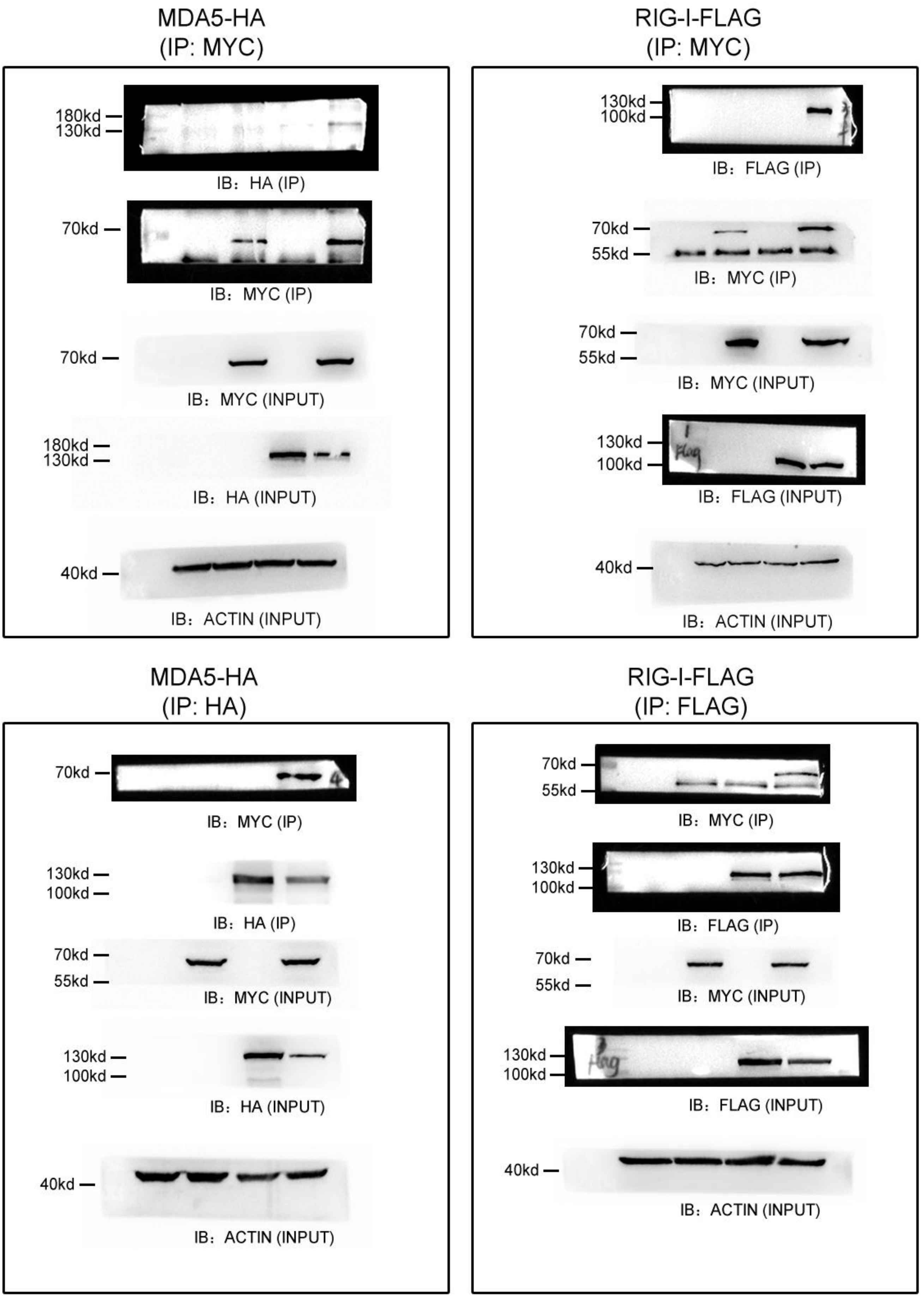

Supplementary Figure 7G

BT549

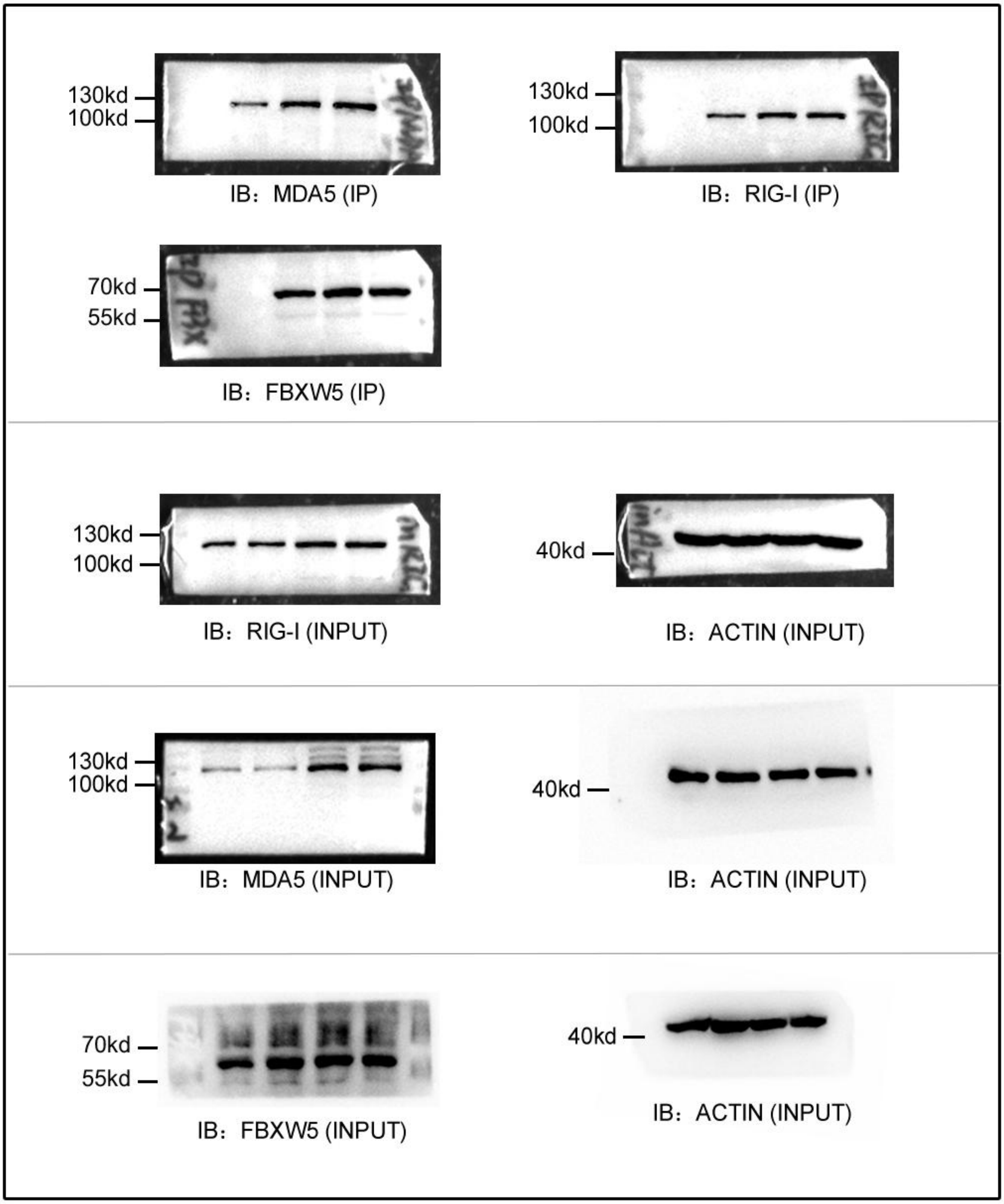

Supplementary Figure 7G

MDA-MB-453

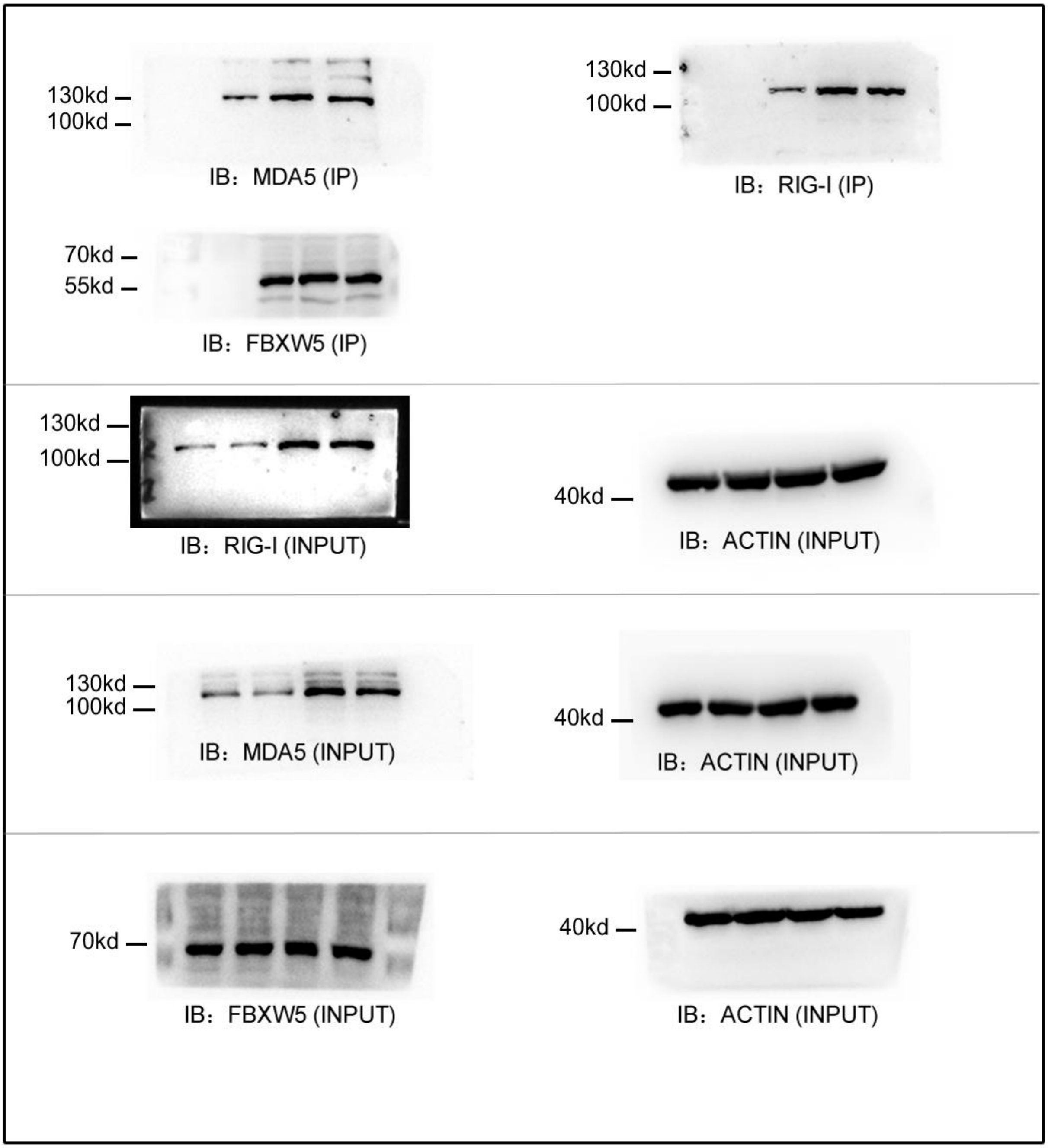

Supplementary Figure 8A

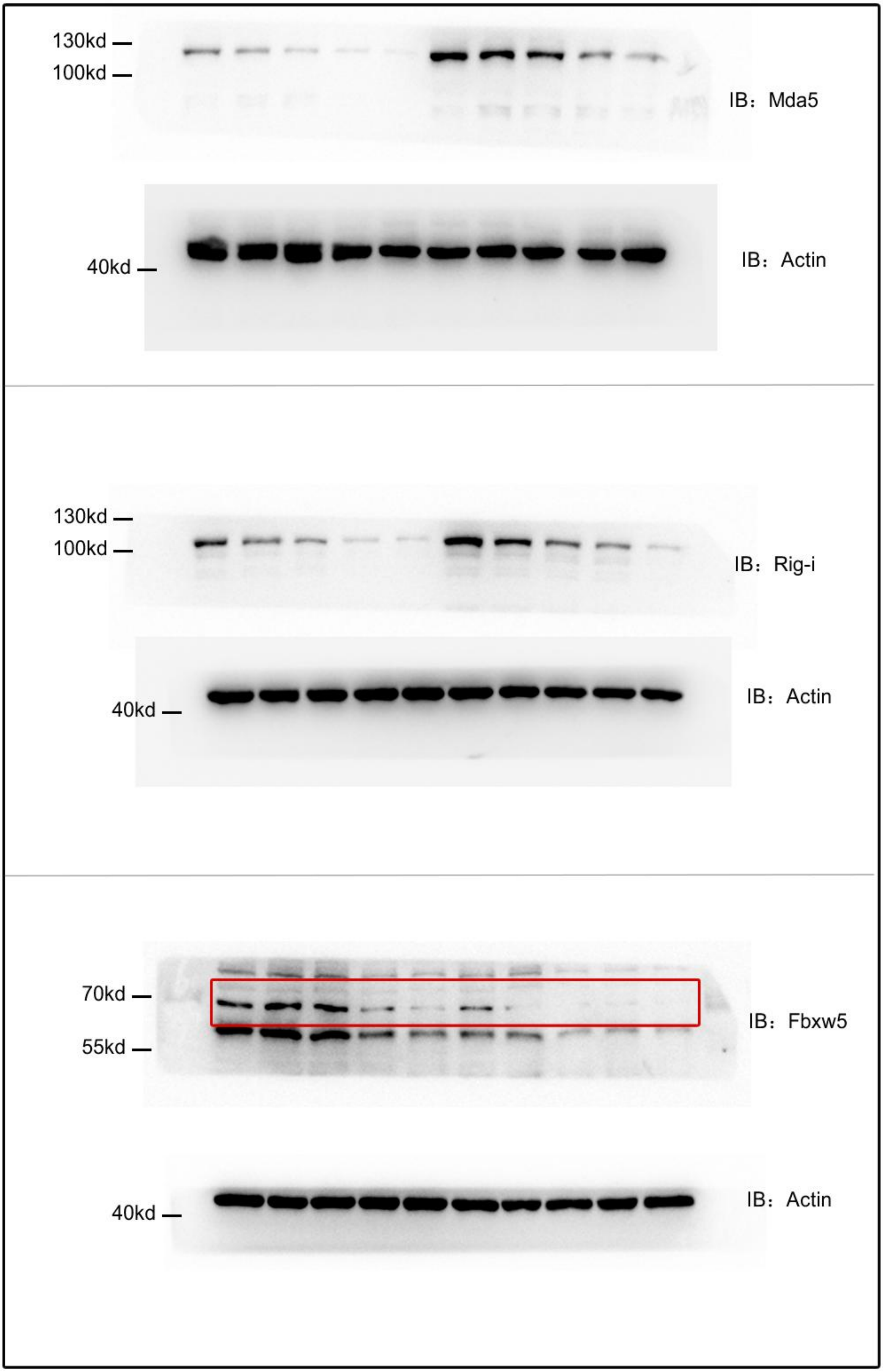

Supplementary Figure 8B

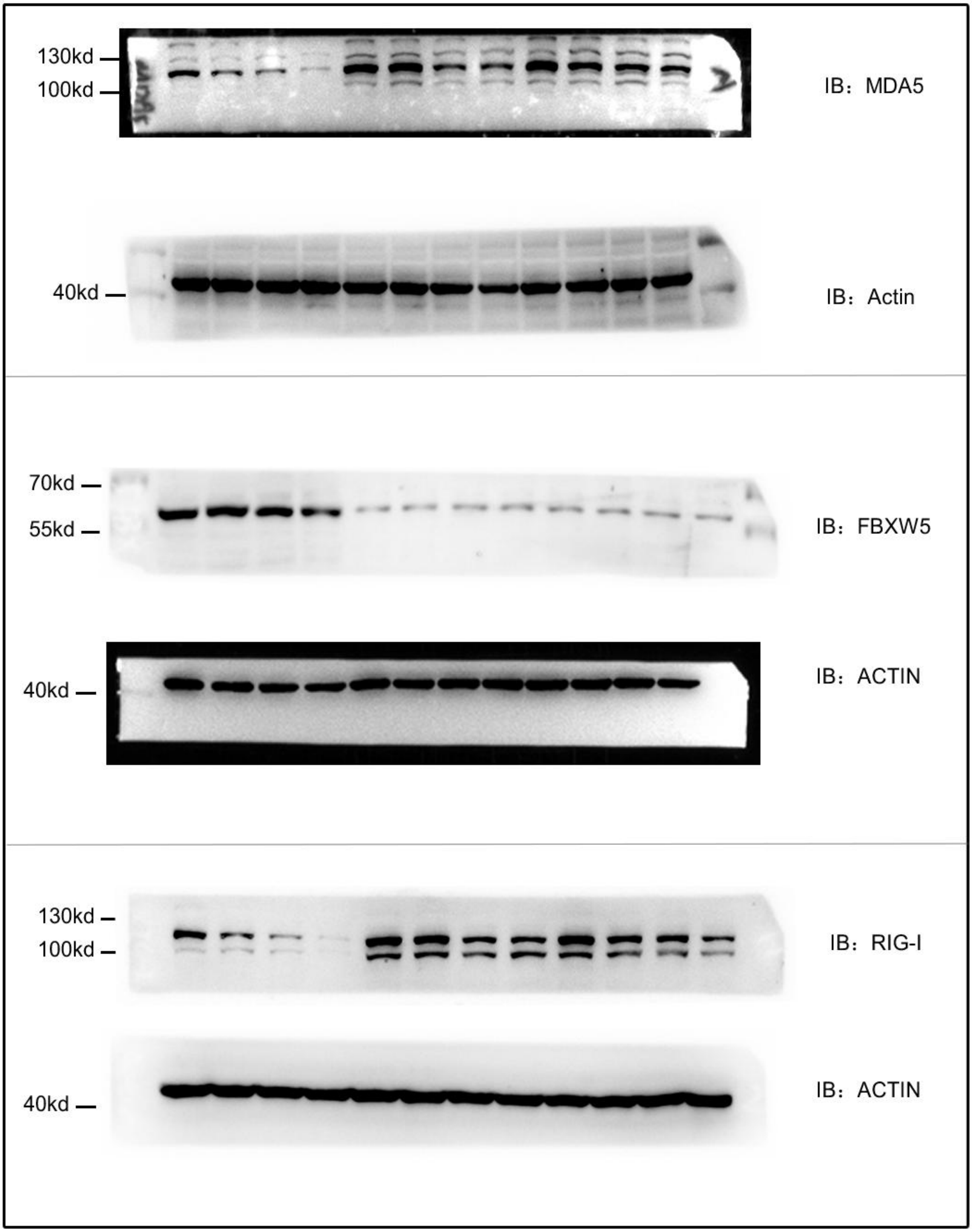

Supplementary Figure 8C

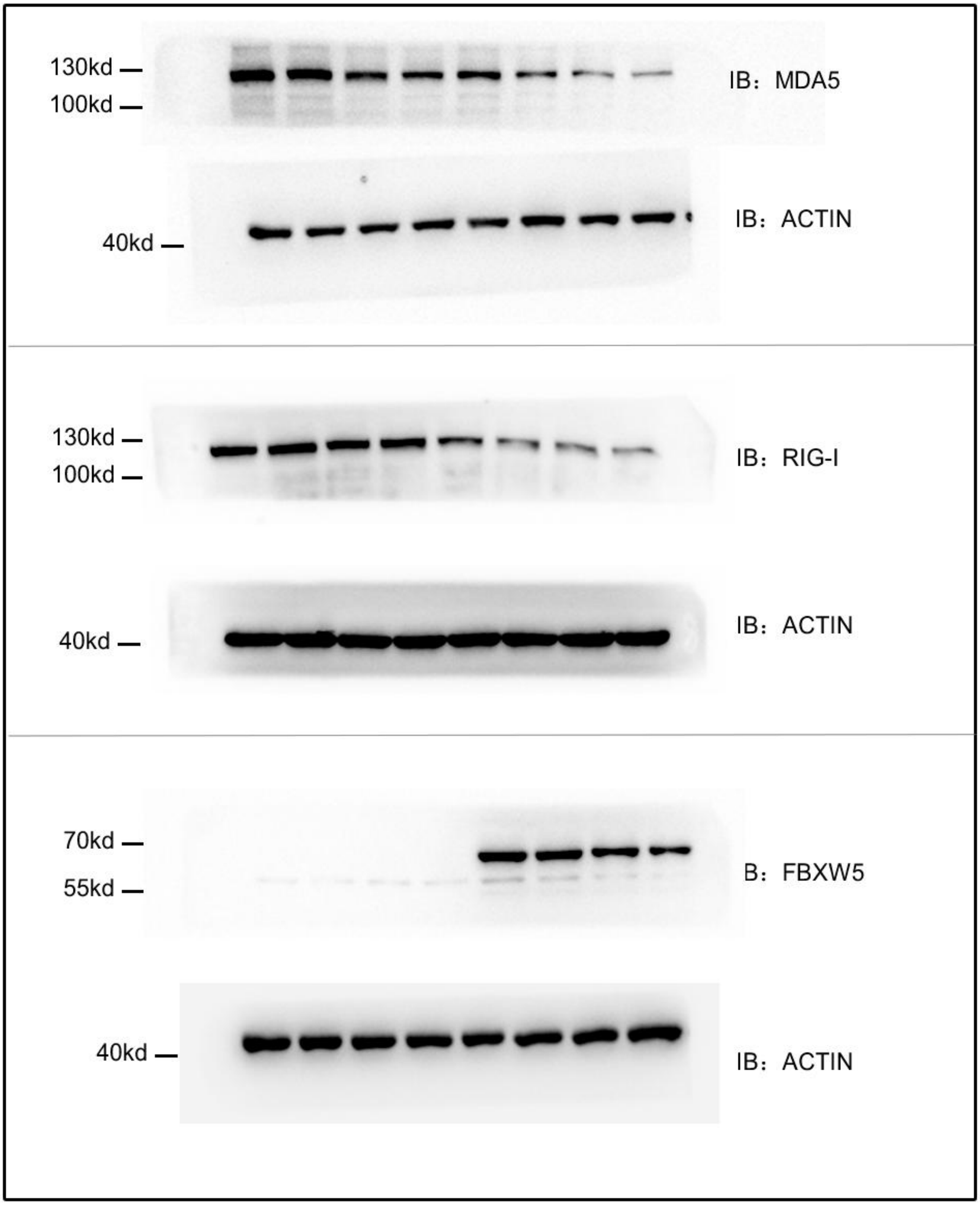

Supplementary Figure 8D

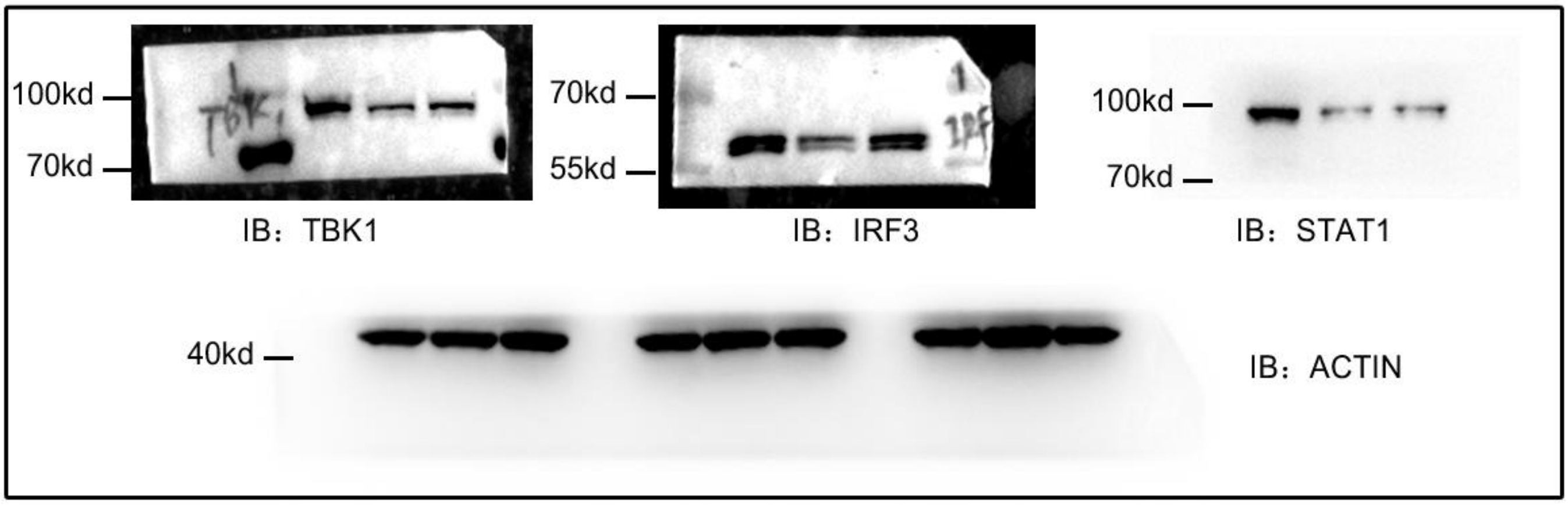

Supplementary Figure 8E

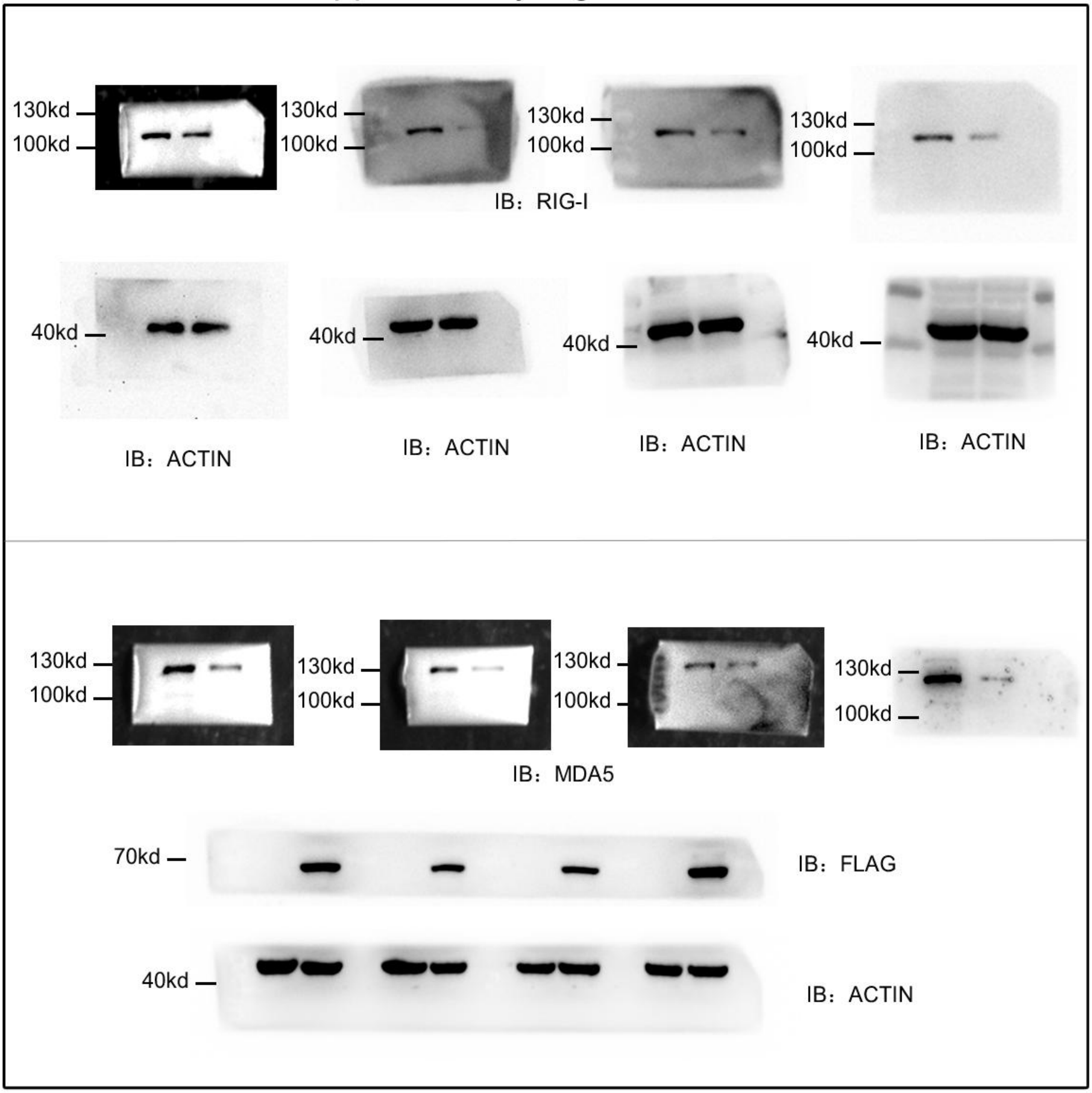

Supplementary Figure 8H

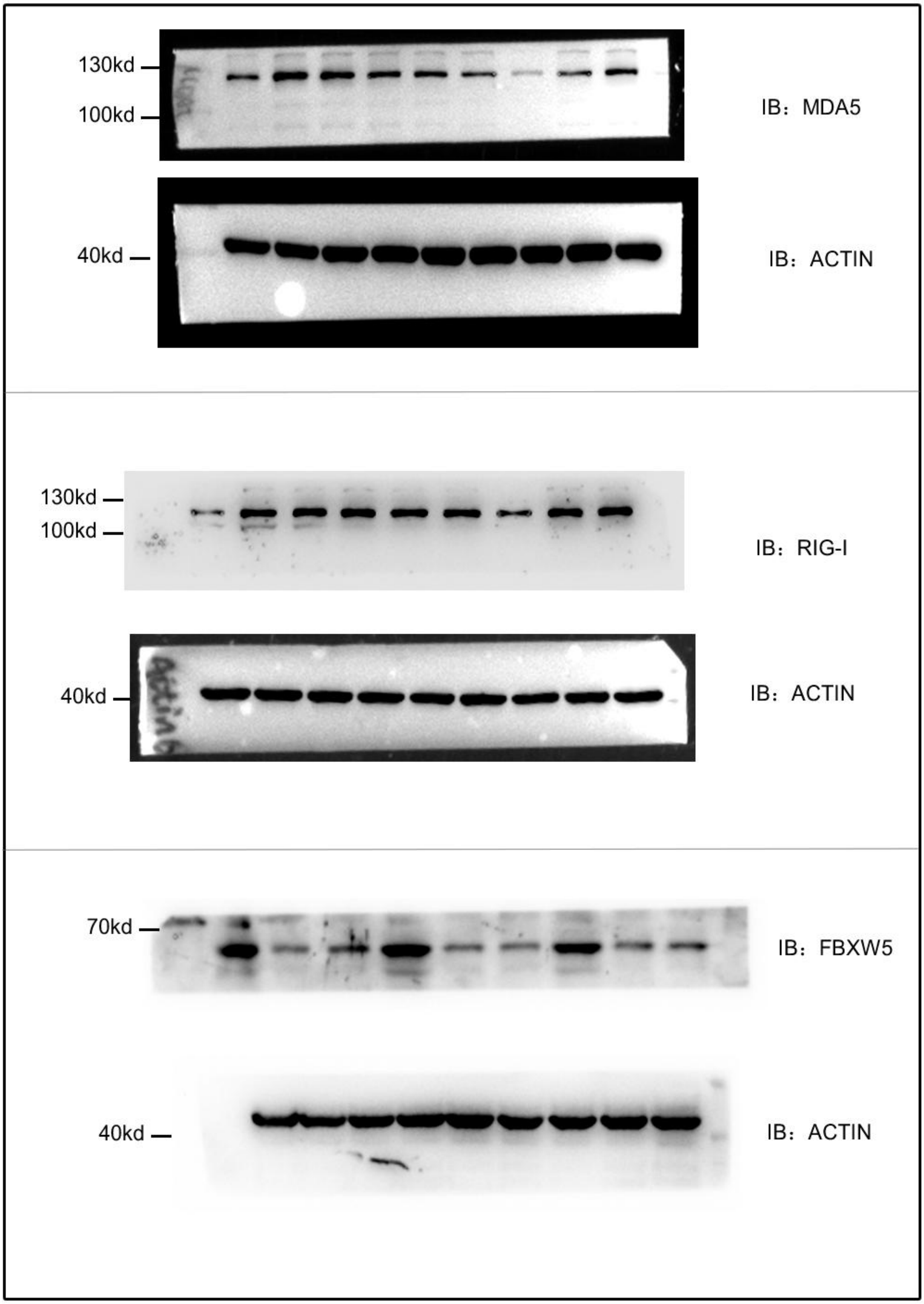

Supplementary Figure 8G

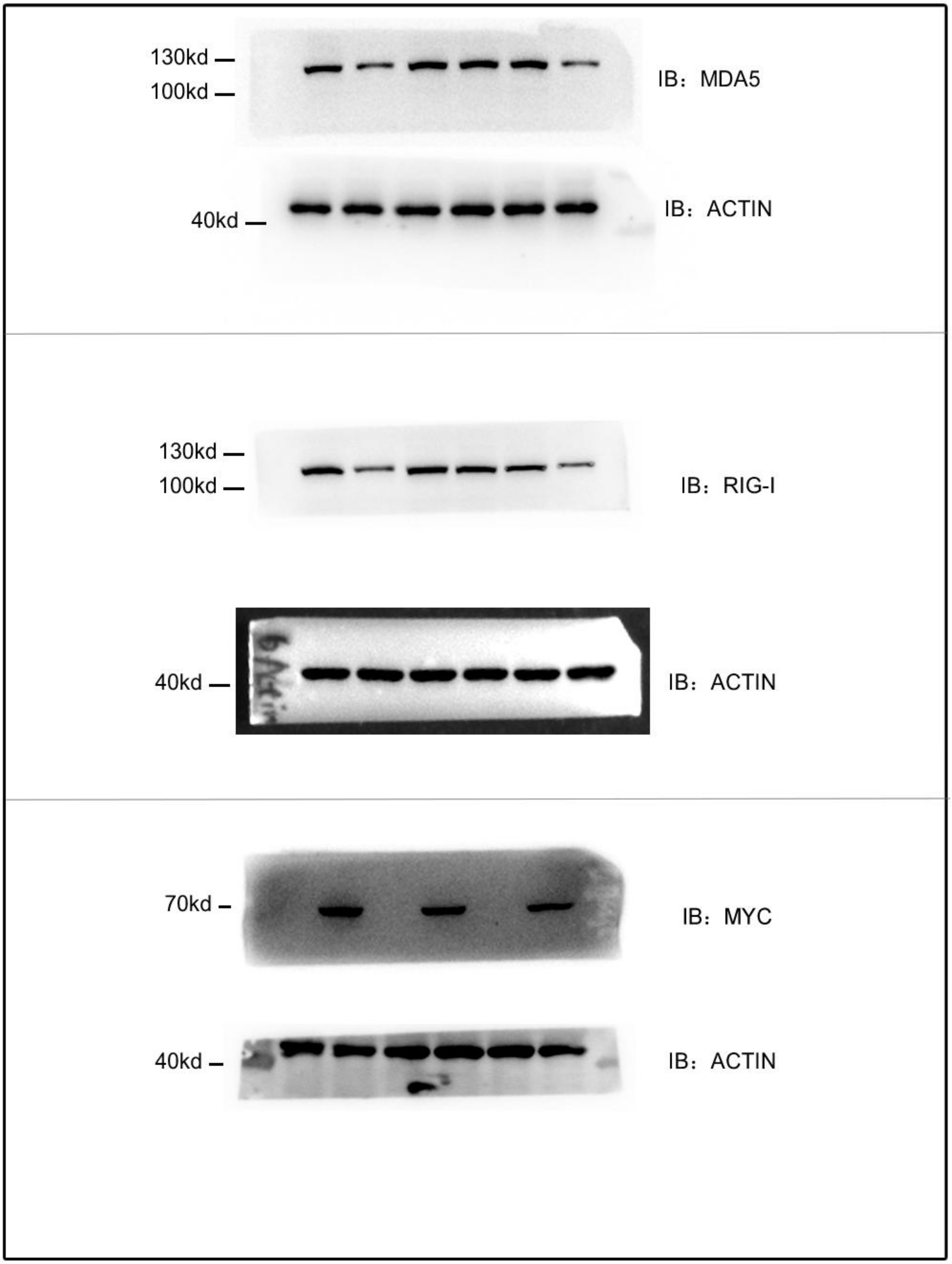

Supplementary Figure 9A

MDA5-FLAG

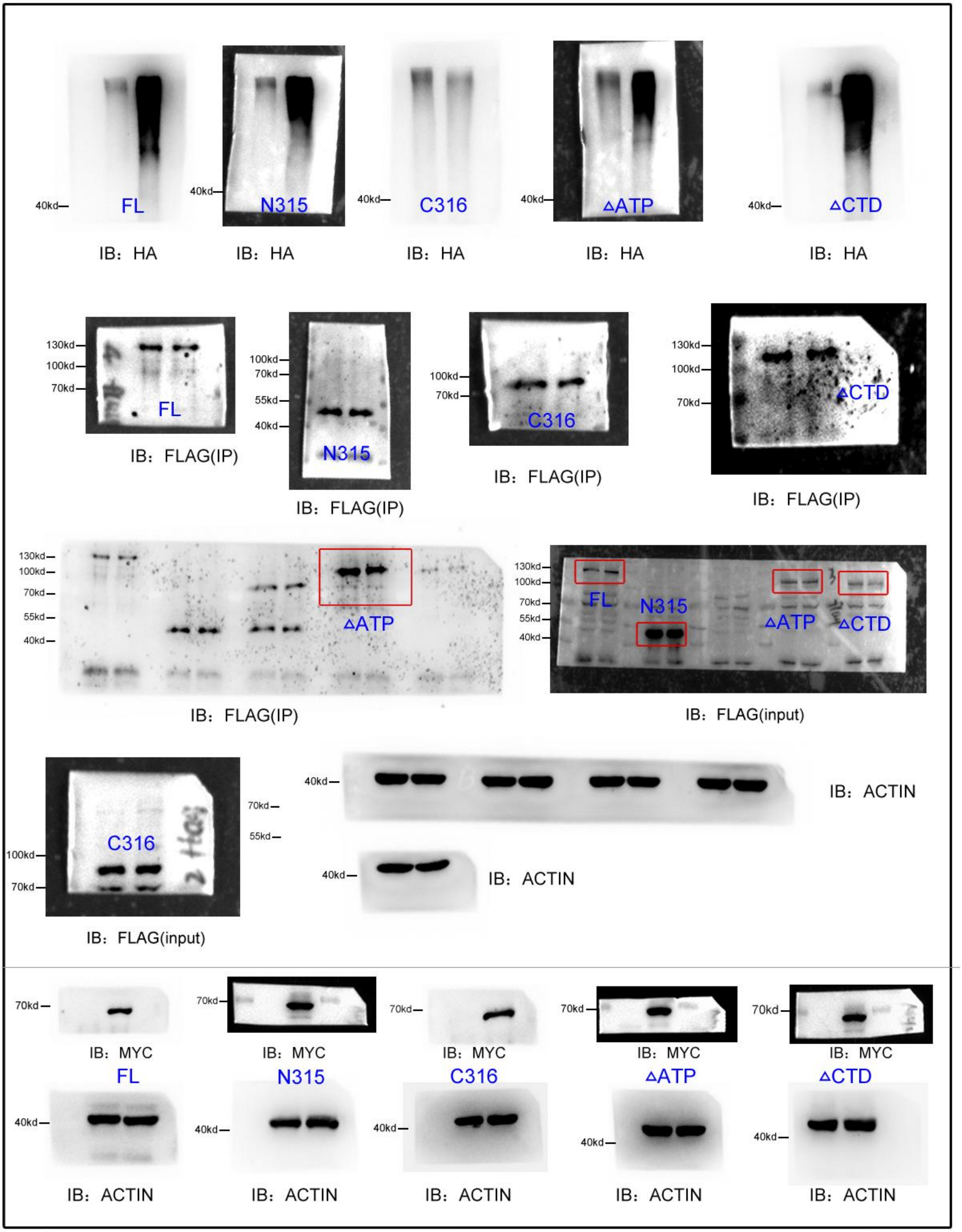

Supplementary Figure 9A

RIG-I-FLAG

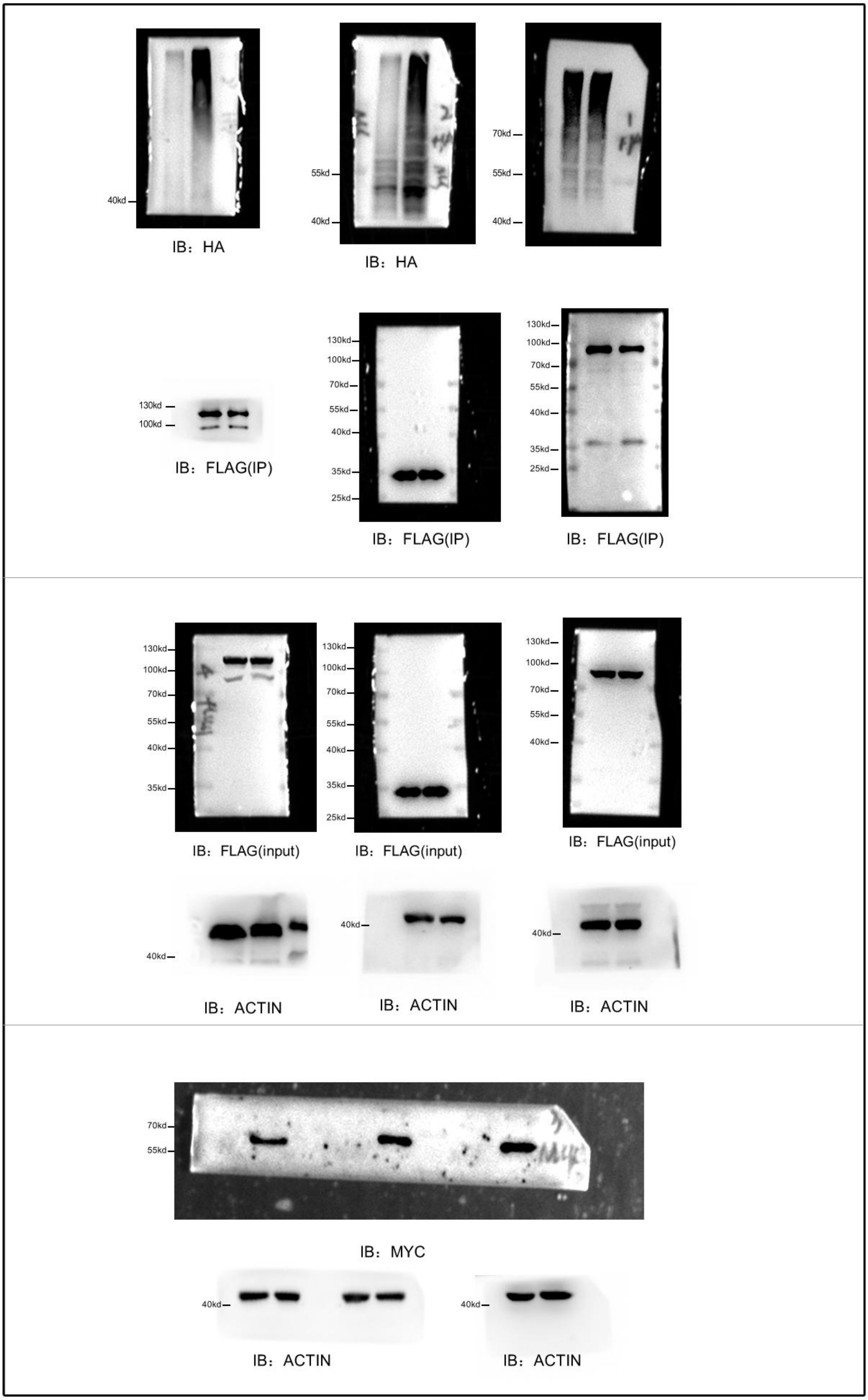

Supplementary Figure 9B

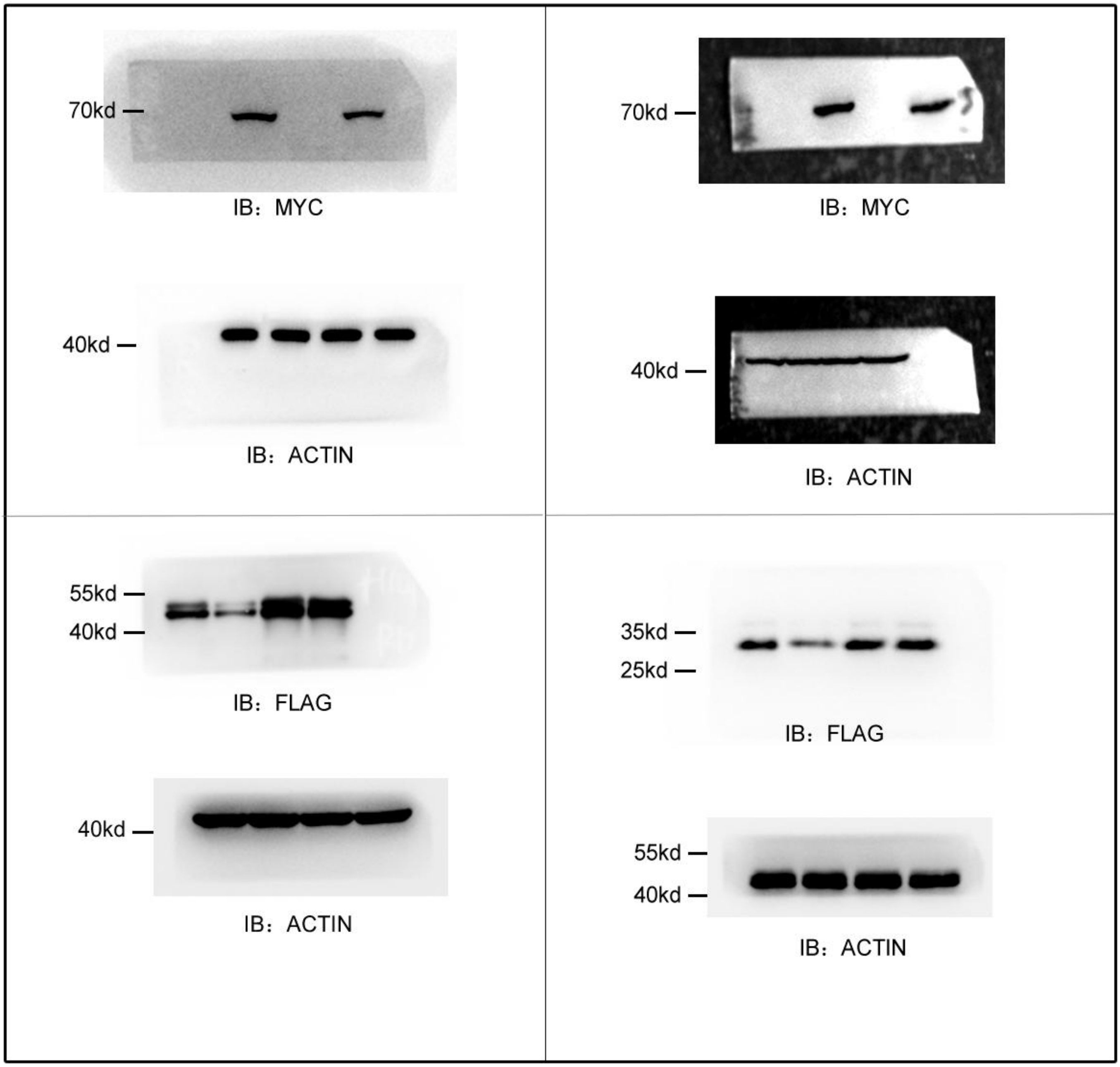

Supplementary Figure 9F

siRBX1

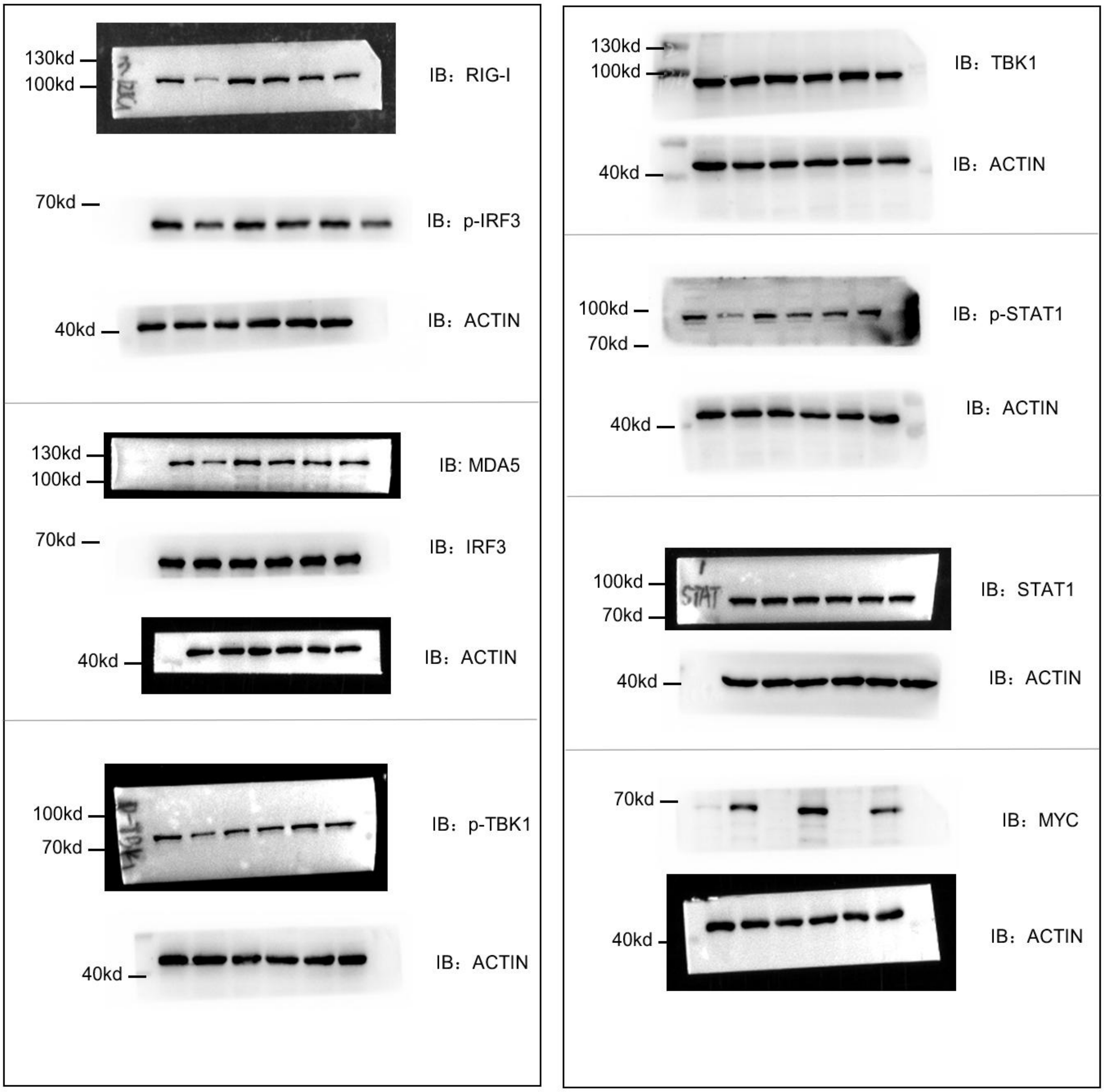

Supplementary Figure 9F

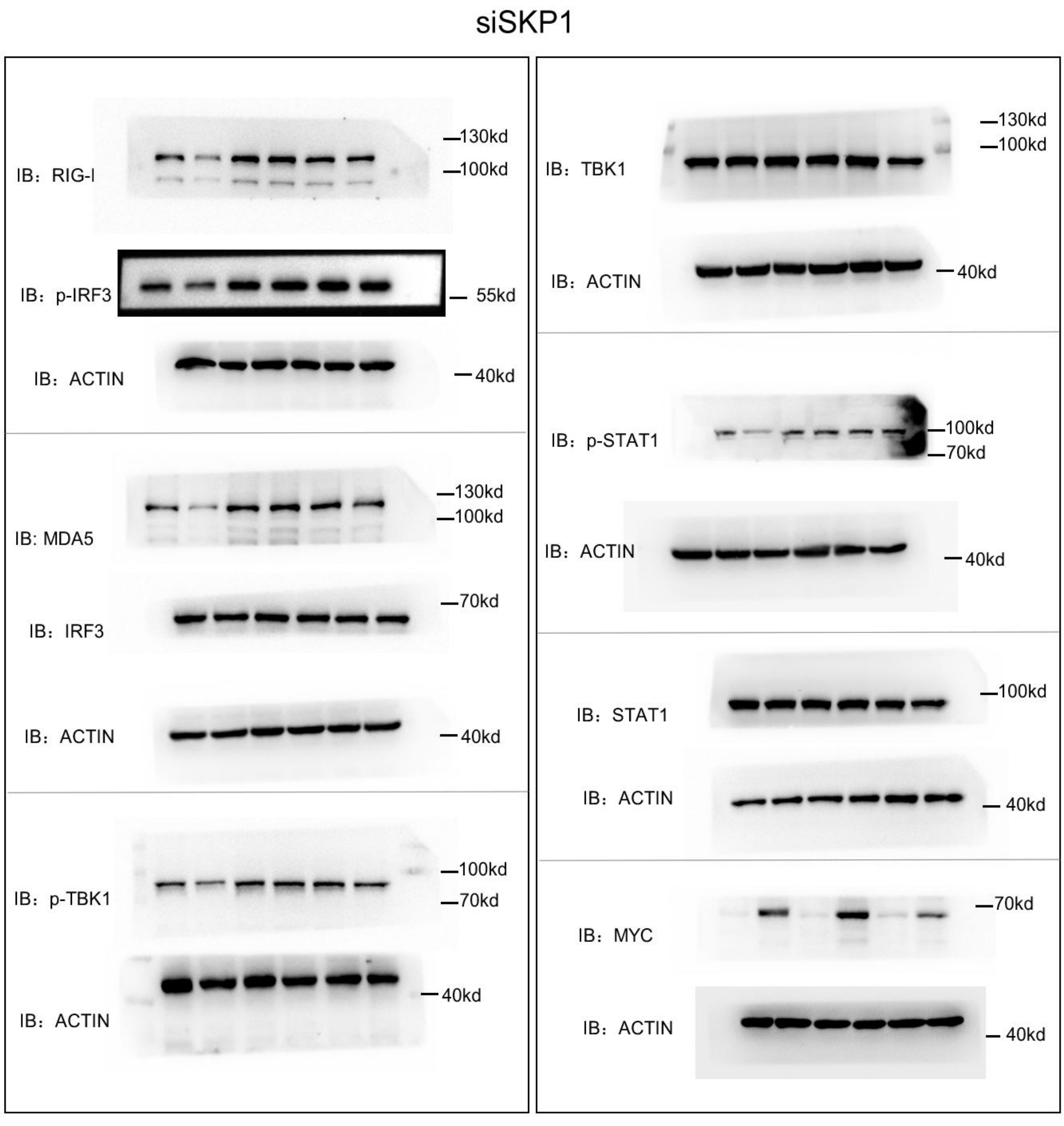

Supplementary Figure 9G

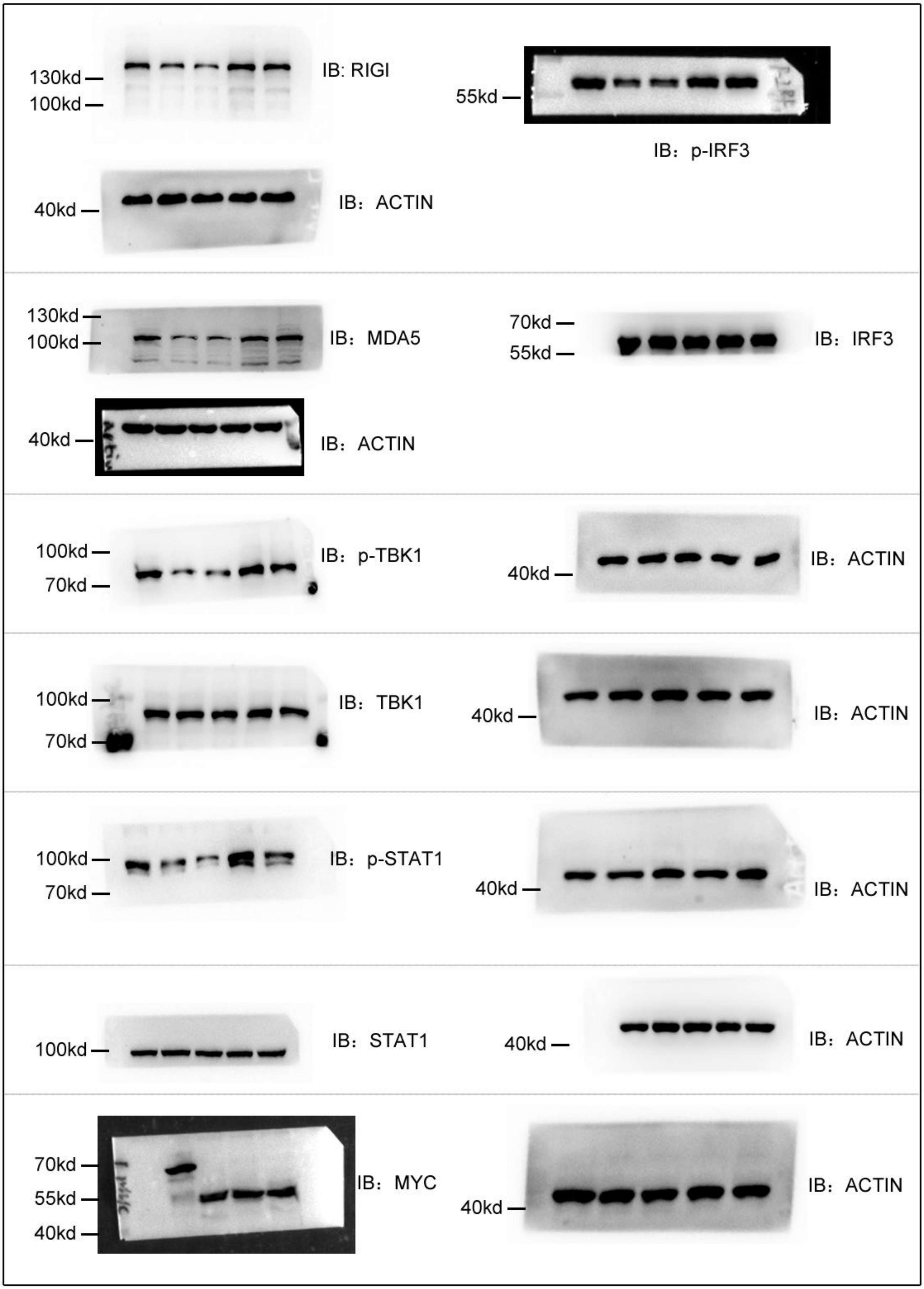

Supplementary Figure 10A

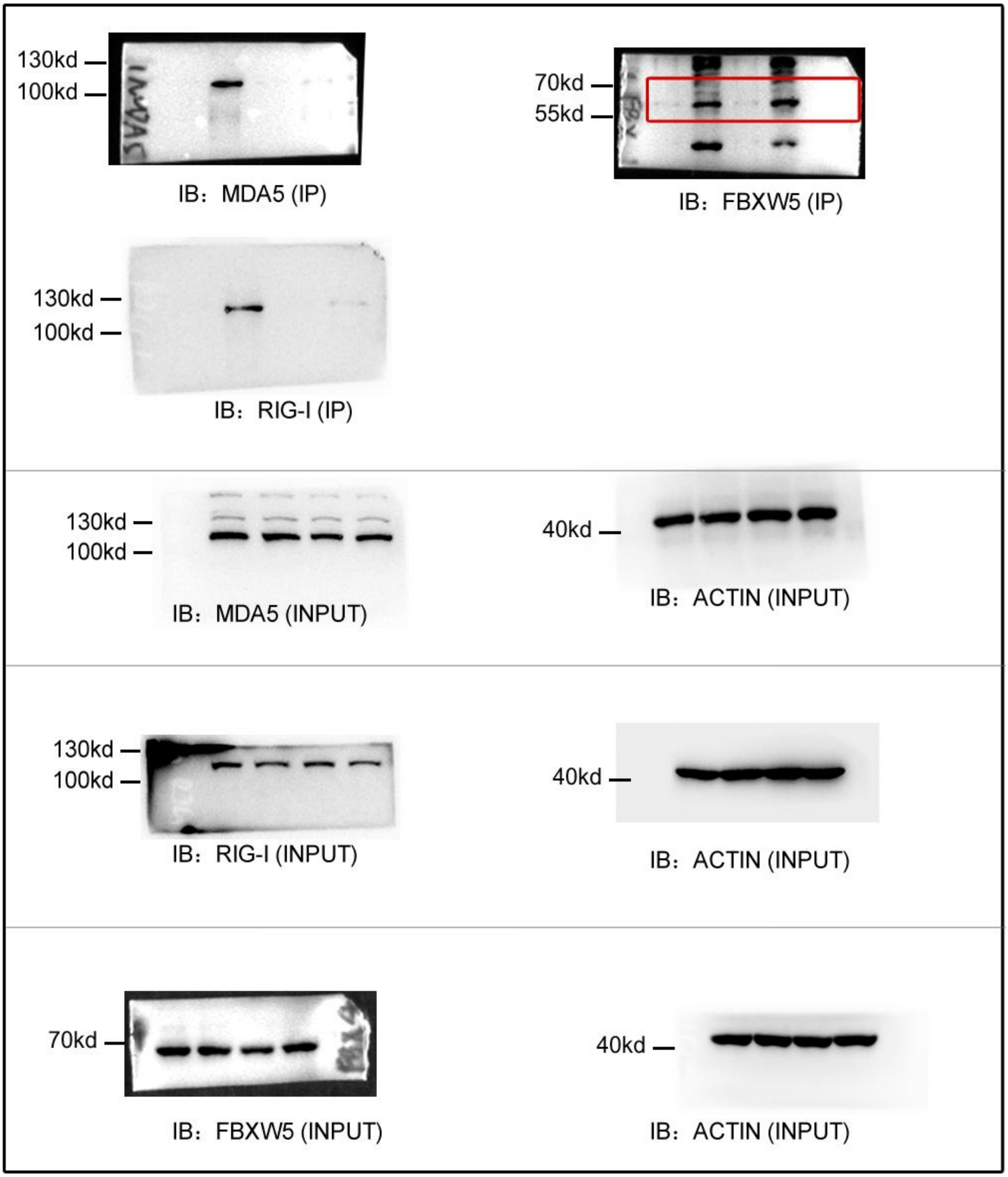

Supplementary Figure 10B

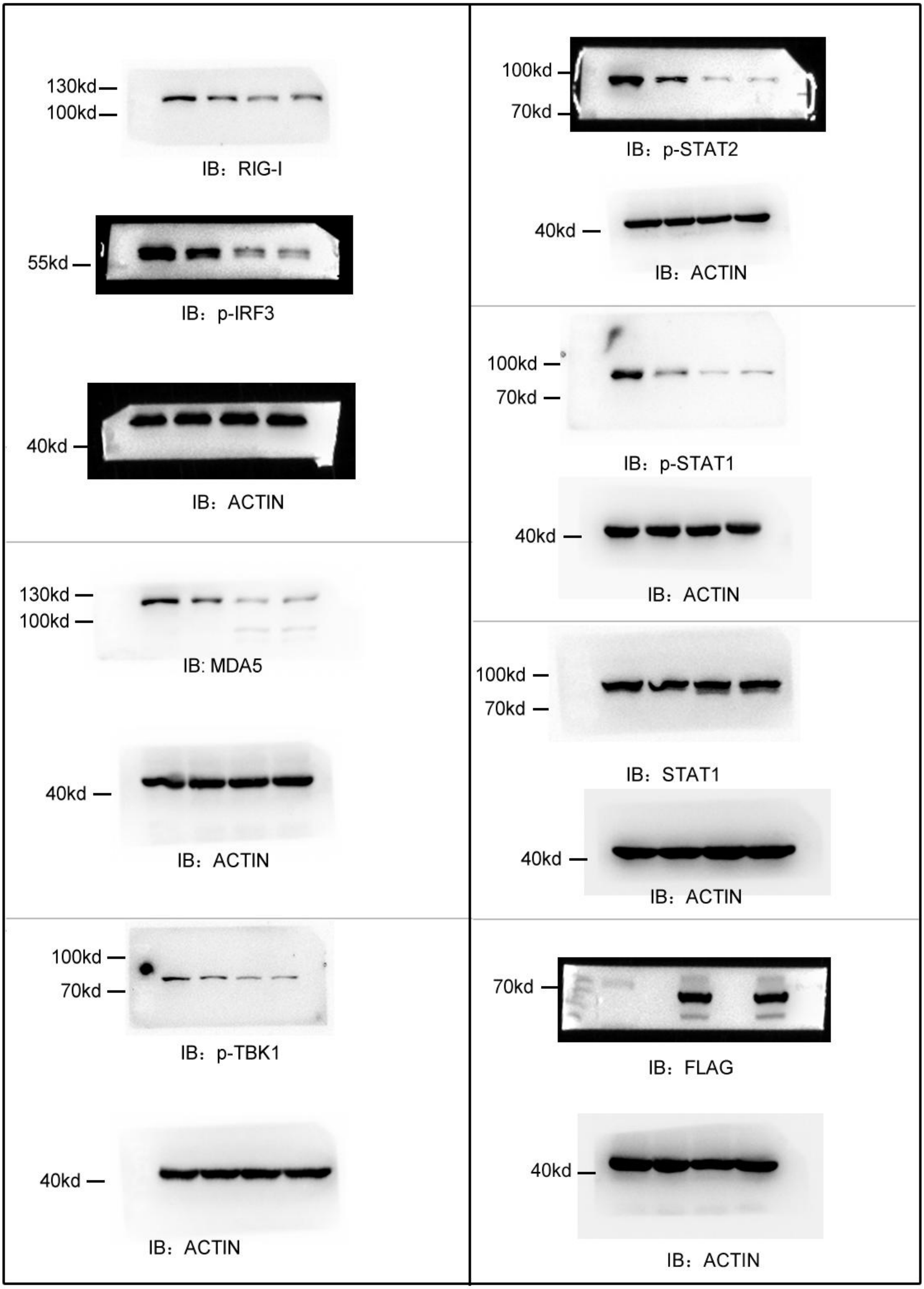

Supplementary Figure 10C

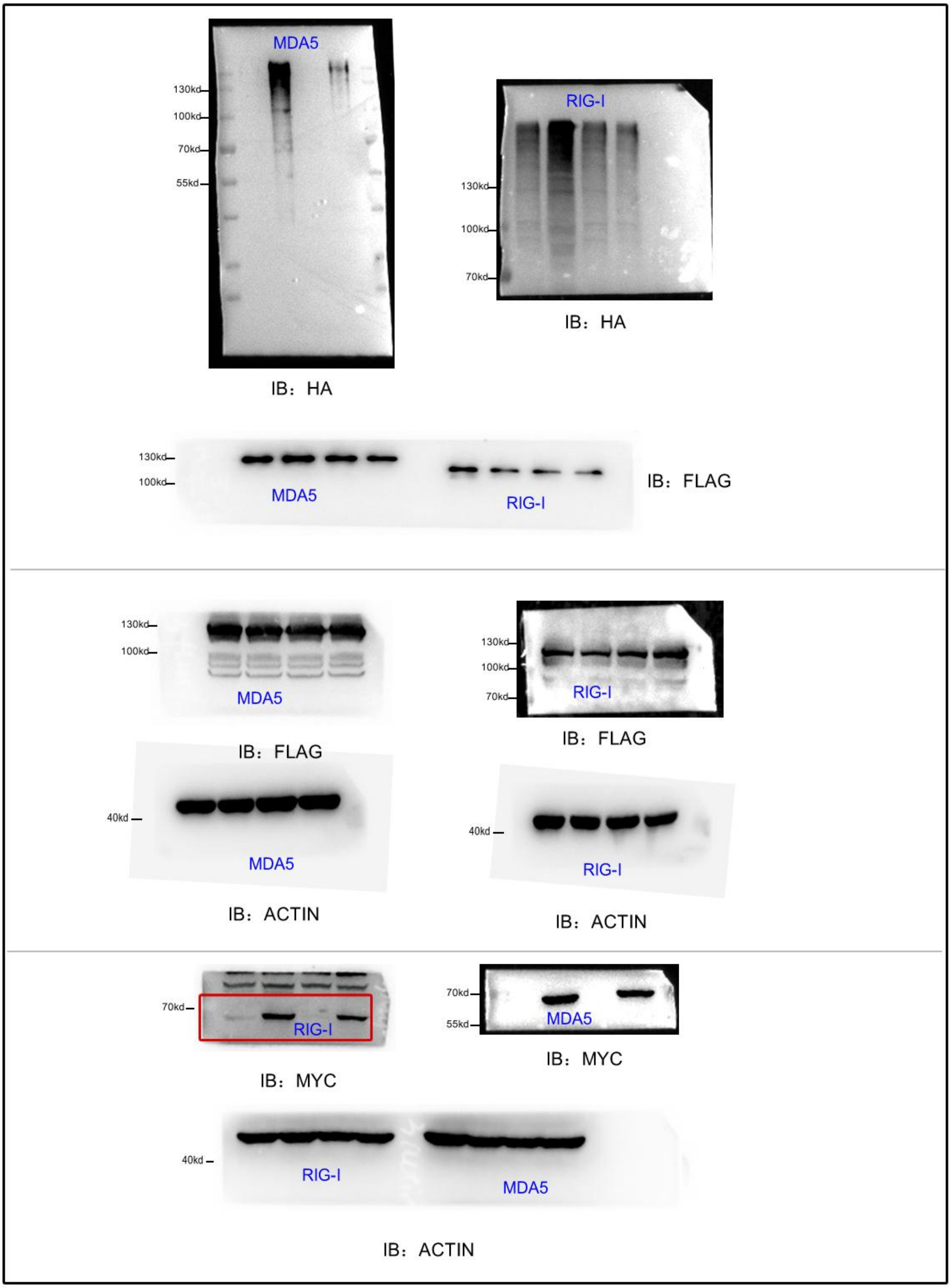

Supplementary Figure 10D

MDA5 N315-FLAG

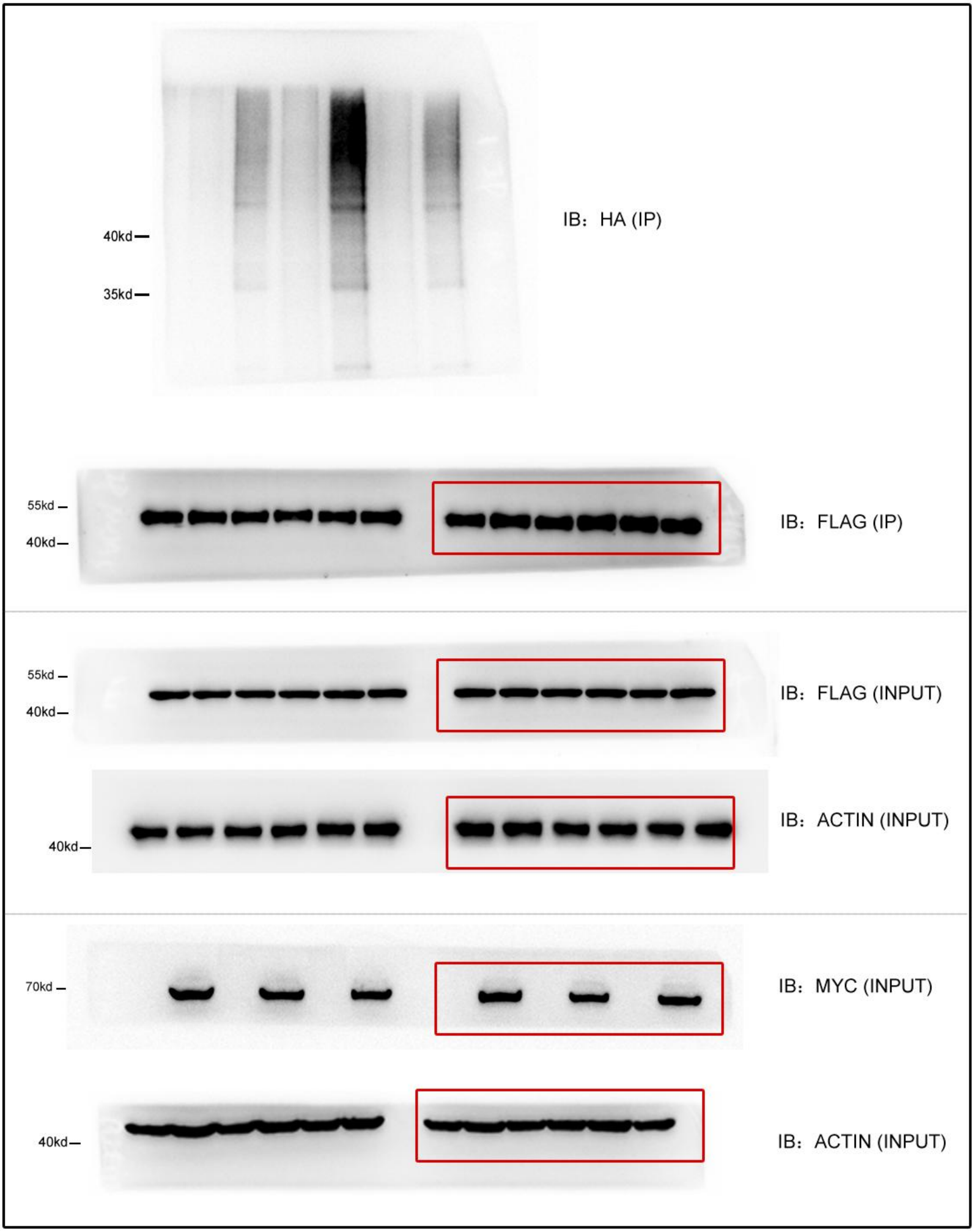

Supplementary Figure 10D

RIGI N218-FLAG

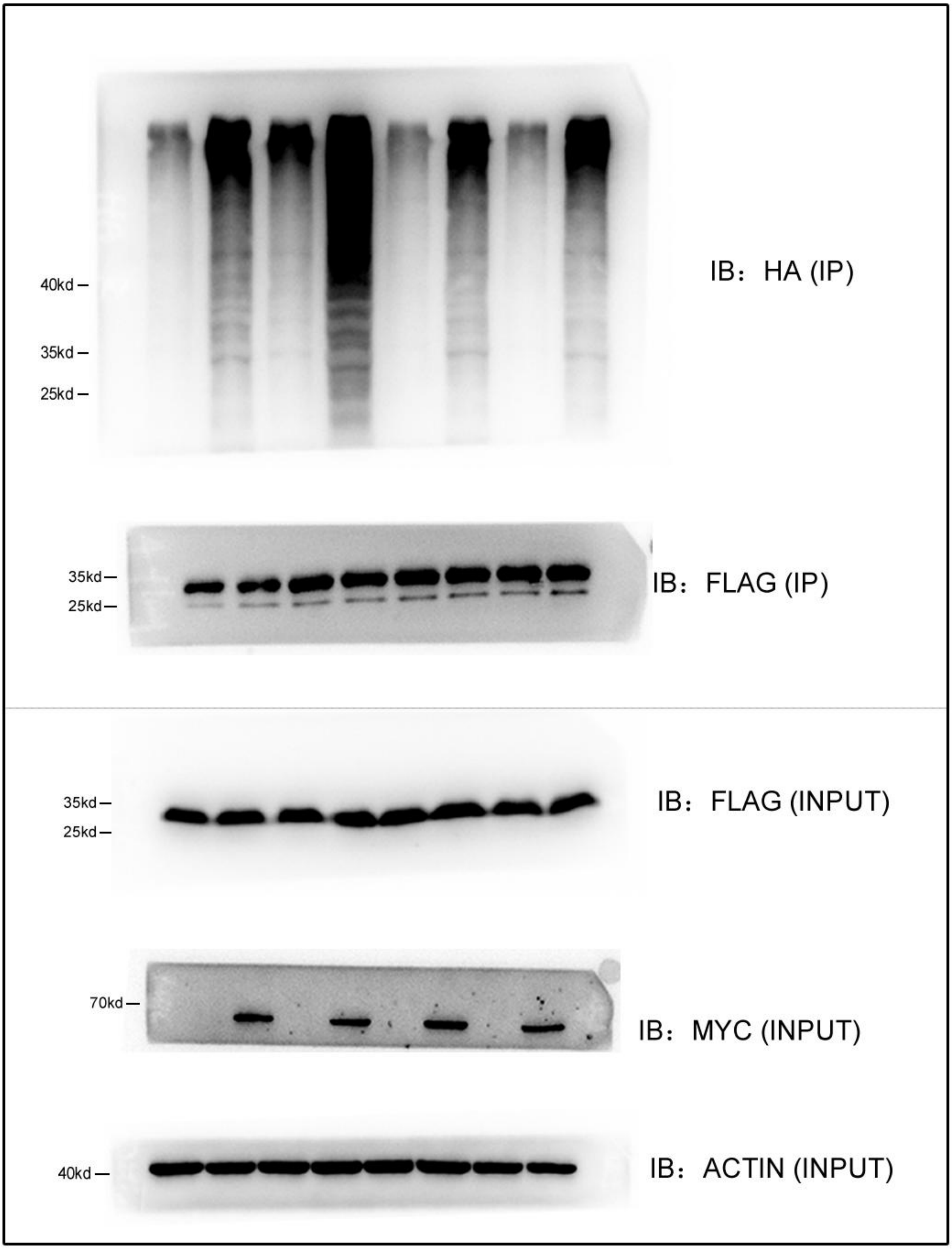

Supplementary Figure 10E

RIG-I N218-FLAG

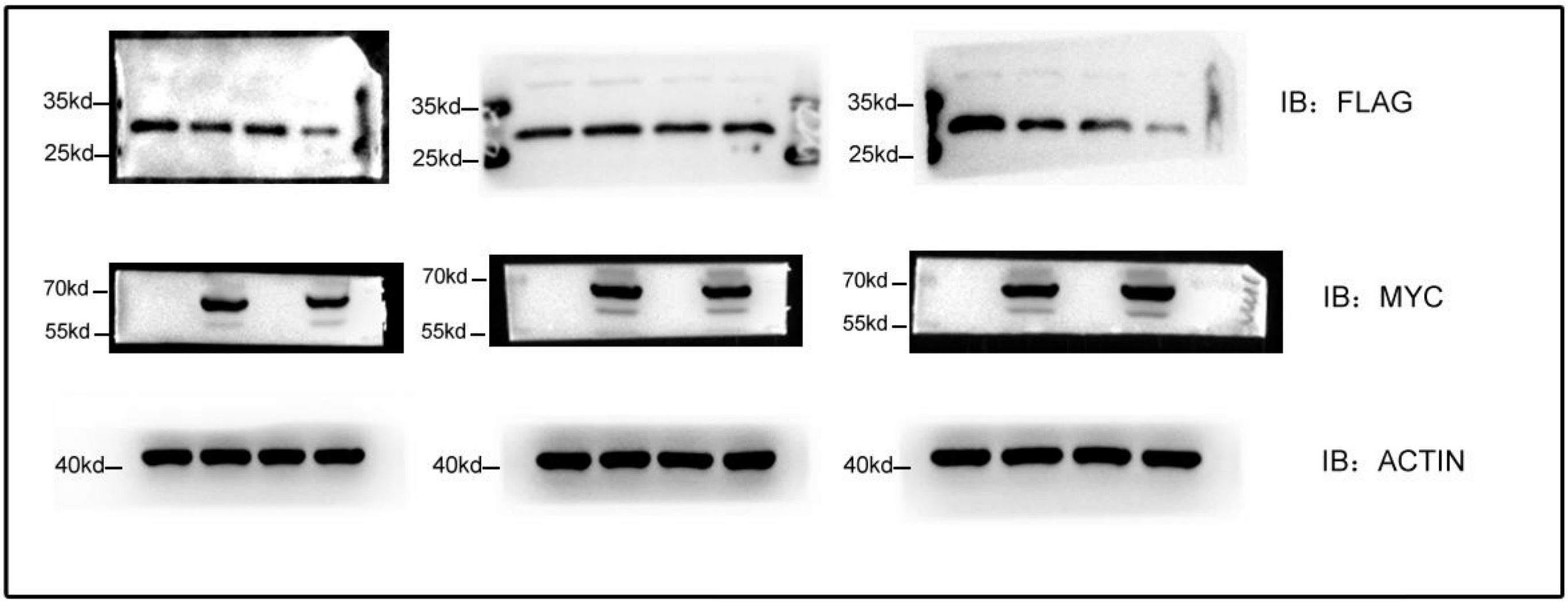

MDA5 N315-FLAG

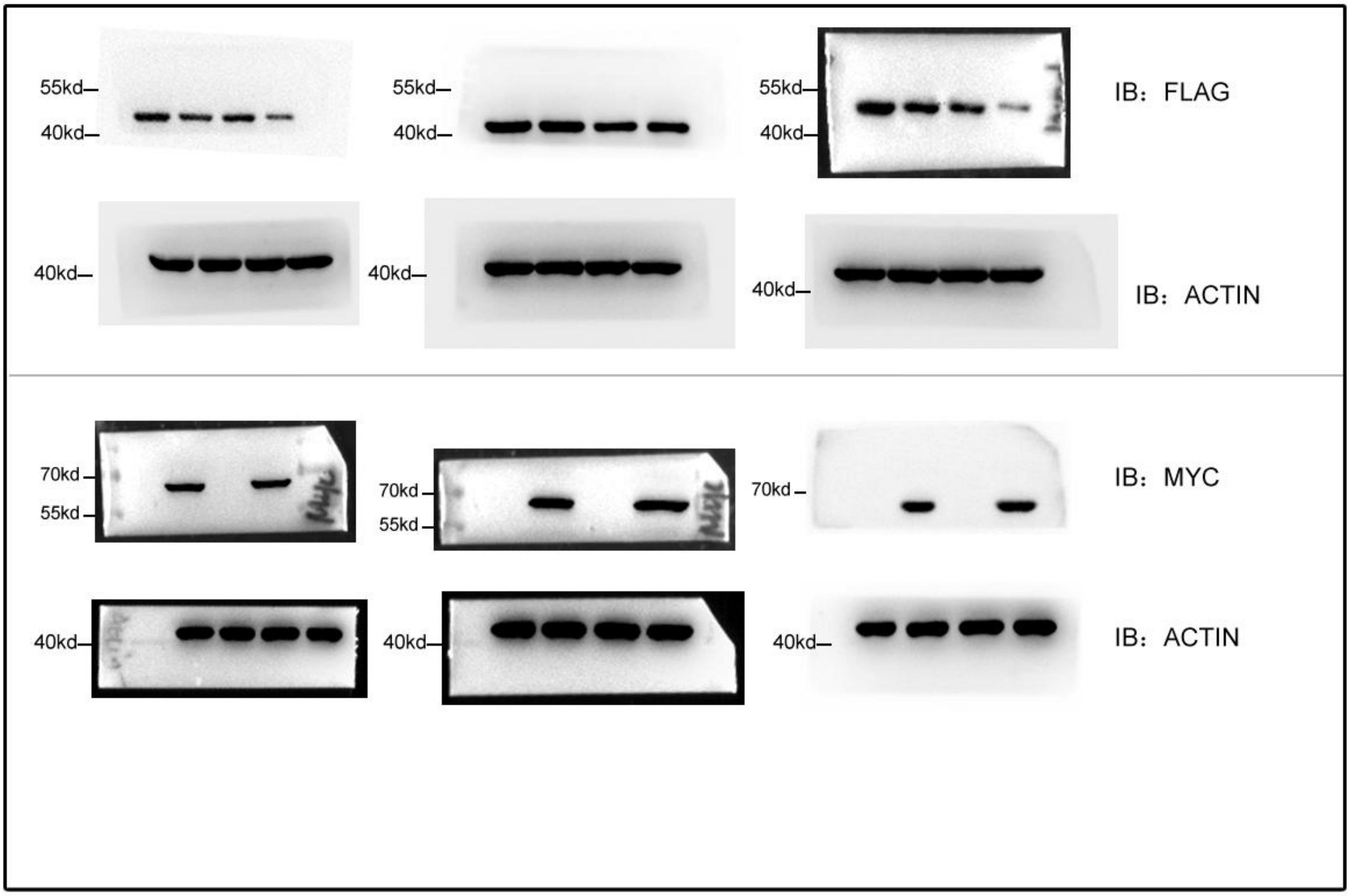

Supplementary Figure 11B

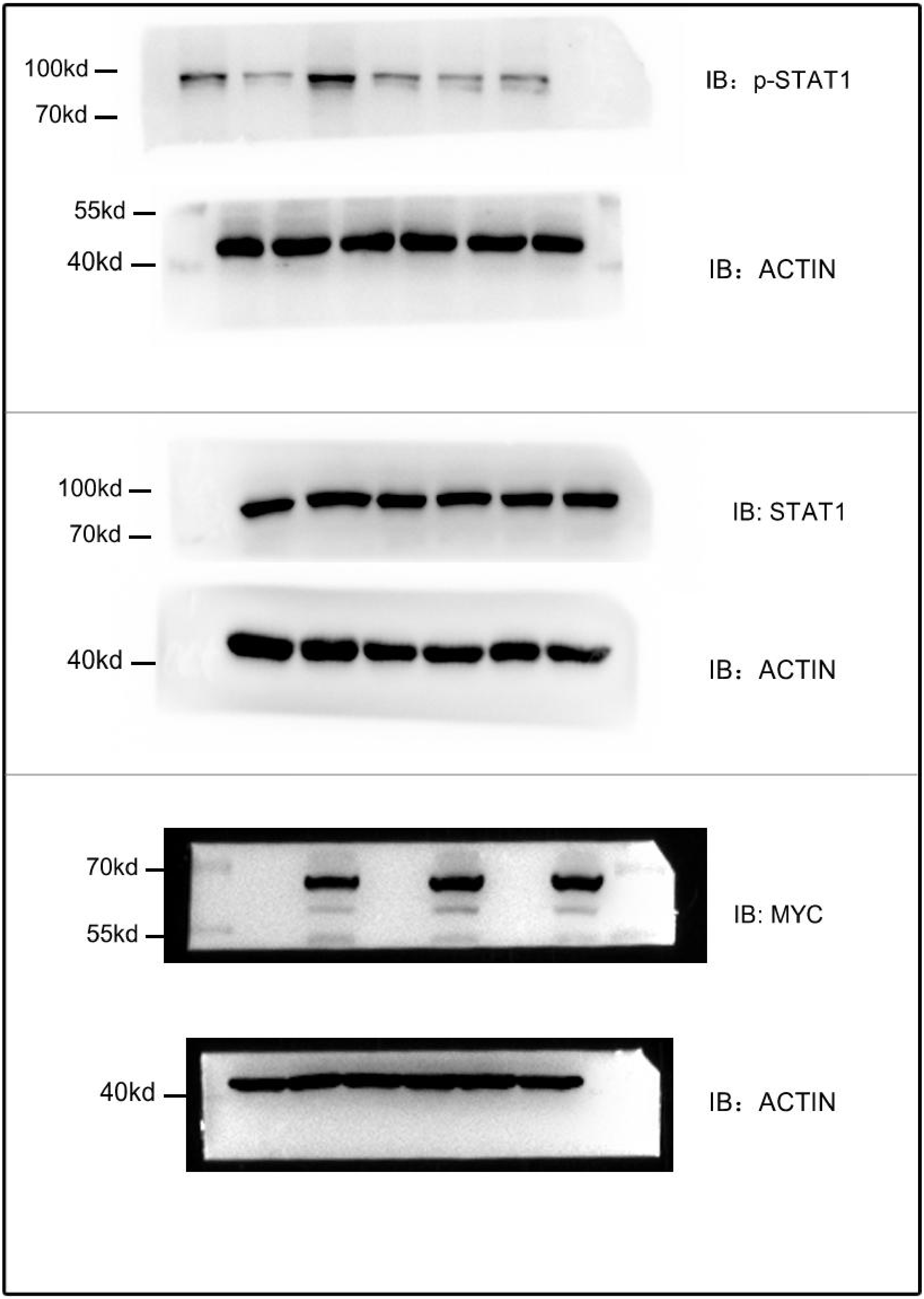

Supplementary Figure 11F

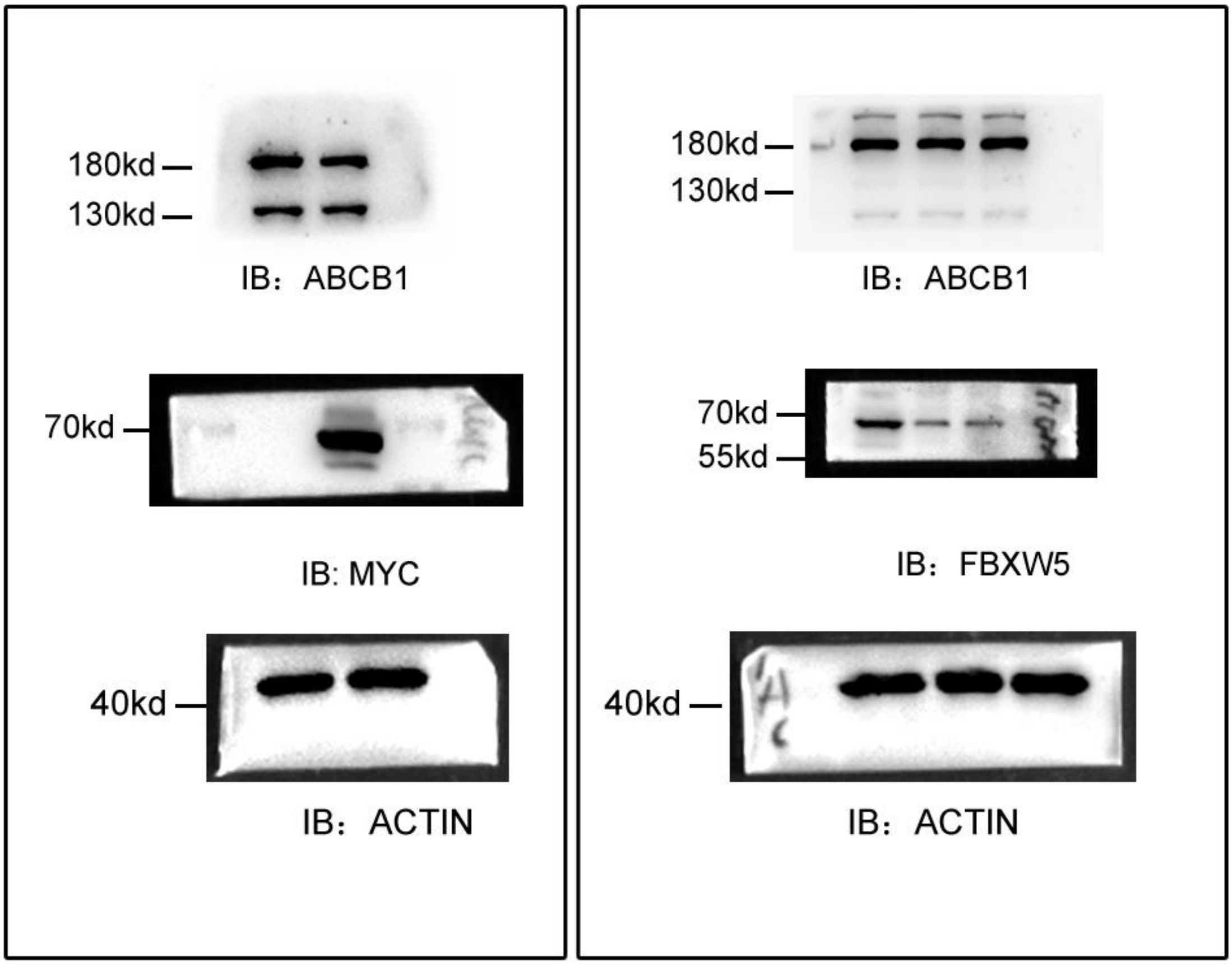

Supplement: Supplementary file 2 [file jci-136-201573-s088.pdf]
